# Catalytic Enantioselective Synthesis of *C*_1_‐ and *C*_2_‐Symmetric Spirobiindanones through Counterion‐Directed Enolate *C*‐Acylation

**DOI:** 10.1002/anie.201607731

**Published:** 2016-10-06

**Authors:** Benjamin F. Rahemtulla, Hugh F. Clark, Martin D. Smith

**Affiliations:** ^1^ Chemistry Research LaboratoryUniversity of Oxford12 Mansfield RoadOxfordOX1 3TAUK; ^2^ GlaxoSmithKline PharmaceuticalsMedicines Research CentreGunnels Wood Road, StevenageHertfordshireSG1 2NYUK

**Keywords:** axial chirality, *C*-acylation, counterion, enantioselective synthesis, organocatalysis, phase-transfer

## Abstract

A catalytic enantioselective route to C_1_‐ and C_2_‐symmetric 2,2′‐spirobiindanones has been realized through an intramolecular enolate C‐acylation. This reaction employs a chiral ammonium counterion to direct the acylation of an in situ generated ketone enolate with a pentafluorophenyl ester. This reaction constitutes the first example of a direct catalytic enantioselective C‐acylation of a ketone and provides an efficient and highly enantioselective route to axially chiral spirobiindanediones. These products can be diastereoselectively derivatized, offering access to a range of functionalized spirocyclic architectures.

β‐dicarbonyl derivatives are versatile and valuable intermediates for an enormous range of synthetic chemistry, and can be synthesized in enantioenriched form by enantioselective 1,4‐addition and alkylation reactions.^1^ In comparison, enantioselective methods for their synthesis by enolate *C*‐acylation are relatively limited.^2^ Enolate *C*‐acylation offers a valuable strategic approach to such derivatives, but is often limited by poor *O*‐ vs. *C*‐regioselectivity.^3^ One approach to solving this problem employs silyl enol ethers or silyl ketene acetals as enolate precursors,^4^ and this has led to several distinct strategies for enantioselective *C*‐acylation (Scheme 1).^5^ Fu has demonstrated that planar chiral 4‐pyrrolidinopyridine derivative **1** is highly effective in promoting the *C*‐acylation of α‐aryl ketene acetal derivatives with high levels of enantioselectivity.^6^ This reaction proceeds through the in situ formation of a chiral acylpyridinium cation and the generation of a silicon‐free enolate. A related Lewis base approach from Smith employs a chiral isothiourea^7^ **2** to mediate highly enantioselective *C*‐acylation of a cyclic silyl ketene acetal, most likely via an activated acyl‐isothiouronium ion.^8^ Jacobsen has disclosed a distinct method that utilizes a chiral thiourea **3** with a 4‐pyrrolidinopyridine co‐catalyst to effect enantioselective *C*‐acylation with acyl fluoride derivatives.^9^, ^10^ This process likely proceeds through the reaction of a thiourea‐bound enolate and a catalyst‐associated acylpyridinium ion. A very recent approach from Stoltz achieves the first intermolecular enantioselective *C*‐acylation of a carbonyl derivative that is not a ketene acetal. This process utilizes a Ni‐catalyzed three‐component coupling of lactam enolates, benzonitriles and aryl halides in the presence of ligand **4** to produce β‐imino lactams that afford β‐keto lactams upon hydrolysis.^11^ We considered that an alternative approach that did not utilize a preformed enolate derivative could be extremely valuable, but also recognized that this was likely to be challenging due to competitive *O*‐acylation, especially as the oxygen of an enolate is intrinsically the most nucleophilic site;^12^ to our knowledge no methods for the direct catalytic enantioselective *C*‐acylation of ketones have been disclosed. We rationalized that intramolecular acylation of a ketone enolate could geometrically disfavour reaction on oxygen and favour the desired *C*‐acylation. To achieve this, we propose that an enolate generated from an indanone^13^ derivative **5** could be trapped in an intramolecular fashion by an activated ester to generate chiral spirobiindanones **6**. If the enolate were to be generated under phase transfer conditions in the presence of a chiral counterion, a catalytic enantioselective route to spiro β‐dicarbonyl derivatives could be envisaged. Spiro compounds are valuable intermediates in synthesis, materials and catalysis,^14^ but catalytic enantioselective routes for their synthesis are relatively underdeveloped.^15^ Catalytic enantioselective methods for the generation of axially chiral spiro compounds are particularly rare.^16^

**Scheme 1:**
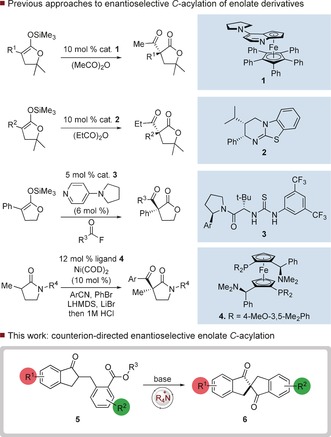
Previous work and strategy for enantioselective *C*‐acylation.

We prepared a suitable substrate **7** in which the acyl group was activated as a phenyl ester in three steps from indan‐1‐one.^17^ Upon exposure of **7** to tetrabutylammonium bromide (TBAB) and 50 % aqueous potassium carbonate in toluene at room temperature, we observed smooth cyclization to the racemic *C*_2_‐symmetric spirobiindanone **9**, with no trace of *O*‐acylation observed (Table 1, entry 1).

**Table 1:** Optimization: cation‐directed enantioselective *C*‐acylation.^[a]^ 
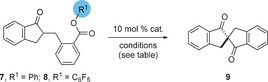

Entry	R^1^	Catalyst	Base	e.r.^[b]^
1	Ph	Bu_4_NBr	K_2_CO_3_ (aq.)^[c]^	50:50
2	Ph	**10**	K_2_CO_3_ (aq.)^[c]^	56:44
3	Ph	**10**	KOH (aq.)^[c]^	–
4	Ph	**10**	KOH (s)	59:41
5	Ph	**11**	KOH (s)	69:31
6	Ph	**11**	NaOPh (s)	51:49
7	C_6_F_5_	**11**	KOH (s)	42:58
8	C_6_F_5_	**12**	KOH (s)	79:21
9	C_6_F_5_	**13**	KOH (s)	25:75
10	C_6_F_5_	**14**	KOH (s)	36:64
11	C_6_F_5_	**15**	KOH (s)	29:71
12	C_6_F_5_	**16**	KOH (s)	17:83
13	C_6_F_5_	**17**	KOH (s)	88:12
14	C_6_F_5_	**17**	K_3_PO_4_ (s)	89:11
15	C_6_F_5_	**17**	K_3_PO_4_ (aq.)^[c]^	97:3

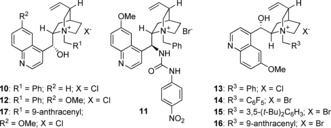				

[a] Conditions: substrate **7** or **8** (0.02 mmol), catalyst (10 mol %), solid base (1.0 equiv.), PhMe ([substrate]=0.1 mol dm^−3^), RT, 48 h. [b] e.r. determined by chiral stationary phase HPLC. [c] base: 50 % aq., w/w, 10.0 equiv.

Use of *N*‐benzyl cinchonidinium chloride **10** in place of TBAB gave complete conversion to the desired product and a modest e.r. of 56:44. We observed that the use of aqueous hydroxide bases led to hydrolysis of the phenyl ester **7** (entry 3) and hence we switched to solid bases in order to screen catalysts for higher enantioselectivity. This led to a small increase in selectivity (to 59:41 e.r., entry 4) and under these conditions no hydrolysis was observed. We subsequently screened a range of ammonium salts and under these conditions, **11**, which bears a urea as a Brønsted acidic group, was found to give improved selectivity of 69:31 e.r. (entry 5). We were concerned that the relatively low selectivities observed were a consequence of a background reaction mediated by the basic phenolate leaving group, and hence we examined a reaction with a stoichiometric amount of sodium phenolate in the presence of catalyst **11** (entry 6). This led to very low enantioselectivity (51: 49 e.r.), which was consistent with our hypothesis. Consequently, we changed our substrate to pentafluorophenyl ester **8**, rationalizing that the lower p*K*_a_ of pentafluorophenol to phenol (19.5 vs. 27.4 in MeCN)^18^ would limit this background reaction.^19^ Subjecting this substrate **8** to the same conditions (entry 7) led to a relatively poor 42:58 e.r. but a significantly slower reaction time, and hence we decided to reoptimize the catalyst for this substrate.^17^ Changing to quininium catalyst **12** led to a significantly higher selectivity (79:21 e.r., entry 8), and pseudoenantiomer **13** led to a very similar 25:75 e.r. and the expected reversal of absolute configuration (entry 9). We subsequently examined a range of different *N*‐substituents on the quinidinium nucleus; *N*‐pentafluorophenylbenzyl catalyst **14** gave poorer selectivity (36:64 e.r., entry 10) but we observed that selectivity improved with larger *N*‐substituents (29:71 e.r. with 3,5‐di‐*tert*‐butyl derivative **15**, and 17:83 e.r. with 9‐anthracenyl catalyst **16**, entries 11 and 12 respectively). Quininium catalyst **17** gave better e.r. (88:12), and a switch to a weaker base with solid K_3_PO_4_ gave similar e.r. (89:11; entry 13). Changing to aqueous K_3_PO_4_ led to an increase in e.r. (to 97:3) without the issues of ester hydrolysis that plagued our earlier attempts to use aqueous base. We believe this to be a rare example of a catalytic enantioselective *C*‐acylation of an enolate.^20^

With an optimized procedure in hand, we examined the scope and limitations of this reaction (Table 2). The unsubstituted *C*_2_‐symmetric spirobiindanone **9** can be produced in 93 % yield and 97:3 e.r. via this approach; this cyclization can be performed on a gram scale without compromising yield or enantioselectivity. The absolute configuration of this material was confirmed to be (*S*)‐ through comparison with literature data.21a Substitution is tolerated on both the indan‐1‐one and benzoic acid sections of substrates; thus *C*_2_‐symmetric dimethyl **18** (92:8 e.r., 84 % yield) and bistrifluoromethyl spiro derivatives **19** (95:5 e.r., 95 % yield) are both produced with excellent enantioselectivity and yield. This represents a novel method for the catalytic enantioselective synthesis of these axially chiral motifs; other methods rely on resolution or the use of chiral auxiliaries.^21^ The reaction is not limited to *C*_2_‐symmetric products and we have explored substitution at all sp^2^ positions on the 1‐indanone ring. Cyclization of substrates bearing substituents in the 5‐position of the indanone ring occured with high levels of enantioselectivity to afford **20** (5‐bromo) and **21** (5‐fluoro) with consistently high levels of enantioselectivity (96:4 e.r. in both cases). The absolute configuration of bromide **20** was confirmed through X‐ray crystallography.^22^ An indanone substrate substituted with an electron‐donating substituent in the 4‐position cyclized to afford methoxy derivative **22** in 99 % yield and 94:6 e.r. Similarly, cyclization of a 4‐bromo indanone to give **23** occurred in 93:7 e.r. We have also examined the formation of spirobiindanones with different groups on each of the two aromatic rings such as **24**, which can be synthesized in 99 % yield and 95:5 e.r. Reaction of an indanone containing a 7‐trifluoromethyl group occurred smoothly under the optimized conditions to give **25** (95:5 e.r. and 88 % yield). Monosubstituted derivatives such as **26** and **27** can be made by the reaction of precursors bearing a group on either the indanone or the benzoic acid portion of the precursor. Consequently, we investigated whether the enantioselectivity observed is different via cyclization from these two distinct starting materials. For the formation of trifluoromethyl substituted compound **26**, there are small differences between the two approaches, with higher selectivity observed with the substituent on the indanone (95:5 e.r. vs. 93:7 e.r.). This small difference was also observed in the formation of **27**, where cyclization from the precursor carrying the methyl group on the indanone cyclized in 99:1 e.r. and 71 % yield versus 96:4 e.r. and 99 % yield from the substituted benzoate derivative. The observed difference in yield is likely a consequence of a significantly slower cyclization from the 6‐substituted indanone.

**Table 2:** Scope of cation‐directed enantioselective direct *C*‐acylation. 
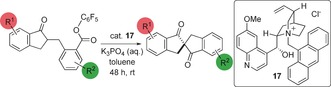

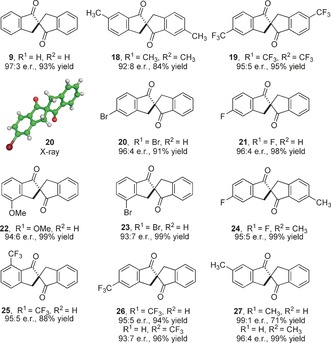

Conditions: substrate (0.10 mmol), catalyst (10 mol %), K_3_PO_4_ (50 % aq., w/w, 10.0 equiv.), PhMe ([substrate]=0.1 mol dm^−3^), RT, 48 h. e.r. determined by chiral stationary phase HPLC; yields refer to isolated materials.

We subsequently examined whether these spirobiindanones could be chemoselectively derivatized to afford access to unrealized spirocyclic architectures (Scheme 2). Radical bromination of unsubstituted spirobiindanone **9** with *N*‐bromosuccinimide in the presence of AIBN as initiator led to clean monobromination in the benzylic position to afford **28** as a single diastereoisomer in 83 % yield and without any loss of enantiopurity (at 97:3 e.r.). Stereoselective reduction of spirobindanones has been reported previously on racemic material.21e Under the reported conditions, of DIBALH and *t*‐BuLi in THF, reduction of the 1,3‐diketone **9** to diol **29** proceeded with complete diastereoselectivity and without compromising e.r. (97:3 e.r. (major), >20:1:1 d.r., (*S*,*S*,*S*):(*R*,*S*,*S*):(*R*,*S*,*R*)). Temperature control in this transformation is key; allowing the reaction to warm above −78 °C led to significantly lower e.r., presumably through reversible retro‐aldol condensation on mono‐reduced material. We also observed that if an excess of DIBALH in hexane (rather than THF) was employed, an enhancement of e.r. in the major diastereoisomer was observed (from 97:3 to >99:1 e.r.), whilst a reduction in diastereoselectivity (to 7:1:0 d.r., (*S*,*S*,*S*):(*R*,*S*,*S*):(*R*,*S*,*R*)) was attained. We attribute this to a modification of the reducing agent with enantioenriched diol in situ, which leads to discrimination between the major and minor enantiomer in **8** by a chiral non‐racemic reducing agent.^23^ This leads to an augmentation of enantioselectivity (for the major diastereoisomer) at the expense of overall diastereoselectivity.

**Scheme 2:**
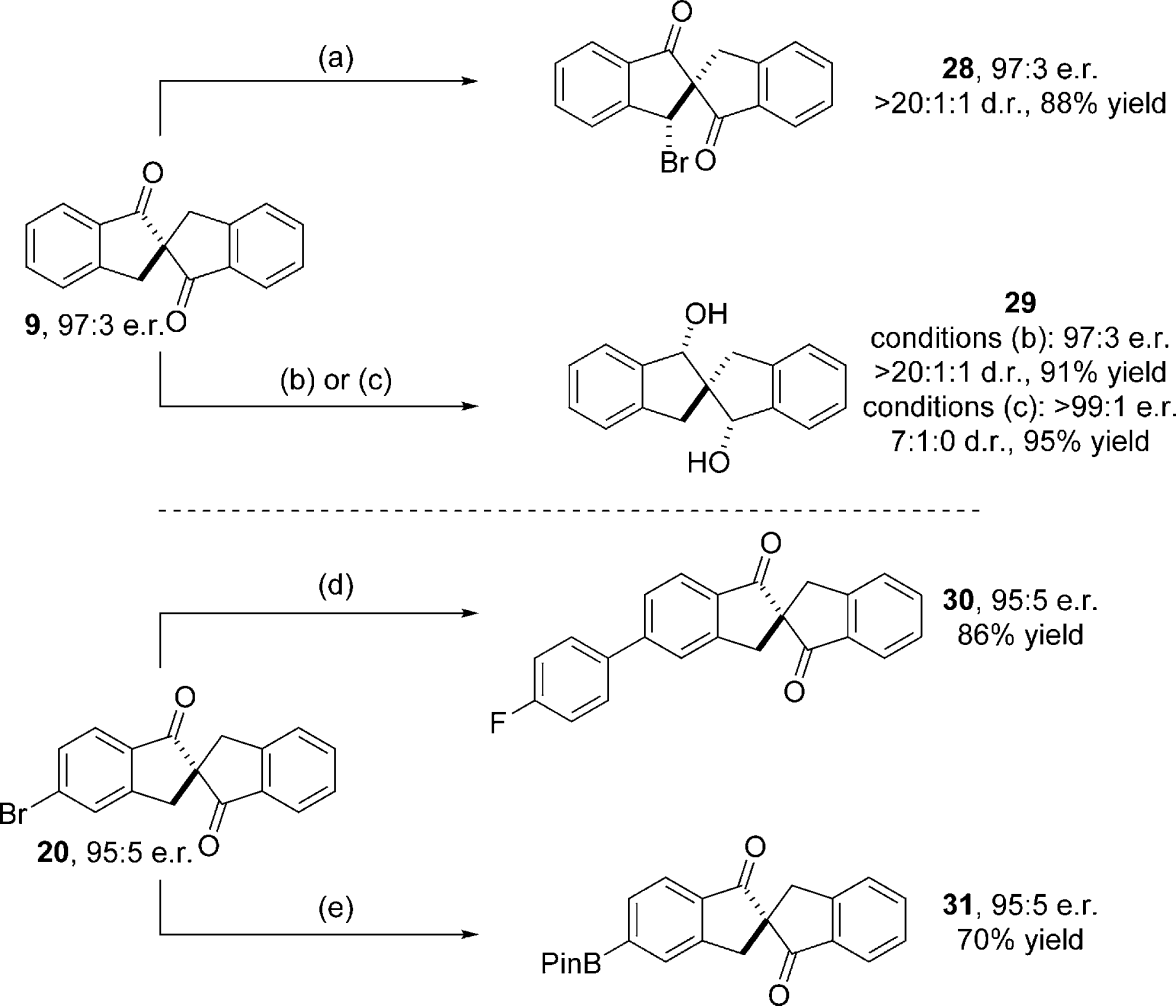
Chemoselective derivatizations of the spirobindanone core. Conditions: a) *N*‐Bromosuccinimide (1.0 equiv.), AIBN (0.02 equiv.), CCl_4_, 80 °C. b) DIBALH (1.0 m in THF, 3.0 equiv.), *t*‐BuLi (1.7 m in pentane, 3.0 equiv.), THF, −78 °C. c) DIBALH (1.0 m in hexanes, 3.3 equiv.), *t*‐BuLi (1.7 m in pentane, 3 equiv.), THF, −78 °C. d) 4‐F(C_6_H_4_)B(OH)_2_ (1.5 equiv.), Pd(PPh_3_)_4_ (2 mol %), Na_2_CO_3_, Toluene/H_2_O (2.3:1, v/v), reflux. e) B_2_Pin_2_ (1.2 equiv.), Pd(dppf)Cl_2_ (5 mol %), KOAc (3.0 equiv.), 1,4‐dioxane, reflux. e.r. determined by chiral stationary phase HPLC; d.r. determined by examination of the crude ^1^H NMR spectra. Yields refer to isolated materials.

Ultimately this means that **29**, which has been employed as a building block in ligand design and construction,21g can be isolated as a single diastereoisomer in 78 % yield and >99:1 e.r. We have also demonstrated that non‐symmetrical spirobindanone **20**, which contains an aryl bromide, can be cross‐coupled with 4‐fluorophenylboronic acid to afford biaryl **30** in 86 % yield and 95:5 e.r.; similarly, the carbon‐bromine bond in **20** can be transformed to a carbon‐boron bond in good yield (70 %) and without compromising enantiointegrity (95:5 e.r.).

In conclusion, we have demonstrated an approach to direct catalytic enantioselective *C*‐acylation of a ketone using a chiral counterion. This approach has been exemplified in the synthesis of both *C*_1_‐ and *C*_2_‐symmetric spirobiindanediones but will likely be applicable to other highly functionalized three‐dimensional scaffolds. We have also shown that these materials can be chemoselectively derivatized; in the case of diastereoselective reduction this led to an increase in enantioenrichment to >99:1 e.r., offering a practical route to a valuable scaffold for ligand and catalyst design.

## Supporting information

As a service to our authors and readers, this journal provides supporting information supplied by the authors. Such materials are peer reviewed and may be re‐organized for online delivery, but are not copy‐edited or typeset. Technical support issues arising from supporting information (other than missing files) should be addressed to the authors.

Supplementary
