# Supplementary material for: Catalytic Enantioselective Synthesis of C 1‐ and C 2‐Symmetric Spirobiindanones through Counterion‐Directed Enolate C‐Acylation
Source: Angew Chem Int Ed Engl. 2016 Oct 6;55(42):13180–3. doi: 10.1002/anie.201607731 (PMC5113699; doi:10.1002/anie.201607731)
Supplement: Supplementary file 1 — Supplementary [file ANIE-55-13180-s001.pdf]

Angewandte Chemie
Eine Zeitschrift der Gesellschaft Deutscher Chemiker

Supporting Information

# Catalytic Enantioselective Synthesis of $C_1$- and $C_2$-Symmetric Spirobiindanones through Counterion-Directed Enolate $C$-Acylation

*Benjamin F. Rahemtulla, Hugh F. Clark, and Martin D. Smith**

anie_201607731_sm_miscellaneous_information.pdf

# Supporting Information

## Contents


**1.1 General Information** S1

**1.2 General Experimental Procedures** S2

**1.3 Catalyst Synthesis** S4

**1.4 Supplemental Optimization Table** S5

**1.5 Experimental Procedures** S6

**1.5.1 Starting Material Synthesis** S6

**1.5.2 Enantioselective *C*-acylation** S30

**1.5.3 Derivatization** S40

**1.6 Determination of Absolute Stereochemistry** S47

**1.7 References** S48

**1.8 NMR Spectra and HPLC Traces** S50

## 1.1 General Information

Reactions requiring moisture-sensitive reagents were carried out in flame-dried glassware, under an atmosphere of argon (balloon pressure). Dichloromethane, tetrahydrofuran and toluene were purified by filtration through activated alumina columns employing the method of Grubbs *et al.*[1] Water was purified by an Elix® UV-10 system. Reagents were used directly as supplied by major chemical suppliers, or following purification procedures described by Perrin and Armarego.[2] Petrol 40-60 refers to the fraction of petroleum ether which boils in the range 40-60 °C. Brine refers to a saturated aqueous solution of sodium chloride.

Silica gel chromatography was carried out using Merck Geduran® Silicagel (40-63 μm particle size). Thin layer chromatography (TLC) was carried out using pre-coated, aluminium backed plates (Merck Kieselgel 60 F254). Visualisation was achieved with ultraviolet irradiation (254 nm) and staining with permanganate.

NMR spectroscopy was carried out using Bruker Avance spectrometers in the deuterated solvent stated, using the residual non-deuterated solvent signal as an internal reference ($^{1}$H NMR: $CDCl_3$ (7.26), $(CD_3)_2SO$ (2.50); $^{13}$C NMR: $CDCl_3$ (77.16), $(CD_3)_2SO$ (39.52); $^{19}$F NMR: $CFCl_3$ (0.00)). Chemical shifts are quoted in ppm, based on appearance rather than interpretation. Signal patterns are indicated as: br s, broad singlet; s, singlet; d, doublet; t, triplet; q, quartet; m, multiplet. Coupling constants, *J*, are quoted to the nearest 0.1 Hz and are presented as observed. Diastereotopic protons are denoted by letters, e.g. $H_A$, $H_B$ etc.
All $^{19}$F NMR spectra are reported as proton/fluorine decoupled unless otherwise stated.
Due to significant (C-F) coupling and unresolved fine structure, perfluorophenyl $^{13}$C peaks are reported only when clearly resolved.

Infrared spectra were prepared as a neat film and were recorded using a Bruker Tensor 27 FTIR spectrometer using an ATR module.

Low resolution mass spectrometry was carried out using ESI and was performed on a Micromass LCT Premier Spectrometer. HRMS was carried out using Bruker MicroTDF and Micromass GCT spectrometers under electrospray ionization (ESI) or ammonia chemical ionization (ACI)/electron ionization (EI) conditions respectively.

Analytical chiral HPLC was carried out on a Dionex UltiMate 3000 HPLC system comprising a Dionex LPG-3400A pump, WPS-3000SL autosampler and TCC-3000SD column

compartment, and a Daicel Chiralpak column (0.46 cm × 25 cm), equipped with an appropriate guard column (0.4 cm × 1 cm).

Melting points were determined using a Reichert melting point apparatus and are uncorrected,

Optical rotations were recorded on a Schmidt-Haensch Unipol L2000 polarimeter and values are quoted [° mL $g^{-1}$ $dm^{-1}$]. Concentrations are quoted in g/100 mL.

## 1.2 General Experimental Procedures

### General Procedure A: Aldol Condensation of 1-Indanones with *o*-Carboxybenzaldehydes[3]

**Aldol condensation:** To a stirred room temperature solution of *the appropriate 1-indanone* (1.0 eq.) and *the appropriate o-carboxybenzaldehyde* (1.0 eq.) in ethanol (2.4 mL/mmol indanone) was added aqueous sodium hydroxide (1 M, 1.8 eq.). The reaction mixture was stirred for 15 minutes after which cold water was added and the resulting solution poured onto diethyl ether. The aqueous layer was separated and the organic layer extracted with water. The combined aqueous extracts were acidified with aqueous sulfuric acid (2.5 M) and the precipitate isolated *via* filtration, then dried under high vacuum to afford the corresponding unsaturated acid.

### General Procedure B: Esterification of Unsaturated Acids

**Esterification of unsaturated acids:** To a stirred suspension of *the appropriate crude unsaturated acid* (1.0 eq.) in dichloromethane (3.0 mL/mmol acid) was added oxalyl chloride (1.5 eq.) followed by 1 drop of *N*,*N*-dimethylformamide. After evolution of gas had ceased (30 minutes), the mixture was concentrated under reduced pressure. The residue was subsequently dissolved in dichloromethane (3.0 mL/mmol acid) and to this solution was added pentafluorophenol (1.0 eq.) and triethylamine (3.0 eq.). The reaction mixture was stirred at room temperature for 2 hours upon which it was quenched by addition of water and diluted in dichloromethane. The organic layer was separated and the aqueous layer extracted twice with dichloromethane. The combined organic extracts were dried over anhydrous magnesium sulfate and concentrated under reduced pressure. Purification of the residue *via* flash chromatography (see experimental methods section for specific details) afforded the corresponding unsaturated activated ester.

**General Procedure C: Hydrogenation of Unsaturated Acids or Activated Esters**[3]

A stirred suspension of *the corresponding unsaturated acid or activated ester* (1.0 eq.) and palladium on carbon (10 wt. %) (0.1 mg/mg *unsaturated acid or ester*) *or* platinum(IV) oxide (0.1 mg/mg *unsaturated acid or ester*) in ethyl acetate (7.0 mL/mmol acid *or* ester) was purged with hydrogen (1 balloon). After 10 minutes, purging was stopped and the balloon was replaced with a new balloon of hydrogen. Once the starting material had been consumed (indicated by TLC analysis) the reaction flask was opened to air, the reaction mixture filtered through Celite® with dichloromethane and the resulting solution concentrated under reduced pressure. Purification of the residue *via* flash chromatography *or* recrystallization (see experimental methods section for specific details) afforded the corresponding saturated acid/activated ester.

**General Procedure D: Asymmetric Phase-Transfer *C*-Acylation of Activated Esters**

In a 7 mL screw-topped vial, *the appropriate saturated activated ester* (1.0 eq.) and **17** (0.10 eq.) were dissolved in toluene (10 mL/mmol ester) and 50% w/w aqueous potassium phosphate (10.0 eq.) was added. The biphasic reaction mixture was rapidly stirred at room temperature for *the appropriate time* and then water and dichloromethane were added. The organic layer was separated and the aqueous layer was extracted twice with dichloromethane. The combined organic extracts were dried over anhydrous magnesium sulfate, filtered and concentrated under reduced pressure. Purification of the residue *via* flash chromatography (see experimental methods section for specific details) afforded the corresponding enantioenriched spirobiindanone.

**General Procedure E: Racemic Phase-Transfer *C*-Acylation of Activated Esters**

*The appropriate saturated activated ester* (1.0 eq.) and tetrabutylammonium bromide (0.10 eq.) were dissolved in toluene (10 mL/mmol ester) and potassium hydroxide (2.0 eq.) was added. The biphasic reaction mixture was rapidly stirred at room temperature for *the appropriate time* and then water and dichloromethane were added. The organic layer was separated and the aqueous layer was extracted twice with dichloromethane. The combined organic extracts were dried over anhydrous magnesium sulfate, filtered and concentrated under reduced pressure. Purification of the residue *via* flash chromatography (see experimental methods section for specific details) afforded the corresponding racemic spirobiindanone.

## 1.3 Catalyst Synthesis

Catalysts that were not commercially available were prepared following well established literature procedures of *N*-alkylation of cinchona alkaloids.[4]

## 1.4 Supplemental Optimization Table

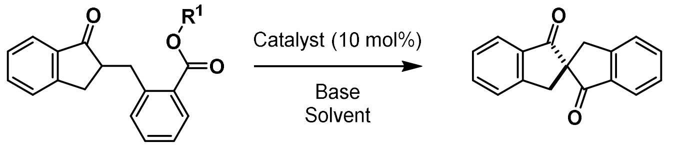


**7**. $R^1$ = Ph; **8**. $R^1$ = $C_6F_5$ **9**

| Entry | $R^1$ | Catalyst | Base (eq.) | Solvent (M) | e.r.[b] |
|---|---|---|---|---|---|
| 1 | Ph | $Bu_4NBr$ | $K_2CO_3$ (10) (aq) [c] | PhMe (0.1) | 50:50 |
| 2 | Ph | **10** | $K_2CO_3$ (10) (aq) [c] | PhMe (0.1) | 56:44 |
| 3 | Ph | **10** | KOH (10) (aq) [c] | PhMe (0.1) | - |
| 4 | Ph | **10** | KOH (1) (s) | PhMe (0.1) | 59:41 |
| 5 | Ph | **10** | $CsOH{\cdot}H_2O$ (1) (s) | PhMe (0.1) | 54:46 |
| 6 | Ph | **32** | KOH (1) (s) | PhMe (0.1) | 48:52 |
| 7 | Ph | **12** | KOH (1) (s) | PhMe (0.1) | 54:46 |
| 8 | Ph | **13** | KOH (1) (s) | PhMe (0.1) | 43:57 |
| 9 | Ph | **33** | KOH (1) (s) | PhMe (0.1) | 65:35 |
| 10 | Ph | **11** | KOH (1) (s) | PhMe (0.1) | 69:31 |
| 11 | Ph | **11** | $NaOPh{\cdot}H_2O$ (1) (s) | PhMe (0.1) | 51:49 |
| 12 | Ph | **11** | KOH (0.1) (s) | PhMe (0.1) | 63:37 |
| 13 | Ph | **11** | $NaOPh{\cdot}H_2O$ (0.1) (s) | PhMe (0.1) | 61:39 |
| 14 | $C_6F_5$ | **11** | KOH (1) (s) | PhMe (0.1) | 42:58 |
| 15 | $C_6F_5$ | **10** | KOH (1) (s) | PhMe (0.1) | 75:25 |
| 16 | $C_6F_5$ | **32** | KOH (1) (s) | PhMe (0.1) | 27:73 |
| 17 | $C_6F_5$ | **12** | KOH (1) (s) | PhMe (0.1) | 79:21 |
| 18 | $C_6F_5$ | **13** | KOH (1) (s) | PhMe (0.1) | 25:75 |
| 19 | $C_6F_5$ | **14** | KOH (1) (s) | PhMe (0.1) | 36:64 |
| 20 | $C_6F_5$ | **15** | KOH (1) (s) | PhMe (0.1) | 29:71 |
| 21 | $C_6F_5$ | **16** | KOH (1) (s) | PhMe (0.1) | 17:83 |
| 22 | $C_6F_5$ | **33** | KOH (1) (s) | PhMe (0.1) | 85:15 |
| 23 | $C_6F_5$ | **34** | KOH (1) (s) | PhMe (0.1) | 80:20 |
| 24 | $C_6F_5$ | **35** | KOH (1) (s) | PhMe (0.1) | 87:13 |
| 25 | $C_6F_5$ | **17** | KOH (1) (s) | PhMe (0.1) | 88:12 |
| 26 | $C_6F_5$ | **17** | $CsOH{\cdot}H_2O$ (2) (s) | PhMe (0.1) | - |
| 27 | $C_6F_5$ | **17** | $K_2CO_3$ (1) (s) | PhMe (0.1) | 95:5 |
| 28 | $C_6F_5$ | **17** | $K_2CO_3$ (10) (aq) [c] | PhMe (0.1) | 96:4 |
| 29 | $C_6F_5$ | **17** | $Cs_2CO_3$ (10) (aq) [c] | PhMe (0.1) | 93:7 |
| 30 | $C_6F_5$ | **17** | $K_3PO_4$ (2) (s) | PhMe (0.1) | 89:11 |
| 31 | $C_6F_5$ | **17** | $K_3PO_4$ (10) (aq) [c] | PhMe (0.1) | 97:3 |
| 32 | $C_6F_5$ | **17** | $K_3PO_4$ (10) (aq) [c] | $CH_2Cl_2$ (0.1) | 89:11 |
| 33 | $C_6F_5$ | **17** | $K_3PO_4$ (10) (aq) [c] | $^i\mathrm{Pr_2O}$ (0.1) | 81:19 |
| 34 | $C_6F_5$ | **17** | $K_3PO_4$ (10) (aq) [c] | PhH (0.1) | 95:5 |
| 35 | $C_6F_5$ | **17** | $K_3PO_4$ (10) (aq) [c] | PhMe (1) | 89:11 |

[a] Conditions: substrate **7** or **8** (0.02 mmol), Catalyst (10 mol%), Base, Solvent, r.t., 48 h. [b] e.r. determined by chiral stationary phase HPLC. [c] Base: 50% aq., w/w.

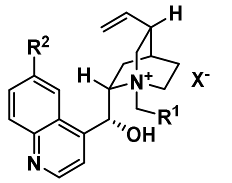


**10**: $R^1$ = Ph, $R^2$ = H, X = Cl
**12**: $R^1$ = Ph, $R^2$ = OMe, X = Cl
**17**: $R^1$ = 9-anthracenyl, $R^2$ = OMe, X = Cl
**33**: $R^1$ = 9-anthracenyl, $R^2$ = H, X = Cl
**34**: $R^1$ = 2-naphthyl, $R^2$ = OMe, X = Cl
**35**: $R^1$ = 9-anthracenyl, $R^2$ = OMe, X = Br

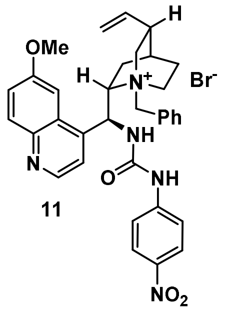


**11**

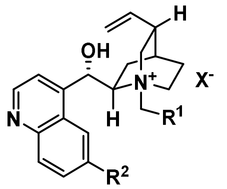


**13**: $R^1$ = Ph, $R^2$ = OMe, X = Cl
**14**: $R^1$ = $C_6F_5$, $R^2$ = OMe, X = Cl
**15**: $R^1$ = 3,5-$(^t\mathrm{Bu})_2C_6H_3$, $R^2$ = OMe, X = Br
**16**: $R^1$ = 9-anthracenyl, $R^2$ = OMe, X = Br
**32**: $R^1$ = Ph, $R^2$ = H, X = Cl

## 1.5 Experimental Procedures

### 1.5.1 Starting Material Synthesis

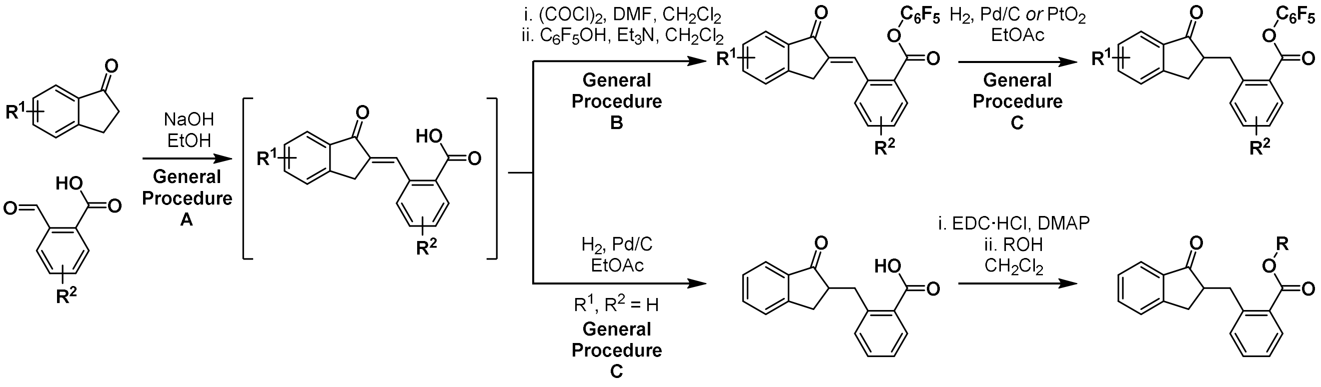

The requisite saturated ester starting materials were prepared according to the general scheme outlined above. Indan-1-ones were commercially available or prepared according to literature procedures.[5] *o*-Carboxybenzaldehydes were prepared according to literature procedures through *o*-lithiation of benzoic acid derivatives, trapping with *N*,*N*-dimethylformamide, followed by hydrolysis of the amide.[6] Indan-1-ones and *o*-carboxybenzaldehydes were then converted to the corresponding saturated ester *via* a three-step sequence of aldol condensation/esterification/hydrogenation according to **General Procedures A, B and C**.

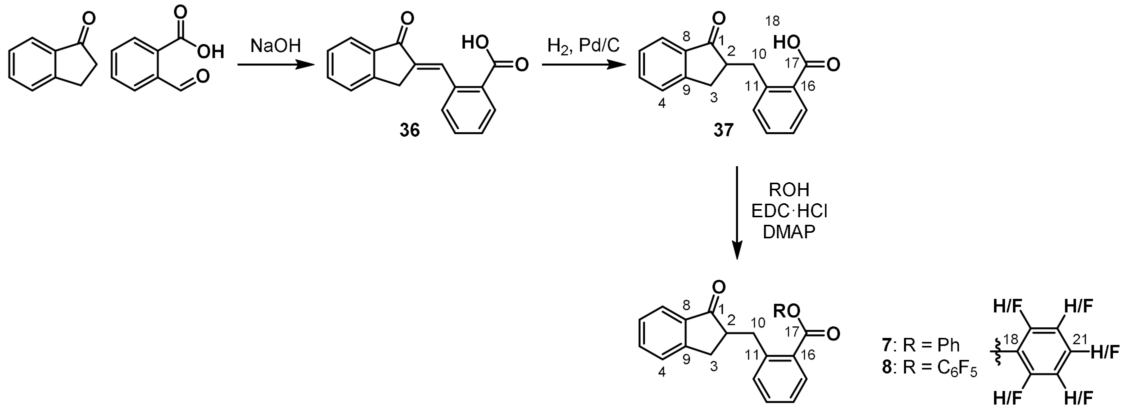

**2-((1-Oxo-1,3-dihydro-2*H*-inden-2-ylidene)methyl)benzoic acid, 36**

This compound was prepared according to **General Procedure A.**

Aldol condensation with 1-indanone (2.20 g, 16.7 mmol), *o*-carboxybenzaldehyde (2.50 g, 16.7 mmol), ethanol (40 mL), aqueous sodium hydroxide (1 M, 30 mL, 30 mmol). Crude unsaturated acid **36** was obtained as a pale yellow solid (4.13 g).

**2-((1-Oxo-2,3-dihydro-1*H*-inden-2-yl)methyl)benzoic acid, 37**

This compound was prepared according to **General Procedure C**.

Hydrogenation with crude unsaturated acid **36** (2.90 g, 11.0 mmol), palladium on carbon (290 mg), ethyl acetate (76 mL). Purification *via* recrystallization from benzene afforded the title compound **37** as a white crystalline solid (2.25 g, 72% for 2 steps). The spectral data matched that previously reported in the literature.[7]

m.p. 144-146 °C (benzene), [lit. 141-143 °C][7];

$^1$H NMR (400 MHz, $CDCl_3$) $\delta_H$ = 11.30 (br s, 1H, $H_{18}$), 8.09 (dd, $J$ = 7.8, 1.3 Hz, 1H, $H_{15}$), 7.78 (d, $J$ = 7.6 Hz, 1H, $H_7$), 7.55 (td, $J$ = 7.4, 1.1 Hz, 1H, $H_{13}$), 7.50 (td, $J$ = 7.5, 1.3 Hz, 1H, $H_5$), 7.43–7.30 (m, 4H, $H_4$, $H_6$, $H_{12}$ & $H_{14}$), 3.87–3.75 (m, 1H, $H_{10A}$), 3.23–3.10 (m, 3H, $H_2$, $H_{3A}$, $H_{10B}$), 2.96–2.85 (m, 1H, $H_{3B}$);

$^{13}$C NMR (101 MHz, $CDCl_3$) $\delta_C$ = 208.0, 172.7, 153.6, 142.6, 136.4, 134.7, 133.0, 131.9, 131.7, 128.7, 128.3, 127.4, 126.6, 126.5, 124.0, 48.7, 35.0, 32.4;

FTIR (neat) ν/cm$^{-1}$ = 3070, 1716, 1687, 1603, 1575, 1489, 1465, 1434, 1404, 1271, 1209, 1150, 1075;

LRMS (ESI$^+$) calculated for $C_{17}H_{14}O_3Na^+$ = 289, mass found = 289.

**Phenyl 2-((1-oxo-2,3-dihydro-1*H*-inden-2-yl)methyl)benzoate, 7**

To a mixture of **37** (600 mg, 2.28 mmol), *N*-(3-dimethylaminopropyl)-*N'*-ethylcarbodiimide hydrochloride (562 mg, 2.93 mmol, 1.30 eq.), and 4-(dimethylamino)pyridine (13 mg, 0.11 mmol, 0.05 eq.) in flame-dried round-bottom flask was added dichloromethane (3 mL) and the mixture was stirred for 30 minutes. A solution of phenol (208 mg, 2.21 mmol, 0.98 eq.) in dichloromethane (3 mL) was added to the mixture and stirred for 14 hours. The reaction mixture was concentrated under reduced pressure and then poured onto brine (15 mL) and extracted with dichloromethane (10 mL × 3). The combined organic extracts were dried over anhydrous sodium sulfate, filtered and concentrated *in vacuo*. The residue was purified by flash chromatography, eluting with 1:9 ethyl acetate/petrol 40-60, to afford title compound **7** as a colourless oil that solidified on standing (554 mg, 73%).

m.p. 63-65 °C (ethyl acetate/petrol 40-60);

$^1$H NMR (400 MHz, $CDCl_3$) $\delta_H$ = 8.19 (dd, *J* = 7.8, 1.3 Hz, 1H, $H_{15}$), 7.76 (d, *J* = 7.6 Hz, 1H, $H_7$), 7.59–7.51 (m, 2H, $H_5$ & $H_{13}$), 7.49–7.32 (m, 6H, $H_4$, $H_{12}$, $H_{14}$, $H_{20}$ & $H_{21}$), 7.30–7.20 (m, 3H, $H_6$ & $H_{19}$), 3.83–3.69 (m, 1H, $H_{10A}$), 3.26–3.13 (m, 3H, $H_2$, $H_{3A}$, $H_{10B}$), 3.00–2.88 (m, 1H, $H_{3B}$);

$^{13}$C NMR (101 MHz, $CDCl_3$) $\delta_C$ = 207.6, 166.0, 153.5, 150.8, 142.5, 136.6, 134.7, 132.7, 131.9, 131.4, 129.6, 129.2, 127.4, 126.7, 126.6, 126.0, 124.0, 115.4, 48.7, 35.0, 32.3;

FTIR (neat) $\nu/cm^{-1}$ = 3068, 2918, 1734, 1708, 1599, 1576, 1486, 1464, 1434, 1328, 1288, 1247, 1191, 1163, 1120, 1071, 1046, 1001;

HRMS (ESI$^+$) calculated for $C_{23}H_{18}O_3Na^+$ = 365.1148, mass found = 365.1144.

**Perfluorophenyl 2-((1-oxo-2,3-dihydro-1*H*-inden-2-yl)methyl)benzoate, 8**

To a mixture of **37** (600 mg, 2.25 mmol), *N*-(3-dimethylaminopropyl)-*N'*-ethylcarbodiimide hydrochloride (648 mg, 3.38 mmol, 1.5 eq.), and 4-(dimethylamino)pyridine (14 mg, 0.12 mmol, 0.05 eq.) in a flame-dried round-bottom flask was added dichloromethane (3.0 mL) and the mixture was stirred for 30 minutes. A solution of pentafluorophenol (415 mg, 2.25 mmol, 1.0 eq.) in dichloromethane (3.0 mL) was added to the mixture and stirred for 14 hours. The reaction mixture was concentrated under reduced pressure and then poured onto brine (15 mL) and extracted with dichloromethane (10 mL × 3). The combined organic extracts were dried over anhydrous sodium sulfate, filtered and concentrated *in vacuo*. The residue was purified by flash chromatography, eluting with 1:19 to 1:9 ethyl acetate/petrol 40-60, to afford title compound **7** as a colourless oil that solidified on standing to a white solid (748 mg, 77%).

m.p. 83-85 °C (ethyl acetate/petrol 40-60);

$^1$H NMR (500 MHz, $CDCl_3$) $\delta_H$ = 8.23 (dd, *J* = 7.9, 1.3 Hz, 1H, $H_{15}$), 7.76 (d, *J* = 7.7 Hz, 1H, $H_7$), 7.61 (td, *J* = 7.6, 1.4 Hz, 1H, $H_{13}$), 7.57 (td, *J* = 7.5, 1.1 Hz, 1H, $H_5$), 7.46 (dd, *J* = 7.8, 1.2 Hz, 1H, $H_{14}$), 7.43 (td, *J* = 7.4, 1.0 Hz, 1H, $H_{12}$), 7.41 (dt, *J* = 7.5, 0.8 Hz, 1H, $H_4$), 7.36 (ddd, *J* = 7.6, 7.2, 0.6 Hz, 1H, $H_6$), 3.76 (dd, *J* = 13.2, 5.0 Hz, 1H, $H_{10A}$), 3.23-3.0.6 (m, 3H, $H_2$, $H_{3A}$, $H_{10B}$), 2.89 (dd, *J* = 16.9, 4.1 Hz, 1H, $H_{3B}$);

$^{13}$C NMR (126 MHz, $CDCl_3$) $\delta_C$ = 206.2, 161.8, 152.2, 142.7, 140.3 (ddq, *J* = 247.9, 12.1, 3.9 Hz), 138.5 (dtt, *J* = 253.4, 13.5, 3.8 Hz), 136.9 (dddd, *J* = 251.6, 14.1, 4.3, 3.8 Hz), 135.4, 133.8, 133.0, 131.2, 131.1, 126.4, 125.9, 125.5, 125.1, 124.2 (tt, *J* = 14.3, 2.4 Hz), 123.0, 47.5, 34.0, 31.3;

$^{19}$F NMR (470 MHz, $CDCl_3$) $\delta_F$ = -152.4, -157.9, -162.3;

FTIR (neat) ν/cm$^{-1}$ = 2928, 1759, 1712, 1602, 1519, 1488, 1466, 1320, 1288, 1273, 1227, 1145, 1115, 1030;

HRMS (ESI$^+$) calculated for $C_{23}H_{13}O_3F_5Na^+$ = 455.0677, mass found = 455.0675.

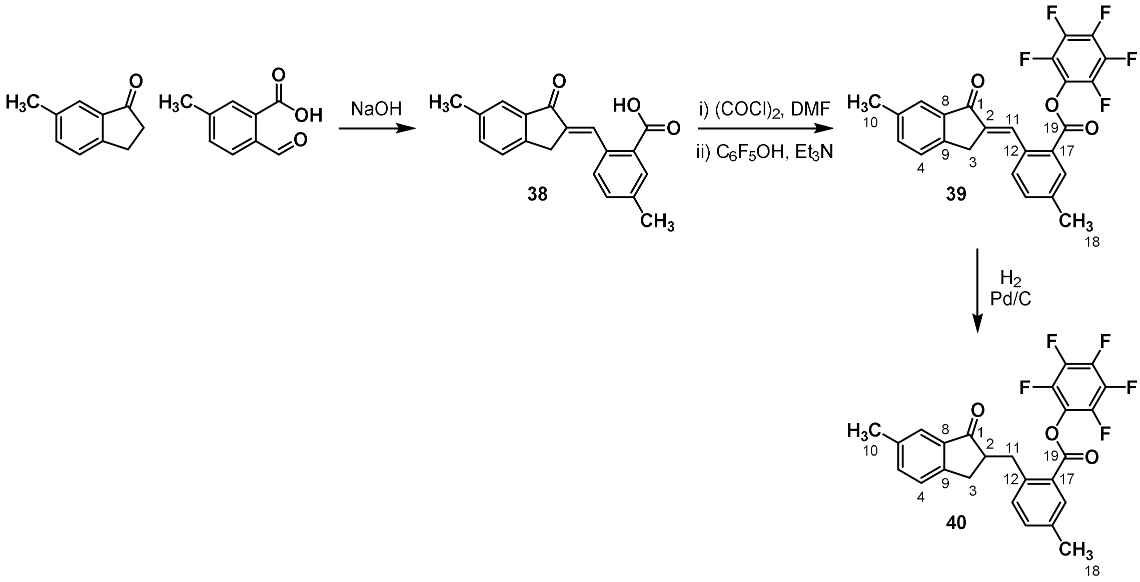


**5-Methyl-2-((6-methyl-1-oxo-1,3-dihydro-2*H*-inden-2-ylidene)methyl)benzoic acid, 38**

This compound was prepared according to **General Procedure A.**

Aldol condensation with 6-methyl-1-indanone (89 mg, 0.61 mmol), 2-formyl-5-methylbenzoic acid (100 mg, 0.609 mmol), ethanol (1.46 mL), aqueous sodium hydroxide (1 M, 1.10 mL, 1.10 mmol). Crude unsaturated acid **38** was obtained as a pale yellow solid (95 mg).

**Perfluorophenyl 5-methyl-2-((6-methyl-1-oxo-1,3-dihydro-2*H*-inden-2-ylidene)methyl) benzoate, 39**

This compound was prepared according to **General Procedure B**.

Esterification with crude unsaturated acid **38** (88 mg, 0.32 mmol), oxalyl chloride (41 μL, 0.48 mmol), *N*,*N*-dimethylformamide (1 drop), dichloromethane (1 mL) then pentafluorophenol (55 mg, 0.32 mmol), triethylamine (78 μL, 0.96 mmol), dichloromethane (1 mL). Purification *via* flash chromatography, eluting with 1:9 to 3:7 ethyl acetate/petrol 40-60, afforded title compound **39** as a yellow solid (113 mg, 42% for 2 steps).

m.p. 168-170 °C (ethyl acetate/petrol 40-60);

$^{1}$H NMR (500 MHz, $CDCl_3$) $\delta_H$ = 8.11 (t, *J* = 2.2 Hz, 1H, $H_{11}$), 8.00 (d, *J* = 1.8 Hz, 1H, $H_{16}$), 7.63 (dt, *J* = 1.6, 0.8 Hz, 1H, $H_7$), 7.55 (d, *J* = 8.0 Hz, 1H, $H_{14}$), 7.46 (dd, *J* = 8.0, 1.8 Hz, 1H, $H_{13}$), 7.34 (dd, *J* = 7.9, 1.7 Hz, 1H, $H_5$), 7.30 (dd, *J* = 7.8, 0.9 Hz, 1H, $H_4$), 3.77 (d, *J* = 1.4 Hz, 2H, $H_3$), 2.43 (s, 3H. $H_{18}$), 2.35 (s, 3H, $H_{10}$);

$^{13}$C NMR (126 MHz, $CDCl_3$) $\delta_C$ = 193.6, 162.4, 147.1, 139.3, 138.3, 137.7, 137.4, 135.9, 135.8, 134.5, 132.5, 131.9, 130.1, 126.9, 125.8, 124.6, 31.0, 21.2, 21.2;

$^{19}$F NMR (470 MHz, $CDCl_3$) $\delta_F$ = -152.1, -157.8, -162.3;

FTIR (neat) ν/cm$^{-1}$ = 2925, 2361, 1756, 1699, 1629, 1520, 1493, 1428, 1319, 1284, 1251, 1178, 1138, 1118, 1138, 1096, 1032;

HRMS (ESI$^+$) calculated for $C_{25}H_{16}O_3F_5^+$ = 459.1014, mass found = 459.1013.

**Perfluorophenyl 5-methyl-2-((6-methyl-1-oxo-2,3-dihydro-1*H*-inden-2-yl)methyl)benzoate, 40**

This compound was prepared according to **General Procedure C**.

Hydrogenation with unsaturated ester **39** (56 mg, 0.11 mmol), palladium on carbon (6 mg), ethyl acetate (1.2 mL). Purification *via* flash chromatography, eluting with 1:19 ethyl acetate/petrol 40-60, afforded title compound **40** as a colourless oil (45 mg, 84%).

$^{1}$H NMR (400 MHz, $CDCl_3$) $\delta_H$ = 8.02 (d, *J* = 1.9 Hz, 1H, $H_{16}$), 7.55 (d, *J* = 1.5 Hz, 1H, $H_7$), 7.41 (dd, *J* = 8.4, 1.9 Hz, 1H, $H_{14}$), 7.38 (dd, *J* = 8.2, 1.6 Hz, 1H, $H_4$), 7.33 (d, *J* = 7.9 Hz, 1H, $H_{13}$), 7.28 (d, *J* = 7.8 Hz, 1H, $H_5$), 4.14–3.37 (m, 1H, $H_{11}$), 3.30–2.99 (m, 3H, $H_2$, $H_3$, $H_{11}$), 2.88–2.69 (m, 1H, $H_3$), 2.43 (s, 3H, $H_{18}$), 2.39 (s, 3H, $H_{10}$);

$^{13}$C NMR (101 MHz, $CDCl_3$) $\delta_C$ = 207.4, 163.0, 150.7, 140.7, 137.4, 136.7, 136.6, 136.0, 134.8, 132.4, 132.12, 126.2, 125.9, 123.9, 48.9, 34.8, 32.0, 21.1, 20.9;

$^{19}$F NMR (377 MHz, $CDCl_3$) $\delta_F$ = -152.4, -158.1, -162.2;

FTIR (neat) ν/cm$^{-1}$ = 3020, 2925, 2358, 1759, 1711, 1520, 1282, 1255, 1215, 1176, 1032;

HRMS (ESI$^+$) calculated for $C_{25}H_{18}O_3F_5^+$ = 461.1171, mass found = 461.1170.

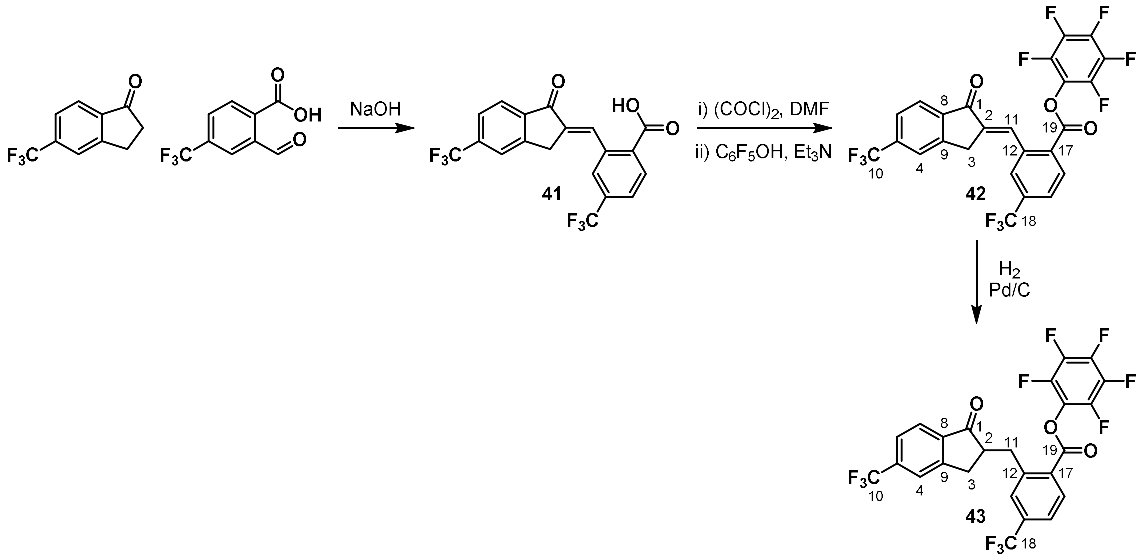


**2-((1-Oxo-5-(trifluoromethyl)-1,3-dihydro-2*H*-inden-2-ylidene)methyl)-4-(trifluoromethyl)benzoic acid, 41**

This compound was prepared according to **General Procedure A.**

Aldol condensation with 6-(trifluoromethyl)-1-indanone (144 mg, 0.719 mmol), 2-formyl-5-(trifluoromethyl)benzoic acid (157 mg, 0.719 mmol), ethanol (1.7 mL), aqueous sodium hydroxide (1 M, 1.29 mL, 1.29 mmol). Crude unsaturated acid **41** was obtained as a pale yellow solid (271 mg).

**Perfluorophenyl 2-((1-oxo-5-(trifluoromethyl)-1,3-dihydro-2*H*-inden-2-ylidene)methyl)-4-(trifluoromethyl)benzoate, 42**

This compound was prepared according to **General Procedure B**.

Esterification with crude unsaturated acid **41** (271 mg, 0.677 mmol), oxalyl chloride (86.3 µL, 1.02 mmol), *N*,*N*-dimethylformamide (1 drop), dichloromethane (2.0 mL) then pentafluorophenol (125 mg, 0.677 mmol), triethylamine (283 µL, 2.03 mmol), dichloromethane (2.0 mL). Purification *via* flash chromatography, eluting with 3:97 to 5:95 ethyl acetate/petrol 40-60, afforded title compound **42** as a yellow solid (160 mg, 39% for 2 steps).

m.p. 115-117 °C (ethyl acetate/petrol 40-60);

$^1$H NMR (400 MHz, $CDCl_3$) $\delta_H$ = 8.42 (d, *J* = 8.2 Hz, 1H, $H_{15}$), 8.24 (t, *J* = 2.2 Hz, 1H, $H_{11}$), 8.00 (d, *J* = 8.0 Hz, 1H, $H_7$), 7.96 (d, *J* = 1.7 Hz, 1H, $H_{13}$), 7.85 (dd, *J* = 8.3, 1.7 Hz, 1H, $H_{16}$), 7.81 (s, 1H, $H_4$), 7.70 (d, *J* = 8.2 Hz, 1H, $H_6$), 3.97 (d, *J* = 2.2 Hz, 2H, $H_3$);

$^{13}C$ NMR (126 MHz, $CDCl_3$) $\delta_C$ = 191.9, 161.3, 149.5, 140.4, 139.1, 137.8, 136.2 (q, *J* = 32.4 Hz), 135.6 (q, *J* = 33.3 Hz), 132.7, 131.9, 130.1, 126.7 (q, *J* = 4.0 Hz), 125.9 (q, *J* = 3.4 Hz), 125.2, 125.0 (d, *J* = 3.8 Hz), 123.6 (q, *J* = 4.0 Hz), 123.5 (d, *J* = 272.8 Hz), 123.0 (d, *J* = 273.4 Hz), 30.9;

$^{19}F$ NMR (377 MHz, $CDCl_3$) $\delta_F$ = -63.0, -63.4, -152.2, -156.8 -161.6;

FTIR (neat) ν/$cm^{-1}$ = 2361, 2341, 1767, 1713, 1522, 1434, 1329, 1238, 1174, 1134, 1082, 1059, 1038;

HRMS ($ESI^+$) calculated for $C_{25}H_9O_3F_{11}Na^+$ = 589.0268, mass found = 589.0264.

**Perfluorophenyl 2-((1-oxo-5-(trifluoromethyl)-2,3-dihydro-1*H*-inden-2-yl)methyl)-4-(trifluoromethyl)benzoate, 43**

This compound was prepared according to **General Procedure C**.

Hydrogenation with unsaturated ester **42** (160 mg, 0.11 mmol), palladium on carbon (16 mg), ethyl acetate (2.9 mL). Purification *via* recrystallization from ethyl acetate/petrol 60-80 afforded title compound **43** as a yellow crystalline solid (123 mg, 77%).

m.p. 98-100 °C (ethyl acetate/petrol 60-80);

$^{1}H$ NMR (500 MHz, $CDCl_3$) $\delta_H$ = 8.35 (d, *J* = 8.1 Hz, 1H, $H_{16}$), 7.87 (d, *J* = 7.9 Hz, 1H, $H_7$), 7.73 (s, 1H, $H_{13}$), 7.72 (d, *J* = 7.7 Hz, 1H, $H_{15}$), 7.71 (s, 1H, $H_4$), 7.64 (d, *J* = 7.9 Hz, 1H, $H_6$) 3.78 (dd, *J* = 13.6, 5.7 Hz, 1H, $H_{11A}$), 3.32 (dd, *J* = 17.2, 7.9 Hz, 1H, $H_{3A}$), 3.23 (dd, *J* = 13.6, 9.0 Hz, 1H, $H_{11B}$), 3.16 (dddd, *J* = 9.0, 8.0, 5.7, 4.5 Hz, 1H, $H_2$), 2.96 (dd, *J* = 17.2, 4.5 Hz, 1H, $H_{3B}$);

$^{13}C$ NMR (126 MHz, $CDCl_3$) $\delta_C$ = 205.5, 162.0, 152.9, 144.1, 138.9, 136.3 (q, *J* = 32.1 Hz), 135.4 (q, *J* = 33.0 Hz), 132.5, 129.5, 128.9 (q, *J* = 3.7 Hz), 124.8 (q, *J* = 3.8 Hz), 124.7, 124.1 (q, *J* = 3.7 Hz), 123.8 (q, *J* = 3.9 Hz), 123.6 (d, *J* = 273.3 Hz), 123.2 (d, *J* = 272.6 Hz), 48.6, 35.0, 32.4;

$^{19}F$ NMR (470 MHz, $CDCl_3$) $\delta_F$ = -62.9, -63.4, -152.4, -156.9, -161.7;

FTIR (neat) ν/$cm^{-1}$ = 2981, 1766, 1722, 1521, 1433, 1328, 1231, 1171, 1133, 1084, 1059, 1035;

HRMS ($ACI^+$) calculated for $C_{25}H_{12}O_3F_{11}^+$ = 569.0605, mass found = 569.0607.

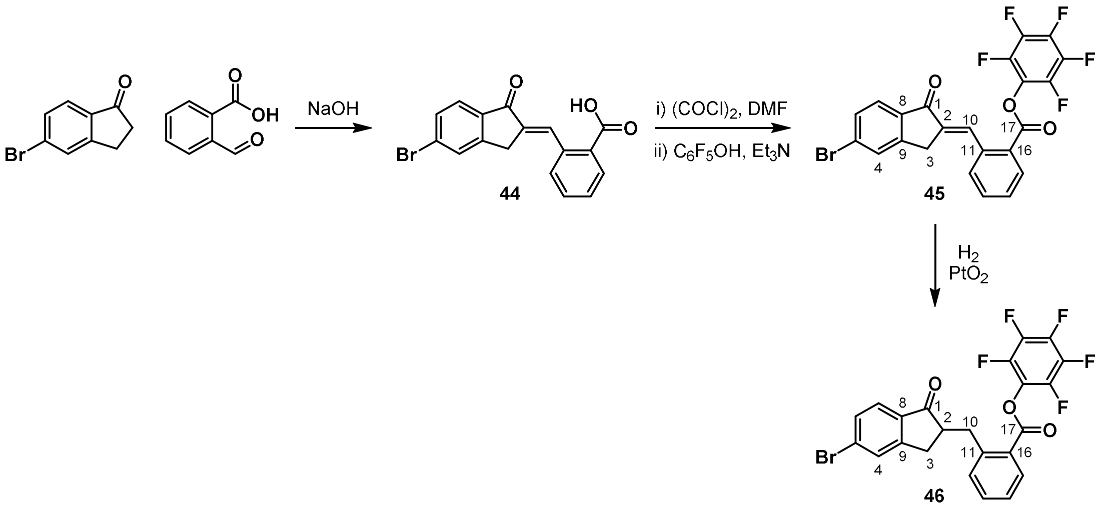


**2-((5-Bromo-1-oxo-1,3-dihydro-2*H*-inden-2-ylidene)methyl)benzoic acid, 44**

This compound was prepared according to **General Procedure A.**

Aldol condensation with 5-bromo-1-indanone (445 mg, 2.11 mmol), *o*-carboxybenzaldehyde (318 mg, 2.11 mmol), ethanol (5.09 mL), aqueous sodium hydroxide (1 M, 3.81 mL, 3.81 mmol). Crude unsaturated acid **43** was obtained as a pale yellow solid (504 mg).

**Perfluorophenyl 2-((5-bromo-1-oxo-1,3-dihydro-2*H*-inden-2-ylidene)methyl)benzoate, 45**

This compound was prepared according to **General Procedure B**.

Esterification with crude unsaturated acid **44** (504 mg, 1.46 mmol), oxalyl chloride (187 μL, 1.46 mmol), *N*,*N*-dimethylformamide (1 drop), dichloromethane (6 mL) then pentafluorophenol (271 mg, 1.46 mmol), triethylamine (620 μL, 4.40 mmol), dichloromethane (6 mL). Purification *via* flash chromatography, eluting with 1:9 to 1:3 ethyl acetate/petrol 40-60, afforded title compound **45** as a brown solid (717 mg, 67% for 2 steps).

m.p. 188-190 °C (ethyl acetate/petrol 40-60);

$^{1}$H NMR (500 MHz, $CDCl_3$) $\delta_H$ = 8.30 (dd, *J* = 7.9, 1.4 Hz, 1H, $H_{15}$), 8.24 (t, *J* = 2.2 Hz, 1H, $H_{10}$), 7.80–7.72 (m, 2H, $H_7$, $H_{13}$), 7.68 (d, *J* = 7.6 Hz, 1H, $H_{12}$), 7.65 (d, *J* = 1.0 Hz, 1H, $H_4$), 7.60–7.54 (m, 2H, $H_6$, $H_{14}$), 3.87 (d, *J* = 2.3 Hz, 2H, $H_3$);

$^{13}$C NMR (126 MHz, $CDCl_3$) $\delta_C$ = 192.2, 162.1, 151.2, 138.5, 137.0, 136.7, 134.0, 133.1, 132.2, 131.4, 130.0, 129.9, 129.5, 129.1, 126.8, 125.9, 30.9;

$^{19}$F NMR (470 MHz, $CDCl_3$) $\delta_F$ = -152.2, -157.5, -162.1;

FTIR (neat) ν/cm$^{-1}$ = 2362, 1756, 1697, 1619, 1598, 1575, 1518, 1417, 1319, 1263, 1234, 1298, 1093, 1034;

HRMS (ESI$^+$) calculated for $C_{23}H_{11}O_3BrF_5^+$ = 508.9806, mass found = 508.9808.

**Perfluorophenyl 2-((5-bromo-1-oxo-2,3-dihydro-1*H*-inden-2-yl)methyl)benzoate, 46**

This compound was prepared according to **General Procedure C**.

Hydrogenation with unsaturated ester **45** (717 mg, 1.41 mmol), platinum(IV) oxide (72 mg), ethyl acetate (14.3 mL). Purification *via* flash chromatography, eluting with 1:9 ethyl acetate/petrol 40-60 afforded title compound **46** as a white solid (491 mg, 68%).

m.p. 88-90 °C (ethyl acetate/petrol 40-60);

$^1$H NMR (500 MHz, CDCl$_3$) $\delta_H$ = 8.24 (dd, $J$ = 8.2, 1.5 Hz, 1H, $H_{15}$), 7.70–7.59 (m, 2H, $H_7$, $H_{13}$), 7.59–7.55 (m, 1H, $H_4$), 7.50 (ddt, $J$ = 8.0, 1.5, 0.7 Hz, 1H, $H_6$), 7.47–7.40 (m, 2H, $H_{12}$, $H_{14}$), 3.74 (dd, $J$ = 13.2, 5.0 Hz, 1H, $H_{10A}$), 3.57–3.04 (m, 3H, $H_2$, $H_{3A}$, $H_{10B}$), 2.88 (dd, $J$ = 17.1, 4.0 Hz, 1H, $H_{3B}$);

$^{13}$C NMR (126 MHz, CDCl$_3$) $\delta_C$ = 205.9, 162.8, 154.9, 143.4, 135.2, 134.1, 132.2, 132.1, 131.1, 130.2, 129.8, 127.1, 126.1, 125.3, 48.5, 35.0, 32.0;

$^{19}$F NMR (470 MHz, CDCl$_3$) $\delta_F$ = -152.5, -157.8, -162.2;

FTIR (neat) ν/cm$^{-1}$ = 2980, 1760, 1715, 1597, 1520, 1228, 1031;

HRMS (ESI$^+$) calculated for $C_{23}H_{13}O_3BrF_5^+$ = 532.9782, mass found = 532.9783.

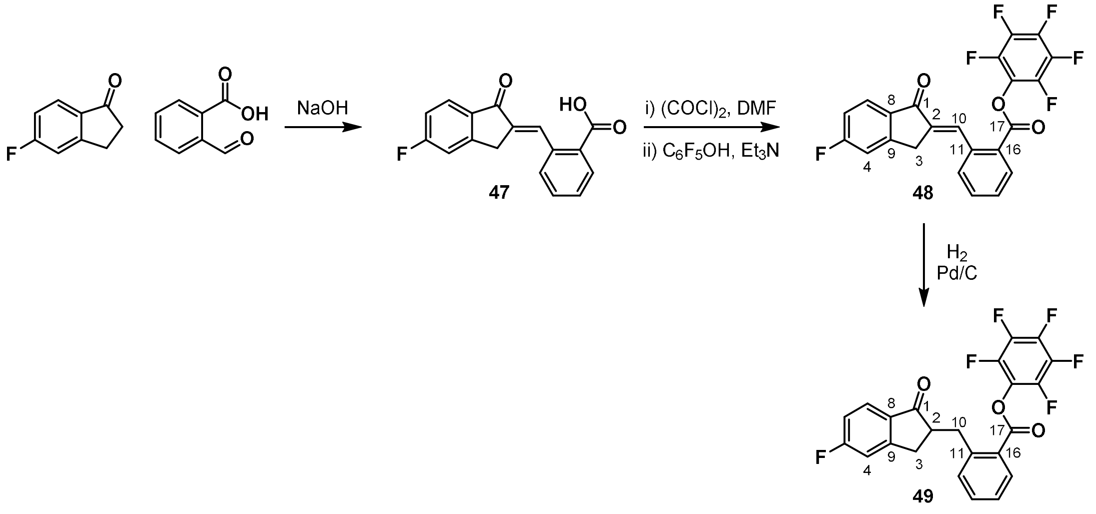


**2-((5-Fluoro-1-oxo-1,3-dihydro-2*H*-inden-2-ylidene)methyl)benzoic acid, 47**

This compound was prepared according to **General Procedure A.**

Aldol condensation with 5-fluoro-1-indanone (100 mg, 0.666 mmol), *o*-carboxybenzaldehyde (100 mg, 0.666 mmol), ethanol (1.60 mL), aqueous sodium hydroxide (1 M, 1.20 mL, 1.20 mmol). Crude unsaturated acid **47** was obtained as a pink solid (132 mg).

**Perfluorophenyl 2-((5-fluoro-1-oxo-1,3-dihydro-2*H*-inden-2-ylidene)methyl)benzoate, 48**

This compound was prepared according to **General Procedure B**.

Esterification with crude unsaturated acid **47** (132 mg, 0.468 mmol), oxalyl chloride (59.4 µL, 0.702 mmol), *N*,*N*-dimethylformamide (1 drop), dichloromethane (1.5 mL) then pentafluorophenol (86 mg, 0.47 mmol), triethylamine (192 µL, 1.41 mmol), dichloromethane (1.5 mL). Purification *via* flash chromatography, eluting with 1:9 ethyl acetate/petrol 40-60 afforded title compound **48** as a white solid (157 mg, 53% for 2 steps).

m.p. 148-150 °C (ethyl acetate/petrol 40-60);

$^{1}$H NMR (400 MHz, $CDCl_3$) $\delta_H$ = 8.29 (dd, *J* = 7.9, 1.4 Hz, 1H, $H_{15}$), 8.20 (t, *J* = 2.3 Hz, 1H, $H_{10}$), 7.91 (dd, *J* = 8.4, 5.3 Hz, 1H, $H_7$), 7.75 (td, *J* = 7.6, 1.4 Hz, 1H, $H_{13}$), 7.69 (dd, *J* = 7.8, 1.4 Hz, 1H, $H_{12}$), 7.58 (td, *J* = 7.6, 1.5 Hz, 1H, $H_{14}$), 7.20–7.06 (m, 2H, $H_4$, $H_6$), 3.87 (d, *J* = 2.2 Hz, 2H, $H_3$);

$^{13}$C NMR (101 MHz, $CDCl_3$) $\delta_C$ = 191.6, 167.0 (d, *J* = 255.6 Hz), 162.2, 152.5 (d, *J* = 10.3 Hz), 138.6, 137.0, 134.5, 133.9, 132.5, 132.1, 130.0, 129.0, 127.0 (d, *J* = 10.4 Hz), 126.8, 116.0 (d, *J* = 23.8 Hz), 112.9 (d, *J* = 22.6 Hz), 31.1;

$^{19}$F NMR (377 MHz, $CDCl_3$) $\delta_F$ = -102.1, -152.2, -157.6, -162.1;

FTIR (neat) $\nu$/cm$^{-1}$ = 1758, 1702, 1618, 1593, 1518, 1481, 1332, 1264, 1233, 1145, 1127, 1086, 1030;

HRMS (ESI$^+$) calculated for $C_{23}H_{11}O_3F_6^+$ = 449.0607, mass found = 449.0608.

**Perfluorophenyl 2-((5-fluoro-1-oxo-2,3-dihydro-1*H*-inden-2-yl)methyl)benzoate, 49**

This compound was prepared according to **General Procedure C**.

Hydrogenation with unsaturated ester **48** (100 mg, 0.223 mmol), palladium on carbon (10 mg), ethyl acetate (2.2 mL). Purification *via* flash chromatography, eluting with 1:19 to 1:9 ethyl acetate/petrol 40-60 afforded title compound **49** as a white solid (86 mg, 86%).

m.p. 74-76 °C (ethyl acetate/petrol 40-60);

$^1$H NMR (500 MHz, $CDCl_3$) $\delta_H$ = 8.24 (dd, $J$ = 7.9, 1.4 Hz, 1H, $H_{15}$), 7.76 (dd, $J$ = 9.1, 5.3 Hz, 1H, $H_7$), 7.61 (td, $J$ = 7.6, 1.4 Hz, 1H, $H_{13}$), 7.52–7.38 (m, 2H, $H_{12}$, $H_{14}$), 7.15–6.95 (m, 2H, $H_4$, $H_6$), 3.74 (dd, $J$ = 12.7, 4.3 Hz, 1H, $H_{10A}$), 3.54–3.02 (m, 3H, $H_2$, $H_{3A}$, $H_{10B}$), 2.88 (dd, $J$ = 17.2, 3.5 Hz, 1H, $H_{3B}$);

$^{13}$C NMR (126 MHz, $CDCl_3$) $\delta_C$ = 205.2, 167.3 (d, $J$ = 256.1 Hz), 162.8, 156.1 (d, $J$ = 10.0 Hz), 143.5, 134.1, 132.8 (d, $J$ = 1.8 Hz), 132.2, 132.1, 127.1, 126.3 (d, $J$ = 10.6 Hz), 126.1, 115.8 (d, $J$ = 23.8 Hz), 113.1 (d, $J$ = 22.2 Hz), 48.7, 35.0, 32.2 (d, $J$ = 2.1 Hz);

$^{19}$F NMR (470 MHz, $CDCl_3$) $\delta_F$ = -102.8, -152.5, -157.8, -162.2;

FTIR (neat) ν/cm$^{-1}$ = 3066, 1760, 1714, 1617, 1594, 1520, 1483, 1248, 1229, 1144, 1085, 1031;

HRMS (ESI$^+$) calculated for $C_{23}H_{13}O_3F_6^+$ = 451.0763, mass found = 451.0762.

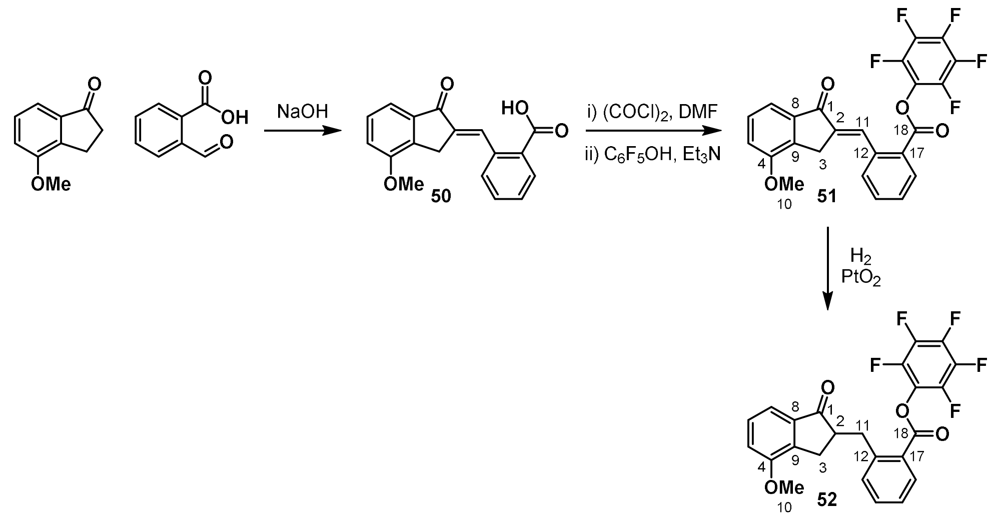


**2-((4-Methoxy-1-oxo-1,3-dihydro-2*H*-inden-2-ylidene)methyl)benzoic acid, 50**

This compound was prepared according to **General Procedure A.**

Aldol condensation with 4-methoxy-1-indanone (150 mg, 0.925 mmol), *o*-carboxybenzaldehyde (139 mg, 0.925 mmol), ethanol (2.2 mL), aqueous sodium hydroxide (1 M, 1.67 mL, 1.67 mmol). Crude unsaturated acid **50** was obtained as a white solid (226 mg).

**Perfluorophenyl 2-((4-methoxy-1-oxo-1,3-dihydro-2*H*-inden-2-ylidene)methyl)benzoate, 51**

This compound was prepared according to **General Procedure B**.

Esterification with crude unsaturated acid **50** (226 mg, 0.768 mmol), oxalyl chloride (98.0 µL, 1.15 mmol), *N*,*N*-dimethylformamide (1 drop), dichloromethane (2.5 mL) then

pentafluorophenol (142 mg, 0.768 mmol), triethylamine (321 μL, 2.30 mmol), dichloromethane (2.5 mL). Purification *via* flash chromatography, eluting with 1:4 ethyl acetate/petrol 40-60 afforded title compound **51** as a white solid (300 mg, 71% for 2 steps).

m.p. 110-112 °C (ethyl acetate/petrol 40-60);

$^1$H NMR (400 MHz, $CDCl_3$) $\delta_H$ = 8.28 (dt, $J$ = 7.7, 1.0 Hz, 1H, $H_{16}$), 8.23 (t, $J$ = 2.1 Hz, 1H, $H_{11}$), 7.93–7.70 (m, 2H, $H_{13}$, $H_{14}$), 7.68–7.53 (m, 1H, $H_{15}$), 7.50 (dd, $J$ = 7.6, 0.8 Hz, 1H, $H_7$), 7.39 (t, $J$ = 7.8 Hz, 1H, $H_6$), 7.07 (dd, $J$ = 8.0, 0.8 Hz, 1H, $H_5$), 3.91 (s, 3H, $H_{10}$), 3.78 (d, $J$ = 2.1 Hz, 2H, $H_{11}$);

$^{13}$C NMR (101 MHz, $CDCl_3$) $\delta_C$ = 193.8, 162.2, 156.6, 139.4, 138.7, 138.6, 137.3, 134.0, 132.6, 132.0, 130.2, 129.2, 128.9, 126.8, 116.3, 115.2, 55.5, 28.1;

$^{19}$F NMR (377 MHz, $CDCl_3$) $\delta_F$ = -152.2, -157.7, -162.2;

FTIR (neat) ν/cm$^{-1}$ = 2981, 2359, 1760, 1704, 1635, 1604, 1520, 1489, 1279, 1234, 1146, 1077, 1032;

HRMS (ESI$^+$) calculated for $C_{24}H_{13}O_4F_5Na^+$ = 483.0626, mass found = 483.0625.

**Perfluorophenyl 2-((4-methoxy-1-oxo-2,3-dihydro-1*H*-inden-2-yl)methyl)benzoate, 52**

This compound was prepared according to **General Procedure C**.

Hydrogenation with unsaturated ester **51** (284 mg, 0.617 mmol), platinum(IV) oxide (28 mg), ethyl acetate (6.2 mL). Purification *via* flash chromatography, eluting with 1:9 ethyl acetate/petrol 40-60 afforded title compound **52** as a colourless oil (73 mg, 26%) and recovered starting material **51** (163 mg, 53%).

$^1$H NMR (500 MHz, $CDCl_3$) $\delta_H$ = 8.23 (dd, $J$ = 7.9, 1.5 Hz, 1H, $H_{16}$), 7.61 (td, $J$ = 7.6, 1.5 Hz, 1H, $H_{14}$), 7.46 (dd, $J$ = 7.7, 1.3 Hz, 1H, $H_{13}$), 7.42 (td, $J$ = 7.6, 1.3 Hz, 1H, $H_{15}$), 7.39–7.31 (m, 2H, $H_6$, $H_7$), 7.02 (dd, $J$ = 7.1, 1.9 Hz, 1H, $H_5$), 3.87 (s, 3H, $H_{10}$), 3.80–3.56 (m, 1H, $H_{11A}$), 3.53–2.95 (m, 3H, $H_2$, $H_{3A}$, $H_{11B}$), 2.88–2.53 (m, 1H, $H_{3B}$);

$^{13}$C NMR (126 MHz, $CDCl_3$) $\delta_C$ = 207.3, 162.8, 156.9, 143.8, 142.1, 137.9, 134.0, 132.1, 132.1, 129.0, 126.9, 126.1, 115.6, 114.9, 55.4, 48.2, 35.3, 29.0;

$^{19}$F NMR (470 MHz, $CDCl_3$) $\delta_F$ = -152.4, -158.1, -162.4;

FTIR (neat) ν/cm$^{-1}$ = 3659, 2981, 1759, 1715, 1601, 1520, 1488, 1382, 1264, 1232, 1146, 1071, 1030;

HRMS (ESI$^+$) calculated for $C_{24}H_{16}O_4F_5^+$ = 463.0963, mass found = 463.0962.

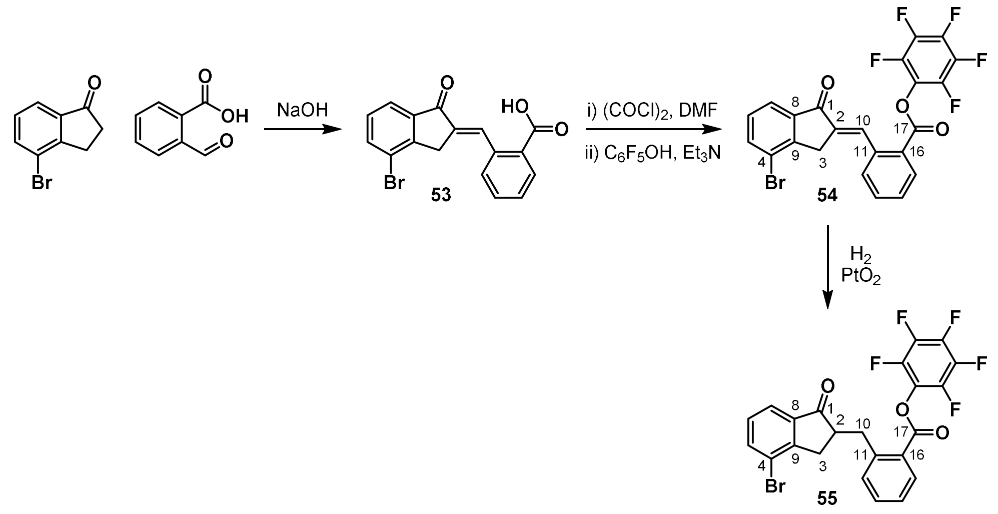

**2-((4-Bromo-1-oxo-1,3-dihydro-2*H*-inden-2-ylidene)methyl)benzoic acid, 53**

This compound was prepared according to **General Procedure A.**

Aldol condensation with 4-bromo-1-indanone (100 mg, 0.473 mmol), *o*-carboxybenzaldehyde (71 mg, 0.47 mmol), ethanol (1.15 mL), aqueous sodium hydroxide (1 M, 0.85 mL, 0.85 mmol). Crude unsaturated acid **53** was obtained as a white solid (154 mg).

**Perfluorophenyl 2-((4-bromo-1-oxo-1,3-dihydro-2*H*-inden-2-ylidene)methyl)benzoate, 54**

This compound was prepared according to **General Procedure B**.

Esterification with crude unsaturated acid **53** (154 mg, 0.449 mmol), oxalyl chloride (56.9 µL, 0.673 mmol), *N*,*N*-dimethylformamide (1 drop), dichloromethane (1.3 mL) then pentafluorophenol (83 mg, 0.45 mmol), triethylamine (188 µL, 1.35 mmol), dichloromethane (1.3 mL). Purification *via* flash chromatography, eluting with 1:19 ethyl acetate/petrol 40-60 followed by recrystallization with ethyl acetate/petrol 60-80 afforded title compound **54** as a yellow solid (132 mg, 41% for 2 steps).

m.p. 108-110 °C (ethyl acetate/petrol 60-80);

$^1$H NMR (500 MHz, $CDCl_3$) $\delta_H$ = 8.31 (dd, *J* = 7.9, 1.4 Hz, 1H, $H_{15}$), 8.27 (t, *J* = 2.2 Hz, 1H, $H_{10}$), 7.86 (dd, *J* = 7.6, 0.9 Hz, 1H, $H_7$), 7.83–7.71 (m, 3H, $H_5$, $H_{12}$, $H_{13}$), 7.60 (td, *J* = 7.7, 1.3 Hz, 1H, $H_{14}$), 7.34 (tt, *J* = 7.6, 0.8 Hz, 1H, $H_6$), 3.81 (d, *J* = 2.1 Hz, 2H, $H_3$);

$^{13}$C NMR (126 MHz, $CDCl_3$) $\delta_C$ = 191.6, 161.1, 148.6, 139.1, 137.4, 136.4, 135.3, 133.1, 132.6, 131.1, 129.0, 128.5, 128.2, 125.8, 122.4, 120.7, 31.3;

$^{19}$F NMR (470 MHz, $CDCl_3$) $\delta_F$ = -152.2, -157.6, -162.1;

FTIR (neat) ν/cm$^{-1}$ = 1759, 1707, 1639, 1598, 1520, 1459, 1325, 1266, 1235, 1124, 1098, 1032;

HRMS (ACI$^+$) calculated for $C_{23}H_{10}O_3BrF_5^+$ = 508.9806, mass found = 508.9807.

**Perfluorophenyl 2-((4-bromo-1-oxo-2,3-dihydro-1*H*-inden-2-yl)methyl)benzoate, 55**

This compound was prepared according to **General Procedure C**.

Hydrogenation with unsaturated ester **54** (124 mg, 0.244 mmol), platinum(IV) oxide (12 mg), ethyl acetate (2.4 mL). Purification *via* flash chromatography, eluting with 1:19 ethyl acetate/petrol 40-60 afforded title compound **55** as a colourless oil (79 mg, 68%).

$^1$H NMR (500 MHz, $CDCl_3$) $\delta_H$ = 8.27 (dd, *J* = 7.9, 1.4 Hz, 1H, $H_{15}$), 7.75 (dd, *J* = 7.7, 1.0 Hz, 1H, $H_7$), 7.71 (dd, *J* = 7.6, 0.9 Hz, 1H, $H_5$), 7.64 (td, *J* = 7.6, 1.5 Hz, 1H, $H_{13}$), 7.49–7.43 (m, 2H, $H_{12}$, $H_{14}$), 7.28 (t, *J* = 7.6 Hz, 1H, $H_6$), 3.91–3.71 (m, 1H, $H_{10A}$), 3.30–3.07 (m, 3H, $H_2$, $H_{3A}$, $H_{10B}$), 2.91–2.76 (m, 1H, $H_{3B}$);

$^{13}$C NMR (126 MHz, $CDCl_3$) $\delta_C$ = 206.3, 162.8, 152.9, 143.4, 138.4, 137.6, 134.2, 132.3, 132.2, 129.3, 127.2, 126.0, 122.9, 122.2, 48.4, 35.2, 33.5;

$^{19}$F NMR (470 MHz, $CDCl_3$) $\delta_F$ = -152.4, -157.9, -162.2;

FTIR (neat) ν/cm$^{-1}$ = 2981, 1759, 1719, 1599, 1520, 1457, 1326, 121, 1229, 1145, 1120, 1030;

HRMS (ACI$^+$) calculated for $C_{23}H_{12}O_3BrF_5^+$ = 510.9963, mass found = 510.9967.

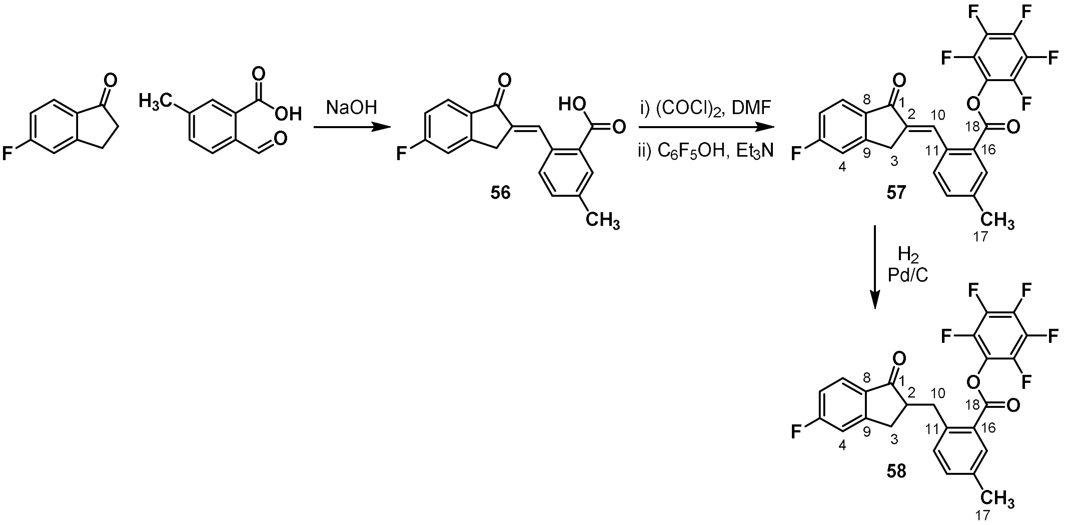


**2-((5-Fluoro-1-oxo-1,3-dihydro-2*H*-inden-2-ylidene)methyl)-5-methylbenzoic acid, 56**

This compound was prepared according to **General Procedure A.**

Aldol condensation with 5-fluoro-1-indanone (100 mg, 0.666 mmol), 2-formyl-5-methylbenzoic acid (110 mg, 0.666 mmol), ethanol (1.60 mL), aqueous sodium hydroxide (1 M, 1.20 mL, 1.20 mmol). Crude unsaturated acid **56** was obtained as a white solid (120 mg).

**Perfluorophenyl 2-((5-fluoro-1-oxo-1,3-dihydro-2*H*-inden-2-ylidene)methyl)-5-methyl benzoate, 57**

This compound was prepared according to **General Procedure B**.

Esterification with crude unsaturated acid **56** (120 mg, 0.405 mmol), oxalyl chloride (51.5 µL, 0.608 mmol), *N*,*N*-dimethylformamide (1 drop), dichloromethane (1.5 mL) then pentafluorophenol (75 mg, 0.40 mmol), triethylamine (170 µL, 1.22 mmol), dichloromethane (1.5 mL). Purification *via* flash chromatography, eluting with 1:9 ethyl acetate/petrol 40-60 afforded title compound **57** as a yellow solid (130 mg, 44% for 2 steps).

m.p. 136-138 °C (ethyl acetate/petrol 40-60);

$^1$H NMR (500 MHz, $CDCl_3$) $\delta_H$ = 8.19 (d, $J$ = 2.2 Hz, 1H, $H_{10}$), 8.08 (d, $J$ = 1.7 Hz, 1H, $H_{15}$), 7.90 (dd, $J$ = 8.4, 5.3 Hz, 1H, $H_7$), 7.60 (d, $J$ = 7.9 Hz, 1H, $H_{12}$), 7.54 (dd, $J$ = 8.0, 1.8 Hz, 1H, $H_{13}$), 7.14 (dd, $J$ = 8.4, 2.1 Hz, 1H, $H_4$), 7.11 (td, $J$ = 8.7, 2.3 Hz, 1H, $H_6$), 3.88 (br s, 2H, $H_3$), 2.50 (s, 3H, $H_{17}$);

$^{13}$C NMR (126 MHz, $CDCl_3$) $\delta_C$ = 191.7, 167.0 (d, $J$ = 256.1 Hz), 162.3, 152.5 (d, $J$ = 10.1 Hz), 139.5, 136.5, 135.5, 134.6, 134.6 (d, $J$ = 1.9 Hz), 132.6, 132.4, 130.0, 126.9 (d, $J$ = 10.2 Hz), 126.9, 116.0 (d, $J$ = 23.6 Hz), 112.9 (d, $J$ = 22.6 Hz), 31.3 (d, $J$ = 2.2 Hz), 21.2;

$^{19}$F NMR (470 MHz, $CDCl_3$) $\delta_F$ = -102.3, -152.2, -157.7, -162.2.;

FTIR (neat) ν/cm$^{-1}$ = 3658, 2981, 2924, 2358, 1759, 1699, 1618, 1520, 1462, 1382, 1251, 1150, 1085, 1034;

HRMS (ESI$^+$) calculated for $C_{24}H_{13}O_3F_6^+$ = 463.0763, mass found = 463.0761.

**Perfluorophenyl 2-((5-fluoro-1-oxo-2,3-dihydro-1*H*-inden-2-yl)methyl)-5-methyl benzoate, 58**

This compound was prepared according to **General Procedure C**.

Hydrogenation with unsaturated ester **57** (120 mg, 0.260 mmol), palladium on carbon (12 mg), ethyl acetate (2.6 mL). Purification *via* flash chromatography, eluting with 3:37 ethyl acetate/petrol 40-60 afforded title compound **58** as a white solid (112 mg, 93%).

m.p. 62-66 °C (ethyl acetate/petrol 40-60);

$^1$H NMR (500 MHz, CDCl$_3$) $\delta_H$ = 8.03 (d, *J* = 1.3 Hz, 1H, H$_{15}$), 7.75 (dd, *J* = 9.2, 5.3 Hz, 1H, H$_7$), 7.41 (dd, *J* = 7.8, 1.9 Hz, 1H, H$_{13}$), 7.32 (d, *J* = 7.9 Hz, 1H, H$_{12}$), 7.13–6.99 (m, 2H, H$_4$, H$_6$), 3.95–3.59 (m, 1H, H$_{10A}$), 3.21–3.06 (m, 3H, H$_2$, H$_{3A}$, H$_{10B}$), 2.86 (dd, *J* = 17.1, 3.1 Hz, 1H, H$_{3B}$), 2.43 (s, 3H, H$_{17}$);

$^{13}$C NMR (126 MHz, CDCl$_3$) $\delta_C$ = 205.4, 167.2 (d, *J* = 256.1 Hz), 163.0, 156.2 (d, *J* = 10.0 Hz), 140.4, 136.9, 134.9, 132.8 (d, *J* = 1.8 Hz), 132.5, 132.1, 126.3 (d, *J* = 10.5 Hz), 125.9, 115.8 (d, *J* = 23.8 Hz), 113.1 (d, *J* = 22.0 Hz), 48.8, 34.6, 32.2 (d, *J* = 2.2 Hz), 20.9;

$^{19}$F NMR (470 MHz, CDCl$_3$) $\delta_F$ = -102.9, -152.5, -157.9, -162.2;

FTIR (neat) ν/cm$^{-1}$ = 2930, 1759, 1715, 1617, 1594, 1520, 1482, 1434, 1333, 1248, 1208, 1176, 1144, 1084, 1032;

HRMS (ESI$^+$) calculated for $C_{24}H_{14}O_3F_6Na^+$ = 487.0739, mass found = 487.0740.

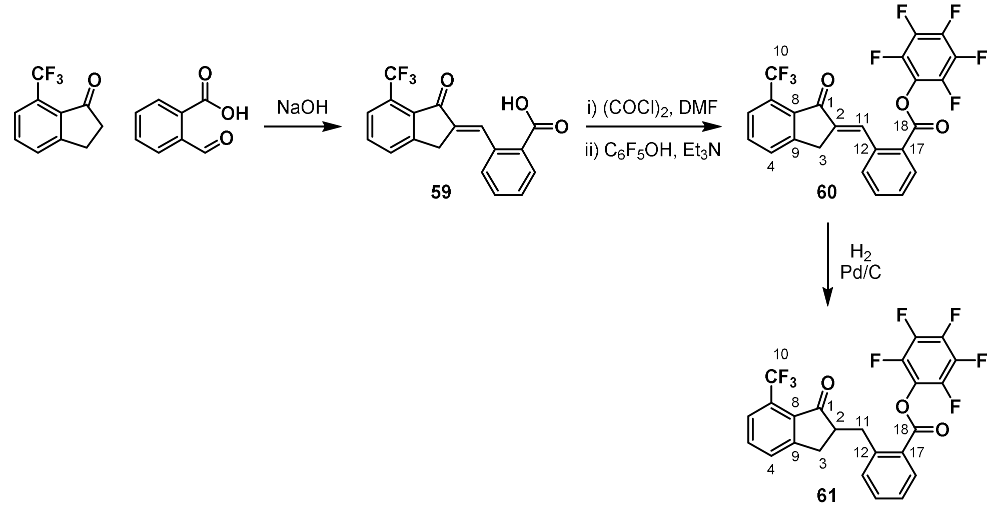


**2-((1-Oxo-7-(trifluoromethyl)-1,3-dihydro-2*H*-inden-2-ylidene)methyl)benzoic acid, 59**

This compound was prepared according to **General Procedure A.**

Aldol condensation with 7-(trifluoromethyl)-1-indanone (100 mg, 0.500 mmol), *o*-carboxybenzaldehyde (75 mg, 0.50 mmol), ethanol (1.20 mL), aqueous sodium hydroxide (1 M, 0.90 mL, 0.90 mmol). Crude unsaturated acid **59** was obtained as a white solid (155 mg).

**Perfluorophenyl 2-((1-oxo-7-(trifluoromethyl)-1,3-dihydro-2*H*-inden-2-ylidene)methyl) benzoate, 60**

This compound was prepared according to **General Procedure B**.

Esterification with crude unsaturated acid **59** (155 mg, 0.466 mmol), oxalyl chloride (60 µL, 0.70 mmol), *N*,*N*-dimethylformamide (1 drop), dichloromethane (1.1 mL) then pentafluorophenol (86 mg, 0.47 mmol), triethylamine (194 µL, 1.40 mmol), dichloromethane (1.1 mL). Purification *via* flash chromatography, eluting with 1:4 ethyl acetate/petrol 40-60 afforded title compound **60** as a yellow solid (131 mg, 53% for 2 steps).

m.p. 154-156 °C (ethyl acetate/petrol 40-60);

$^1$H NMR (500 MHz, $CDCl_3$) $\delta_H$ = 8.30 (dd, *J* = 7.9, 1.4 Hz, 1H, $H_{16}$), 8.24 (t, *J* = 2.4 Hz, 1H, $H_{11}$), 7.75 (td, *J* = 7.7, 1.4 Hz, 1H, $H_{14}$), 7.73–7.67 (m, 4H, $H_4$, $H_5$, $H_6$, $H_{13}$), 7.59 (td, *J* = 7.7, 1.4 Hz, 1H, $H_{15}$), 3.93 (d, *J* = 2.3 Hz, 2H, $H_3$);

$^{13}$C NMR (126 MHz, $CDCl_3$) $\delta_C$ = 189.6, 162.1, 151.8, 138.6, 136.1, 134.9, 134.0 (d, *J* = 1.5 Hz), 133.8 (q, *J* = 6.1 Hz), 133.7, 132.1, 130.1, 130.0, 129.1, 127.8 (q, *J* = 34.7 Hz), 126.9, 125.5 (q, *J* = 6.0 Hz), 122.7 (d, *J* = 273.8 Hz), 31.1;

$^{19}$F NMR (470 MHz, $CDCl_3$) $\delta_F$ = -61.5, -152.3, -157.5, -162.1;

FTIR (neat) ν/cm$^{-1}$ = 2360, 1758, 1708, 1636, 1599, 1521, 1484, 1414, 1322, 1251, 1234, 1203, 1160, 1142, 1101, 1034;

HRMS (ESI$^+$) calculated for $C_{24}H_{11}O_3F_8^+$ = 499.0575, mass found = 499.0576.

**Perfluorophenyl 2-((1-oxo-7-(trifluoromethyl)-2,3-dihydro-1*H*-inden-2-yl)methyl) benzoate, 61**

This compound was prepared according to **General Procedure C**.

Hydrogenation with unsaturated ester **60** (100 mg, 0.201 mmol), palladium on carbon (10 mg), ethyl acetate (2.0 mL). Purification *via* flash chromatography, eluting with 1:9 ethyl acetate/petrol 40-60 afforded title compound **61** as a colourless oil (56 mg, 56%).

$^1$H NMR (500 MHz, $CDCl_3$) $\delta_H$ = 8.24 (dd, *J* = 7.9, 1.4 Hz, 1H, $H_{16}$), 7.81–7.57 (m, 4H, $H_4$, $H_5$, $H_{13}$, $H_{14}$), 7.48 (dd, *J* = 7.7, 1.3 Hz, 1H, $H_6$), 7.43 (td, *J* = 7.7, 1.3 Hz, 1H, $H_{15}$), 3.73 (dd, *J* = 13.4, 5.5 Hz, 1H, $H_{11A}$), 3.25 (dd, *J* = 17.0, 7.9 Hz, 1H, $H_{3A}$), 3.23 (dd, *J* = 13.6, 8.7 Hz, 1H, $H_{11B}$), 3.19–3.11 (m, 1H, $H_2$), 2.94 (dd, *J* = 17.0, 4.7 Hz, 1H, $H_{3B}$);

$^{13}$C NMR (126 MHz, $CDCl_3$) $\delta_C$ = 203.1, 162.9, 155.2, 143.5, 134.1, 134.0, 133.2, 132.4, 132.1, 130.4, 127.1 (q, *J* = 34.5 Hz), 127.1, 126.1, 125.2 (q, *J* = 6.0 Hz), 122.7 (d, *J* = 273.7 Hz), 49.1, 34.8, 32.4;

$^{19}F$ NMR (470 MHz, $CDCl_3$) $\delta_F$ = -61.6, -152.5, -157.8, -162.2;

FTIR (neat) ν/cm$^{-1}$ = 2928, 2360, 2121, 1759, 1724, 1601, 1521, 1487, 1454, 1433, 1325, 1229. 1144, 1112, 1031;

HRMS (ESI$^+$) calculated for $C_{24}H_{12}O_3F_8Na^+$ = 523.0551, mass found = 523.0552.

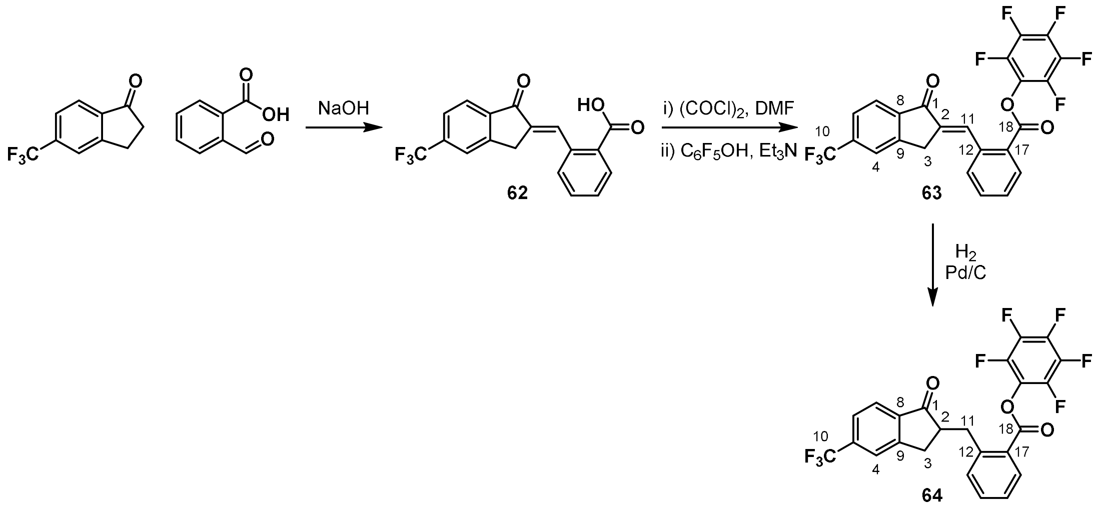


**2-((1-Oxo-5-(trifluoromethyl)-1,3-dihydro-2*H*-inden-2-ylidene)methyl)benzoic acid, 62**

This compound was prepared according to **General Procedure A.**

Aldol condensation with 5-(trifluoromethyl)-1-indanone (70 mg, 0.35 mmol), *o*-carboxybenzaldehyde (53 mg, 0.35 mmol), ethanol (0.84 mL), aqueous sodium hydroxide (1 M, 0.63 mL, 0.63 mmol). Crude unsaturated acid **62** was obtained as a yellow powder (121 mg).

**Perfluorophenyl 2-((1-oxo-5-(trifluoromethyl)-1,3-dihydro-2*H*-inden-2-ylidene)methyl) benzoate, 63**

This compound was prepared according to **General Procedure B**.

Esterification with crude unsaturated acid **62** (120 mg, 0.361 mmol), oxalyl chloride (45.8 µL, 0.542 mmol), *N*,*N*-dimethylformamide (1 drop), dichloromethane (1.5 mL) then pentafluorophenol (66 mg, 0.36 mmol), triethylamine (151 µL, 1.08 mmol), dichloromethane (1.5 mL). Purification *via* flash chromatography, eluting with 1:19 to 1:20 ethyl acetate/petrol 40-60 afforded title compound **63** as a yellow solid (88 mg, 50% for 2 steps).

m.p. 108-110 °C (ethyl acetate/petrol 40-60);

$^1$H NMR (500 MHz, $CDCl_3$) $\delta_H$ = 8.25 (dd, $J$ = 7.9, 1.4 Hz, 1H, $H_{16}$), 8.22 (t, $J$ = 2.3 Hz, 1H, $H_{11}$), 7.95 (d, $J$ = 8.2 Hz, 1H, $H_7$), 7.74–7.60 (m, 4H, $H_4$, $H_6$, $H_{13}$, $H_{14}$), 7.54 (td, $J$ = 7.6, 1.3 Hz, 1H, $H_{15}$), 3.88 (d, $J$ = 1.4 Hz, 2H, $H_3$);

$^{13}$C NMR (126 MHz, $CDCl_3$) $\delta_C$ = 192.3, 162.1, 149.7, 140.7, 138.4, 136.5, 135.9 (q, $J$ = 32.3 Hz), 134.0, 133.9, 132.2, 130.0, 129.3, 126.9, 125.2, 124.9 (q, $J$ = 3.6 Hz), 123.6 (d, $J$ = 273.1 Hz), 123.4 (q, $J$ = 3.9 Hz), 31.2;

$^{19}$F NMR (377 MHz, $CDCl_3$) $\delta_F$ = -62.8, -152.2, -157.4, -162.0;

FTIR (neat) ν/cm$^{-1}$ = 2981, 1761, 1707, 1638, 1521, 1483, 1434, 1382, 1327, 1264, 1236, 1206, 1169, 1131, 1060, 1033;

HRMS (ESI$^+$) calculated for $C_{24}H_{10}O_3F_8Na^+$ = 521.0394, mass found = 521.0393.

**Perfluorophenyl 2-((1-oxo-5-(trifluoromethyl)-2,3-dihydro-1*H*-inden-2-yl)methyl) benzoate, 64**

This compound was prepared according to **General Procedure C**.

Hydrogenation with unsaturated ester **63** (88 mg, 0.18 mmol), palladium on carbon (9 mg), ethyl acetate (1.8 mL). Purification *via* flash chromatography, eluting with 1:19 to 1:9 ethyl acetate/petrol 40-60 afforded title compound **64** as a colourless oil (25 mg, 39%).

$^1$H NMR (400 MHz, $CDCl_3$) $\delta_H$ = 8.18 (dd, $J$ = 8.2, 1.5 Hz, 1H, $H_{16}$), 7.78 (d, $J$ = 8.0 Hz, 1H, $H_7$), 7.61 (s, 1H, $H_4$), 7.59–7.52 (m, 2H, $H_6$, $H_{14}$), 7.42–7.31 (m, 2H, $H_{13}$, $H_{15}$), 3.76–3.61 (m, 1H, $H_{11A}$), 3.19 (dd, $J$ = 17.0, 6.9 Hz, 1H, $H_{3A}$), 3.14–3.03 (m, 2H, $H_2$, $H_{11B}$), 2.89 (dd, $J$ = 17.0, 3.4 Hz, 1H, $H_{3B}$);

$^{13}$C NMR (101 MHz, $CDCl_3$) $\delta_C$ = 206.1, 162.8, 153.3, 143.3, 139.1, 136.1 (q, $J$ = 32.1 Hz), 134.2, 132.2, 132.2, 127.2, 126.1, 124.6 (q, $J$ = 3.6 Hz), 124.6, 123.7 (q, $J$ = 3.9 Hz), 123.6 (d, $J$ = 273.3 Hz), 48.8, 35.0, 32.3;

$^{19}$F NMR (377 MHz, $CDCl_3$) $\delta_F$ = -62.9, -152.5, -157.7, -162.1;

FTIR (neat) ν/cm$^{-1}$ = 2920, 1760, 1721, 1600, 1520, 1490, 1432, 1326, 1228, 1169, 1131, 1059, 1031;

HRMS (ESI$^+$) calculated for $C_{24}H_{12}O_3F_8Na^+$ = 523.0551, mass found = 523.0550.

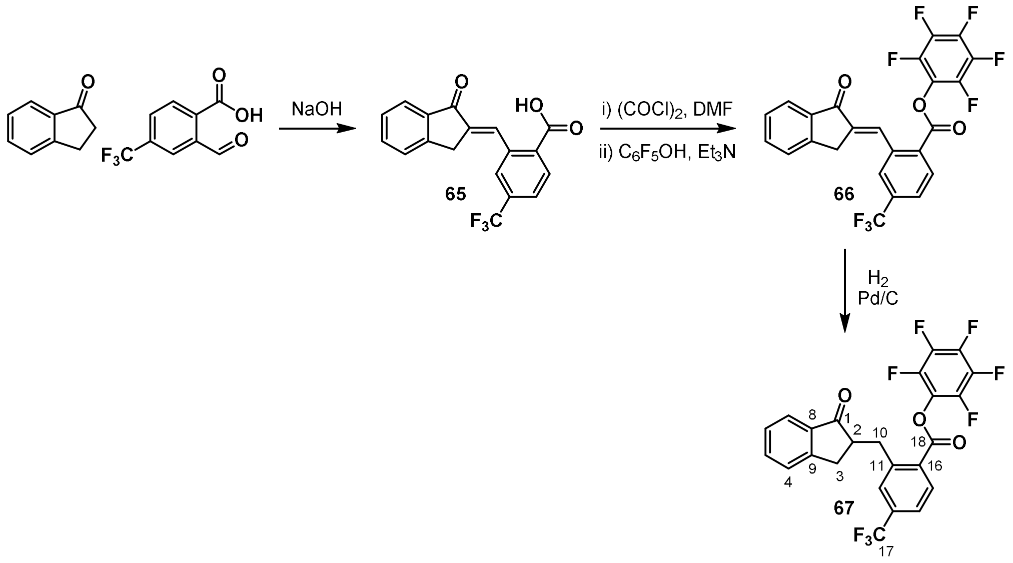

**2-((1-Oxo-1,3-dihydro-2*H*-inden-2-ylidene)methyl)-4-(trifluoromethyl)benzoic acid, 65**

This compound was prepared according to **General Procedure A.**

Aldol condensation with 1-indanone (85 mg, 0.64 mmol), 2-formyl-4-(trifluoromethyl)benzoic acid (140 mg, 0.643 mmol), ethanol (1.55 mL), aqueous sodium hydroxide (1 M, 1.16 mL, 1.16 mmol). Crude unsaturated acid **65** was obtained as a yellow powder (120 mg).

**Perfluorophenyl 2-((1-oxo-1,3-dihydro-2*H*-inden-2-ylidene)methyl)-4-(trifluoromethyl) benzoate, 66**

This compound was prepared according to **General Procedure B**.

Esterification with crude unsaturated acid **65** (120 mg, 0.361 mmol), oxalyl chloride (45.8 μL, 0.542 mmol), *N*,*N*-dimethylformamide (1 drop), dichloromethane (1.5 mL) then pentafluorophenol (67 mg, 0.36 mmol), triethylamine (151 μL, 1.08 mmol), dichloromethane (1.5 mL). Crude unsaturated ester **66** was obtained as a yellow solid (126 mg).

**Perfluorophenyl 2-((1-oxo-2,3-dihydro-1*H*-inden-2-yl)methyl)-4-(trifluoromethyl) benzoate, 67**

This compound was prepared according to **General Procedure C**.

Hydrogenation with crude unsaturated ester **66** (89 mg, 0.18 mmol), palladium on carbon (9 mg), ethyl acetate (1.8 mL). Purification *via* flash chromatography, eluting with 1:19 to 1:9 ethyl acetate/petrol 40-60 afforded title compound **67** as a colourless oil (31 mg, 14% for 3 steps) and recovered starting material **66** (30 mg, 33%).

$^{1}H$ NMR (500 MHz, $CDCl_3$) $\delta_H$ = 8.32 (d, *J* = 8.2 Hz, 1H, $H_{15}$), 7.77 (d, *J* = 7.7 Hz, 1H, $H_7$), 7.75 (d, *J* = 1.7 Hz, 1H, $H_{12}$), 7.69 (dd, *J* = 8.2, 1.8 Hz, 1H, $H_{14}$), 7.59 (td, *J* = 7.5, 1.3 Hz, 1H, $H_5$), 7.43 (d, *J* = 7.7 Hz, 1H, $H_4$), 7.38 (t, *J* = 7.4 Hz, 1H, $H_6$), 3.80 (dd, *J* = 13.8, 5.6 Hz, 1H, $H_{10A}$), 3.25 (dd, *J* = 17.2, 8.1 Hz, 1H, $H_{3A}$), 3.20 (dd, *J* = 13.6, 9.2 Hz, 1H, $H_{10B}$), 3.13–3.02 (m, 1H, $H_2$), 2.89 (dd, *J* = 17.0, 4.4 Hz, 1H, $H_{3B}$);

$^{13}C$ NMR (126 MHz, $CDCl_3$) $\delta_C$ = 206.5, 162.1, 152.9, 144.6, 136.2, 135.2 (q, *J* = 33.0 Hz), 135.0, 132.4, 129.6, 128.8 (q, *J* = 3.7 Hz), 127.6, 126.5, 124.1, 123.8 (q, *J* = 3.7 Hz), 123.2 (d, *J* = 273.1 Hz), 48.2, 35.1, 32.5;

$^{19}F$ NMR (470 MHz, $CDCl_3$) $\delta_F$ = -63.4, -152.3, -157.2, -161.8;

FTIR (neat) ν/cm$^{-1}$ = 3563, 2981, 2925, 2358, 1767 1716, 1521, 1463, 1382, 1332, 1237, 1138, 1084, 1035;

HRMS (ESI$^+$) calculated for $C_{24}H_{13}O_3F_8^+$ = 501.0732, mass found = 501.0731.

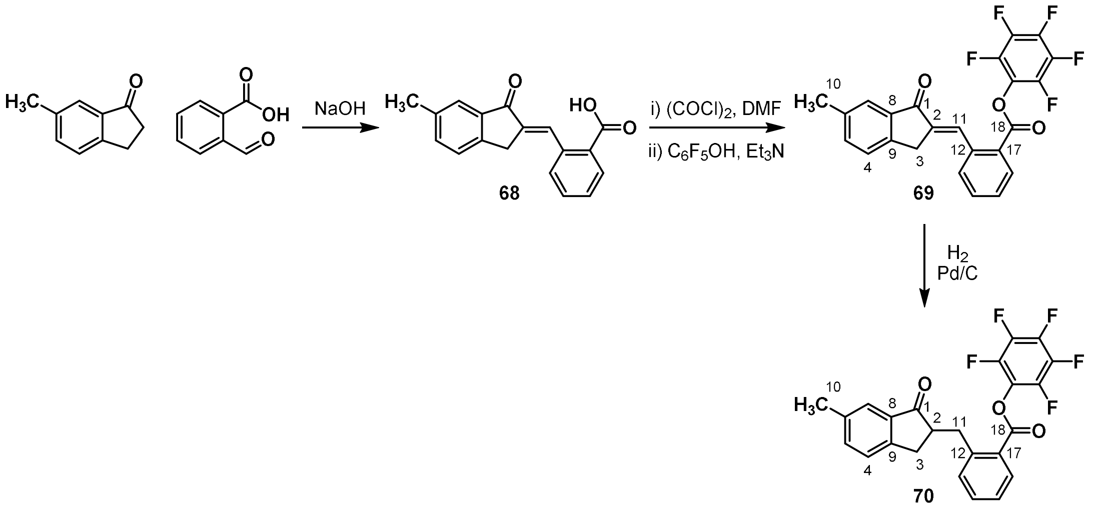


**2-((6-Methyl-1-oxo-1,3-dihydro-2*H*-inden-2-ylidene)methyl)benzoic acid, 68**

This compound was prepared according to **General Procedure A.**

Aldol condensation with 6-methyl-1-indanone (100 mg, 0.684 mmol), *o*-carboxybenzaldehyde (103 mg, 0.684 mmol), ethanol (1.6 mL), aqueous sodium hydroxide (1 M, 1.23 mL, 1.23 mmol). Crude unsaturated acid **68** was obtained as a white solid (146 mg).

**Perfluorophenyl 2-((6-methyl-1-oxo-1,3-dihydro-2*H*-inden-2-ylidene)methyl)benzoate, 69**

This compound was prepared according to **General Procedure B**.

Esterification with crude unsaturated acid **68** (146 mg, 0.525 mmol), oxalyl chloride (66.6 μL, 0.787 mmol), *N*,*N*-dimethylformamide (1 drop), dichloromethane (1.5 mL) then pentafluorophenol (96 mg, 0.53 mmol), triethylamine (218 μL, 1.56 mmol), dichloromethane (1.5 mL). Purification *via* flash chromatography, eluting with 1:9 ethyl acetate/petrol 40-60 afforded title compound **69** as a white solid (88 mg, 29% for 2 steps).

m.p. 152-154 °C (ethyl acetate/petrol 40-60);

$^{1}$H NMR (500 MHz, $CDCl_3$) $\delta_H$ = 8.28 (dd, *J* = 8.0, 1.4 Hz, 1H, $H_{16}$), 8.20 (t, *J* = 2.3 Hz, 1H, $H_{11}$), 7.76–7.69 (m, 3H, $H_7$, $H_{14}$, $H_{13}$), 7.56 (td, *J* = 7.9, 6.9, 1.7 Hz, 1H, $H_{15}$), 7.42 (dd, *J* = 7.7, 1.7 Hz, 1H, $H_5$), 7.37 (dd, *J* = 7.8, 0.9 Hz, 1H, $H_4$), 3.84 (d, *J* = 1.6 Hz, 2H, $H_3$), 2.43 (s, 3H, $H_{10}$);

$^{13}$C NMR (126 MHz, $CDCl_3$) $\delta_C$ = 193.5, 162.2, 147.1, 138.9, 138.2, 138.0, 137.7, 136.0, 133.8, 132.0, 132.0, 130.1, 128.8, 126.9, 125.8, 124.6, 30.8, 21.2;

$^{19}$F NMR (470 MHz, $CDCl_3$) $\delta_F$ = -152.1, -157.7, -162.2;

FTIR (neat) ν/cm$^{-1}$ = 2926, 2361, 1760, 1701, 1637, 1520, 1284, 1234, 1160, 1116, 1093, 1031;

HRMS (ESI$^+$) calculated for $C_{24}H_{14}O_3F_5^+$ = 445.0858, mass found = 445.0859.

**Perfluorophenyl 2-((6-methyl-1-oxo-2,3-dihydro-1*H*-inden-2-yl)methyl)benzoate, 70**

This compound was prepared according to **General Procedure C**.

Hydrogenation with unsaturated ester **69** (85 mg, 0.19 mmol), palladium on carbon (8 mg), ethyl acetate (1.9 mL). Purification *via* flash chromatography, eluting with 1:19 to 1:9 ethyl acetate/petrol 40-60 afforded title compound **70** as a colourless oil (78 mg, 91%).

$^{1}$H NMR (500 MHz, $CDCl_3$) $\delta_H$ = 8.22 (dd, *J* = 7.9, 1.4 Hz, 1H, $H_{16}$), 7.60 (td, *J* = 7.6, 1.4 Hz, 1H, $H_{14}$), 7.56 (d, *J* = 1.6 Hz, 1H, $H_7$), 7.42 (m, 3H, $H_5$, $H_{13}$, $H_{15}$), 7.29 (d, *J* = 7.8 Hz, 1H, $H_4$), 3.75 (dd, *J* = 13.1, 4.9 Hz, 1H, $H_{11A}$), 3.22–3.04 (m, 3H, $H_2$, $H_{3A}$, $H_{11B}$), 2.83 (dd, *J* = 16.6, 3.6 Hz, 1H, $H_{3B}$), 2.39 (s, 3H, $H_{10}$);

$^{13}$C NMR (126 MHz, $CDCl_3$) $\delta_C$ = 207.3, 162.9, 150.6, 143.9, 137.4, 136.6, 136.0, 134.0, 132.2, 132.0, 126.9, 126.2, 126.2, 124.0, 48.8, 35.1, 32.0, 21.1;

$^{19}$F NMR (470 MHz, $CDCl_3$) $\delta_F$ = -152.5, -158.0, -162.3;

FTIR (neat) ν/cm$^{-1}$ = 2927, 1760, 1711, 1618, 1520, 1492, 1282, 1228, 1146, 1113, 1030;

HRMS (ESI$^+$) calculated for $C_{24}H_{16}O_3F_5^+$ = 447.1014, mass found = 447.1014.

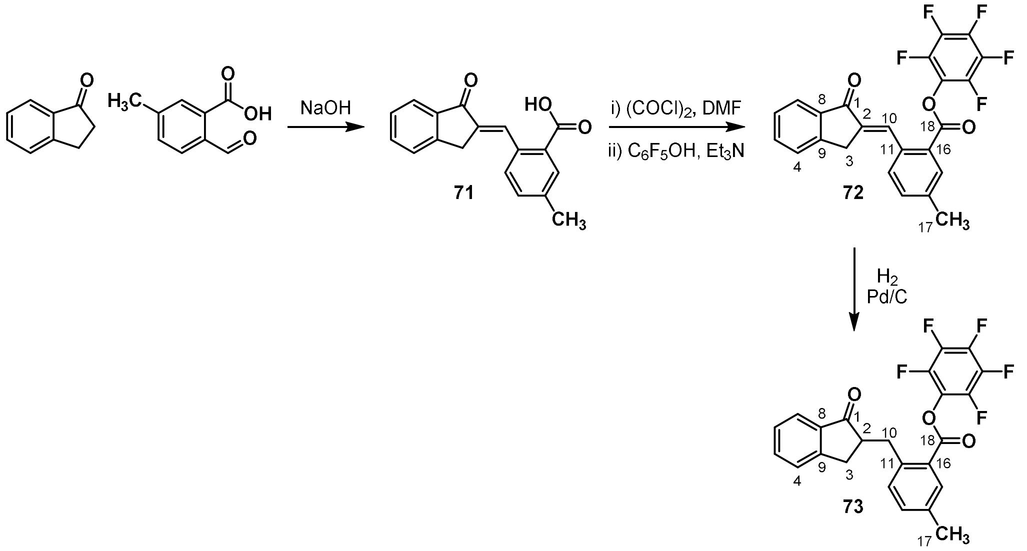


**5-Methyl-2-((1-oxo-1,3-dihydro-2*H*-inden-2-ylidene)methyl)benzoic acid, 71**

This compound was prepared according to **General Procedure A.**

Aldol condensation with 1-indanone (100 mg, 0.757 mmol), 2-formyl-5-methylbenzoic acid (124 mg, 0.757 mmol), ethanol (1.8 mL), aqueous sodium hydroxide (1 M, 1.36 mL, 1.36 mmol). Crude unsaturated acid **71** was obtained as a white solid (140 mg).

**Perfluorophenyl 5-methyl-2-((1-oxo-1,3-dihydro-2*H*-inden-2-ylidene)methyl)benzoate, 72**

This compound was prepared according to **General Procedure B**.

Esterification with crude unsaturated acid **71** (140 mg, 0.500 mmol), oxalyl chloride (63.5 µL, 0.750 mmol), *N*,*N*-dimethylformamide (1 drop), dichloromethane (1.5 mL) then pentafluorophenol (93 mg, 0.50 mmol), triethylamine (211 µL, 1.50 mmol), dichloromethane (1.5 mL). Purification *via* flash chromatography, eluting with 1:9 ethyl acetate/petrol 40-60 afforded title compound **72** as a white solid (170 mg, 50% for 2 steps).

m.p. 110-112 °C (ethyl acetate/petrol 40-60);

$^1$H NMR (500 MHz, $CDCl_3$) $\delta_H$ = 8.21 (t, $J$ = 1.9 Hz, 1H, $H_{10}$), 8.08 (d, $J$ = 1.8 Hz, 1H, $H_{15}$), 7.90 (dt, $J$ = 7.7, 0.9 Hz, 1H, $H_7$), 7.63 (d, $J$ = 8.0 Hz, 1H, $H_{12}$), 7.60 (td, $J$ = 7.5, 1.2 Hz, 1H, $H_5$), 7.54 (dd, $J$ = 7.9, 1.9 Hz, 1H, $H_{13}$), 7.49 (dt, $J$ = 7.7, 1.0 Hz, 1H, $H_4$), 7.42 (td, $J$ = 7.4, 1.0 Hz, 1H, $H_6$), 3.89 (d, $J$ = 2.2 Hz, 2H, $H_3$), 2.51 (s, 3H, $H_{17}$);

$^{13}$C NMR (126 MHz, $CDCl_3$) $\delta_C$ = 193.5, 162.4, 149.7, 139.4, 138.2, 136.9, 135.7, 134.7, 134.6, 132.6, 132.2, 130.1, 127.7, 126.9, 126.1, 124.6, 31.3, 21.2;

$^{19}$F NMR (470 MHz, $CDCl_3$) $\delta_F$ = -152.1, -157.7, -162.2;

FTIR (neat) ν/cm$^{-1}$ = 1758, 1701, 1634, 1609, 1519, 1494, 1468, 1325, 1296, 1253, 1176, 1142, 1092, 1033;

HRMS (ESI$^+$) calculated for $C_{24}H_{14}O_3F_5^+$ = 445.0858, mass found = 445.0857.

**Perfluorophenyl 5-methyl-2-((1-oxo-2,3-dihydro-1*H*-inden-2-yl)methyl)benzoate, 73**

This compound was prepared according to **General Procedure C**.

Hydrogenation with unsaturated ester **72** (140 mg, 0.315 mmol), palladium on carbon (14 mg), ethyl acetate (3.2 mL). Purification *via* flash chromatography, eluting with 1:19 to 1:9 ethyl acetate/petrol 40-60 afforded title compound **73** as a colourless oil (64 mg, 46%).

$^1$H NMR (500 MHz, $CDCl_3$) $\delta_H$ = 8.03 (dd, *J* = 1.8, 0.8 Hz, 1H, $H_{15}$), 7.76 (dt, *J* = 7.7, 1.0 Hz, 1H, $H_7$), 7.56 (td, *J* = 7.5, 1.2 Hz, 1H, $H_5$), 7.43–7.38 (m, 2H, $H_4$, $H_{13}$), 7.38–7.32 (m, 2H, $H_6$, $H_{12}$), 3.70 (dd, *J* = 12.7, 4.5 Hz, 1H, $H_{10A}$), 3.19 (dd, *J* = 17.0, 7.3 Hz, 1H, $H_{3A}$), 3.15–3.02 (m, 2H, $H_2$, $H_{10B}$), 2.87 (dd, *J* = 17.0, 3.9 Hz, 1H, $H_{3B}$), 2.43 (s, 3H, $H_{17}$);

$^{13}$C NMR (126 MHz, $CDCl_3$) $\delta_C$ = 207.3, 163.0, 153.3, 140.7, 136.8, 136.5, 134.8, 134.7, 132.4, 132.1, 127.4, 126.5, 125.9, 124.0, 48.5, 34.7, 32.3, 20.9;

$^{19}$F NMR (470 MHz, $CDCl_3$) $\delta_F$ = -152.5, -158.0, -162.3;

FTIR (neat) ν/cm$^{-1}$ = 2980, 1759, 1714, 1610, 1520, 1465, 1254, 1208, 1176, 1145, 1031;

HRMS (ESI$^+$) calculated for $C_{24}H_{16}O_3F_5^+$ = 447.1014, mass found = 447.1015.

## 1.5.2 Enantioselective *C*-acylation

The enantioselective *C*-acylation was carried out according to the general schemes shown below.

**Asymmetric reaction conditions according to General Procedure D**

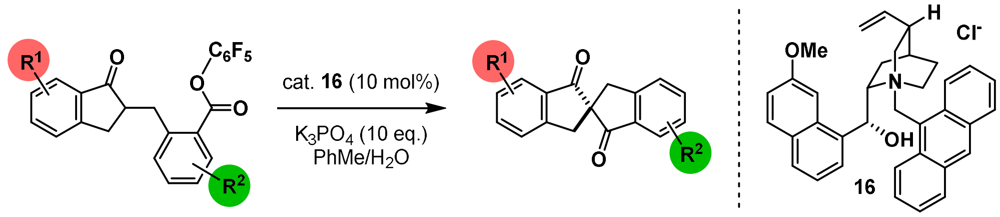


**Racemic reaction conditions according to General Procedure E**

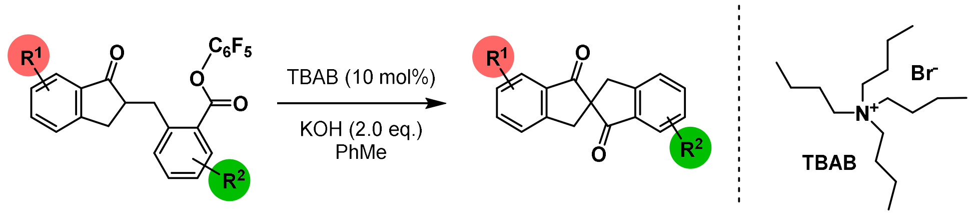


**(*S*)-2,2′-Spirobiindane-1,1′-dione, 9**

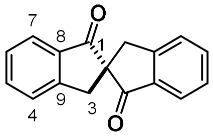

**Asymmetric:** Prepared according to **General Procedure D** with **8** (50 mg, 0.12 mmol), **17** (6.4 mg, 12 µmol), 50% w/w aqueous potassium phosphate (245 µL, 1.15 mmol), toluene (1.1 mL). Reaction conditions: 48 h. Purification *via* flash chromatography, eluting with 1:5 ethyl acetate/petrol 40-60 afforded the title compound **9** as a white solid (27 mg, 93%, 97:3 e.r.). The spectral data for **9** matched that previously reported in the literature.[8]

**Asymmetric (gram scale):** Prepared according to **General Procedure D** with **8** (1.42 g, 3.28 mmol), **17** (181 mg, 0.328 mmol), 50% w/w aqueous potassium phosphate (6.97 mL, 32.8 mmol), toluene (32.8 mL). Reaction conditions: 48 h. Purification *via* flash chromatography, eluting with 1:5 ethyl acetate/petrol 40-60 afforded the title compound **9** as a white solid (750 mg, 92%, 97:3 e.r.). The spectral data for **9** matched those obtained previously.

**Racemic:** Prepared according to **General Procedure E** with **7** (300 mg, 694 µmol), tetrabutylammonium bromide (23 mg, 69 µmol), potassium hydroxide (77.9 mg, 1.39 mmol,), toluene (7.0 mL). Reaction conditions: 16 h. Purification *via* flash chromatography, eluting with 1:5 ethyl acetate/petrol 40-60 afforded the title compound (*rac*)-**9** as a white solid (155 mg, 90%). The spectral data for (*rac*)-**9** matched those obtained previously.

m.p. 170-172 °C (ethyl acetate/petrol 40-60), [lit. 173-176 °C][8];

$^1$H NMR (500 MHz, $CDCl_3$) $\delta_H$ = 7.76 (d, *J* = 7.7Hz, 2H, $H_7$), 7.66 (ddd, *J* =7.9, 7.0, 0.8 Hz, 2H, $H_5$), 7.56 (d, *J* = 7.7 Hz, 2H, $H_4$), 7.41 (dd, *J* = 7.5, 7.5 Hz, 2H, $H_6$), 3.73 (d, *J* = 16.9 Hz, 2H, $H_{3A}$), 3.20 (d, *J* = 16.9 Hz, 2H, $H_{3B}$);

$^{13}$C NMR (126 MHz, $CDCl_3$) $\delta_C$ = 202.7, 153.8, 135.5, 135.3, 127.8, 126.4, 124.9, 65.4, 38.1;

FTIR (film) $\nu_{max}/cm^{-1}$ = 2917, 1720, 1696, 1605, 1586, 1463, 1429, 1325, 1273, 1209, 1186, 1154, 1137, 1088, 1027;

LRMS (ESI$^+$) calculated for $C_{17}H_{12}O_2^+$ = 249, mass found = 249;

$[\alpha]_D^{25}$ = +123.5 (c = 0.65, $CHCl_3$).

**(*S*)-6,6′-Dimethyl-2,2′-spirobiindane-1,1′-dione, 18**

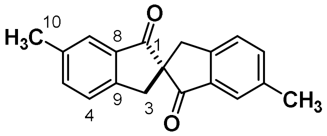


**Asymmetric:** Prepared according to **General Procedure D** with **40** (45 mg, 0.098 mmol), **17** (5.4 mg, 9.8 μmol), 50% w/w aqueous potassium phosphate (208 μL, 0.977 mmol), toluene (0.98 mL). Reaction conditions: 120 h. Purification *via* flash chromatography, eluting with 1:9 ethyl acetate/petrol 40-60 afforded the title compound **18** as a white solid (23 mg, 84%, 92:8 e.r.).

**Racemic:** Prepared according to **General Procedure E** with **40** (4 mg, 9 μmol), tetrabutylammonium bromide (0.3 mg, 1 μmol), potassium hydroxide (1.0 mg, 18 μmol), toluene (0.1 mL). Reaction conditions: 24 h. An analytical HPLC sample of (*rac*)-**18** was prepared by small-scale preparative TLC (1:9 ethyl acetate/petrol 40-60).

m.p. 186-188 °C (ethyl acetate/petrol 40-60);

$^1$H NMR (500 MHz, $CDCl_3$) $\delta_H$ = 7.55 (d, *J* = 1.7 Hz, 2H, $H_7$), 7.47 (dd, *J* = 7.9, 1.7 Hz, 2H, $H_5$), 7.44 (dd, *J* = 7.8, 0.9 Hz, 2H, $H_4$), 3.66 (d, *J* = 16.7 Hz, 2H, $H_{3A}$), 3.13 (d, *J* = 16.8 Hz, 2H, $H_{3B}$), 2.41 (s, 6H, $H_{10}$);

$^{13}$C NMR (126 MHz, $CDCl_3$) $\delta_C$ = 202.9, 151.3, 137.8, 136.5, 135.7, 126.0, 124.8, 66.1, 37.8, 21.1;

FTIR (film) $\nu_{max}/cm^{-1}$ = 2923, 1709, 1689, 1613, 1581, 1494, 1420, 1279, 1220, 1157, 1034;

HRMS (ESI$^+$) calculated for $C_{19}H_{16}O_2Na^+$ = 299.1043, mass found = 299.1043;

$[\alpha]_D^{25}$ = +104.7 (c = 0.5, $CHCl_3$).

**(*S*)-6,6′-Bis(trifluoromethyl)-2,2′-spirobiindane-1,1′-dione, 19**

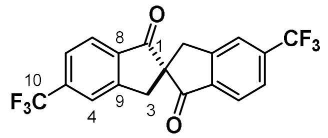


**Asymmetric:** Prepared according to **General Procedure D** with **43** (50 mg, 0.088 mmol), **17** (4.9 mg, 8.8 μmol), 50% w/w aqueous potassium phosphate (187 μL, 0.880 mmol), toluene (0.88 mL). Reaction conditions: 48 h. Purification *via* flash chromatography, eluting with 1:9 ethyl acetate/petrol 40-60 afforded the title compound **19** as a white solid (32 mg, 95%, 95:5 e.r.).

**Racemic:** Prepared according to **General Procedure E** with **43** (5 mg, 9 μmol), tetrabutylammonium bromide (0.3 mg, 0.9 μmol), potassium hydroxide (1.0 mg, 18 μmol), toluene (0.1 mL). Reaction conditions: 24 h. An analytical HPLC sample of (*rac*)-**19** was prepared by small-scale preparative TLC (1:9 ethyl acetate/petrol 40-60).

m.p. 174-176 °C (ethyl acetate/petrol 40-60);

$^1$H NMR (500 MHz, $CDCl_3$) $\delta_H$ = 7.81 (d, $J$ = 7.8 Hz, 2H, $H_7$), 7.80 (s, 2H, $H_4$), 7.63 (dd, $J$ = 7.8, 1.3 Hz, 2H, $H_6$), 3.74 (d, $J$ = 17.3 Hz, 2H, $H_{3A}$), 3.23 (d, $J$ = 17.5 Hz, 2H, $H_{3B}$);

$^{13}$C NMR (126 MHz, $CDCl_3$) $\delta_C$ = 200.9, 153.8, 137.8, 136.9 (q, $J$ = 32.4 Hz), 125.8, 125.3 (q, $J$ = 3.7 Hz), 123.8 (q, $J$ = 3.9 Hz), 123.6 (q, $J$ = 273.3 Hz), 66.2, 37.7;

$^{19}$F NMR (470 MHz, $CDCl_3$) $\delta_F$ = -63.0;

FTIR (film) $\nu_{max}$/cm$^{-1}$ = 3650, 2981, 2361, 1733, 1699, 1433, 1382, 1336, 1299, 1269, 1205, 1169, 1135, 1109, 1057, 1027;

HRMS (EI$^+$) calculated for $C_{19}H_{10}F_6O_2^+$ = 384.0585, mass found = 384.0587;

$[\alpha]_D^{25}$ = +79.4 (c = 0.5, $CHCl_3$).

**(*S*)-5-Bromo-2,2′-spirobiindane-1,1′-dione, 20**

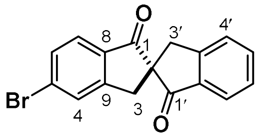


**Asymmetric:** Prepared according to **General Procedure D** with **46** (46 mg, 0.090 mmol),

**17** (5.0 mg, 9.0 μmol), 50% w/w aqueous potassium phosphate (191 μL, 0.900 mmol), toluene (0.90 mL). Reaction conditions: 48 h. Purification *via* flash chromatography, eluting with 1:9 ethyl acetate/petrol 40-60 afforded the title compound **20** as a white solid (27 mg, 91%, 96:4 e.r.).

**Asymmetric (2):** Prepared according to **General Procedure D** with **46** (260 mg, 0.509 mmol), **17** (28 mg, 51 μmol), 50% w/w aqueous potassium phosphate (1.08 mL, 5.09 mmol), toluene (5.1 mL). Reaction conditions: 48 h. Purification *via* flash chromatography, eluting with 1:9 ethyl acetate/petrol 40-60 afforded the title compound **20** as a white solid (151 mg, 91%, 95:5 e.r.). The spectral data for **20** matched those obtained previously.

**Racemic:** Prepared according to **General Procedure E** with **46** (2.0 mg, 3.9 μmol), tetrabutylammonium bromide (0.1 mg, 0.4 μmol), potassium hydroxide (0.4 mg, 8 μmol), toluene (0.1 mL). Reaction conditions: 24 h. An analytical HPLC sample of (*rac*)-**20** was prepared by small-scale preparative TLC (1:9 ethyl acetate/petrol 40-60).

m.p. 52-54 °C (ethyl acetate/petrol 40-60);

$^1$H NMR (400 MHz, $CDCl_3$) $\delta_H$ = 7.77–7.73 (m, 2H, $H_7$, $H_{7'}$), 7.66 (td, *J* = 7.4, 1.2 Hz, 1H, $H_{5'}$), 7.60 (d, *J* = 8.2 Hz, 1H, $H_6$), 7.56 (s, 1H, $H_4$), 7.55 (ddt, *J* = 8.1, 2.2, 1.2 Hz, 1H, $H_{4'}$), 7.41 (td, *J* = 8.4, 7.2, 0.8 Hz, 1H, $H_{6'}$), 3.72 (d, *J* = 12.6 Hz, 1H, $H_{3A}$), 3.68 (d, *J* = 12.7 Hz, 1H, $H_{3A'}$), 3.20 (d, *J* = 6.7 Hz, 1H, $H_{3B}$), 3.16 (d, *J* = 7.0 Hz, 1H, $H_{3B'}$);

$^{13}$C NMR (101 MHz, $CDCl_3$) $\delta_C$ = 202.1, 201.4, 155.3, 153.7, 135.5, 135.2, 134.3, 131.5, 130.9, 129.7, 127.9, 126.4, 126.0, 125.0, 65.4, 37.8, 37.6;

FTIR (film) $\nu_{max}$/cm$^{-1}$ = 2197, 1722, 1694, 1595, 1463, 1421, 1315, 1290, 1264, 1207, 1135, 1058, 1027;

HRMS (ESI$^+$) calculated for $C_{17}H_{11}O_2BrNa^+$= 348.9835, mass found = 384.9837;

$[\alpha]_D^{25}$ = +163.0 (c = 0.5, $CHCl_3$).

**(*S*)-5-Fluoro-2,2′-spirobiindane-1,1′-dione, 21**

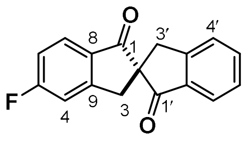


**Asymmetric:** Prepared according to **General Procedure D** with **49** (45 mg, 0.11 mmol), **17** (5.5 mg, 10 μmol), 50% w/w aqueous potassium phosphate (212 μL, 1.00 mmol), toluene (1.0 mL). Reaction conditions: 48 h. Purification *via* flash chromatography, eluting with

3:17 ethyl acetate/petrol 40-60 afforded the title compound **21** as a white solid (26 mg, 98%, 96:4 e.r.).

**Racemic:** Prepared according to **General Procedure E** with **49** (5.0 mg, 11 μmol), tetrabutylammonium bromide (0.3 mg, 1 μmol), potassium hydroxide (1.2 mg, 22 μmol), toluene (0.1 mL). Reaction conditions: 24 h. An analytical HPLC sample of (*rac*)-**21** was prepared by small-scale preparative TLC (3:17 ethyl acetate/petrol 40-60).

m.p. 180-182 °C (ethyl acetate/petrol 40-60);

$^{1}$H NMR (400 MHz, $CDCl_3$) $\delta_H$ = 7.75 (dd, *J* = 8.6, 5.3 Hz, 1H, $H_7$), 7.75 (d, *J* = 7.8 Hz, 1H, $H_{7'}$), 7.65 (td, *J* = 7.5, 1.2 Hz, 1H, $H_{5'}$), 7.56 (dt, *J* = 7.7, 1.0 Hz, 1H, $H_{4'}$), 7.41 (td, *J* = 7.7, 7.1, 0.6 Hz, 1H, $H_{6'}$), 7.22 (dt, *J* = 8.4, 1.9, 1.1 Hz, 1H, $H_4$), 7.11 (td, *J* = 8.7, 2.3 Hz, 1H, $H_6$), 3.72 (d, *J* = 16.9 Hz, 1H, $H_{3A'}$), 3.67 (d, *J* = 17.2 Hz, 1H, $H_{3A}$), 3.19 (d, *J* = 17.0 Hz, 1H, $H_{3B'}$), 3.16 (d, *J* = 17.1 Hz, 1H, $H_{3B}$);

$^{13}$C NMR (101 MHz, $CDCl_3$) $\delta_C$ = 202.4, 200.7, 167.5 (d, *J* = 257.5 Hz), 156.7 (d, *J* = 10.4 Hz), 153.7, 135.4, 135.2, 131.8, 127.9, 127.2 (d, *J* = 10.5 Hz), 126.4, 125.0, 116.2 (d, *J* = 23.9 Hz), 113.1 (d, *J* = 22.9 Hz), 65.6, 37.9, 37.8 (d, *J* = 2.9 Hz);

$^{19}$F NMR (377 MHz, $CDCl_3$) $\delta_F$ = -101.7;

FTIR (film) $\nu_{max}$/cm$^{-1}$ = 2923, 1713, 1688, 1605, 1590, 1475, 1464, 1427, 1328, 1295, 1275, 1252, 1211, 1187, 1132, 1088, 1039;

HRMS (ESI$^+$) calculated for $C_{17}H_{12}O_2F^+$ = 267.0816, mass found = 267.0817;

$[\alpha]_D^{25}$ = +134.6 (c = 0.5, $CHCl_3$).

**(*S*)-4-Methoxy-2,2′-spirobiindane-1,1′-dione, 22**

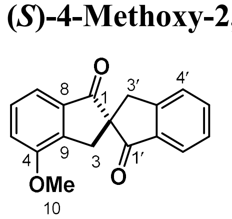


**Asymmetric:** Prepared according to **General Procedure D** with **52** (50 mg, 0.11 mmol), **17** (6.0 mg, 11 μmol), 50% w/w aqueous potassium phosphate (230 μL, 1.08 mmol), toluene (1.1 mL). Reaction conditions: 120 h. Purification *via* flash chromatography, eluting with 1:4 ethyl acetate/petrol 40-60 afforded the title compound **22** as a white solid (30 mg, 99%, 94:6 e.r.).

**Racemic:** Prepared according to **General Procedure E** with **52** (5.0 mg, 11 µmol), tetrabutylammonium bromide (0.4 mg, 1 µmol), potassium hydroxide (1.2 mg, 22 µmol), toluene (0.1 mL). Reaction conditions: 24 h. An analytical HPLC sample of (*rac*)-**22** was prepared by small-scale preparative TLC (1:4 ethyl acetate/petrol 40-60).

m.p. 188-190 °C (ethyl acetate/petrol 40-60);

$^1$H NMR (500 MHz, $CDCl_3$) $\delta_H$ = 7.76 (d, *J* = 7.6 Hz, 1H, $H_{7'}$), 7.65 (td, *J* = 7.5, 1.2 Hz, 1H, $H_{5'}$), 7.56 (dd, *J* = 7.7, 1.5 Hz, 1H, $H_{4'}$), 7.41 (t, *J* = 7.5 Hz, 1H, $H_{6'}$), 7.39 (d, *J* = 6.8 Hz, 1H, $H_7$), 7.35 (dd, *J* = 7.8, 6.7 Hz, 1H, $H_6$), 7.09 (dd, *J* = 7.6, 1.1 Hz, 1H, $H_5$), 3.93 (s, 3H, $H_{10}$), 3.71 (d, *J* = 17.0 Hz, 1H, $H_{3A}$), 3.66 (d, *J* = 17.4 Hz, 1H, $H_{3A'}$), 3.20 (d, *J* = 16.9 Hz, 1H, $H_{3B}$), 3.06 (d, *J* = 17.4 Hz, 1H, $H_{3B'}$);

$^{13}$C NMR (126 MHz, $CDCl_3$) $\delta_C$ = 203.0, 202.8, 156.9, 154.0, 142.9, 137.0, 135.6, 135.4, 129.5, 127.9, 126.5, 125.0, 116.5, 115.6, 65.3, 55.7, 38.3, 35.1;

FTIR (film) $\nu_{max}$/cm$^{-1}$ = 2922, 2852, 1687, 1603, 1488, 1463, 1441, 1422, 1286, 1263, 1212, 1152, 1130, 1080, 1031;

HRMS (ESI$^+$) calculated for $C_{18}H_{15}O_3^+$ = 279.1016, mass found = 279.1017;

$[\alpha]_D^{25}$ = +87.8 (c = 0.5, $CHCl_3$).

**(*S*)-4-Bromo-2,2′-spirobiindane-1,1′-dione, 23**

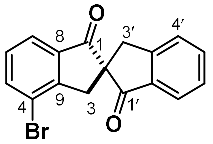


**Asymmetric:** Prepared according to **General Procedure D** with **55** (50 mg, 0.098 mmol), **17** (5.4 mg, 9.8 µmol), 50% w/w aqueous potassium phosphate (208 µL, 0.978 mmol), toluene (0.98 mL). Reaction conditions: 48 h. Purification *via* flash chromatography, eluting with 1:9 ethyl acetate/petrol 40-60 afforded the title compound **23** as a white solid (32 mg, 99%, 93:7 e.r.).

**Racemic:** Prepared according to **General Procedure E** with **55** (2 mg, 4 µmol), tetrabutylammonium bromide (0.1 mg, 0.4 µmol), potassium hydroxide (0.4 mg, 8 µmol), toluene (0.1 mL). Reaction conditions: 24 h. An analytical HPLC sample of (*rac*)-**23** was prepared by small-scale preparative TLC (1:9 ethyl acetate/petrol 40-60).

m.p. 250-252 °C (ethyl acetate/petrol 40-60);

$^1$H NMR (500 MHz, CDCl$_3$) $\delta_H$ = 7.83 (dd, $J$ = 7.8, 1.0 Hz, 1H, H$_7$), 7.77 (d, $J$ = 7.5 Hz, 1H, H$_{7'}$), 7.72 (dd, $J$ = 7.6, 0.9 Hz, 1H, H$_5$), 7.67 (td, $J$ = 7.5, 1.2 Hz, 1H, H$_{5'}$), 7.57 (dt, $J$ = 7.8, 1.0 Hz, 1H, H$_{4'}$), 7.43 (td, $J$ = 7.5, 0.7 Hz, 1H, H$_{6'}$), 7.33 (tt, $J$ = 7.7, 0.9 Hz, 1H, H$_6$), 3.73 (d, $J$ = 17.0 Hz, 1H, H$_{3A'}$), 3.68 (d, $J$ = 17.6 Hz, 1H, H$_{3A}$), 3.24 (d, $J$ = 17.0 Hz, 1H, H$_{3B'}$), 3.12 (d, $J$ = 17.6 Hz, 1H, H$_{3B}$);

$^{13}$C NMR (126 MHz, CDCl$_3$) $\delta_C$ = 202.1, 202.1, 153.7, 153.5, 138.0, 137.4, 135.5, 135.2, 129.6, 128.0, 126.4, 125.1, 123.7, 121.8, 65.2, 39.1, 38.1;

FTIR (film) $\nu_{max}$/cm$^{-1}$ = 3655, 2981, 1718, 1692, 1597, 1462, 1382, 1257, 1151, 1073;

HRMS (ESI$^+$) calculated for $C_{17}H_{11}O_2BrNa^+$ = 348.9835, mass found = 348.9836;

$[\alpha]_D^{25}$ = +112.9 (c = 0.5, CHCl$_3$).

**(*S*)-5-Fluoro-6′-methyl-2,2′-spirobiindane-1,1′-dione, 24**

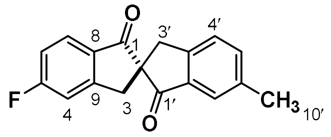


**Asymmetric:** Prepared according to **General Procedure D** with **58** (50 mg, 0.11 mmol), **17** (5.9 mg, 11 µmol), 50% w/w aqueous potassium phosphate (229 µL, 1.08 mmol), toluene (1.1 mL). Reaction conditions: 48 h. Purification *via* flash chromatography, eluting with 3:17 ethyl acetate/petrol 40-60 afforded the title compound **24** as a white solid (30 mg, 99%, 95:5 e.r.).

**Racemic:** Prepared according to **General Procedure E** with **58** (5 mg, 10 µmol), tetrabutylammonium bromide (0.4 mg, 1 µmol), potassium hydroxide (1.2 mg, 22 µmol), toluene (0.1 mL). Reaction conditions: 24 h. An analytical HPLC sample of (*rac*)-**24** was prepared by small-scale preparative TLC (3:17 ethyl acetate/petrol 40-60).

m.p. 170-180 °C (ethyl acetate/petrol 40-60);

$^1$H NMR (400 MHz, CDCl$_3$) $\delta_H$ = 7.75 (dd, $J$ = 8.5, 5.3 Hz, 1H, H$_7$), 7.55 (s, 1H, H$_{7'}$), 7.48 (dd, $J$ = 7.9, 1.6 Hz, 1H, H$_{5'}$), 7.44 (d, $J$ = 7.9 Hz, 1H, H$_{4'}$), 7.22 (dd, $J$ = 8.4, 2.1 Hz, 1H, H$_4$), 7.11 (td, $J$ = 8.6, 2.3 Hz, 1H, H$_6$), 3.69 (d, $J$ = 17.2 Hz, 1H, H$_{3A}$), 3.66 (d, $J$ = 16.8 Hz, 1H, H$_{3A'}$), 3.16 (d, $J$ = 17.2 Hz, 1H, H$_{3B}$), 3.13 (d, $J$ = 16.8 Hz, 1H, H$_{3B'}$), 2.41 (s, 3H, H$_{10'}$);

$^{13}$C NMR (101 MHz, CDCl$_3$) $\delta_C$ = 202.4, 200.8, 167.5 (d, $J$ = 257.4 Hz), 156.7 (d, $J$ = 10.3 Hz), 151.1, 137.9, 136.7, 135.4, 131.8, 127.1 (d, $J$ = 11.0 Hz), 126.0, 124.9, 116.2 (d, $J$ = 23.9 Hz), 113.1 (d, $J$ = 22.9 Hz), 65.9, 37.7 (d, $J$ = 2.3 Hz), 37.6, 21.1;

$^{19}$F NMR (377 MHz, $CDCl_3$) $\delta_F$ = -101.8;

FTIR (film) $\nu_{max}/cm^{-1}$ = 2923, 2852, 1718, 1692, 113, 1592, 1493, 1483, 1427, 1336, 1297, 1281, 1254, 1225, 1156, 1131, 1086, 1032;

HRMS (ESI$^+$) calculated for $C_{18}H_{14}O_2F^+$ = 281.0972, mass found = 281.0972;

$[\alpha]_D^{25}$ = +116.6 (c = 0.5, $CHCl_3$).

**(*S*)-7-(Trifluoromethyl)-2,2′-spirobiindane-1,1′-dione, 25**

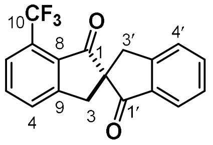


**Asymmetric:** Prepared according to **General Procedure D** with **61** (50 mg, 0.10 mmol), **17** (5.5 mg, 10 µmol), 50% w/w aqueous potassium phosphate (212 µL, 1.00 mmol), toluene (1.0 mL). Reaction conditions: 48 h. Purification *via* flash chromatography, eluting with 1:4 ethyl acetate/petrol 40-60 afforded the title compound **25** as a white solid (28 mg, 88%, 95:5 e.r.).

**Racemic:** Prepared according to **General Procedure E** with **61** (5 mg, 10 µmol), tetrabutylammonium bromide (0.3 mg, 1 µmol), potassium hydroxide (1.2 mg, 20 µmol), toluene (0.1 mL). Reaction conditions: 24 h. An analytical HPLC sample of (*rac*)-**25** was prepared by small-scale preparative TLC (1:4 ethyl acetate/petrol 40-60).

m.p. 168-170 °C (ethyl acetate/petrol 40-60);

$^1$H NMR (400 MHz, $CDCl_3$) $\delta_H$ = 7.78–7.69 (m, 4H, $H_4$, $H_5$, $H_6$, $H_{7'}$), 7.65 (td, $J$ = 7.5, 1.2 Hz, 1H, $H_{5'}$), 7.56 (dt, $J$ = 7.7, 1.0 Hz, 1H, $H_{4'}$), 7.41 (td, $J$ = 7.4, 1.0 Hz, 1H, $H_{6'}$), 3.76 (d, $J$ = 16.9 Hz, 1H, $H_{3A'}$), 3.74 (d, $J$ = 17.1 Hz, 1H, $H_{3A}$), 3.24 (d, $J$ = 17.1 Hz, 1H, $H_{3B}$), 3.20 (d, $J$ = 16.8 Hz, 1H, $H_{3B'}$);

$^{13}$C NMR (101 MHz, $CDCl_3$) $\delta_C$ = 201.5, 198.6, 155.9, 153.7, 135.5, 134.9, 134.5, 132.0, 130.2, 127.9, 127.6, 126.3, 125.6 (q, $J$ = 5.8 Hz), 125.1, 122.5 (q, $J$ = 273.5 Hz), 65.8, 37.7, 37.4;

$^{19}$F NMR (377 MHz, $CDCl_3$) $\delta_F$ = -61.7;

FTIR (film) $\nu_{max}/cm^{-1}$ = 2926, 1730, 1699, 1602, 1509, 1465, 1424, 1324, 1294, 1275, 1250, 1206, 1138, 1113, 1028;

HRMS (ESI$^+$) calculated for $C_{18}H_{12}O_2F_3^+$ = 317.0784, mass found = 317.0787;

$[\alpha]_D^{25}$ = +114.2 (c = 0.5, $CHCl_3$).

**(*S*)-5-(Trifluoromethyl)-2,2′-spirobiindane-1,1′-dione, 26**

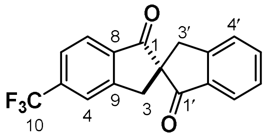


**Asymmetric (1):** Prepared according to **General Procedure D** with **64** (20 mg, 40 μmol), **17** (2.2 mg, 4.0 μmol), 50% w/w aqueous potassium phosphate (85 μL, 0.40 mmol), toluene (0.46 mL). Reaction conditions: 48 h. Purification *via* flash chromatography, eluting with 1:19 ethyl acetate/petrol 40-60 afforded the title compound **26** as a white solid (11 mg, 94%, 95:5 e.r.).

**Asymmetric (2):** Prepared according to **General Procedure D** with **67** (32 mg, 64 μmol), **17** (3.5 mg, 6.4 μmol), 50% w/w aqueous potassium phosphate (136 μL, 0.64 mmol), toluene (0.64 mL). Reaction conditions: 48 h. Purification *via* flash chromatography, eluting with 1:9 ethyl acetate/petrol 40-60 afforded the title compound **26** as a white solid (20 mg, 96%, 93:7 e.r.).

**Racemic:** Prepared according to **General Procedure E** with **64** (2 mg, 4 μmol), tetrabutylammonium bromide (0.1 mg, 0.4 μmol), potassium hydroxide (0.4 mg, 8 μmol), toluene (0.05 mL). Reaction conditions: 24 h. An analytical HPLC sample of (*rac*)-**26** was prepared by small-scale preparative TLC (1:9 ethyl acetate/petrol 40-60).

m.p. 135-137 °C (ethyl acetate/petrol 40-60);

$^1$H NMR (500 MHz, $CDCl_3$) $\delta_H$ = 7.87 (d, *J* = 8.0 Hz, 1H, $H_7$), 7.85 (d, *J* = 1.5 Hz, 1H, $H_4$), 7.77 (d, *J* = 7.7 Hz, 1H, $H_{7'}$), 7.70 – 7.66 (m, 2H, $H_{5'}$, $H_6$), 7.58 (dt, *J* = 7.7, 0.9 Hz, 1H, $H_{4'}$), 7.44 (td, *J* = 7.4, 0.6 Hz, 1H, $H_{6'}$), 3.78 (d, *J* = 17.1 Hz, 1H, $H_{3A}$), 3.75 (d, *J* = 17.0 Hz, 1H, $H_{3A'}$), 3.26 (d, *J* = 17.2 Hz, 1H, $H_{3B}$), 3.23 (d, *J* = 16.9 Hz, 1H, $H_{3B'}$);

$^{13}$C NMR (126 MHz, $CDCl_3$) $\delta_C$ = 201.8, 201.7, 153.9, 153.6, 138.1, 136.5 (q, *J* = 32.3 Hz), 135.6, 135.0, 128.0, 126.4, 125.4, 125.1, 125.0 (q, *J* = 3.6 Hz), 123.6 (q, *J* = 3.9 Hz), 123.6 (q, *J* = 273.3 Hz), 65.7, 37.9, 37.8;

$^{19}$F NMR (470 MHz, $CDCl_3$) $\delta_F$ = -62.9;

FTIR (film) $\nu_{max}$/cm$^{-1}$ = 2981, 1730, 1700, 1607, 1432, 1383, 1328, 1293, 1271, 1209, 1169, 1130, 1059;

HRMS (ESI$^+$) calculated for $C_{18}H_{11}O_2F_3Na^+$ = 339.0603, mass found = 339.0606;

$[\alpha]_D^{25}$ = +90.8 (95:5 e.r., c = 0.5, $CHCl_3$).

**(*S*)-6-Methyl-2,2′-spirobiindane-1,1′-dione, 27**

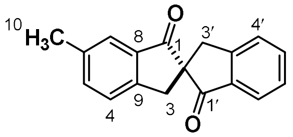


**Asymmetric (1):** Prepared according to **General Procedure D** with **70** (50 mg, 0.11 mmol), **17** (6.2 mg, 11 μmol), 50% w/w aqueous potassium phosphate (238 μL, 1.12 mmol), toluene (1.1 mL). Reaction conditions: 120 h. Purification *via* flash chromatography, eluting with 1:9 ethyl acetate/petrol 40-60 afforded the title compound **27** as a white solid (21 mg, 71%, 99:1 e.r.).

**Asymmetric (2):** Prepared according to **General Procedure D** with **73** (48 mg, 0.11 mmol), **17** (5.9 mg, 11 μmol), 50% w/w aqueous potassium phosphate (230 μL, 1.08 mmol), toluene (1.1 mL). Reaction conditions: 48 h. Purification *via* flash chromatography, eluting with 1:9 ethyl acetate/petrol 40-60 afforded the title compound **27** as a white solid (28 mg, 99%, 96:4 e.r.).

**Racemic:** Prepared according to **General Procedure E** with **73** (4 mg, 9 μmol), tetrabutylammonium bromide (0.3 mg, 0.9 μmol), potassium hydroxide (1.0 mg, 18 μmol), toluene (0.1 mL). Reaction conditions: 24 h. An analytical HPLC sample of (*rac*)-**27** was prepared by small-scale preparative TLC (1:9 ethyl acetate/petrol 40-60).

m.p. 176-178 °C (ethyl acetate/petrol 40-60);

$^1$H NMR (500 MHz, $CDCl_3$) $\delta_H$ = 7.76 (dt, *J* = 7.7, 0.9 Hz, 1H, $H_{7'}$), 7.65 (td, *J* = 7.5, 1.2 Hz, 1H, $H_{5'}$), 7.57–7.54 (m, 2H, $H_{4'}$, $H_7$), 7.48 (dd, *J* = 7.9, 1.6 Hz, 1H, $H_5$), 7.44 (dd, *J* = 7.9, 0.9 Hz, 1H, $H_4$), 7.41 (dq, *J* = 7.6, 0.8 Hz, 1H, $H_{6'}$), 3.72 (d, *J* = 16.9 Hz, 1H, $H_{3A'}$), 3.67 (d, *J* = 16.7 Hz, 1H, $H_{3A}$), 3.19 (d, *J* = 17.0 Hz, 1H, $H_{3B'}$), 3.14 (d, *J* = 16.8 Hz, 1H, $H_{3B}$), 2.42 (s, 3H, $H_{10}$);

$^{13}$C NMR (126 MHz, $CDCl_3$) $\delta_C$ = 202.8, 202.7, 153.9, 151.2, 137.8, 136.6, 135.6, 135.5, 135.2, 127.8, 126.4, 126.0, 124.9, 124.8, 65.7, 38.1, 37.8, 21.1;

FTIR (film) $\nu_{max}$/cm$^{-1}$ = 2924, 2359, 1711, 1691, 1606, 1585, 1494, 1462, 1420, 1323, 1276, 1208, 1189, 1156, 1030;

HRMS (ESI$^+$) calculated for $C_{18}H_{15}O_2^+$ = 263.1067, mass found = 263.1069;

$[\alpha]_D^{25}$ = +133.3 (99:1 e.r., c = 0.5, $CHCl_3$).

## 1.5.3 Derivatization

### (*S*)-3-Bromo-2,2′-spirobiindane-1,1′-dione, 28

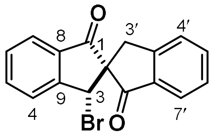


**Asymmetric:** In a 10 mL screw-topped vial, **9** (50 mg, 0.20 mmol, 1.0 eq.), *N*-bromosuccinimide (37 mg, 0.21 mmol, 1.02 eq.) and 2,2′-azobis(2-methylpropionitrile) (1.7 mg, 10 μmol, 0.05 eq.) were dissolved in carbon tetrachloride (3.6 mL). The reaction mixture was heated at reflux for 16 hours, after which it was allowed to cool to room temperature. The mixture was then filtered through a sintered funnel and the residue washed with carbon tetrachloride (15 mL). The filtrate was concentrated under reduced pressure to yield a white solid (65 mg, >20:1 d.r.) which was purified *via* flash chromatography, eluting with toluene, to afford **28** as a white solid (58 mg, 88%, 97:3 e.r., >20:1 d.r.).

**Racemic:** In a 7 mL screw-topped vial, (*rac*)-**9** (20 mg, 81 μmol, 1.0 eq.), *N*-bromosuccinimide (14 mg, 81 μmol, 1.0 eq.), and 2,2′-azobis(2-methylpropionitrile) (0.5 mg, 4 μmol, 0.05 eq.) were dissolved in carbon tetrachloride (1.4 mL). The reaction mixture was heated at reflux for 16 hours, after which it was allowed to cool to room temperature. The mixture was then filtered through a sintered funnel and the residue washed with carbon tetrachloride (10 mL). The filtrate was concentrated under reduced pressure and purified *via* preparatory TLC, eluting with toluene, to afford **28** as a white solid (15 mg, 57%, >20:1 d.r.).

m.p. 80-82 °C (toluene);

$^1$H NMR (500 MHz, $CDCl_3$) $\delta_H$ = 7.85–7.73 (m, 3H, $H_4$, $H_7$, $H_{7'}$), 7.71–7.66 (m, 2H, $H_5$, $H_{5'}$), 7.62 (dd, $J$ = 7.9, 0.8 Hz, 1H, $H_{4'}$), 7.52 (td, $J$ = 7.3, 1.4 Hz, 1H, $H_6$), 7.42 (ddd, $J$ = 8.0, 7.2, 1.0 Hz, 1H, $H_{6'}$), 5.91 (s, 1H, $H_3$), 3.76 (d, $J$ = 17.4 Hz, 1H, $H_{3A'}$), 3.72 (d, $J$ = 17.4 Hz, 1H, $H_{3B'}$);

$^{13}$C NMR (126 MHz, $CDCl_3$) $\delta_C$ = 199.2, 197.9, 154.5, 153.8, 136.0, 135.9, 134.3, 133.8, 130.1, 128.0, 127.1, 126.3, 125.1, 124.9, 71.4, 50.7, 37.8;

FTIR (film) $\nu_{max}$/cm$^{-1}$ = 2919, 2850, 2362, 1728, 1703, 1603, 1465, 1423, 1329, 1271, 1211, 1190, 1153;

Compound **28** was not detected by ACI, EI or ESI mass spectrometry;

$[\alpha]_D^{25}$ = +173.0 (c = 0.5, $CHCl_3$).

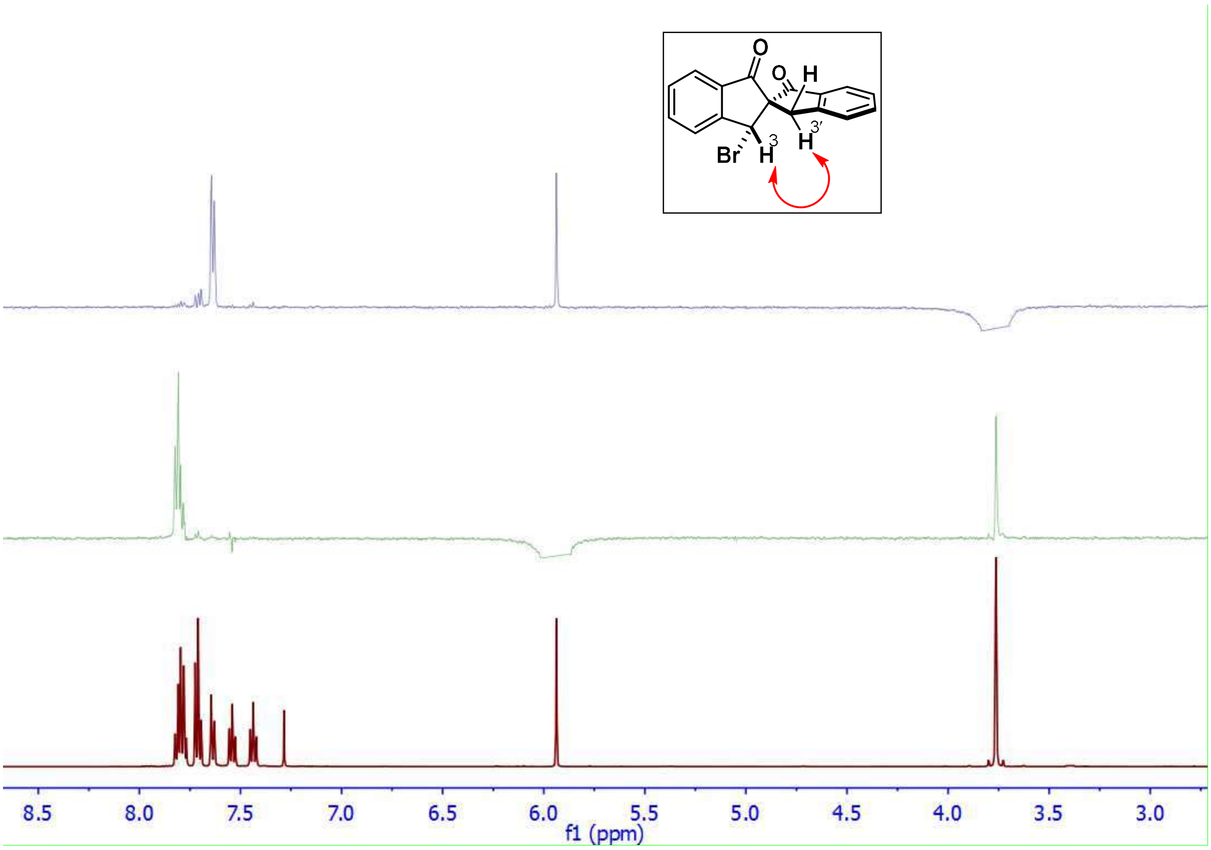


**Figure 1.1** 1D nOe data for compound **28** in $CDCl_3$ (mixing time = 0.8 s).

**(*S*)-*cis*,*cis*-2,2′-Spirobiindane-1,1′-diol, 29**

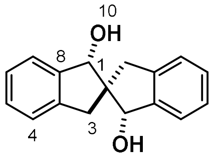


**Asymmetric:** *tert*-Butyllithium (1.7 M in pentane, 376 µL, 0.604 mmol, 3.0 eq.) was slowly added to a solution of DIBAL-H (1.0 M in THF, 604 µL, 0.604 mmol, 3.0 eq.) at -78 °C. The yellow solution was stirred for 5 minutes, warmed to room temperature, at which point the solution turned colourless, and then cooled to -78 °C. To this solution, a suspension of **9** (50 mg, 0.20 mmol, 1.0 eq.) in THF (1.5 mL) was added dropwise over 10 minutes (0.3 mL additional THF was used to rinse in the remaining suspension from the syringe) and the reaction mixture was stirred for 10 hours at -78 °C. Saturated aqueous ammonium chloride at -78 °C was added and the mixture allowed to warm to room temperature, after which time the mixture was poured into a beaker containing chloroform (10 mL) and saturated aqueous ammonium chloride (5 mL) and stirred for 30 minutes. The precipitated aluminium salts were removed *via* filtration through Celite® and the biphasic mixture extracted with chloroform (10 mL × 3). The combined organic extracts were dried over anhydrous sodium sulfate, filtered and concentrated

under reduced pressure to afford a white solid (45 mg, 91%, >20:1:1 d.r.). The residue was purified *via* flash chromatography, eluting with 1:49 ethanol/chloroform, to afford **29** as a white solid (45 mg, 91%, 97:3 e.r., >20:1:1 d.r.). The spectral data for **29** matched that previously reported in the literature.[8]

**Asymmetric (2):** *tert*-Butyllithium (1.7 M in pentane, 376 µL, 0.604 mmol, 3.0 eq.) was slowly added to a solution of DIBAL-H (1.0 M in hexane, 665 µL, 0.665 mmol, 3.3 eq.) at -78 °C. The colourless solution was stirred for 5 minutes, warmed to room temperature, and then cooled to -78 °C. To this solution, a suspension of **9** (50 mg, 0.20 mmol, 1.0 eq.) in THF (1.5 mL) was added dropwise over 10 minutes (0.3 mL additional THF was used to rinse in the remaining suspension from the syringe) and the reaction mixture was stirred for 10 hours at -78 °C. Saturated aqueous ammonium chloride was added at -78 °C and the mixture allowed to warm to room temperature, after which time the mixture was poured into a beaker containing chloroform (10 mL) and saturated aqueous ammonium chloride (5 mL) and stirred for 30 minutes. The precipitated aluminium salts were removed *via* filtration through Celite® and the biphasic mixture extracted with chloroform (10 mL × 3). The combined organic extracts were dried over anhydrous sodium sulfate, filtered and concentrated under reduced pressure to afford a white solid (48 mg, 95% 7:1:0 d.r.). The residue was purified *via* flash chromatography, eluting with 1:99 ethanol/chloroform, to afford **29** as a white solid (39 mg, 78%, 99:1 e.r., >20:1:1 d.r.). The spectral data for **29** matched those obtained previously.

**Racemic:** Prepared according to a literature procedure.[8]

*tert*-Butyllithium (1.7 M in pentane, 376 µL, 0.604 mmol, 3.0 eq.) was slowly added to a solution of DIBAL-H (1.0 M in THF, 604 µL, 0.604 mmol, 3.0 eq.) at -78 °C. The yellow solution was stirred for 5 minutes, warmed to room temperature, at which point the solution turned colourless, and then cooled to -78 °C. To this solution, a suspension of (*rac*)-**9** (50 mg, 0.20 mmol, 1.0 eq.) in THF (1.5 mL) was added (0.3 mL additional THF was used to rinse in the remaining suspension from the syringe) and the stirred reaction mixture was allowed to warm to room temperature overnight. Saturated aqueous ammonium chloride was added and the mixture was poured into a beaker containing chloroform (10 mL) and saturated aqueous ammonium chloride (5 mL) and stirred for 30 minutes. The precipitated aluminium salts were removed *via* filtration through Celite® and the biphasic mixture extracted with chloroform (10 mL × 3). The combined organic extracts were dried over anhydrous sodium sulfate, filtered and concentrated under reduced pressure to afford a white solid (48 mg, 96%, >20:1:1 d.r.). The residue was purified *via* flash chromatography, eluting with 1:49 ethanol/chloroform, to

afford (*rac*)-**29** as a white solid (48 mg, 96%, >20:1:1 d.r.). The spectral data for (*rac*)-**29** matched those obtained previously.

m.p. 196-198 °C (ethanol/chloroform), [lit. 234-6 °C][8];

$^1$H NMR (500 MHz, $CDCl_3$) $\delta_H$ = 7.48 (dd, *J* = 6.0, 2.4 Hz, 2H, $H_4$), 7.34–7.27 (m, 4H, $H_5$, $H_6$), 7.23 (dd, *J* = 6.0, 2.4 Hz, 2H, $H_7$), 5.19 (d, *J* = 3.2 Hz, 2H, $H_1$), 3.17 (d, *J* = 15.6 Hz, 2H, $H_{3A}$), 2.95 (d, *J* = 3.5 Hz, 2H, $H_{10}$), 2.55 (d, *J* = 15.5 Hz, 2H, $H_{3B}$);

$^1$H NMR (500 MHz, $(CD_3)_2SO$) $\delta_H$ = 7.39 (dd, *J* = 7.5, 1.9 Hz, 2H, $H_4$), 7.26–7.19 (m, 6H, $H_5$, $H_6$, $H_7$), 5.41 (d, *J* = 2.9 Hz, 2H, $H_{10}$), 4.97 (d, *J* = 3.0 Hz, 2H, $H_1$), 3.02 (d, *J* = 15.3 Hz, 2H, $H_{3A}$), 2.39 (d, *J* = 15.3 Hz, 2H, $H_{3B}$);

$^{13}$C NMR (126 MHz, $(CD_3)_2SO$) $\delta_C$ = 145.2, 143.2, 128.5, 126.9, 125.5, 80.4, 58.8, 42.2;
N.B. only 8 carbon peaks in the $^{13}$C spectrum due to two coincidental peaks at 125.5 ppm.

FTIR (film) $\nu_{max}$/cm$^{-1}$ = 3365, 3362, 2981, 2892, 1708, 1475, 1462, 1383, 1250, 1152, 1072, 1028;

LRMS (ESI$^+$) calculated for $C_{17}H_{16}O_2Na^+$ = 275, mass found = 275;

$[\alpha]_D^{25}$ = +38.0 (97:3 e.r., c = 0.10, acetone).

**(*S*)-5-(4-Fluorophenyl)-2,2′-spirobiindane-1,1′-dione, 30**

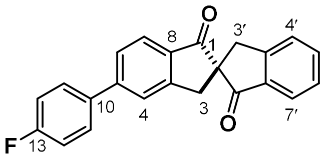


This compound was prepared in analogy to a literature procedure.[9]

**Asymmetric: 20** (95:5 e.r., 30 mg, 0.092 mmol, 1.0 eq.) and 4-fluorophenylboronic acid (19 mg, 0.14 mmol, 1.5 eq.) were dissolved in toluene (0.64 mL) and aqueous sodium carbonate (2 M, 0.28 mL, 0.55 mmol, 6.0 eq.). The mixture was degassed by freeze-pump-thawing[2] (x 3) and flushed with nitrogen. Under a stream of nitrogen, tetrakis(triphenylphosphine)palladium(0) (5.3 mg, 4.6 μmol, 0.05 eq.) was added and the mixture heated at reflux for 7 hours. After cooling to room temperature, the phases were separated and the aqueous layer washed with ethyl acetate (5 mL × 2). The combined organic extracts were dried over anhydrous sodium sulfate, filtered and concentrated under reduced pressure. The residue was purified *via* flash chromatography, eluting with 1:4 ethyl acetate/petrol 40-60, to afford **30** as a white solid (86%, 27 mg, 95:5 e.r.).

**Racemic:** (*rac*)-**20** (10 mg, 31 μmol, 1.0 eq.) and 4-fluorophenylboronic acid (6.4 mg, 45 μmol, 1.5 eq.) were dissolved in toluene (0.21 mL) and aqueous sodium carbonate (2 M, 90 μL, 0.18 mmol, 6.0 eq.). The mixture was degassed by freeze-pump-thawing[2] (x 3) and flushed with nitrogen. Under a stream of nitrogen, tetrakis(triphenylphosphine)palladium(0) (2 mg, 0.002 mmol, 0.05 eq.) was added and the mixture heated at reflux for 7 hours. After cooling to room temperature, the phases were separated and the aqueous layer washed with ethyl acetate (2 mL × 2). The combined organic extracts were dried over anhydrous sodium sulfate, filtered and concentrated under reduced pressure. The residue was purified *via* preparatory TLC, eluting with 1:4 ethyl acetate/petrol 40-60, to afford (*rac*)-**30** as a white solid (4 mg, 38%).

m.p. 186-188 °C (ethyl acetate/petrol 40-60);

$^1$H NMR (500 MHz, $CDCl_3$) $\delta_H$ = 7.82 (d, *J* = 8.0 Hz, 1H, $H_7$), 7.78 (d, *J* = 7.7 Hz, 1H, $H_{7'}$), 7.71 (dd, *J* = 1.6, 0.8 Hz, 1H, $H_4$), 7.67 (td, *J* = 7.5, 1.2 Hz, 1H, $H_{5'}$), 7.63 (ddt, *J* = 8.8, 5.2, 3.0 Hz, 2H, $H_{11}$), 7.59 (dd, *J* = 7.3, 1.4 Hz, 1H, $H_6$), 7.58 (dt, *J* = 7.5, 0.8 Hz, 1H, $H_{4'}$), 7.43 (td, *J* = 7.4, 0.6 Hz, 1H, $H_{6'}$), 7.18 (tt, *J* = 8.5, 2.0 Hz, 2H, $H_{12}$), 3.77 (d, *J* = 16.9 Hz, 1H, $H_{3A}$), 3.76 (d, *J* = 16.9 Hz, 1H, $H_{3A'}$), 3.24 (d, *J* = 17.0 Hz, 1H, $H_{3B}$), 3.22 (d, *J* = 16.9 Hz, 1H, $H_{3B'}$);

$^{13}$C NMR (126 MHz, $CDCl_3$) $\delta_C$ = 201.7, 201.0, 162.1 (d, *J* = 248.5 Hz), 153.5, 152.8, 146.3, 135.2 (d, *J* = 3.3 Hz), 134.4, 134.3, 133.3, 128.2 (d, *J* = 8.4 Hz), 126.8, 126.1, 125.3, 124.3, 123.9, 123.7, 115.0 (d, *J* = 21.7 Hz), 64.6, 37.0;

N.B. only 20 carbon peaks in the $^{13}$C spectrum due to two coincidental peaks at 37.0 ppm.

$^{19}$F NMR (470 MHz, $CDCl_3$) $\delta_F$ = -113.6;

FTIR (film) $\nu_{max}$/cm$^{-1}$ = 2919, 2850, 1720, 1694, 1605, 1517, 1463, 1425, 1275, 1236, 1161, 1025;

HRMS (EI$^+$) calculated for $C_{23}H_{15}O_2F^+$ = 342.1056, mass found = 342.1054;

$[\alpha]_D^{25}$ = +29.2 (c = 0.5, $CHCl_3$).

**(*S*)-5-(4,4,5,5-Tetramethyl-1,3,2-dioxaborolan-2-yl)-2,2′-spirobiindane-1,1′-dione, 31**

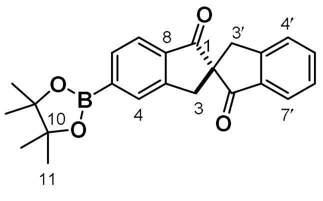

This compound was prepared in analogy to a literature procedure.[10]

A 3.5 mL screw-topped vial was charged with **20** (95:5 e.r., 30 mg, 0.092 mmol, 1.0 eq.), bis(pinacolato)diboron (28 mg, 0.11 mmol, 1.2 eq.), potassium acetate (27 mg, 0.27 mmol, 3.0 eq.) and [1,1′-bis(diphenylphosphino)ferrocene]dichloropalladium(II) (3.4 mg, 4.6 μmol, 0.05 eq.) under an atmosphere of argon. To this was added 1,4-dioxane (0.5 mL) and the mixture heated at reflux for 24 hours. After cooling to room temperature, the mixture was diluted with ethyl acetate (5 mL) and water (5 mL), and filtered through Celite®. The layers were separated and the aqueous layer extracted with ethyl acetate (5 mL × 2). The combined organic extracts were washed with brine (10 mL), dried over anhydrous magnesium sulfate, and concentrated under reduced pressure. The residue was purified *via* flash chromatography, eluting with 1:4 ethyl acetate/petrol 40-60, to afford **31** as a white solid (24 mg, 70%).

m.p. 194-196 °C (ethyl acetate/petrol 40-60);

$^{1}$H NMR (500 MHz, $CDCl_3$) $\delta_H$ = 8.02 (d, *J* = 1.9 Hz, 1H, $H_4$), 7.84 (dd, *J* = 7.7, 1.0 Hz, 1H, $H_7$), 7.75 (dt, *J* = 7.4, 0.6 Hz, 1H, $H_6$), 7.74 (dd, *J* = 7.4, 0.5 Hz, 1H, $H_{7'}$), 7.65 (td, *J* = 7.5, 1.2 Hz, 1H, $H_{5'}$), 7.56 (dt, *J* = 7.7, 0.9 Hz, 1H, $H_{4'}$), 7.41 (td, *J* = 7.4, 1.0 Hz, 1H, $H_{6'}$), 3.73 (d, *J* = 17.0 Hz, 1H, $H_{3A'}$), 3.72 (d, *J* = 16.9 Hz, 1H, $H_{3A}$), 3.19 (d, *J* = 17.1 Hz, 1H, $H_{3B'}$), 3.18 (d, *J* = 16.9 Hz, 1H, $H_{3B}$), 1.38 (s, 12H, $H_{11}$);

$^{13}$C NMR (126 MHz, $CDCl_3$) $\delta_C$ = 203.0, 202.5, 153.8, 152.8, 137.4, 135.4, 135.3, 133.8, 132.7, 127.8, 126.4, 124.9, 123.9, 84.4, 65.6, 38.1, 38.0, 24.9;
N.B. $C_5$ not observed in $^{13}$C NMR spectrum.

FTIR (film) $\nu_{max}$/cm$^{-1}$ = 2977, 2921, 1725, 1698, 1607, 1487, 1412, 1360, 1337, 1266, 1208, 1142, 1073, 1027;

HRMS (ACI$^+$) calculated for $C_{23}H_{23}BO_4^+$ = 375.1775, mass found = 375.1762;

$[\alpha]_D^{25}$ = +86.3 (c = 1.0, $CHCl_3$).

To calculate the e.r. of **31**, it was converted to **30** in analogy to a literature procedure, assuming no degradation in enantioenrichment in this process.[11]

To a solution of **31** (20 mg, 0.05 mmol, 1.0 eq.) in degassed toluene (2.9 mL) and water (0.57 mL) were added 1-fluoro-4-iodobenzene (61 μL, 0.53 mmol, 10.0 eq.), tetrakis(triphenylphosphine)palladium(0) (6 mg, 5 μmol, 0.10 eq.), and cesium carbonate (52 mg, 0.16 mmol, 3.0 eq.) at room temperature. The mixture was heated at 100 °C for

12 hours and then cooled to room temperature. The mixture was diluted with water (2 mL) and extracted with ethyl acetate (5 mL × 3). The combined organic extracts were dried over anhydrous sodium sulfate, filtered and concentrated under reduced pressure. The residue was purified *via* flash chromatography, eluting with 1:4 ethyl acetate/petrol 40-60, to afford **30** as a white solid (12 mg, 66%, 95:5 e.r.). The data matched those obtained previously.

## 1.6 Determination of Absolute Stereochemistry

**Absolute configuration of 20:** Product **20** was recrystallized *via* vapour diffusion by dissolving 5 mg of **20** at 96:4 e.r. in benzene (*c.a.* 50 µL) and using pentane as the volatile component. Low temperature[12] single crystal X-ray diffraction studies were carried out at 150 K on **20** using $CuK_\alpha$ radiation on an Oxford Diffraction SuperNova diffractometer equipped with an area detector and graphite monochromator, within the University of Oxford Chemistry Department. Raw frame data were reduced using CrysAlisPro(Agilent) and the structures were solved using superflip.[13] Full-matrix least-squares refinement of the structures were carried out using CRYSTALS.[14] A Flack x parameter[15] refined to -0.02(1) and Bayesian analysis of the Bijvoet pairs[16] gave the Hooft y parameter as 0.02(5) and the probability that the structure was the correct hand of >99.99% given that the crystal was enantiopure, thus determining the absolute configuration. After completion of the data collection, the crystal used in the single crystal X-ray diffraction studies was compared to the bulk material by dissolution of the crystal in isopropanol and subsequent analysis *via* chiral HPLC. The observed retention time matched that of the major enantiomer of **20** (see S97), thus confirming that the crystal used in the single crystal X-ray diffraction studies comprised of the major enantiomer of **20**. Crystallographic data have been deposited with the Cambridge Crystallographic Data Centre (CCDC 1486386) and copies of these data can be obtained free of charge via www.ccdc.cam.ac.uk/data_request/cif.

## 1.7 References


[1] A. B. Pangborn, M. A. Giardello, R. H. Grubbs, R. K. Rosen, F. J. Timmers, *Organometallics* **1996**, *15*, 1518-1520.

[2] W. L. F. Armarego, D. D. Perrin, *Purification of Laboratory Chemicals*, Butterworth-Heinemann, Oxford, **1996**.

[3] M.-J. Luche-Ronteix, S. Bory, M. Dvolaitsky, R. Lett, A. Marquet, *Bull. Soc. Chim. Fr.* **1970**, 2564-2572.

[4] a) R. Ceccarelli, S. Insogna, M. Bella, *Org. Biomol. Chem.* **2006**, *4*, 4281-4284; b) M. M. Lian, Z. Li, J. A. Du, Q. W. Meng, Z. X. Gao, *Eur. J. Org. Chem.* **2010**, 6525-6530; c) T. Perrard, J. C. Plaquevent, J. R. Desmurs, D. Hebrault, *Org. Lett.* **2000**, *2*, 2959-2962.

[5] a) B. Gomez-Lor, O. de Frutos, P. A. Ceballos, T. Granier, A. M. Echavarren, *Eur. J. Org. Chem.* **2001**, 2107-2114; b) W. Liu, M. Buck, N. Chen, M. Shang, N. J. Taylor, J. Asoud, X. Wu, B. B. Hasinoff, G. I. Dmitrienko, *Org. Lett.* **2007**, *9*, 2915-2918; c) J. N. Levy, C. M. Latham, L. Roisin, N. Kandziora, P. Di Fruscia, A. J. P. White, S. Woodward, M. J. Fuchter, *Org. Biomol. Chem.* **2012**, *10*, 512-515; d) S. E. Walker, C. J. C. Lamb, N. A. Beattie, P. Nikodemiak, A. L. Lee, *Chem. Commun.* **2015**, *51*, 4089-4092.

[6] a) J. Maresh, J. Zhang, Y. L. Tzeng, N. A. Goodman, D. G. Lynn, *Bioorg. Med. Chem. Lett.* **2007**, *17*, 3281-3286; b) F. Faigl, A. Thurner, B. Molnar, G. Simig, B. Volk, *Org. Process Res. Dev.* **2010**, *14*, 617-622; c) K. Vamshikrishna, P. Srihari, *Tetrahedron* **2012**, *68*, 1540-1546; d) M. Dischmann, T. Frassetto, M. A. Breuning, U. Koert, *Chem. Eur. J.* **2014**, *20*, 11300-11302; e) M. Dittmann, F. F. Graupner, B. Maerz, S. Oesterling, R. de Vivie-Riedle, W. Zinth, M. Engelhard, W. Luttke, *Angew. Chem., Int. Ed.* **2014**, *53*, 591-594; f) K. C. Nicolau, M. Lu, D. Mandal, S. Gangwar, N. S. Chowdari, Y. B. Poudel, *WO2015/23879*, **2015**, 111

[7] A. Martinez, M. Fernandez, J. C. Estevez, R. J. Estevez, L. Castedo, *Tetrahedron* **2005**, *61*, 1353-1362.

[8] J. A. Nieman, B. A. Keay, *Tetrahedron: Asymmetry* **1995**, *6*, 1575-1583.

[9] M. A. E. P. B. Mendieta, M. Negri, C. Jagusch, U. M. Vieira, T. Lauterbach, R. W. Hartmann, *J. Med. Chem.* **2008**, *51*, 5009-5018.

[10] S. K. Battacharya, K. O. Cameron, D. P. Fernando, K. F. Mcclure, D. W. Kung, A. T. Londregan, S. T. M. Simila, *US2011/230461 A1*, **2011**, 26

[11] T. Niwa, H. Ochiai, Y. Watanabe, T. Hosoya, *J. Am. Chem. Soc.* **2015**, *137*, 14313-14318.

[12] J. Cosier, A. M. Glazer, *J. Appl. Crystallogr.* **1986**, *19*, 105-107.

[13] L. Palatinus, G. Chapuis, *J. Appl. Crystallogr.* **2007**, *40*, 786-790.

[14] a) P. W. Betteridge, J. R. Carruthers, R. I. Cooper, K. Prout, D. J. Watkin, *J. Appl. Crystallogr.* **2003**, *36*, 1487-1487; b) R. I. Cooper, A. L. Thompson, D. J. Watkin, *J. Appl. Crystallogr.* **2010**, *43*, 1100-1107.

[15] a) H. D. Flack, *Acta Crystallogr. Sect. A* **1983**, *39*, 876-881; b) H. D. Flack, G. Bernardinelli, *J. Appl. Crystallogr.* **2000**, *33*, 1143-1148.

[16] R. W. W. Hooft, L. H. Straver, A. L. Spek, *J. Appl. Crystallogr.* **2008**, *41*, 96-103.

## 1.8 NMR Spectra and HPLC Traces

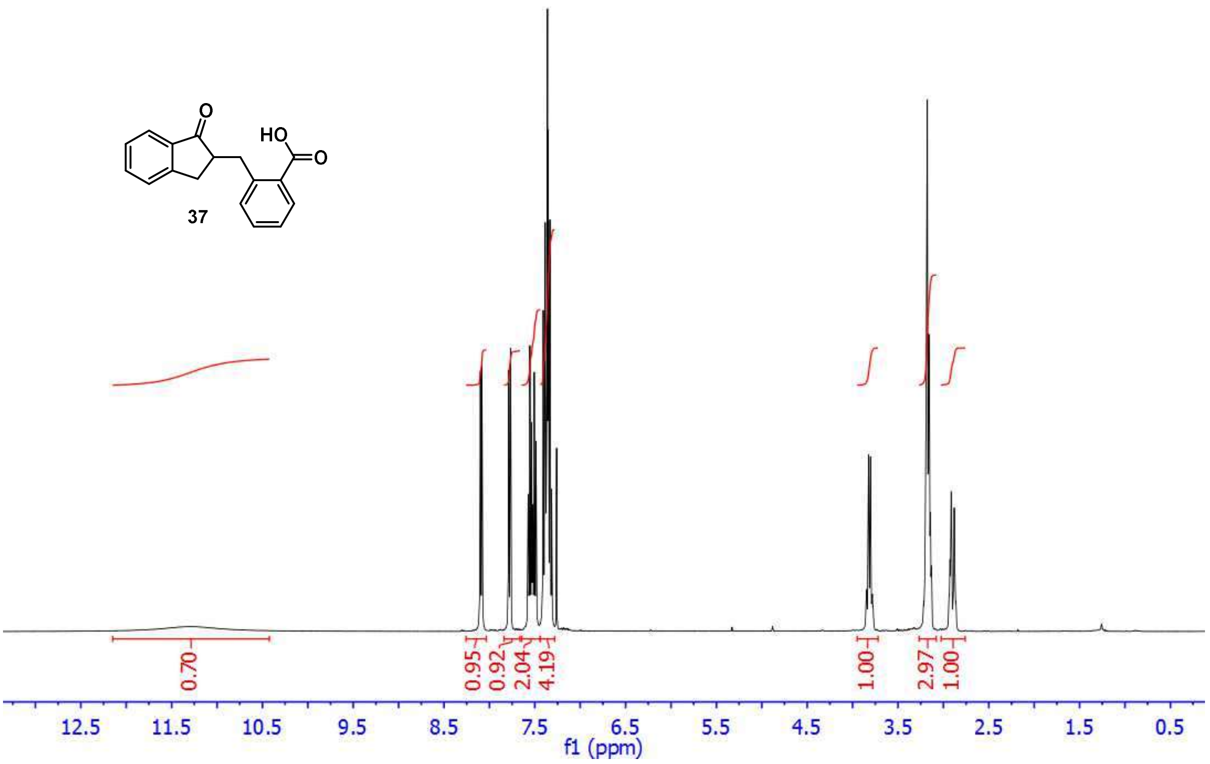


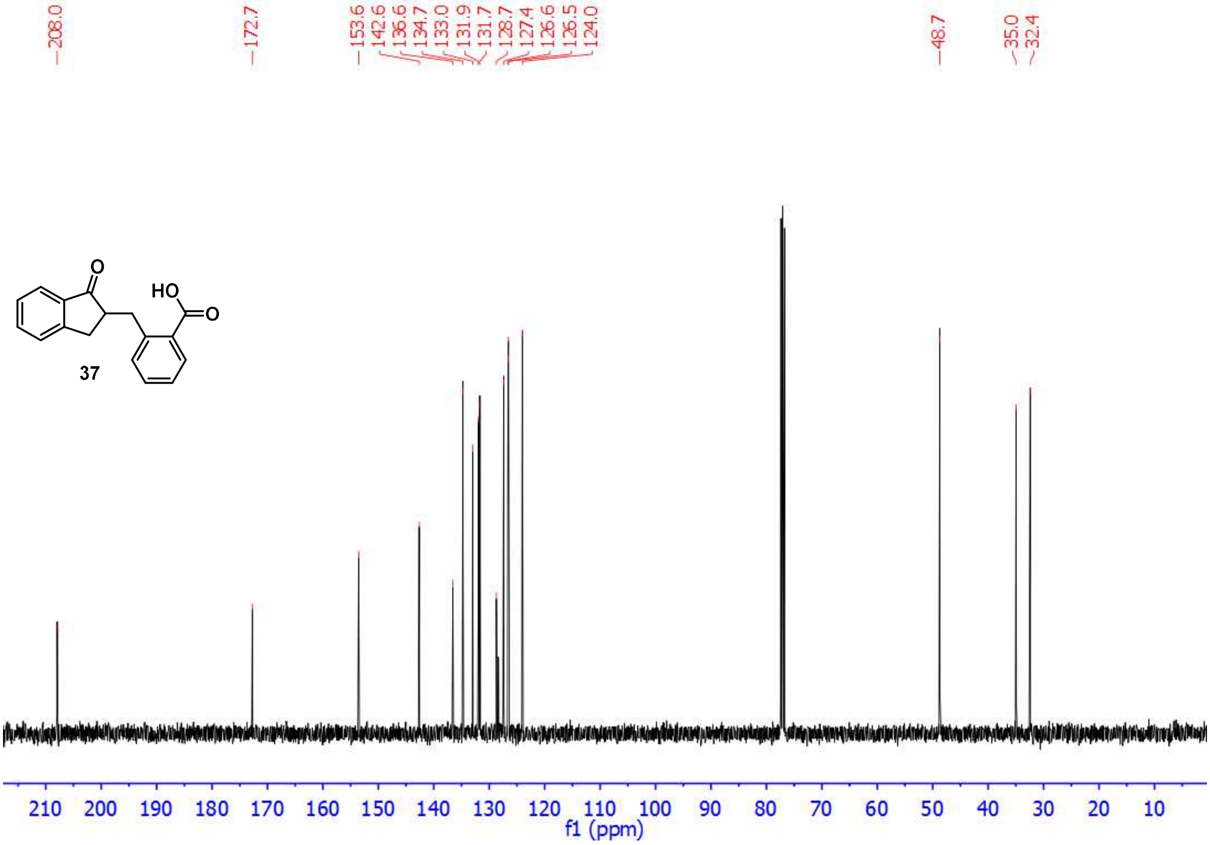

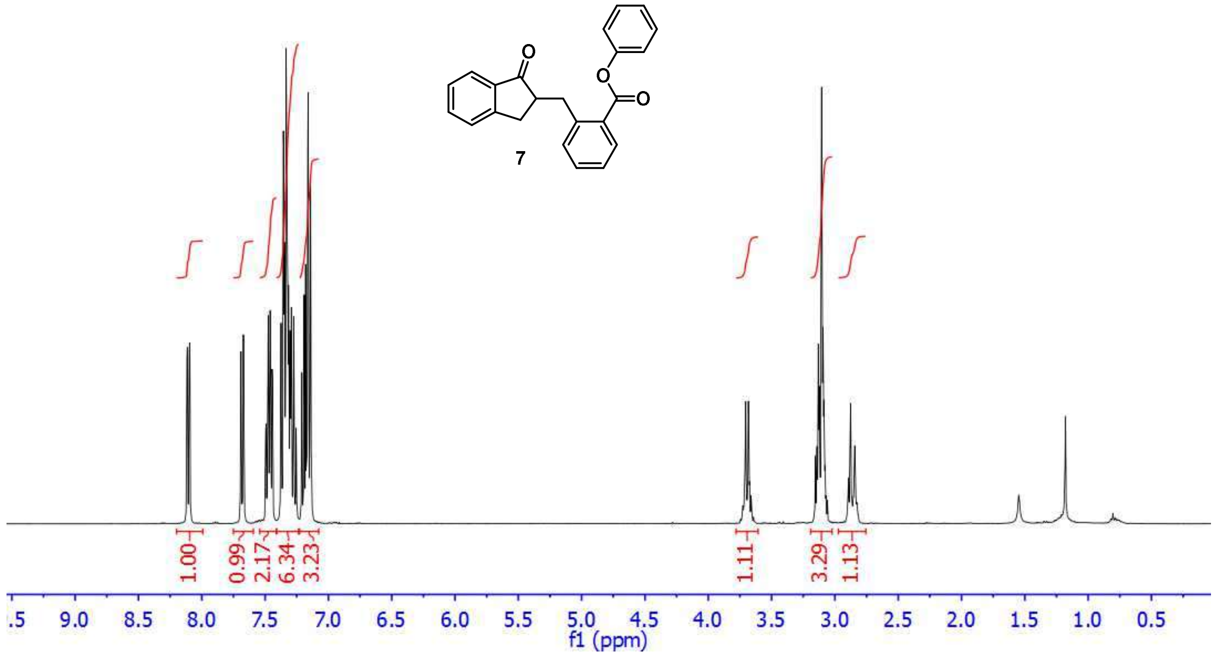
7
1.00
0.99
2.17
6.34
3.23
1.11
3.29
1.13
9.0 8.5 8.0 7.5 7.0 6.5 6.0 5.5 5.0 4.5 4.0 3.5 3.0 2.5 2.0 1.5 1.0 0.5
f1 (ppm)

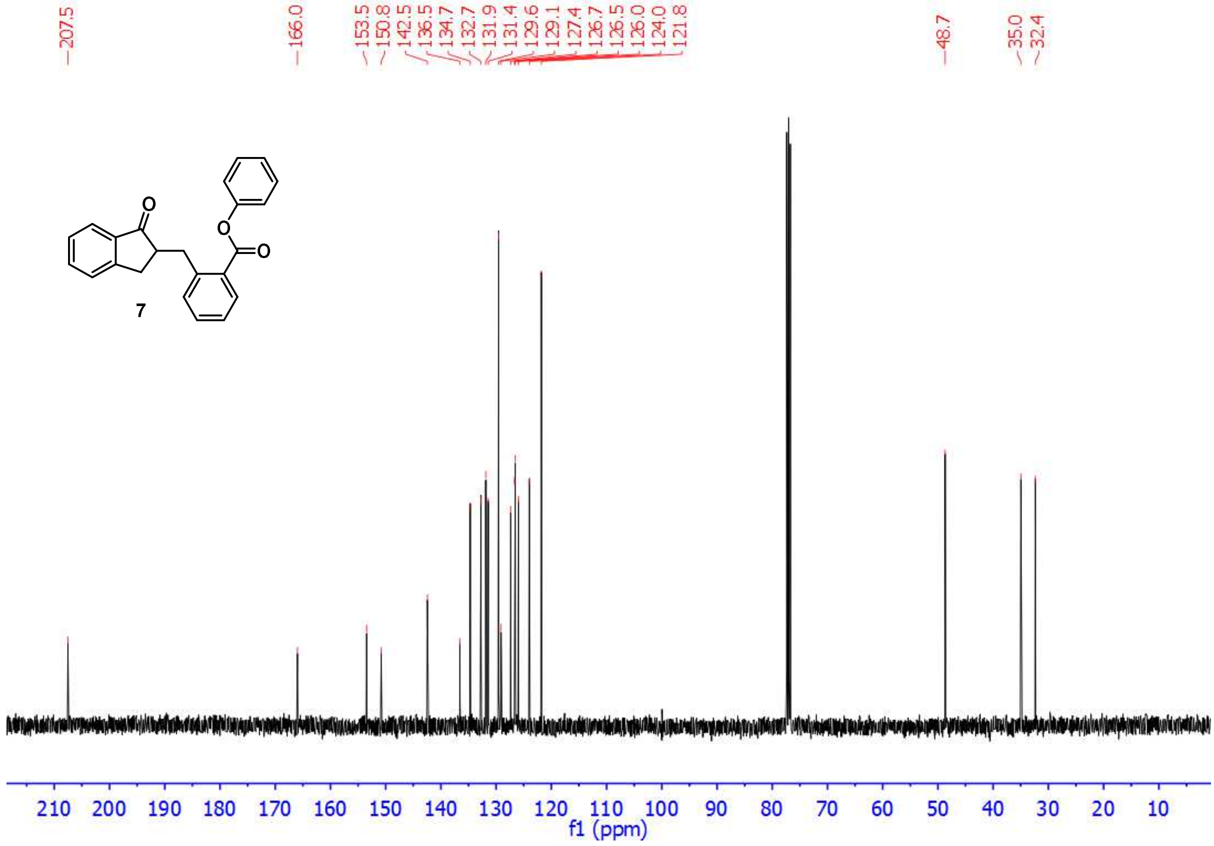
207.5
166.0
153.5
150.8
142.5
136.5
134.7
132.7
131.9
131.4
129.6
129.1
127.4
126.7
126.5
126.0
124.0
121.8
48.7
35.0
32.4
7
210 200 190 180 170 160 150 140 130 120 110 100 90 80 70 60 50 40 30 20 10
f1 (ppm)

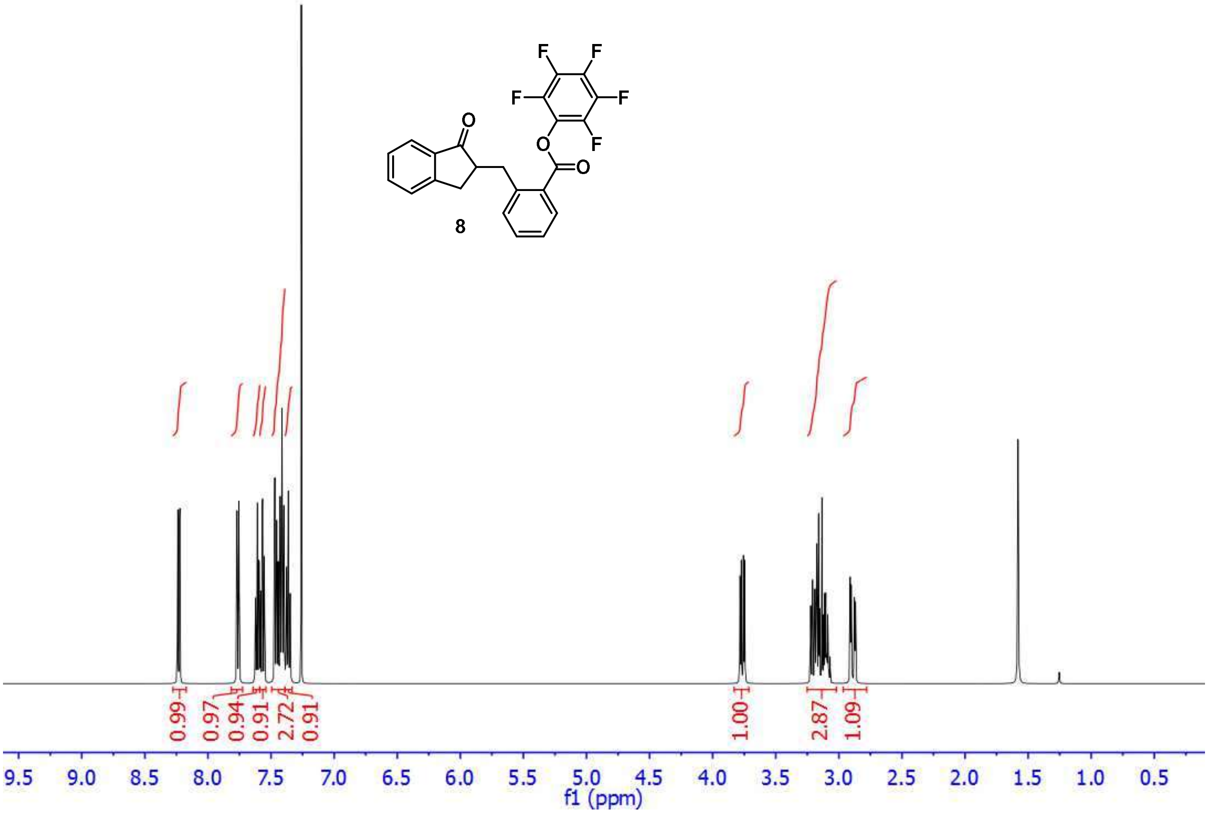
8
0.99
0.97
0.94
0.91
2.72
0.91
1.00
2.87
1.09
9.5 9.0 8.5 8.0 7.5 7.0 6.5 6.0 5.5 5.0 4.5 4.0 3.5 3.0 2.5 2.0 1.5 1.0 0.5
f1 (ppm)

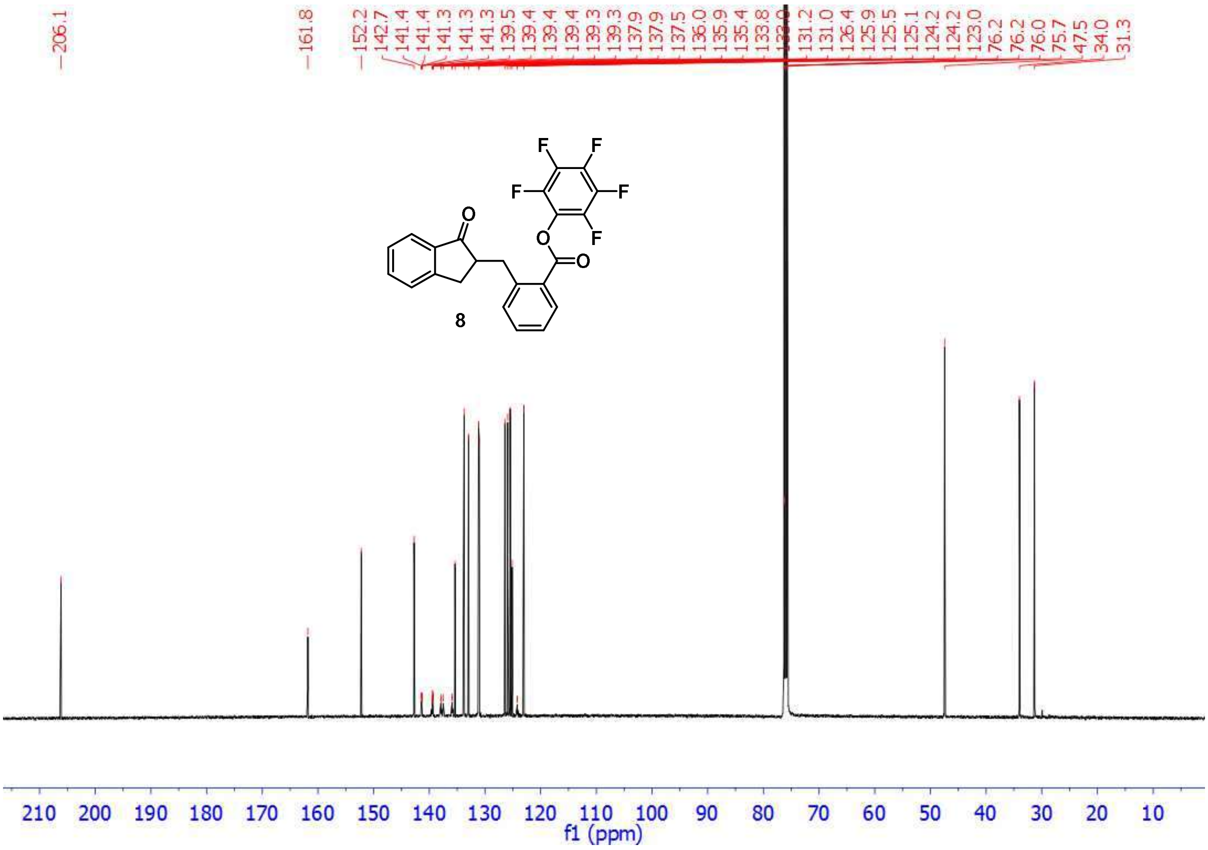
206.1
161.8
152.2
142.7
141.4
141.4
141.3
141.3
141.3
139.5
139.4
139.4
139.4
139.3
139.3
139.3
137.9
137.9
137.5
136.0
135.9
135.4
133.8
[illegible]
131.2
131.0
126.4
125.9
125.5
125.1
124.2
124.2
123.0
76.2
76.0
75.7
47.5
34.0
31.3
8
210 200 190 180 170 160 150 140 130 120 110 100 90 80 70 60 50 40 30 20 10
f1 (ppm)

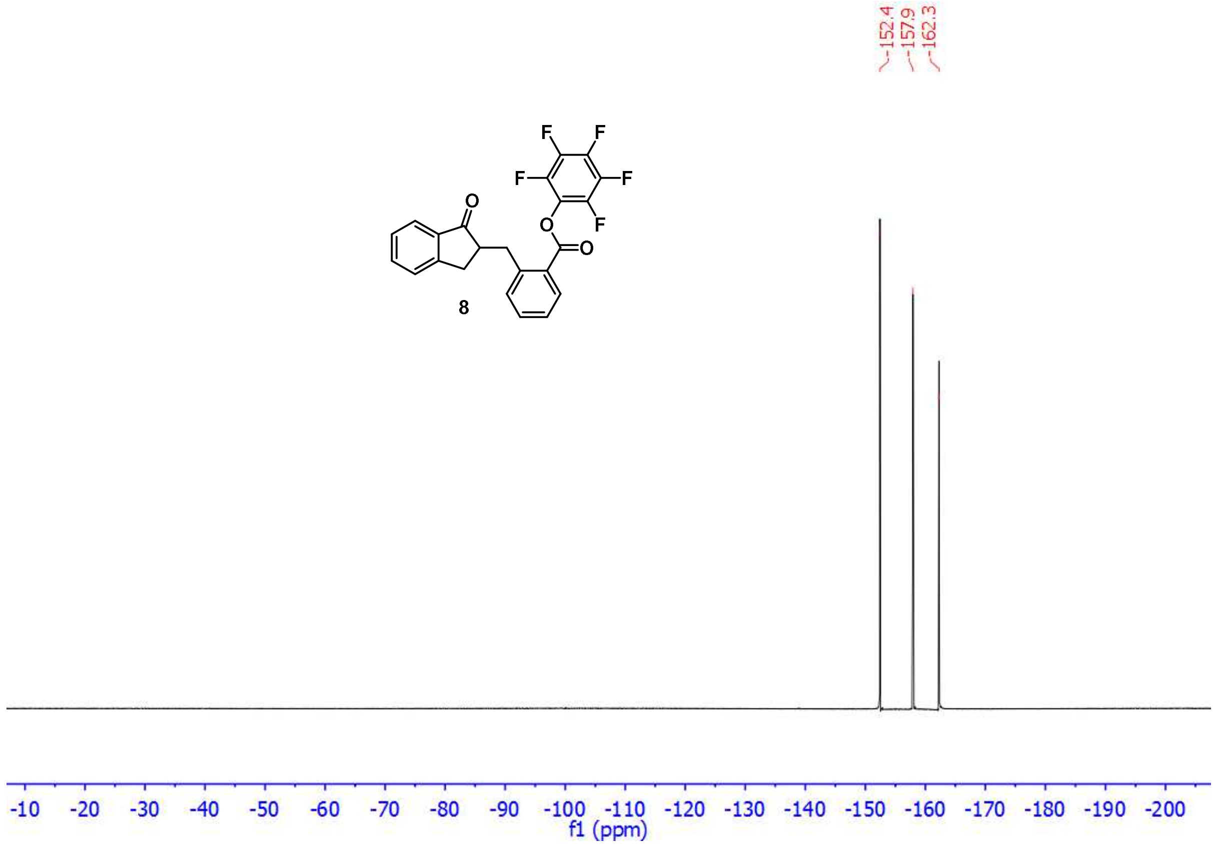
-152.4
-157.9
-162.3
8
-10 -20 -30 -40 -50 -60 -70 -80 -90 -100 -110 -120 -130 -140 -150 -160 -170 -180 -190 -200
f1 (ppm)

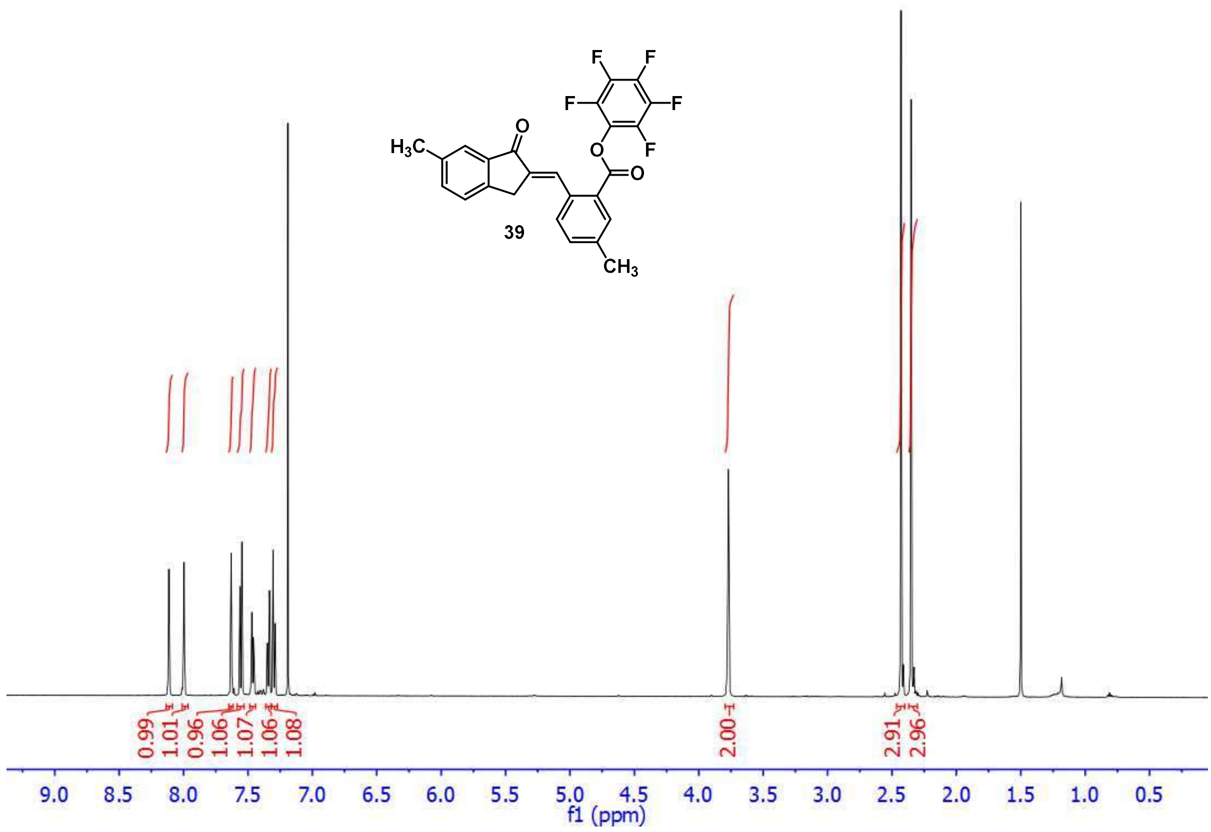
39
0.99
1.01
0.96
1.06
1.07
1.06
1.08
2.00
2.91
2.96
9.0 8.5 8.0 7.5 7.0 6.5 6.0 5.5 5.0 4.5 4.0 3.5 3.0 2.5 2.0 1.5 1.0 0.5
f1 (ppm)

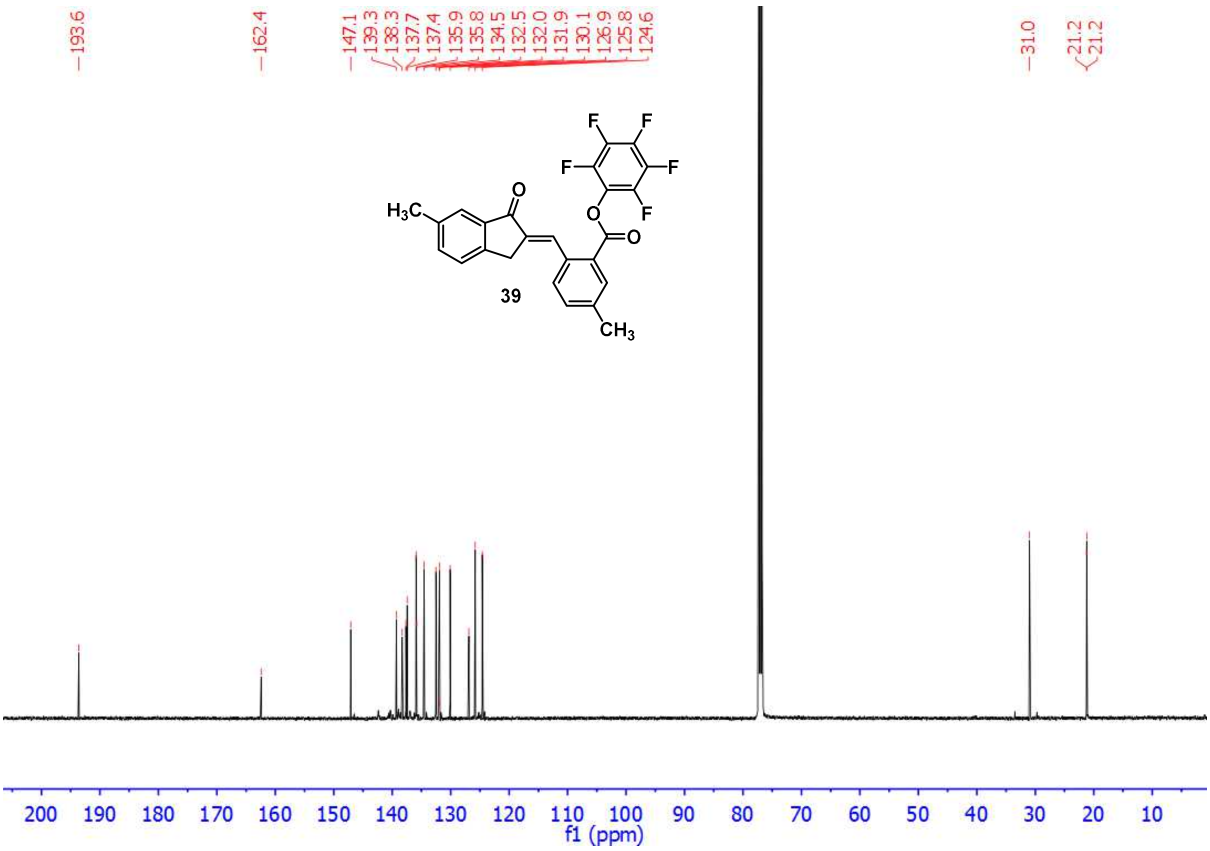
193.6
162.4
147.1
139.3
138.3
137.7
137.4
135.9
135.8
134.5
132.5
132.0
131.9
130.1
126.9
125.8
124.6
31.0
21.2
21.2
39
f1 (ppm)

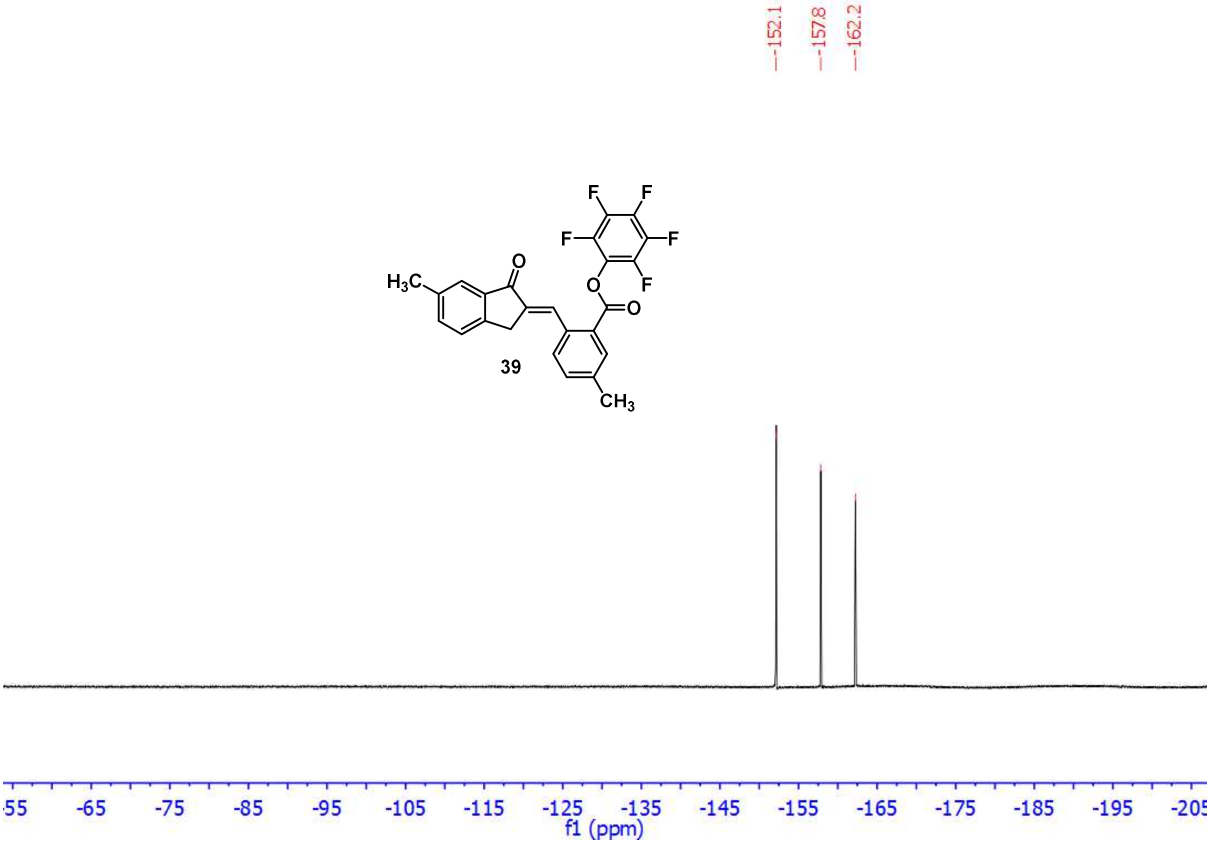
-152.1
-157.8
-162.2
39
f1 (ppm)

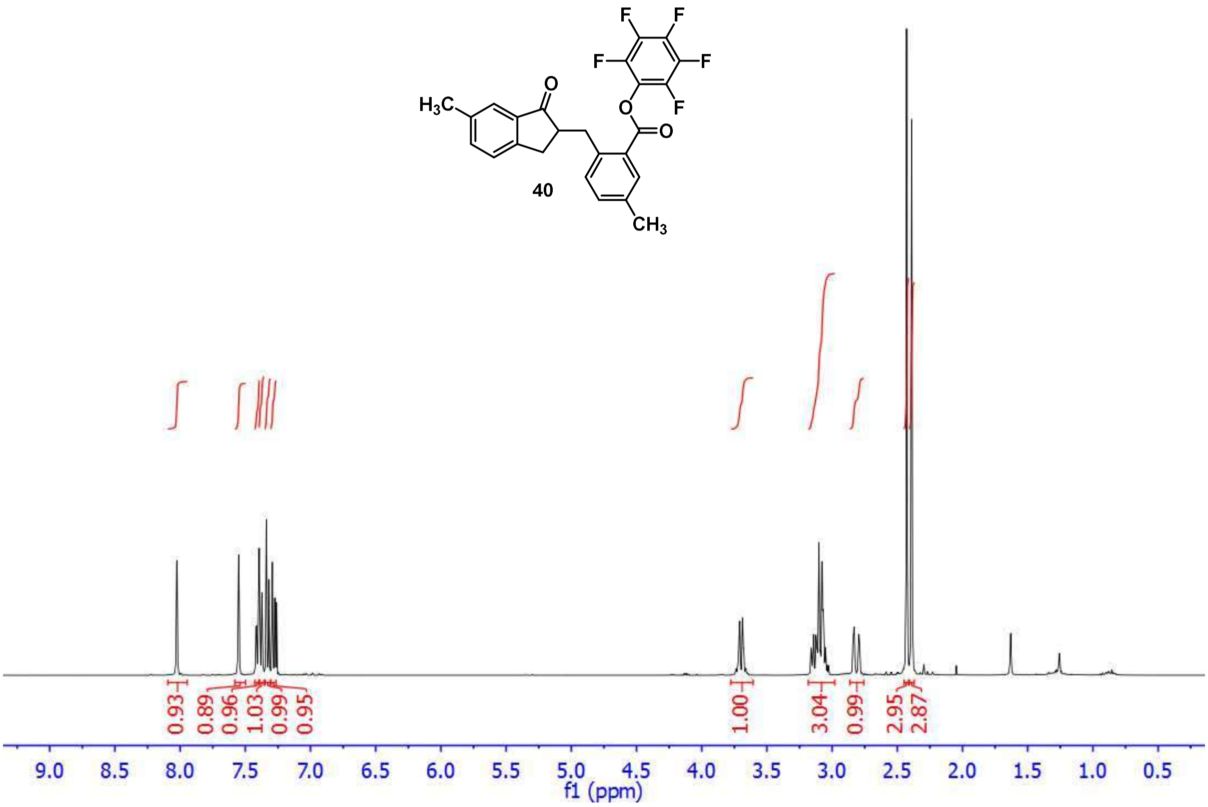

F
F
F
F
F
O
O
O
H3C
CH3
40
0.93
0.89
0.96
1.03
0.99
0.95
1.00
3.04
0.99
2.95
2.87
9.0 8.5 8.0 7.5 7.0 6.5 6.0 5.5 5.0 4.5 4.0 3.5 3.0 2.5 2.0 1.5 1.0 0.5
f1 (ppm)


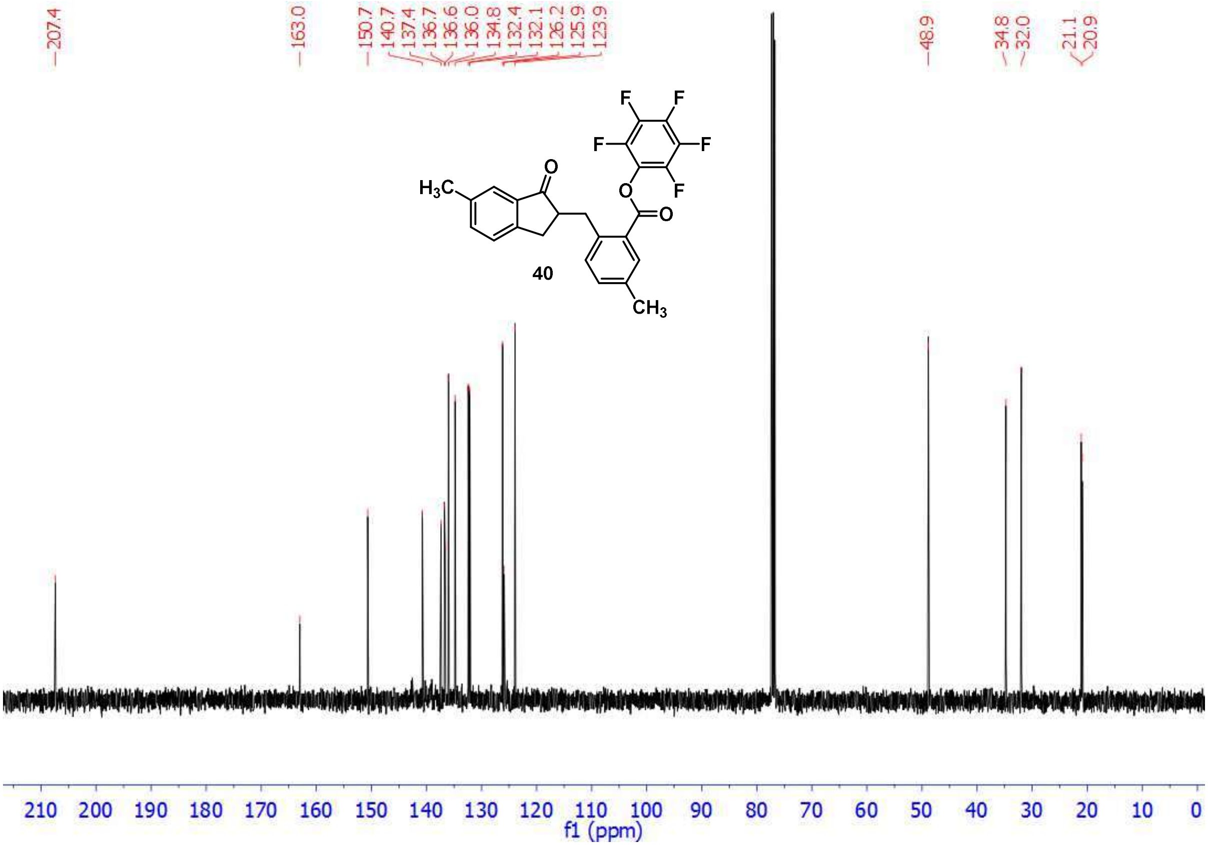

207.4
163.0
150.7
140.7
137.4
136.7
136.6
136.0
134.8
132.4
132.1
126.2
125.9
123.9
48.9
34.8
32.0
21.1
20.9
F
F
F
F
F
O
O
O
H3C
CH3
40
210 200 190 180 170 160 150 140 130 120 110 100 90 80 70 60 50 40 30 20 10 0
f1 (ppm)

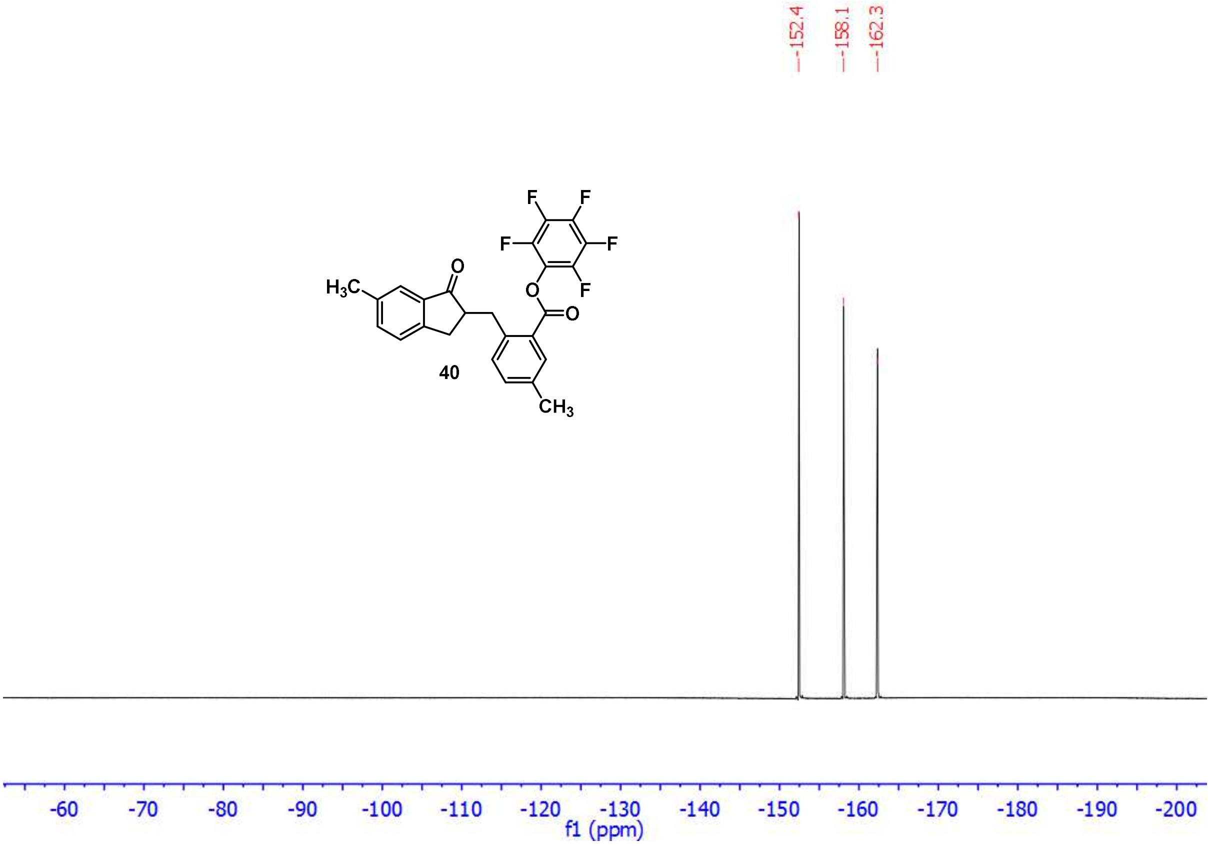
-152.4
-158.1
-162.3
F
F
F
F
F
O
O
O
$H_3C$
40
$CH_3$
-60 -70 -80 -90 -100 -110 -120 -130 -140 -150 -160 -170 -180 -190 -200
f1 (ppm)

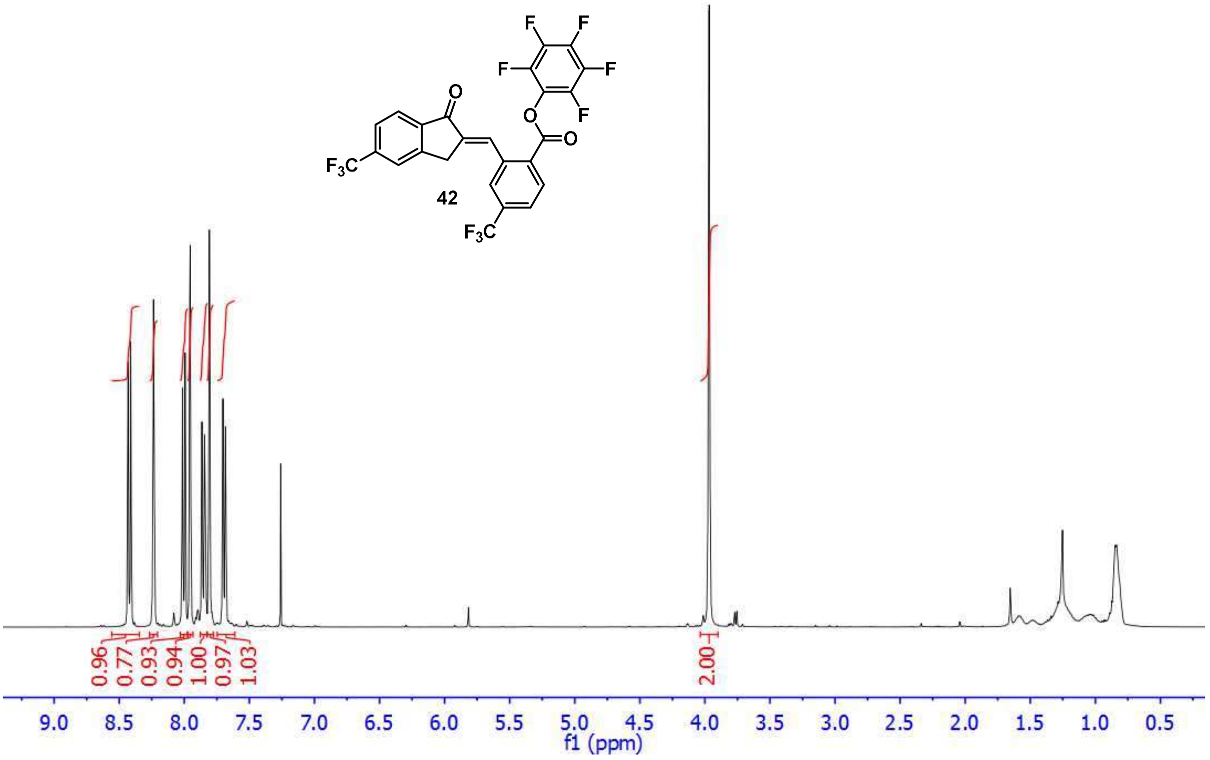
F
F
F
F
F
O
O
O
$F_3C$
42
$F_3C$
0.96
0.77
0.93
0.94
1.00
0.97
1.03
2.00
9.0 8.5 8.0 7.5 7.0 6.5 6.0 5.5 5.0 4.5 4.0 3.5 3.0 2.5 2.0 1.5 1.0 0.5
f1 (ppm)

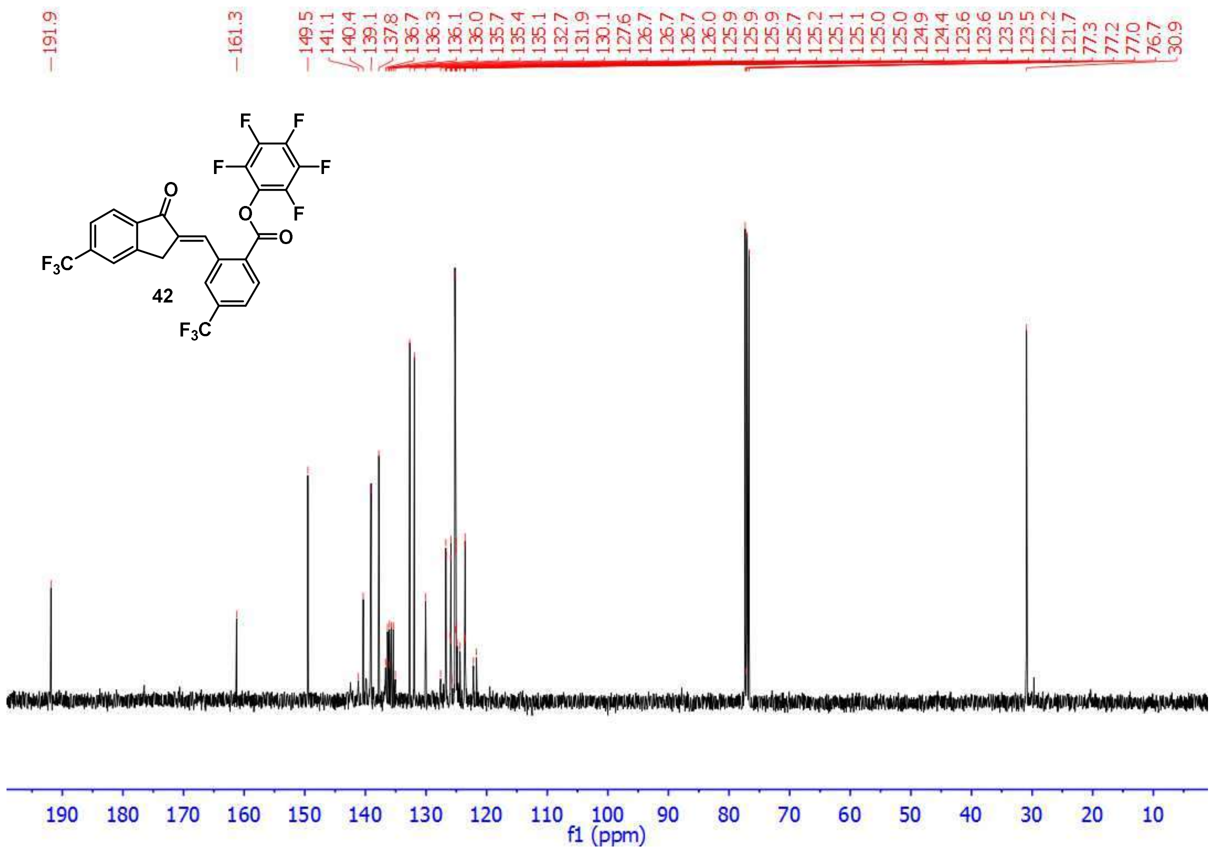
191.9
161.3
149.5
141.1
140.4
139.1
137.8
136.7
136.3
136.1
136.0
135.7
135.4
135.1
132.7
131.9
130.1
127.6
126.7
126.7
126.7
126.7
126.0
125.9
125.9
125.9
125.7
125.2
125.1
125.1
125.0
125.0
124.9
124.4
123.6
123.6
123.5
123.5
122.2
121.7
77.3
77.2
77.0
76.7
30.9
42
f1 (ppm)

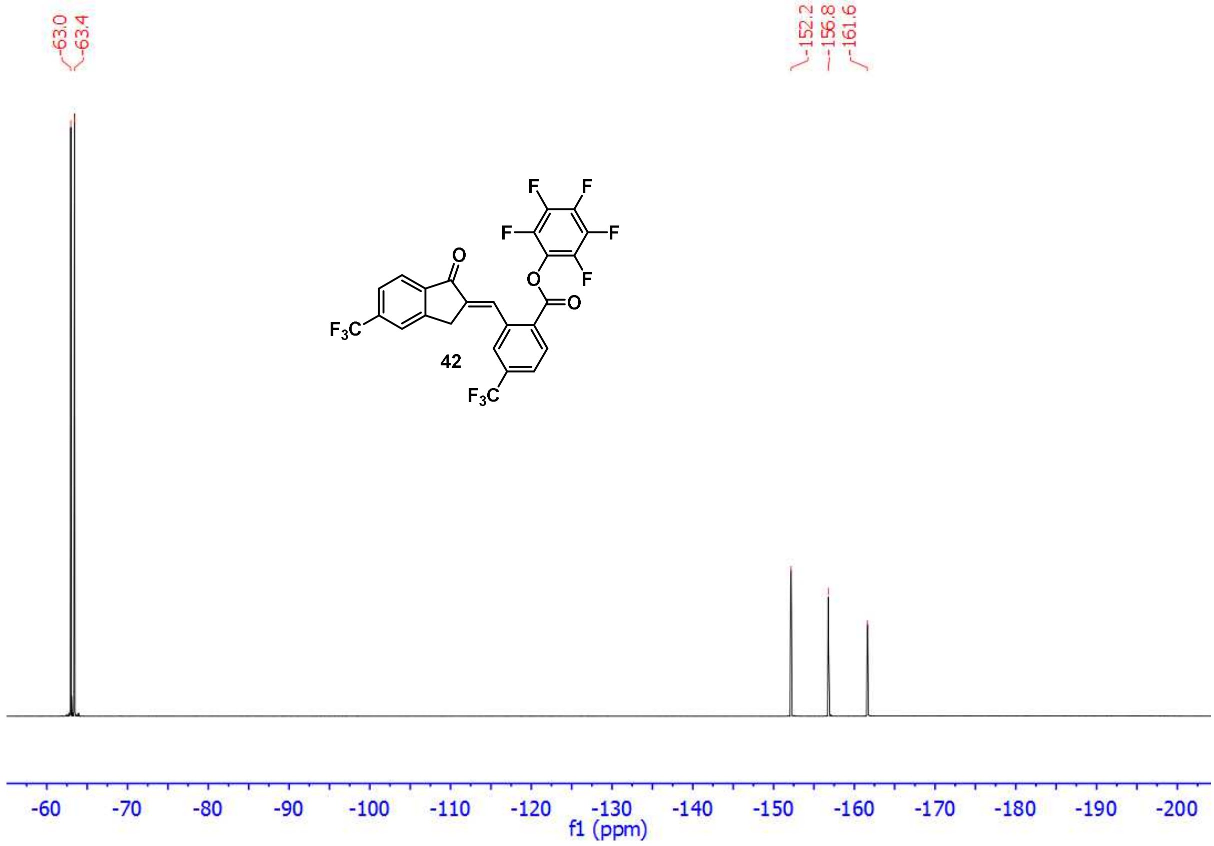
-63.0
-63.4
-152.2
-156.8
-161.6
42
f1 (ppm)

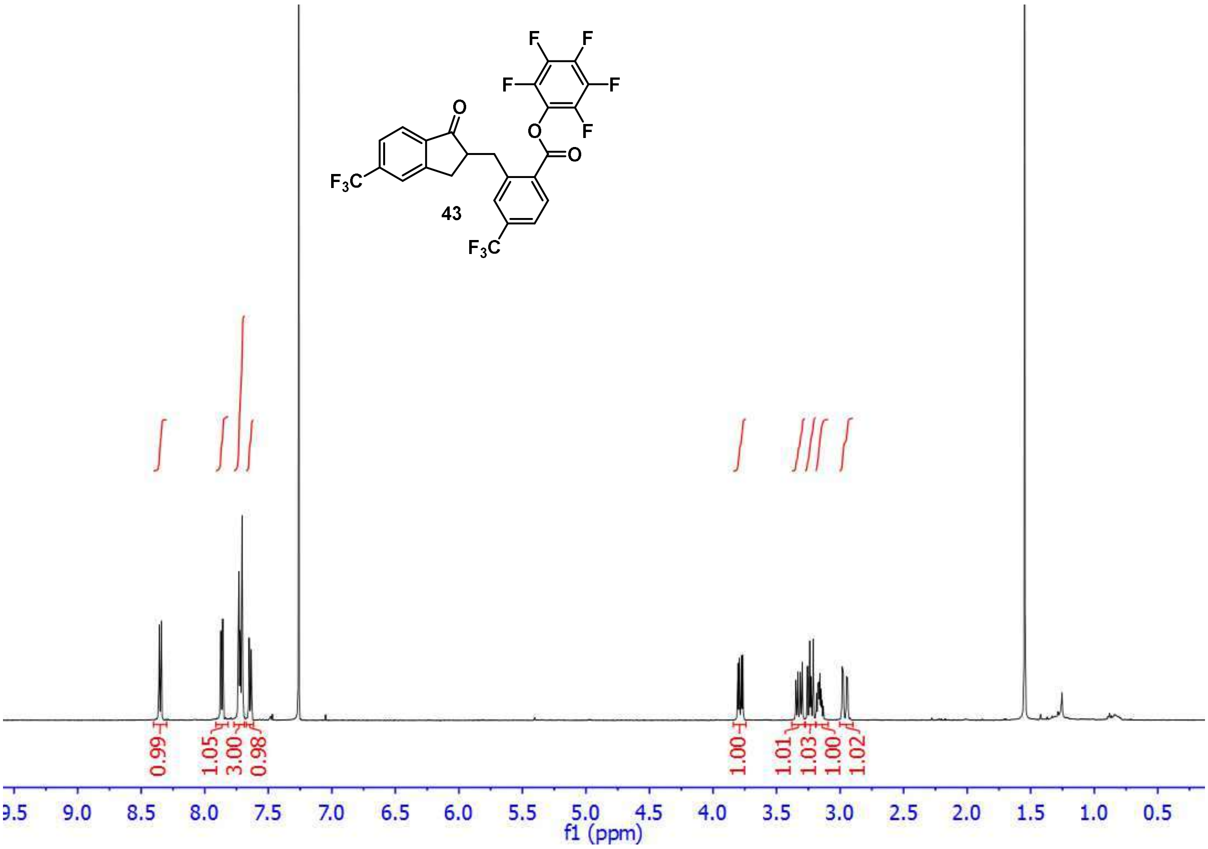
43
0.99
1.05
3.00
0.98
1.00
1.01
1.03
1.00
1.02
f1 (ppm)

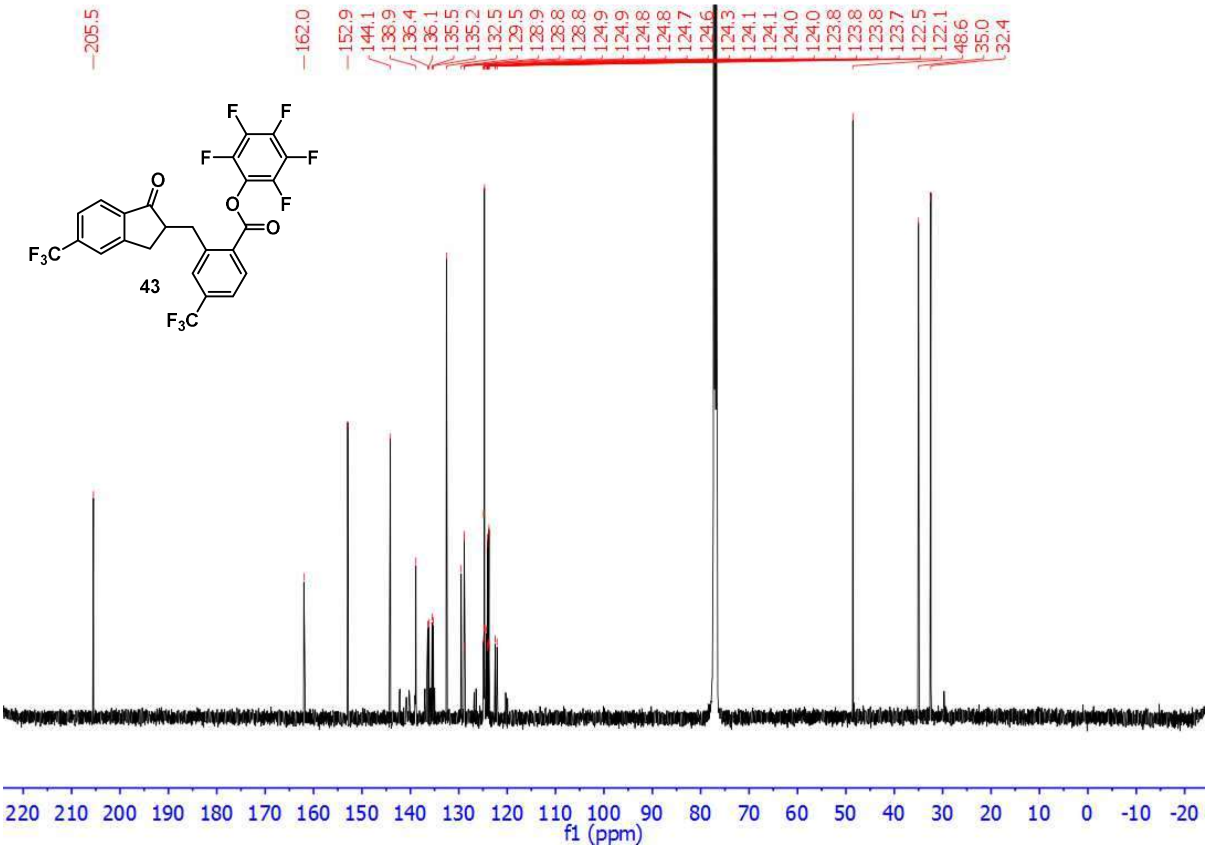
205.5
162.0
152.9
144.1
138.9
136.4
136.1
135.5
135.2
132.5
129.5
128.9
128.8
128.8
124.9
124.9
124.8
124.8
124.7
124.6
124.3
124.1
124.1
124.0
124.0
123.8
123.8
123.7
122.5
122.1
48.6
35.0
32.4
43
f1 (ppm)

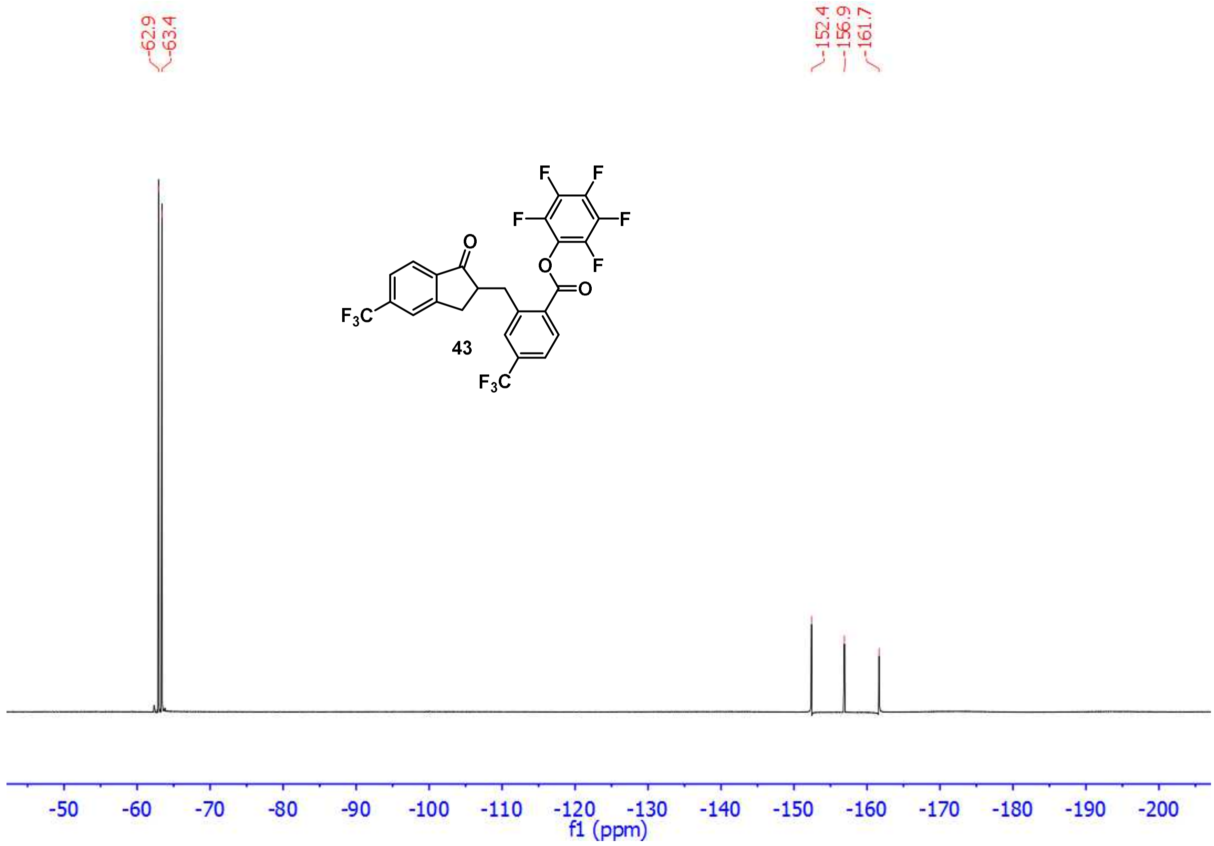
-62.9
-63.4
-152.4
-156.9
-161.7
F
F
F
F
F
O
O
O
F3C
43
F3C
-50 -60 -70 -80 -90 -100 -110 -120 -130 -140 -150 -160 -170 -180 -190 -200
f1 (ppm)

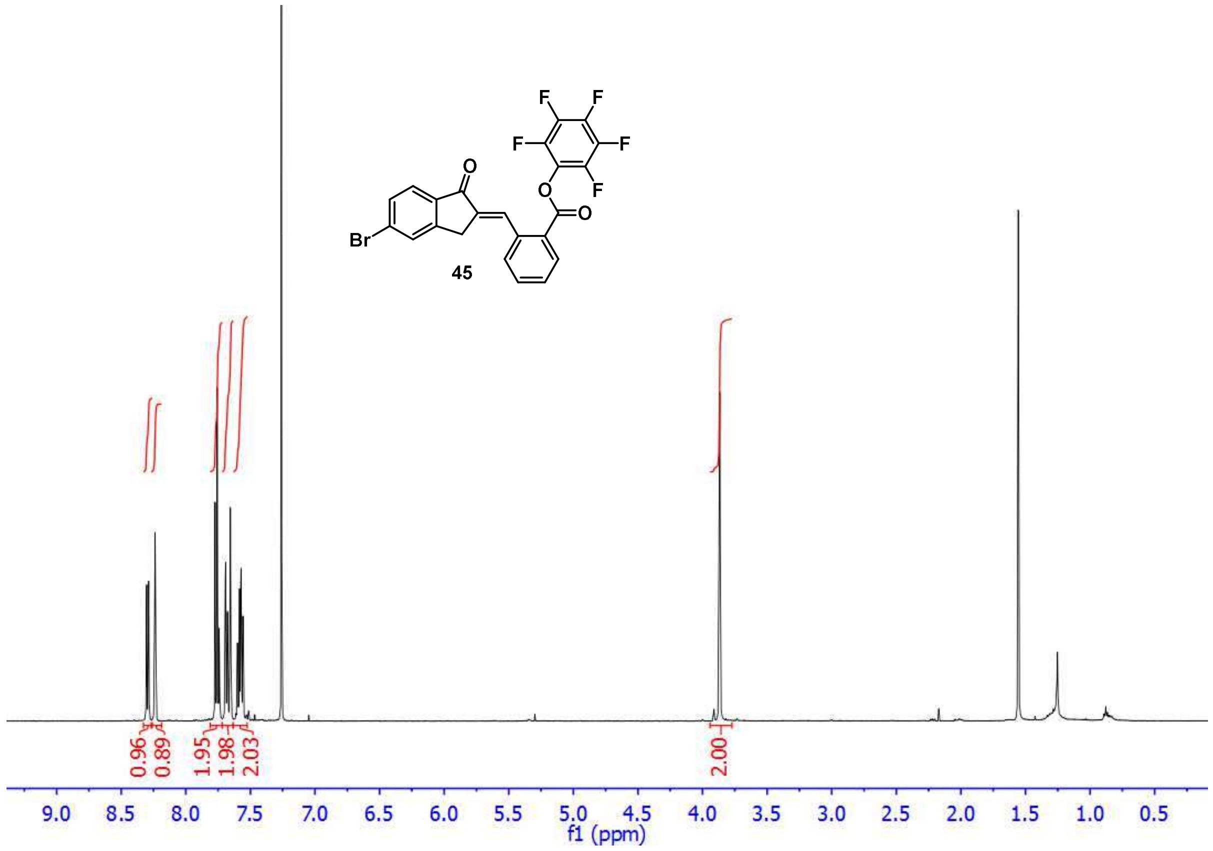
F
F
F
F
F
O
O
O
Br
45
0.96
0.89
1.95
1.98
2.03
2.00
9.0 8.5 8.0 7.5 7.0 6.5 6.0 5.5 5.0 4.5 4.0 3.5 3.0 2.5 2.0 1.5 1.0 0.5
f1 (ppm)

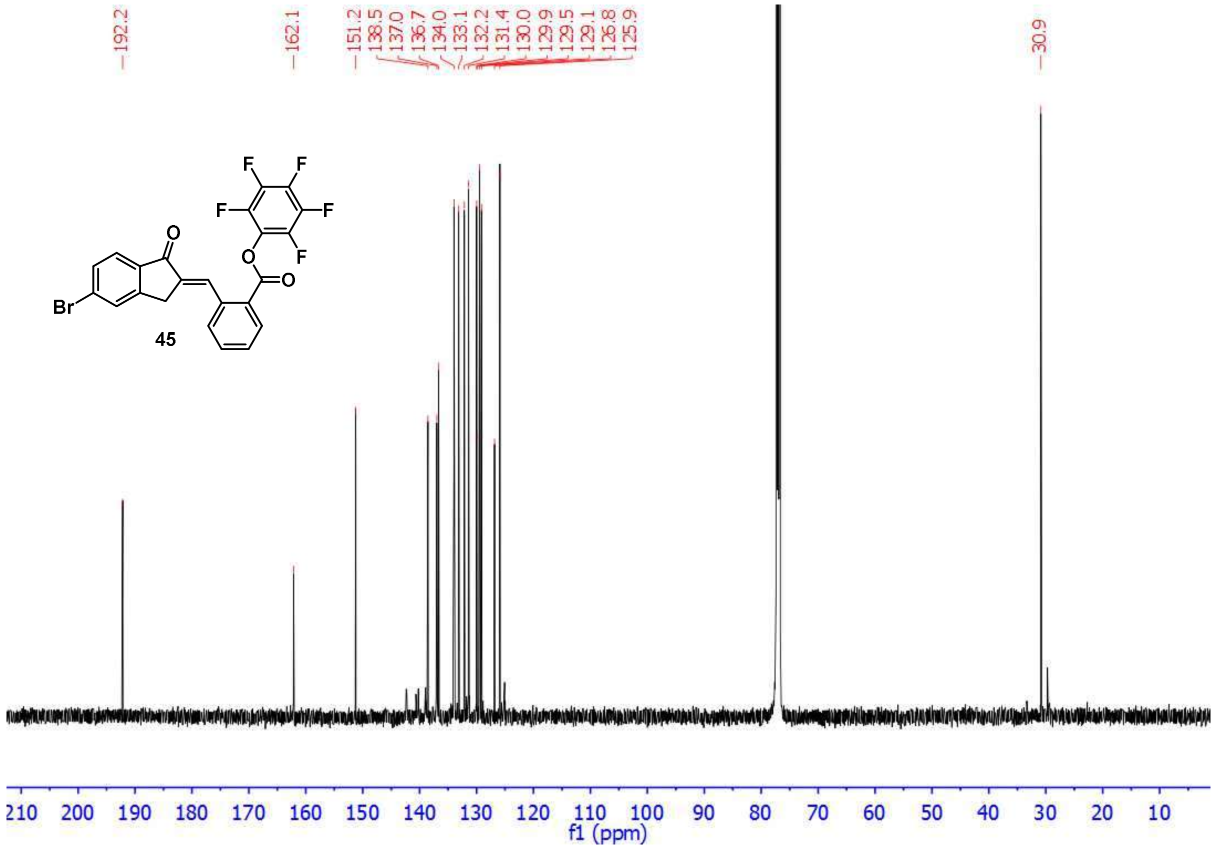

192.2
162.1
151.2
138.5
137.0
136.7
134.0
133.1
132.2
131.4
130.0
129.9
129.5
129.1
126.8
125.9
30.9
Br
O
F
45
210 200 190 180 170 160 150 140 130 120 110 100 90 80 70 60 50 40 30 20 10
f1 (ppm)


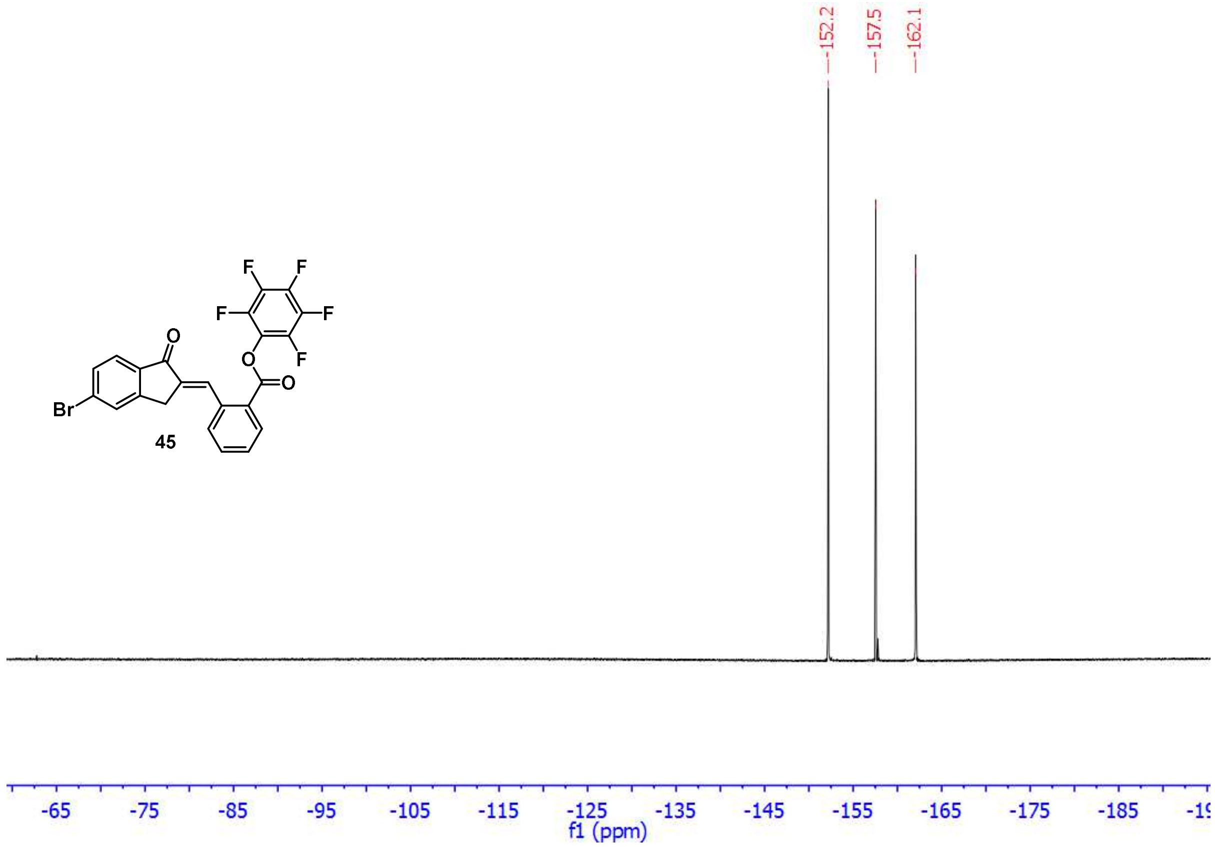

-152.2
-157.5
-162.1
Br
O
F
45
-65 -75 -85 -95 -105 -115 -125 -135 -145 -155 -165 -175 -185
f1 (ppm)

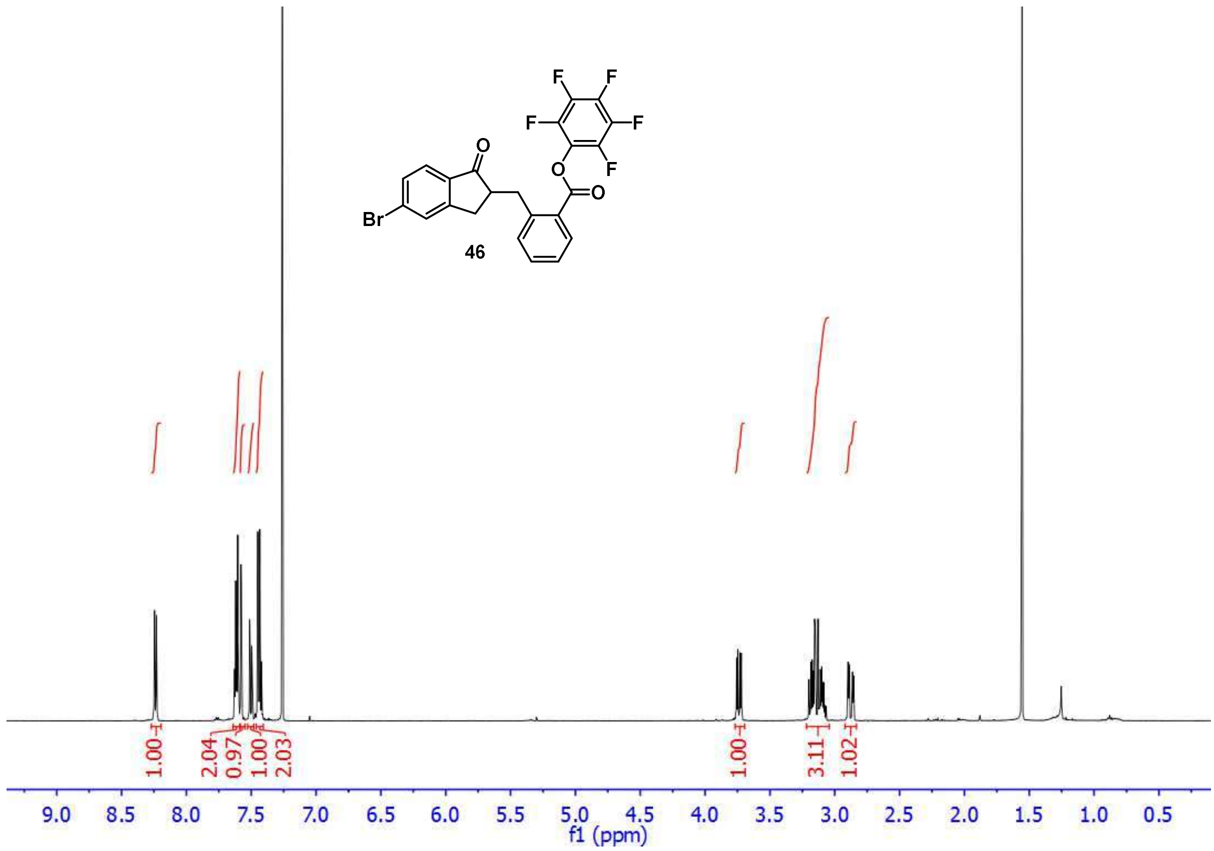
F
F
F
F
F
O
O
O
Br
46
1.00
2.04
0.97
1.00
2.03
1.00
3.11
1.02
9.0 8.5 8.0 7.5 7.0 6.5 6.0 5.5 5.0 4.5 4.0 3.5 3.0 2.5 2.0 1.5 1.0 0.5
f1 (ppm)

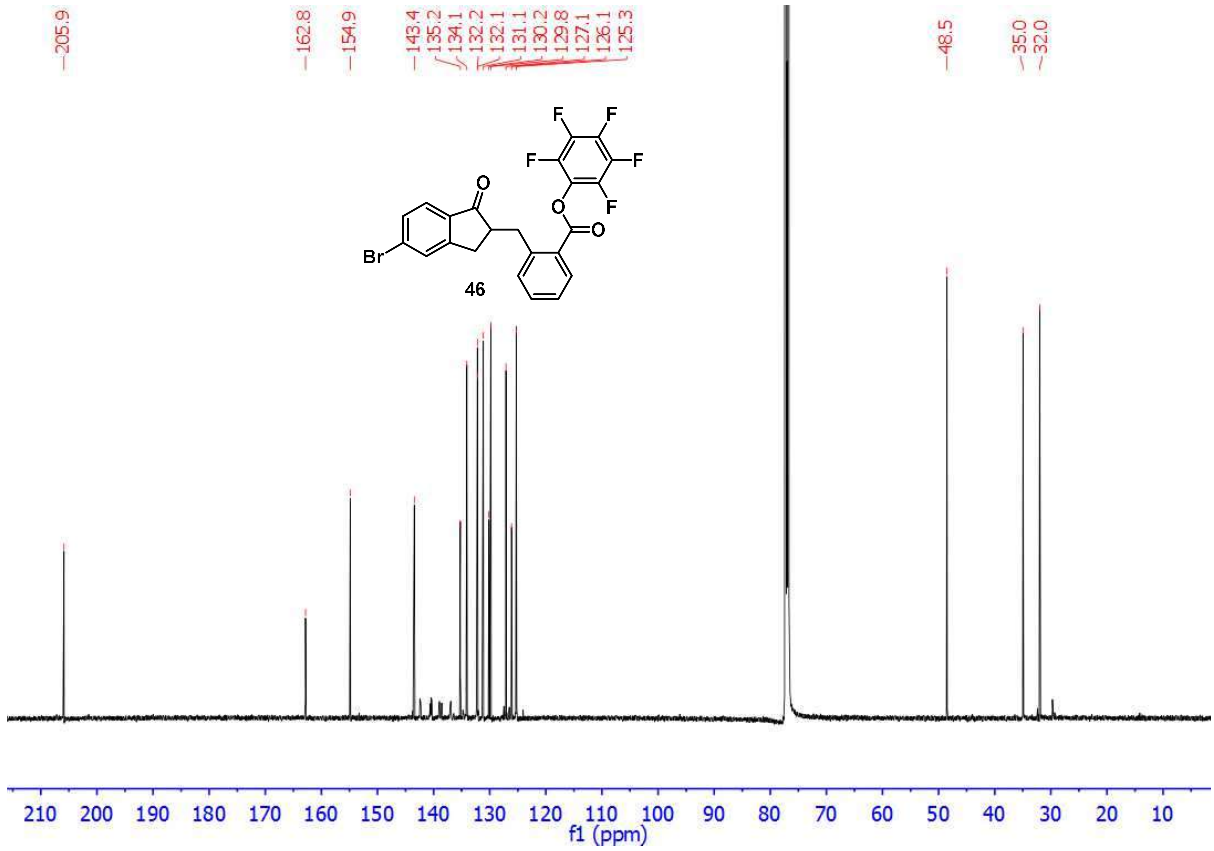
205.9
162.8
154.9
143.4
135.2
134.1
132.2
132.1
131.1
130.2
129.8
127.1
126.1
125.3
48.5
35.0
32.0
F
F
F
F
F
O
O
O
Br
46
210 200 190 180 170 160 150 140 130 120 110 100 90 80 70 60 50 40 30 20 10
f1 (ppm)

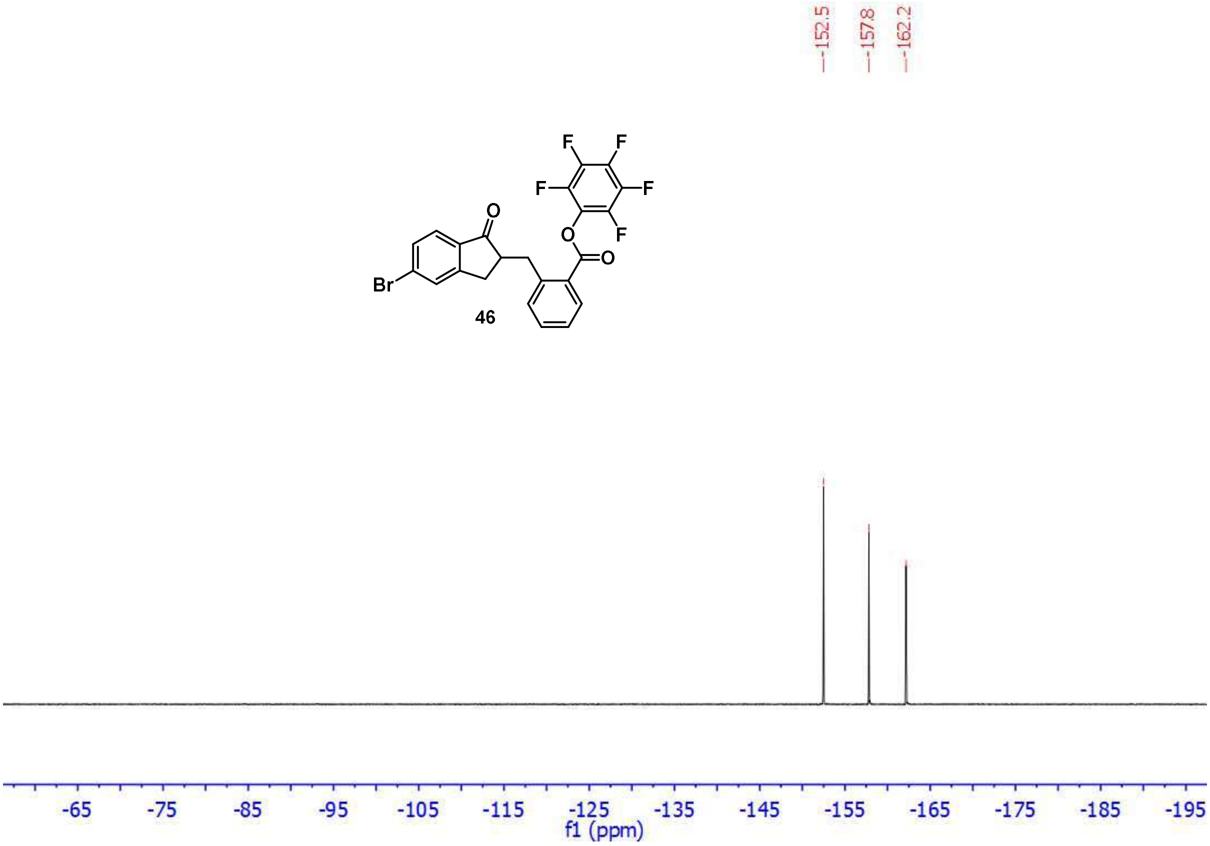
-152.5
-157.8
-162.2
46
-65
-75
-85
-95
-105
-115
-125
-135
-145
-155
-165
-175
-185
-195
f1 (ppm)

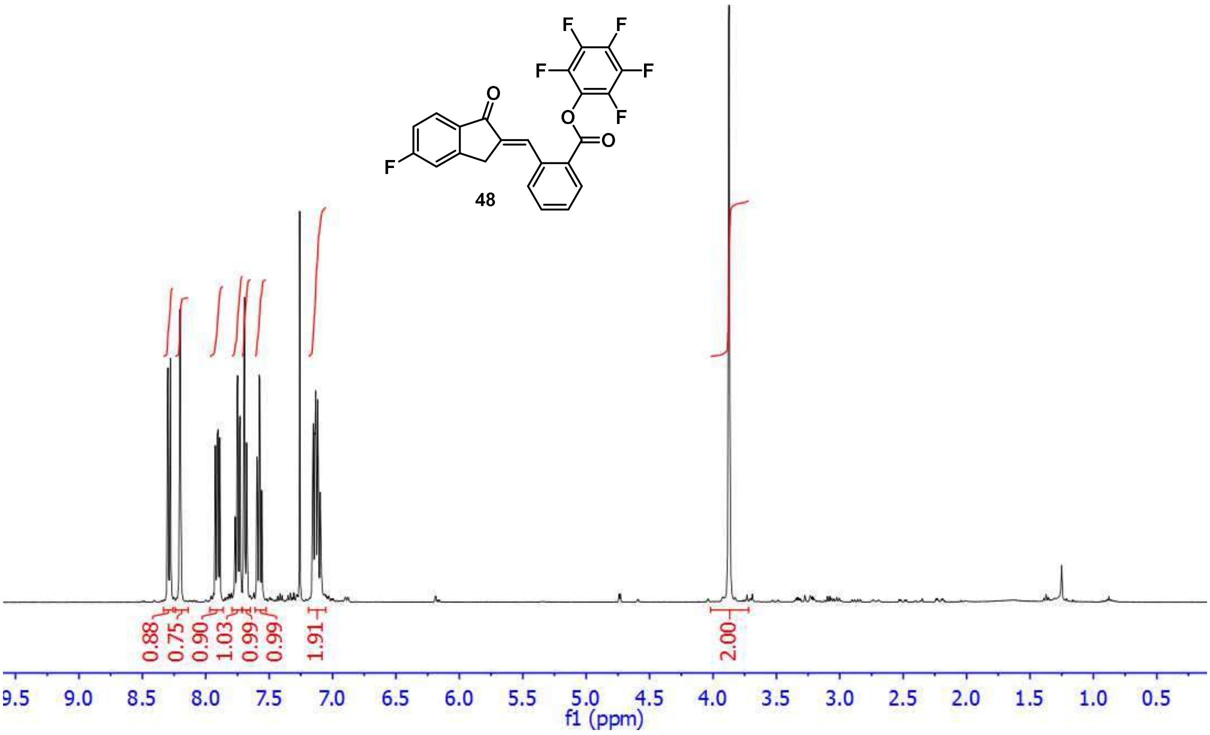
48
0.88
0.75
0.90
1.03
0.99
0.99
1.91
2.00
9.5
9.0
8.5
8.0
7.5
7.0
6.5
6.0
5.5
5.0
4.5
4.0
3.5
3.0
2.5
2.0
1.5
1.0
0.5
f1 (ppm)

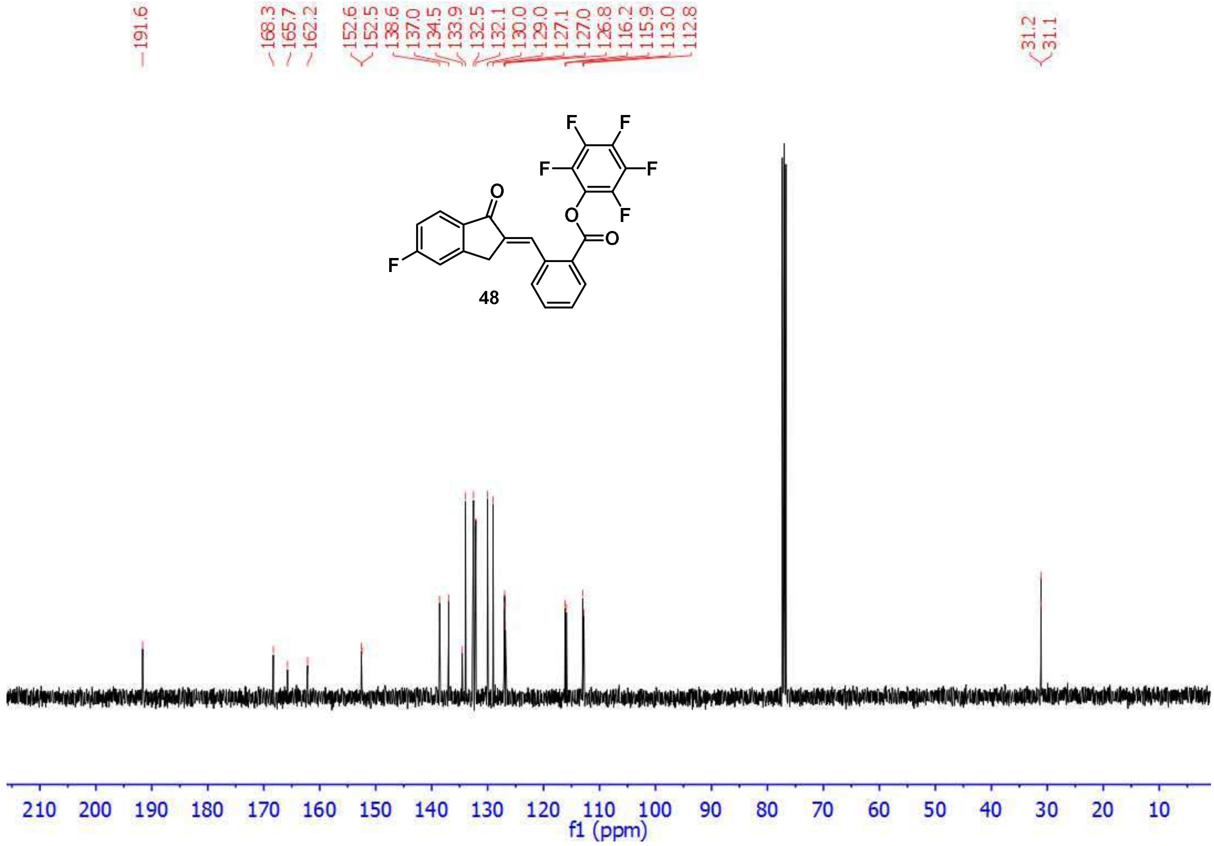
191.6
168.3
165.7
162.2
152.6
152.5
138.6
137.0
134.5
133.9
132.5
132.1
130.0
129.0
127.1
127.0
126.8
116.2
115.9
113.0
112.8
31.2
31.1
48
210 200 190 180 170 160 150 140 130 120 110 100 90 80 70 60 50 40 30 20 10
f1 (ppm)

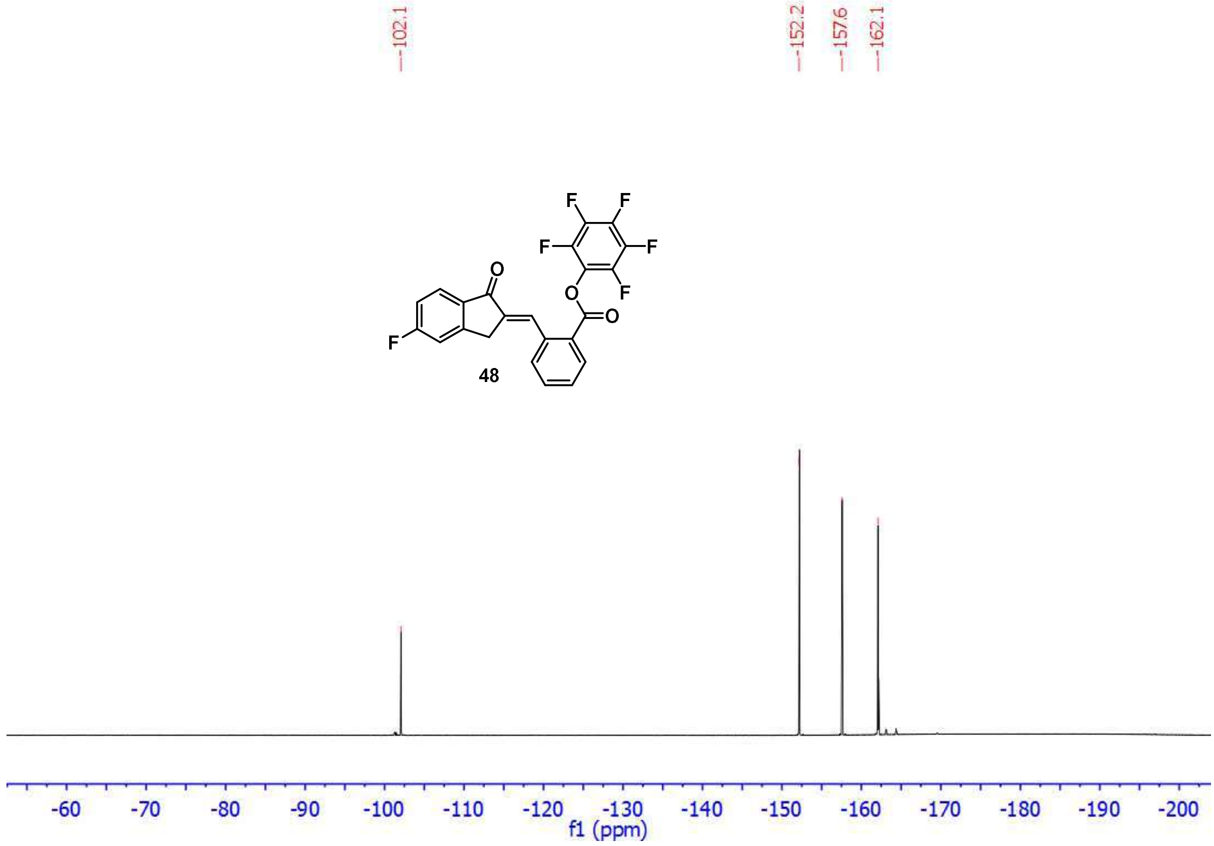
-102.1
-152.2
-157.6
-162.1
48
-60 -70 -80 -90 -100 -110 -120 -130 -140 -150 -160 -170 -180 -190 -200
f1 (ppm)

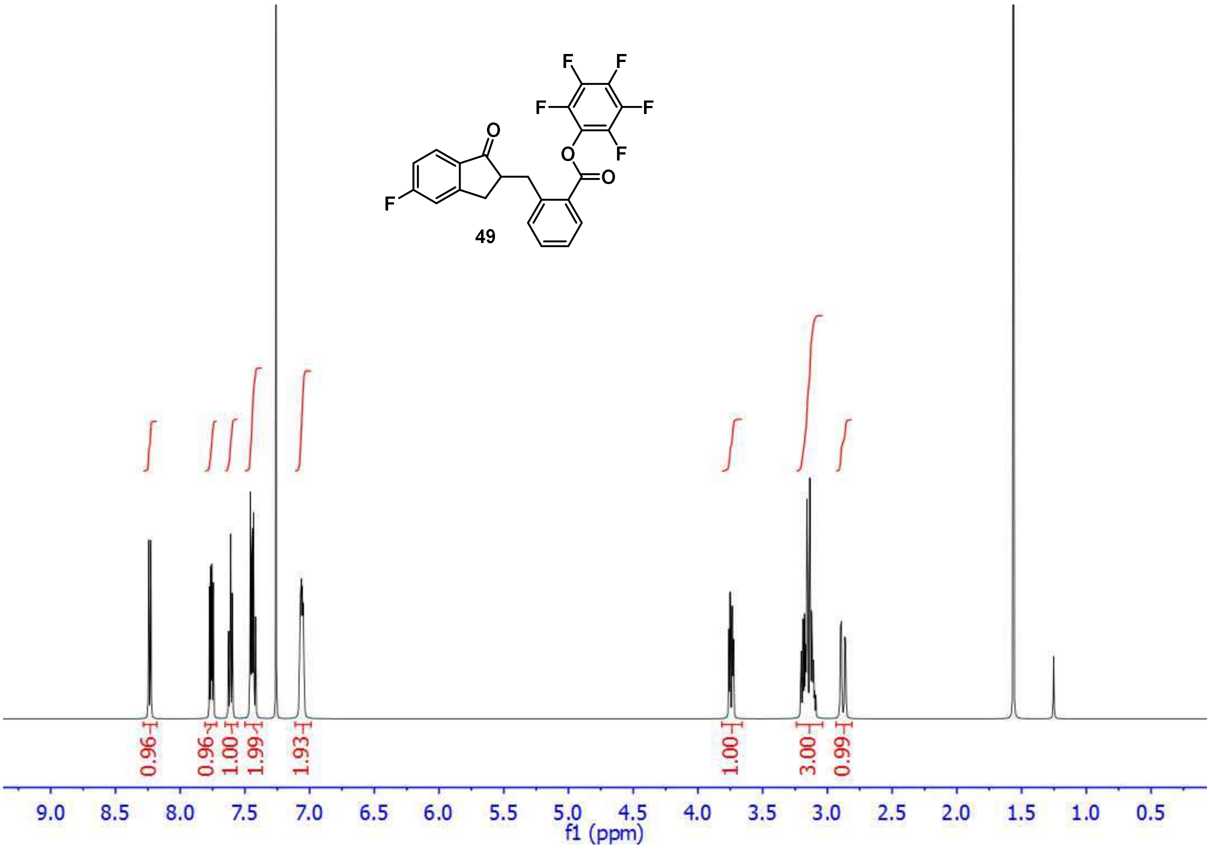
F
F
F
F
F
O
O
O
F
49
0.96
0.96
1.00
1.99
1.93
1.00
3.00
0.99
9.0 8.5 8.0 7.5 7.0 6.5 6.0 5.5 5.0 4.5 4.0 3.5 3.0 2.5 2.0 1.5 1.0 0.5
f1 (ppm)

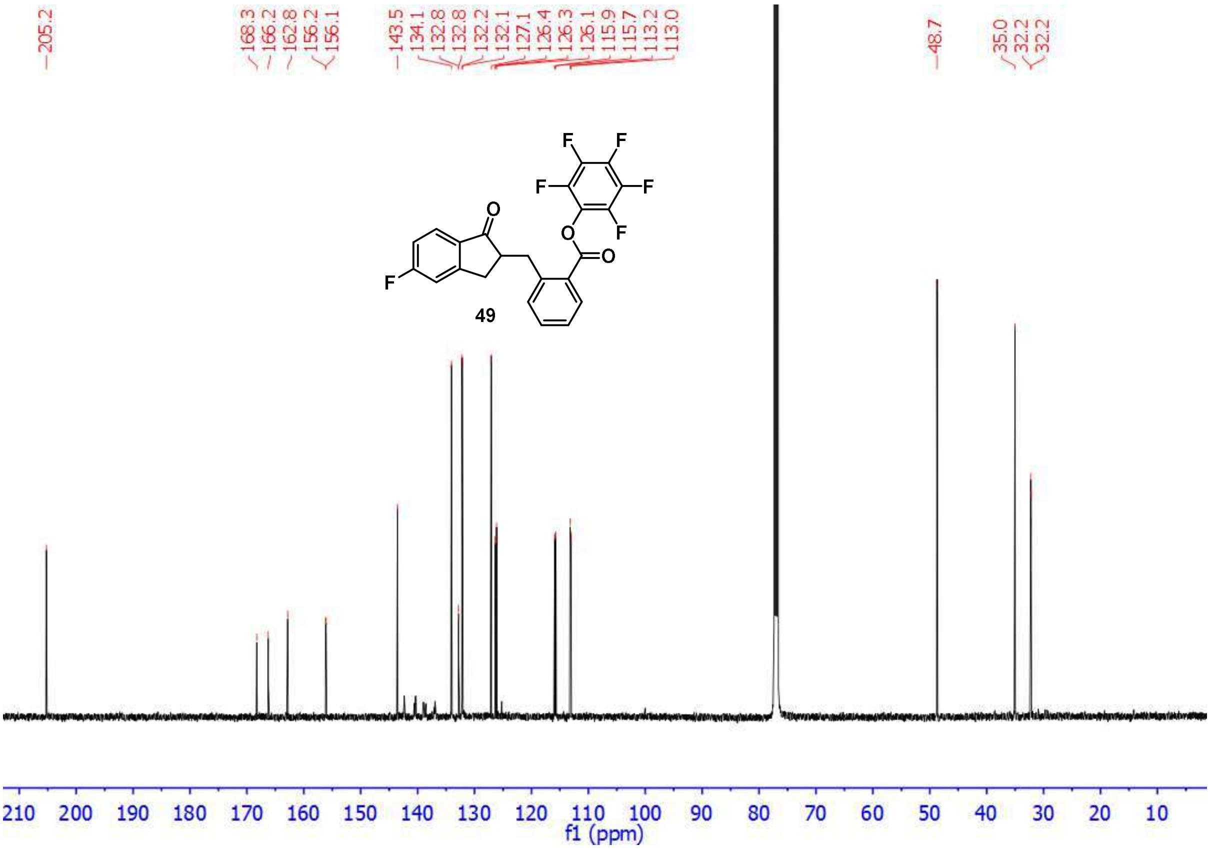
205.2
168.3
166.2
162.8
156.2
156.1
143.5
134.1
132.8
132.8
132.2
132.1
127.1
126.4
126.3
126.1
115.9
115.7
113.2
113.0
48.7
35.0
32.2
32.2
F
F
F
F
F
O
O
O
F
49
210 200 190 180 170 160 150 140 130 120 110 100 90 80 70 60 50 40 30 20 10
f1 (ppm)

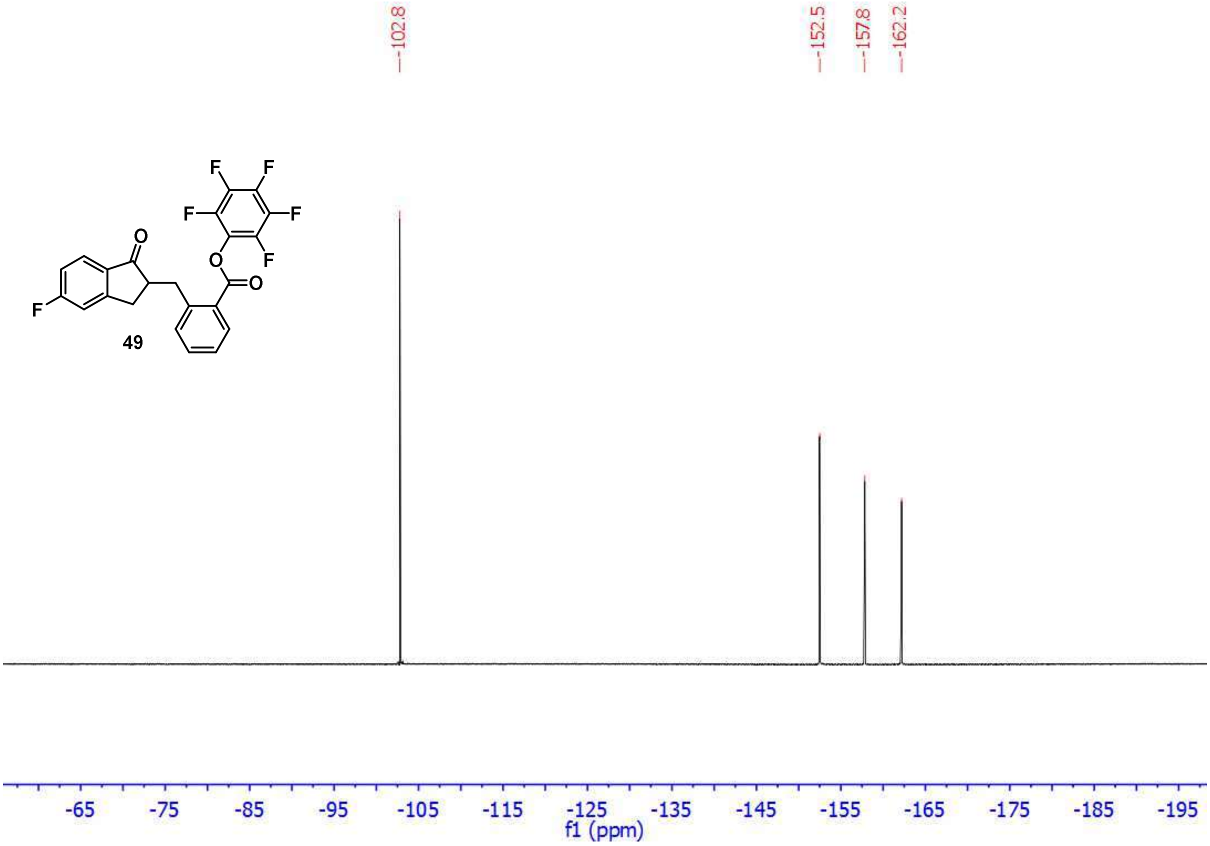
-102.8
-152.5
-157.8
-162.2
49
-65 -75 -85 -95 -105 -115 -125 -135 -145 -155 -165 -175 -185 -195
f1 (ppm)

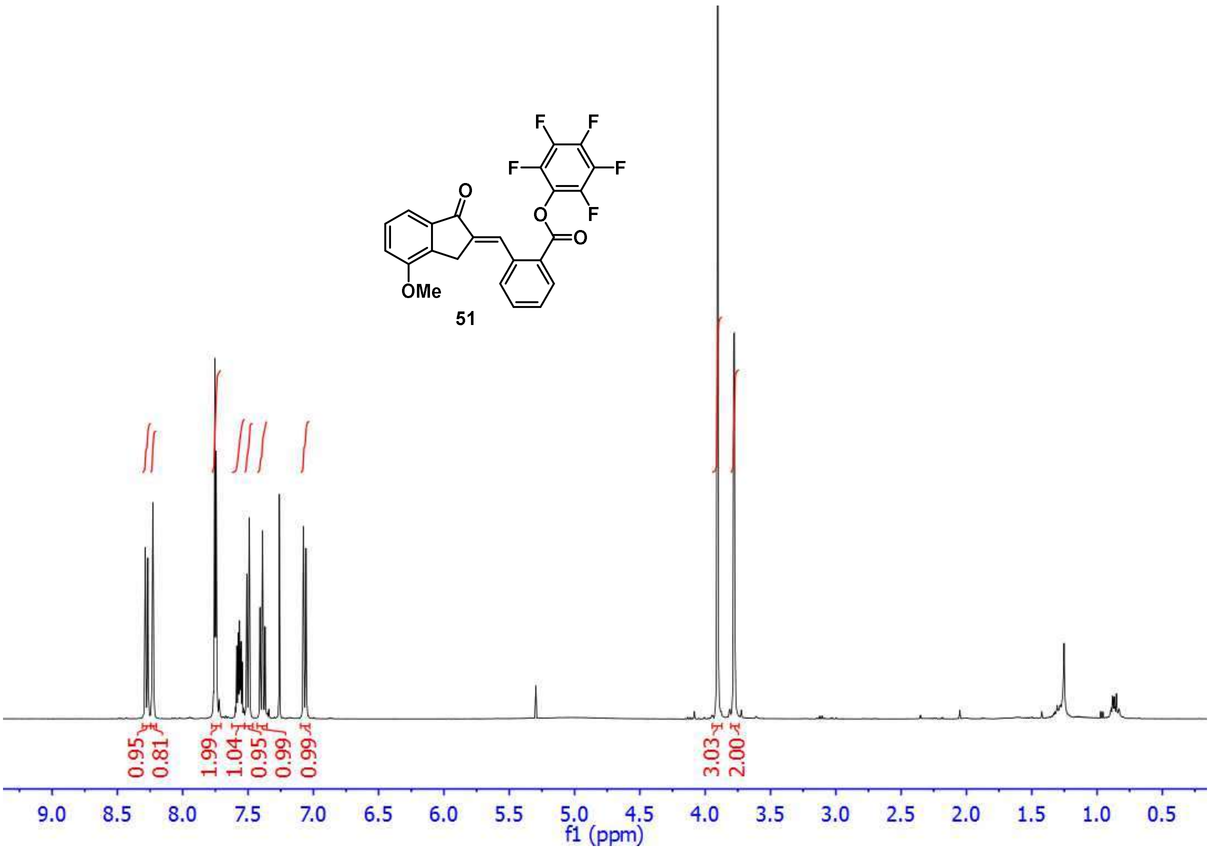
51
OMe
0.95
0.81
1.99
1.04
0.95
0.99
0.99
3.03
2.00
9.0 8.5 8.0 7.5 7.0 6.5 6.0 5.5 5.0 4.5 4.0 3.5 3.0 2.5 2.0 1.5 1.0 0.5
f1 (ppm)

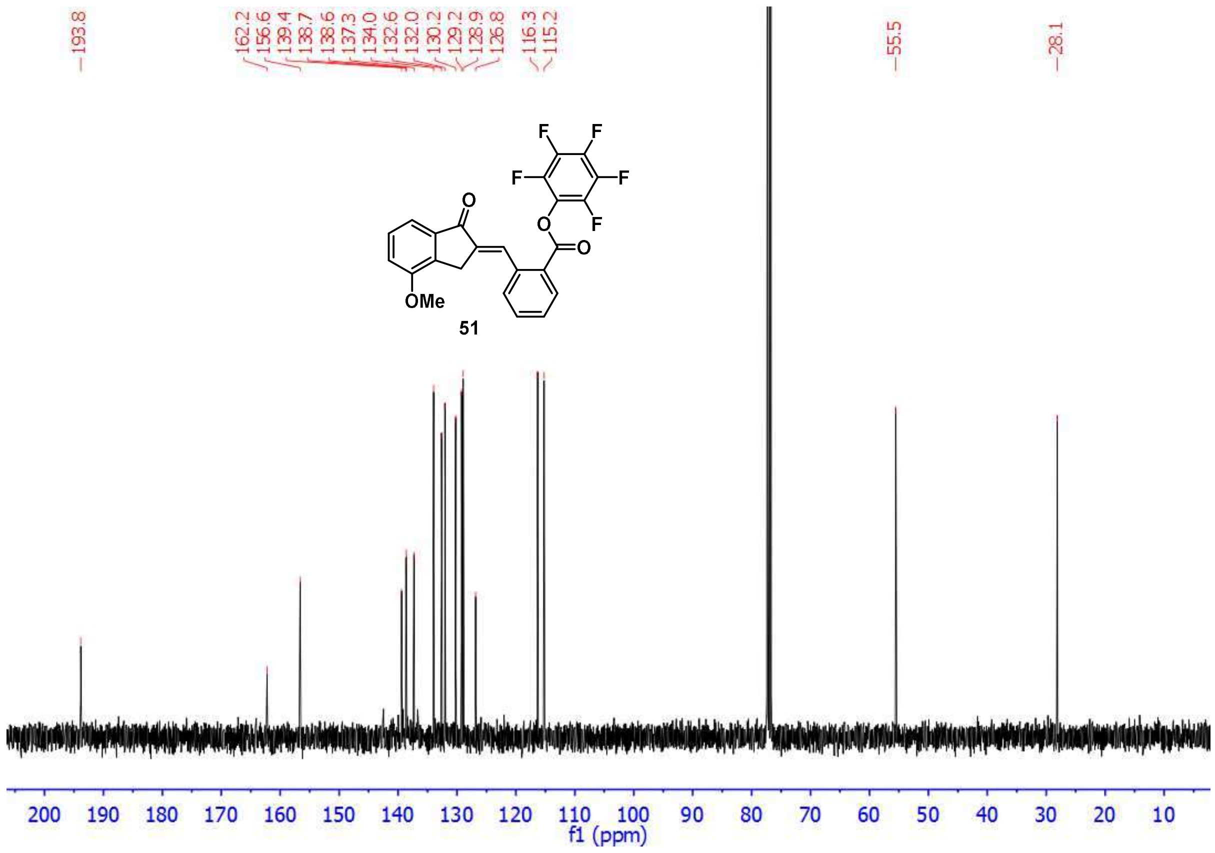
193.8
162.2
156.6
139.4
138.7
138.6
137.3
134.0
132.6
132.0
130.2
129.2
128.9
126.8
116.3
115.2
55.5
28.1
F
F
F
F
F
O
O
O
OMe
51
200 190 180 170 160 150 140 130 120 110 100 90 80 70 60 50 40 30 20 10
f1 (ppm)

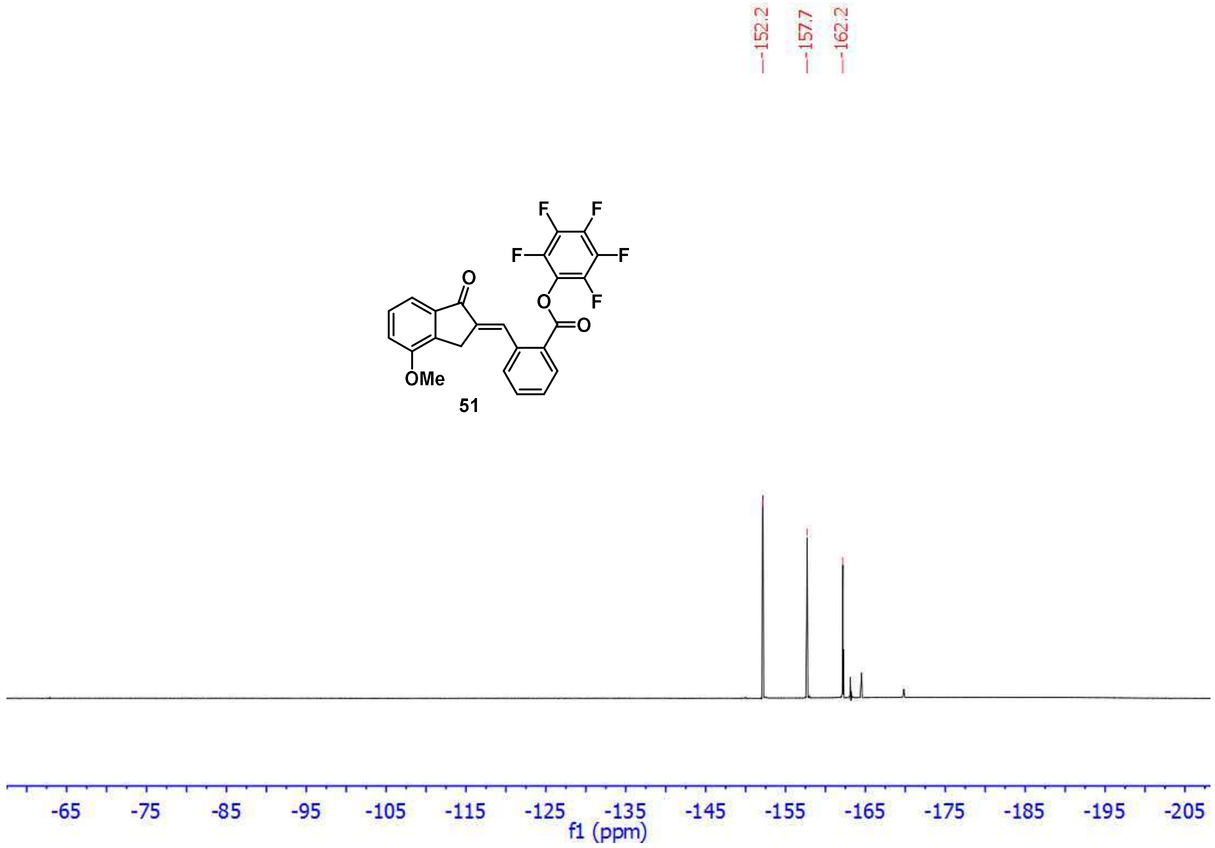
-152.2
-157.7
-162.2
F
F
F
F
F
O
O
O
OMe
51
-65 -75 -85 -95 -105 -115 -125 -135 -145 -155 -165 -175 -185 -195 -205
f1 (ppm)

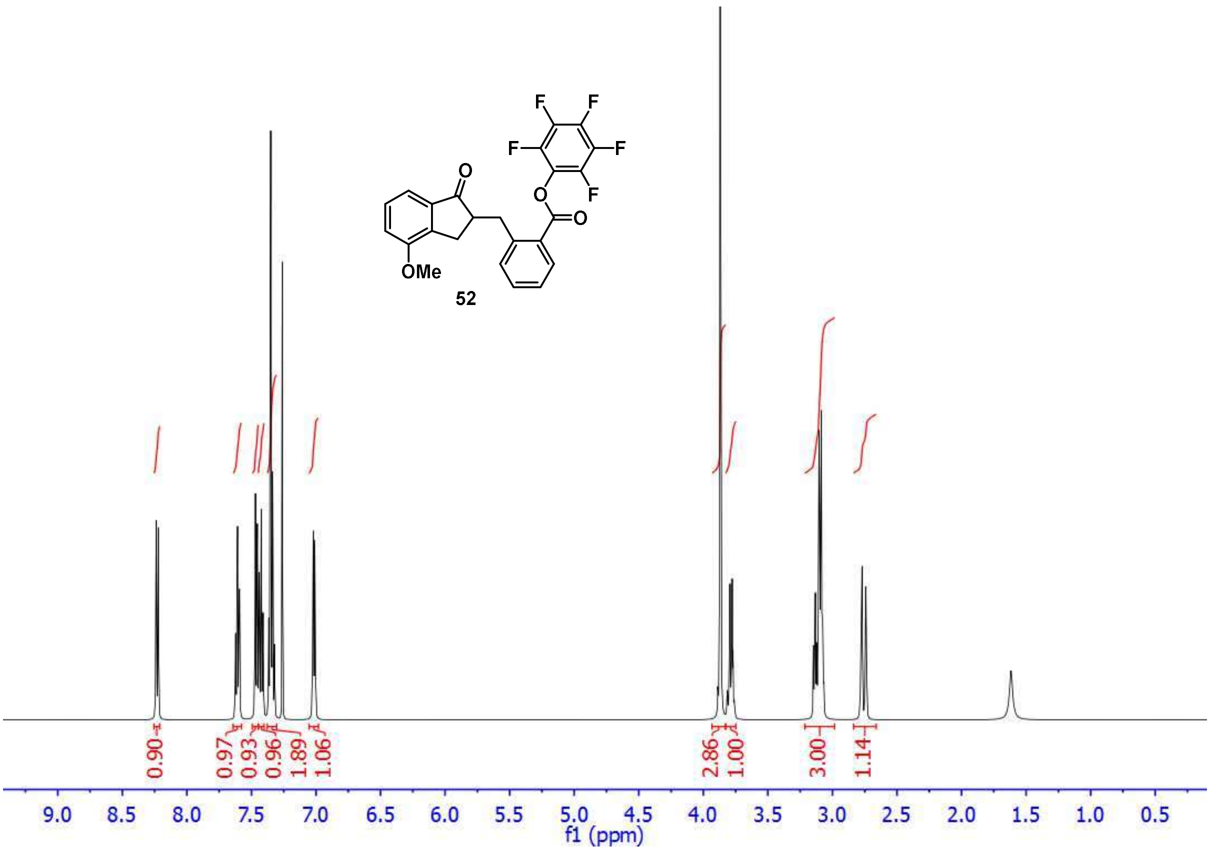
52
0.90
0.97
0.93
0.96
1.89
1.06
2.86
1.00
3.00
1.14
9.0 8.5 8.0 7.5 7.0 6.5 6.0 5.5 5.0 4.5 4.0 3.5 3.0 2.5 2.0 1.5 1.0 0.5
f1 (ppm)

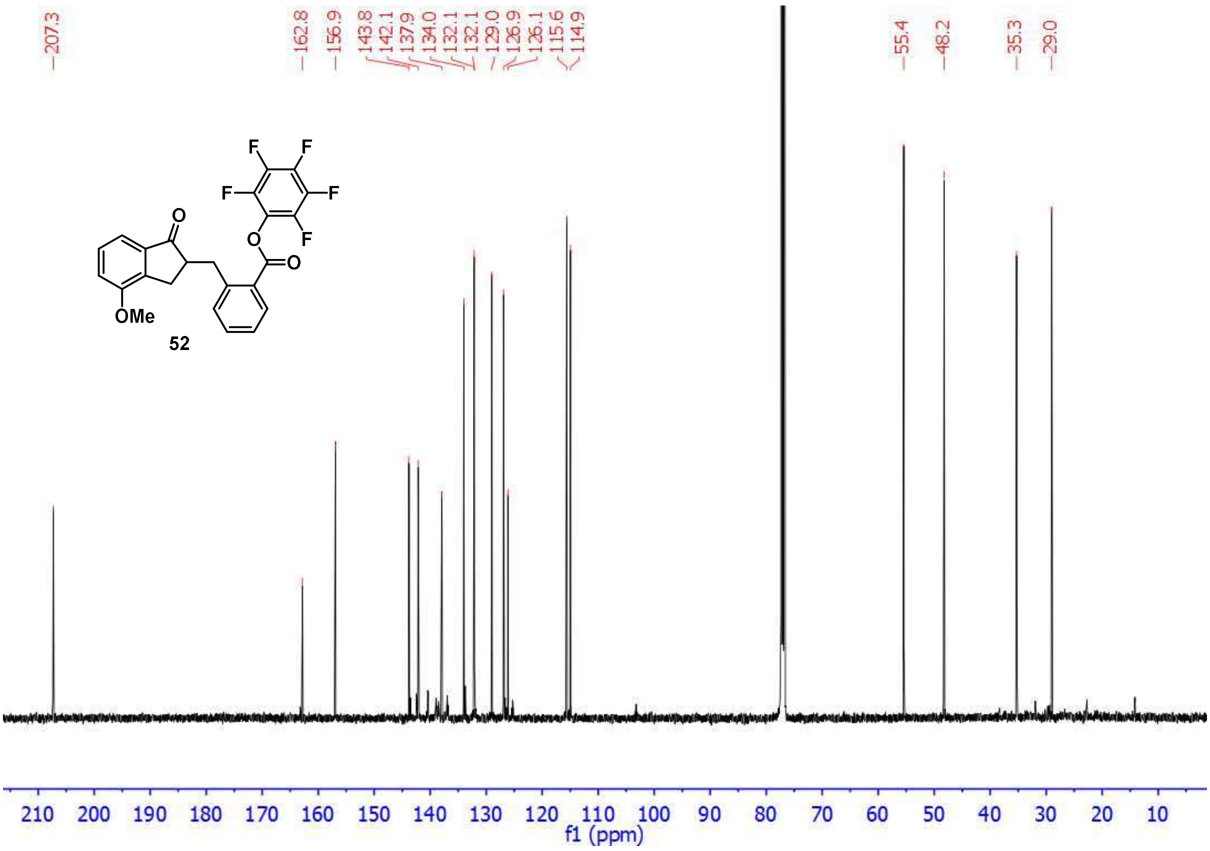
207.3
162.8
156.9
143.8
142.1
137.9
134.0
132.1
132.1
129.0
126.9
126.1
115.6
114.9
55.4
48.2
35.3
29.0
52
210 200 190 180 170 160 150 140 130 120 110 100 90 80 70 60 50 40 30 20 10
f1 (ppm)

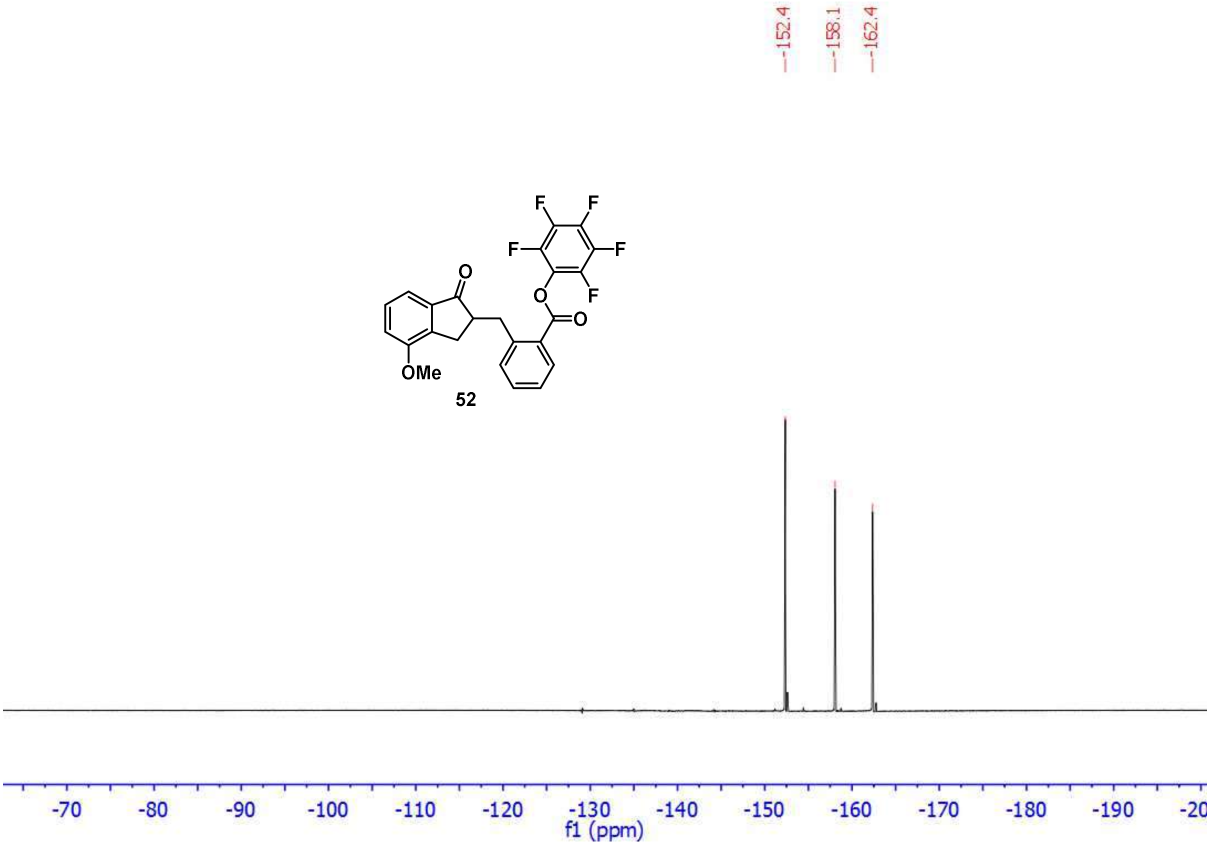

-152.4
-158.1
-162.4
52
OMe
-70 -80 -90 -100 -110 -120 -130 -140 -150 -160 -170 -180 -190
f1 (ppm)


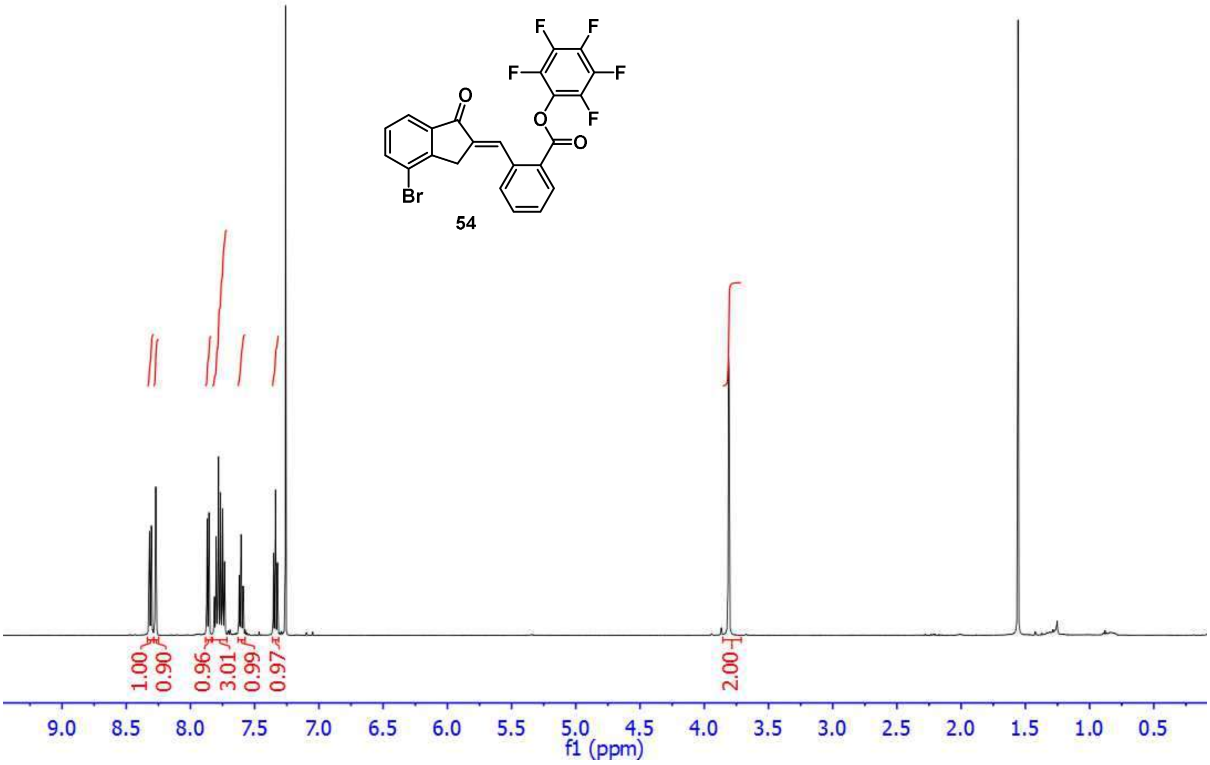

54
Br
1.00
0.90
0.96
3.01
0.99
0.97
2.00
9.0 8.5 8.0 7.5 7.0 6.5 6.0 5.5 5.0 4.5 4.0 3.5 3.0 2.5 2.0 1.5 1.0 0.5
f1 (ppm)

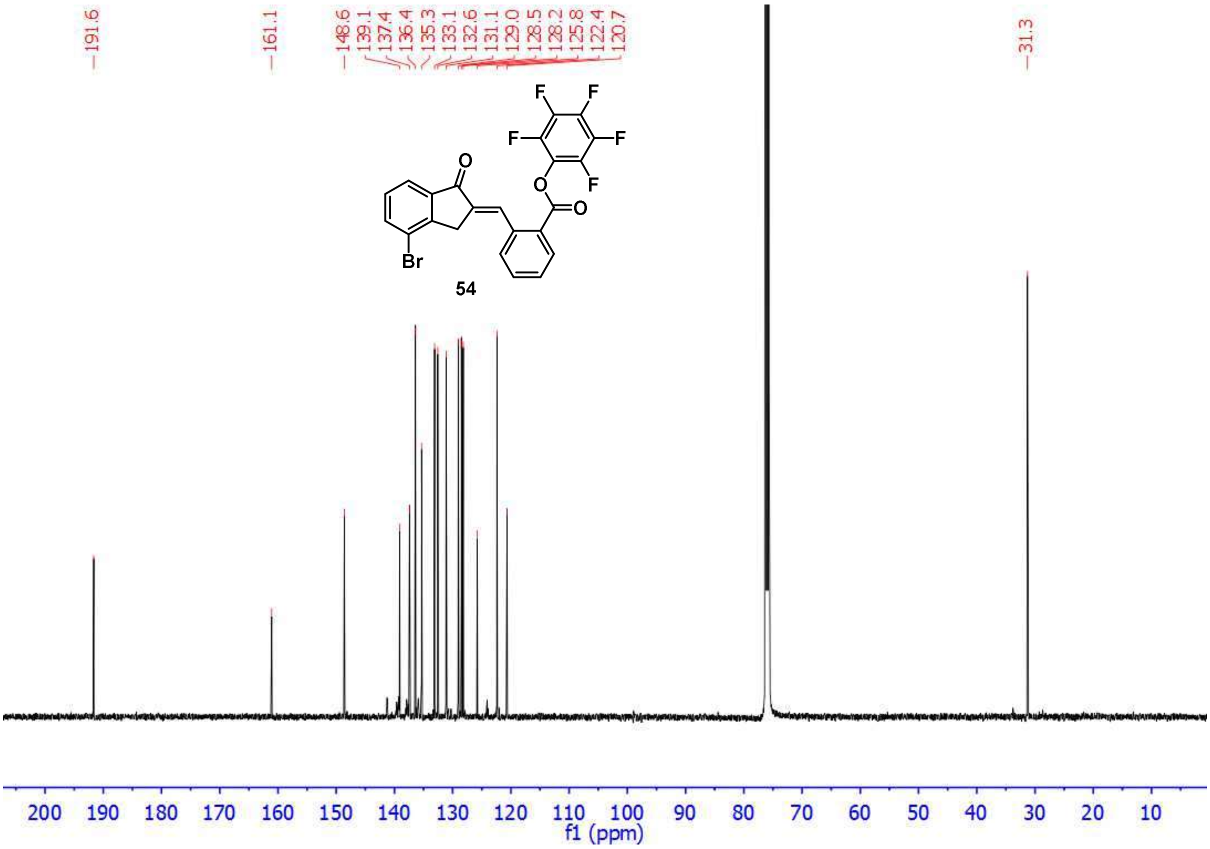
191.6
161.1
148.6
139.1
137.4
136.4
135.3
133.1
132.6
131.1
129.0
128.5
128.2
125.8
122.4
120.7
31.3
54
200 190 180 170 160 150 140 130 120 110 100 90 80 70 60 50 40 30 20 10
f1 (ppm)

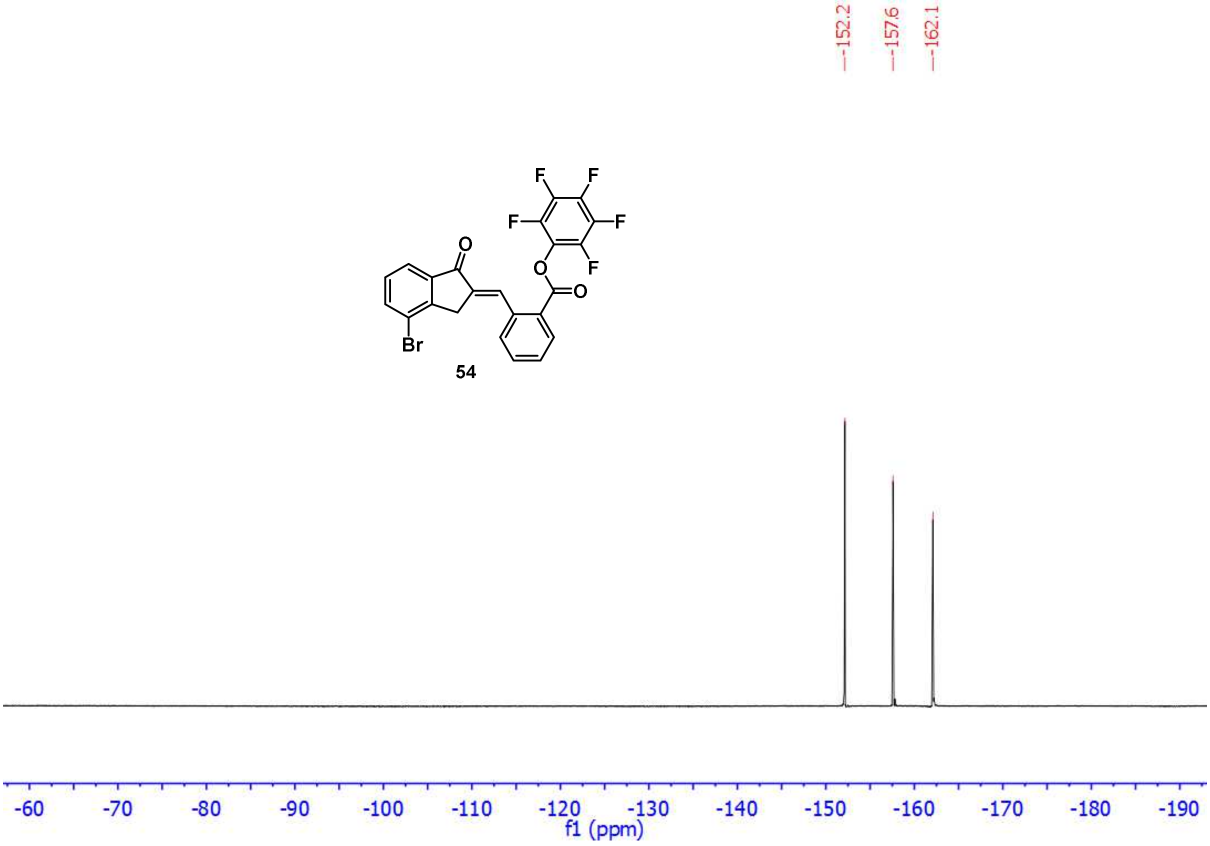
-152.2
-157.6
-162.1
54
-60 -70 -80 -90 -100 -110 -120 -130 -140 -150 -160 -170 -180 -190
f1 (ppm)

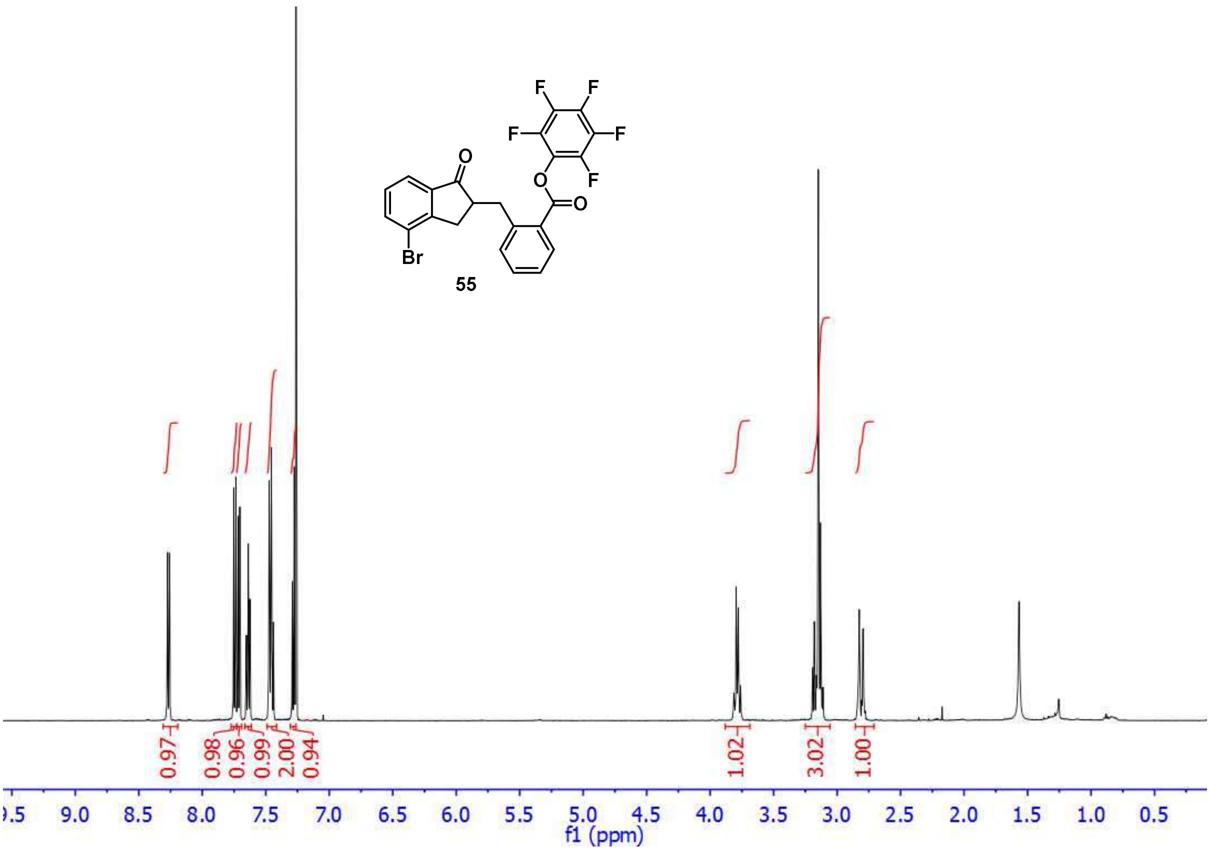

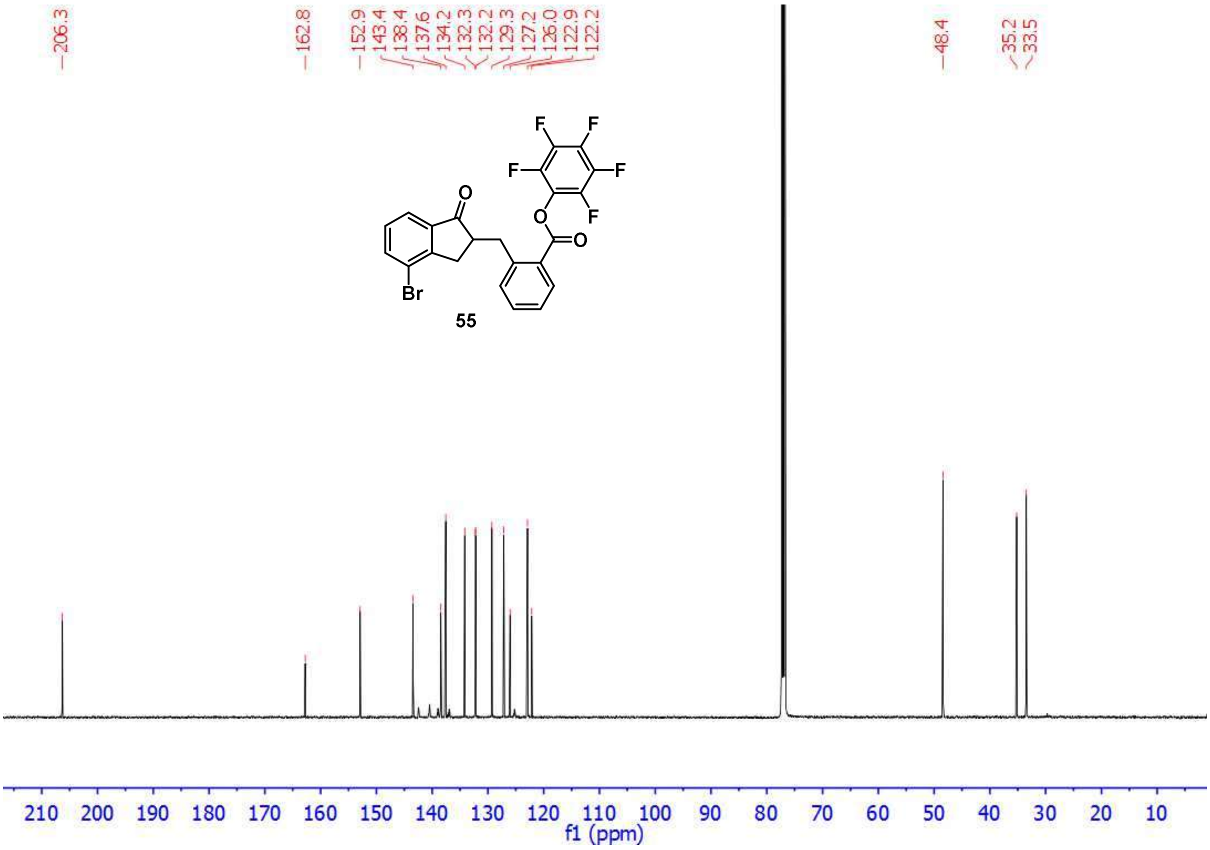

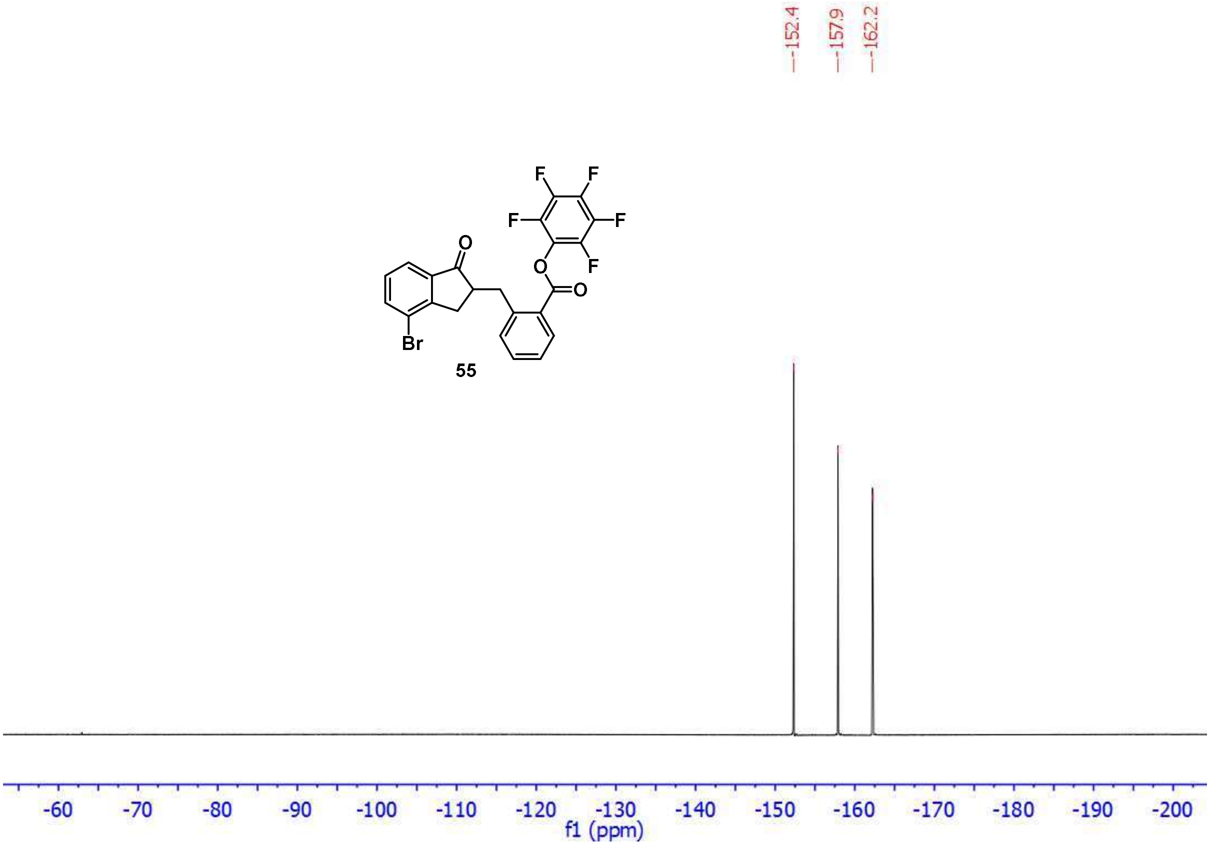

-152.4
-157.9
-162.2
F
F
F
F
F
O
O
O
Br
55
-60
-70
-80
-90
-100
-110
-120
-130
-140
-150
-160
-170
-180
-190
-200
f1 (ppm)


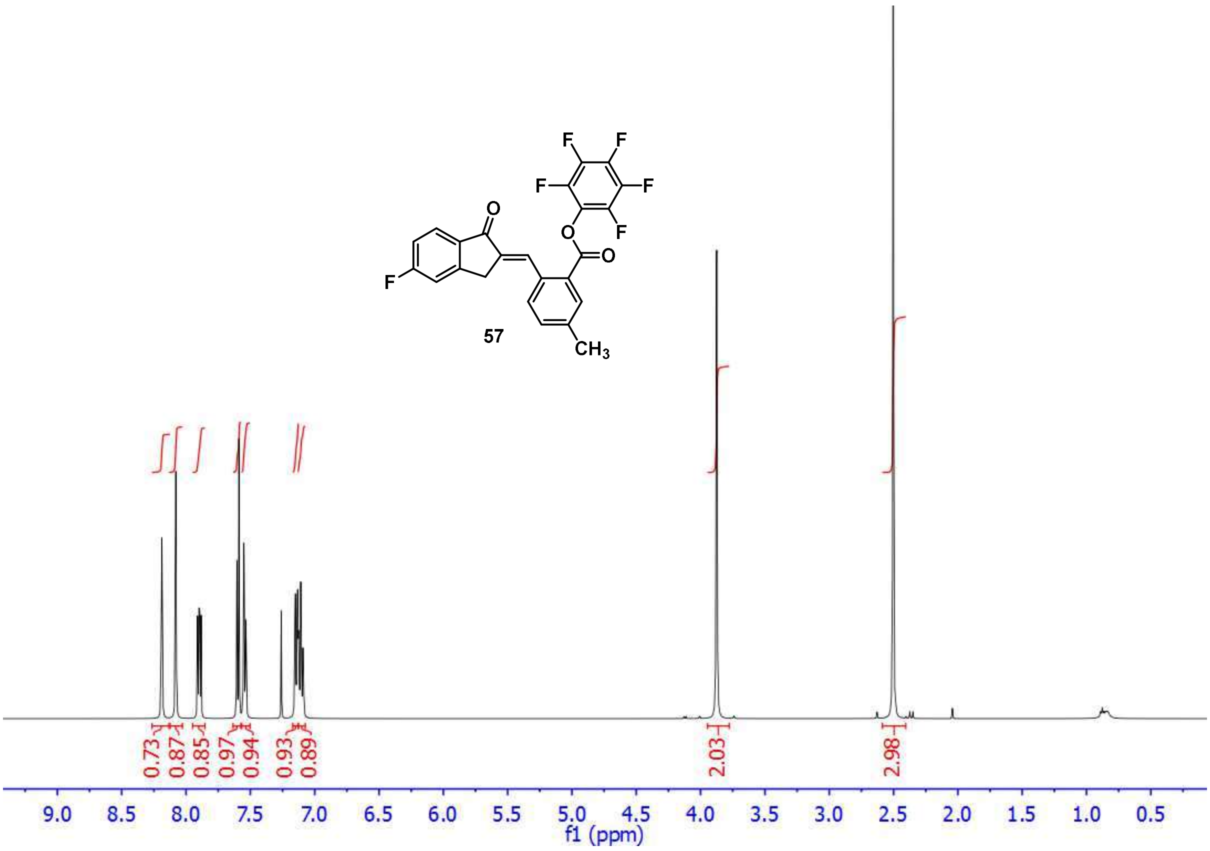

F
F
F
F
F
O
O
O
F
57
CH3
0.73
0.87
0.85
0.97
0.94
0.93
0.89
2.03
2.98
9.0
8.5
8.0
7.5
7.0
6.5
6.0
5.5
5.0
4.5
4.0
3.5
3.0
2.5
2.0
1.5
1.0
0.5
f1 (ppm)

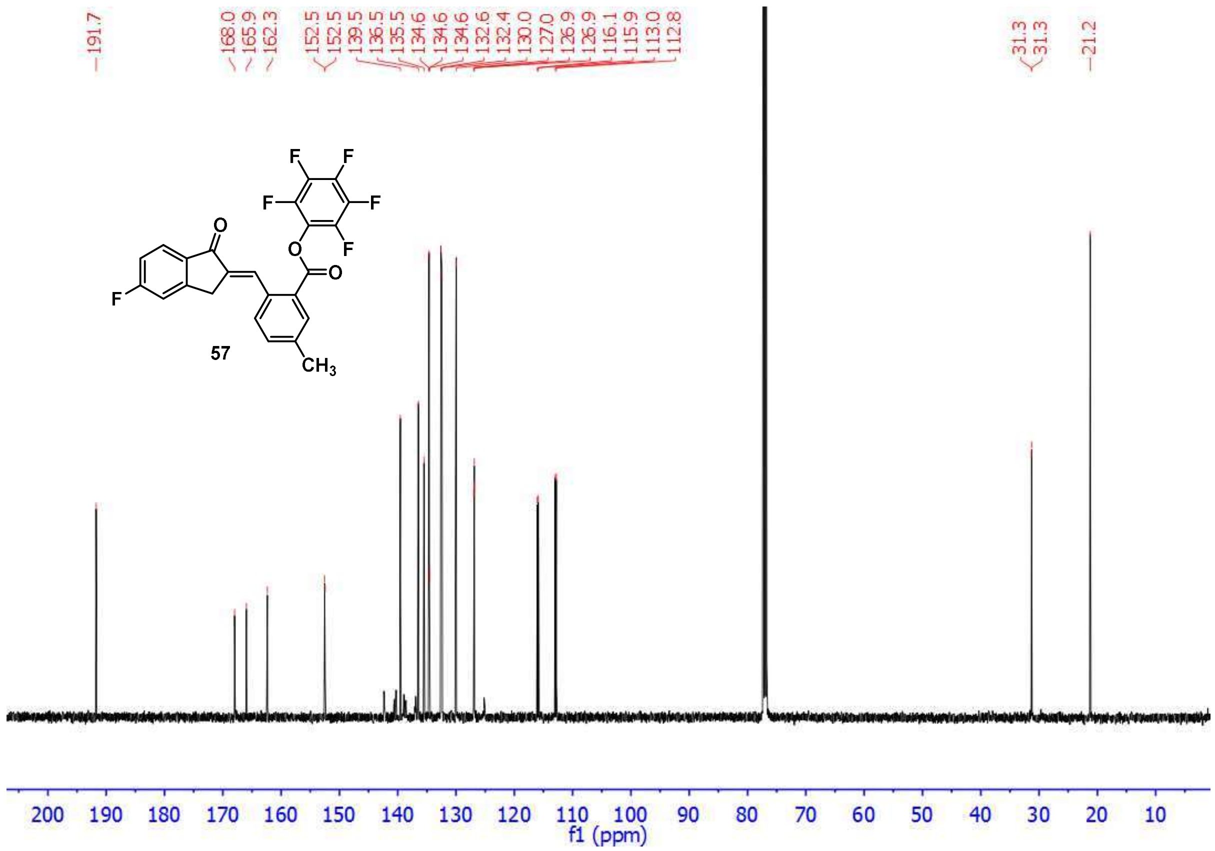
191.7
168.0
165.9
162.3
152.5
152.5
139.5
136.5
135.5
134.6
134.6
134.6
132.6
132.4
130.0
127.0
126.9
126.9
116.1
115.9
113.0
112.8
31.3
31.3
21.2
O
F
F
F
F
F
O
O
F
57
CH3
200 190 180 170 160 150 140 130 120 110 100 90 80 70 60 50 40 30 20 10
f1 (ppm)

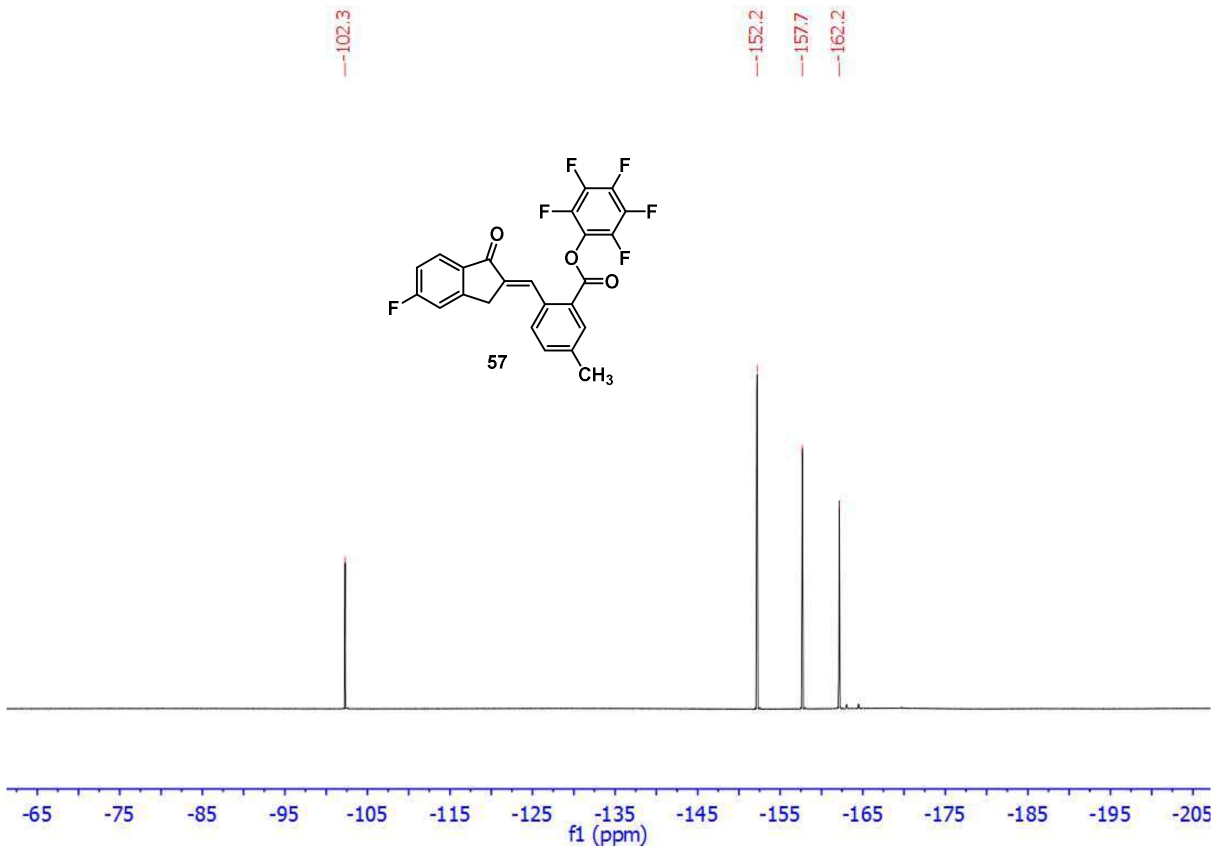
-102.3
-152.2
-157.7
-162.2
O
F
F
F
F
F
O
O
F
57
CH3
-65 -75 -85 -95 -105 -115 -125 -135 -145 -155 -165 -175 -185 -195 -205
f1 (ppm)

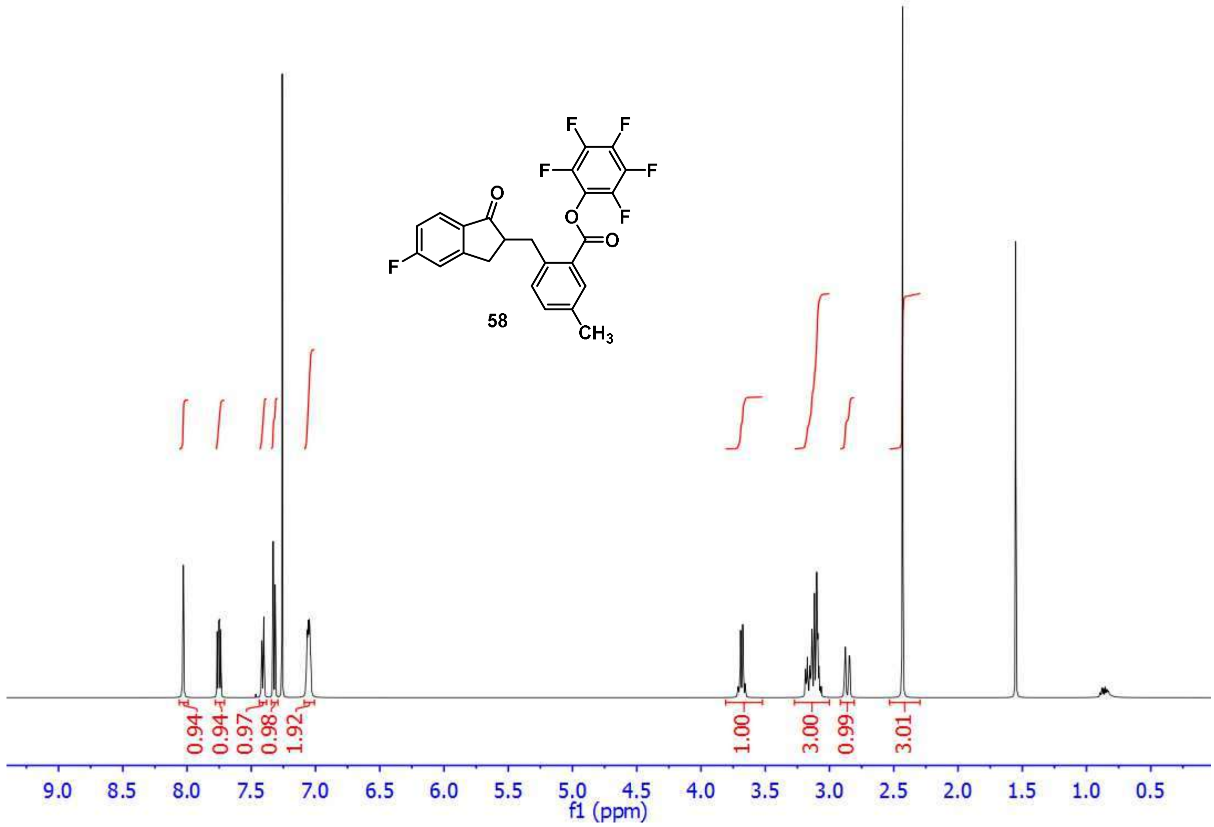

F
O
CH3
58
0.94
0.94
0.97
0.98
1.92
1.00
3.00
0.99
3.01
9.0 8.5 8.0 7.5 7.0 6.5 6.0 5.5 5.0 4.5 4.0 3.5 3.0 2.5 2.0 1.5 1.0 0.5
f1 (ppm)


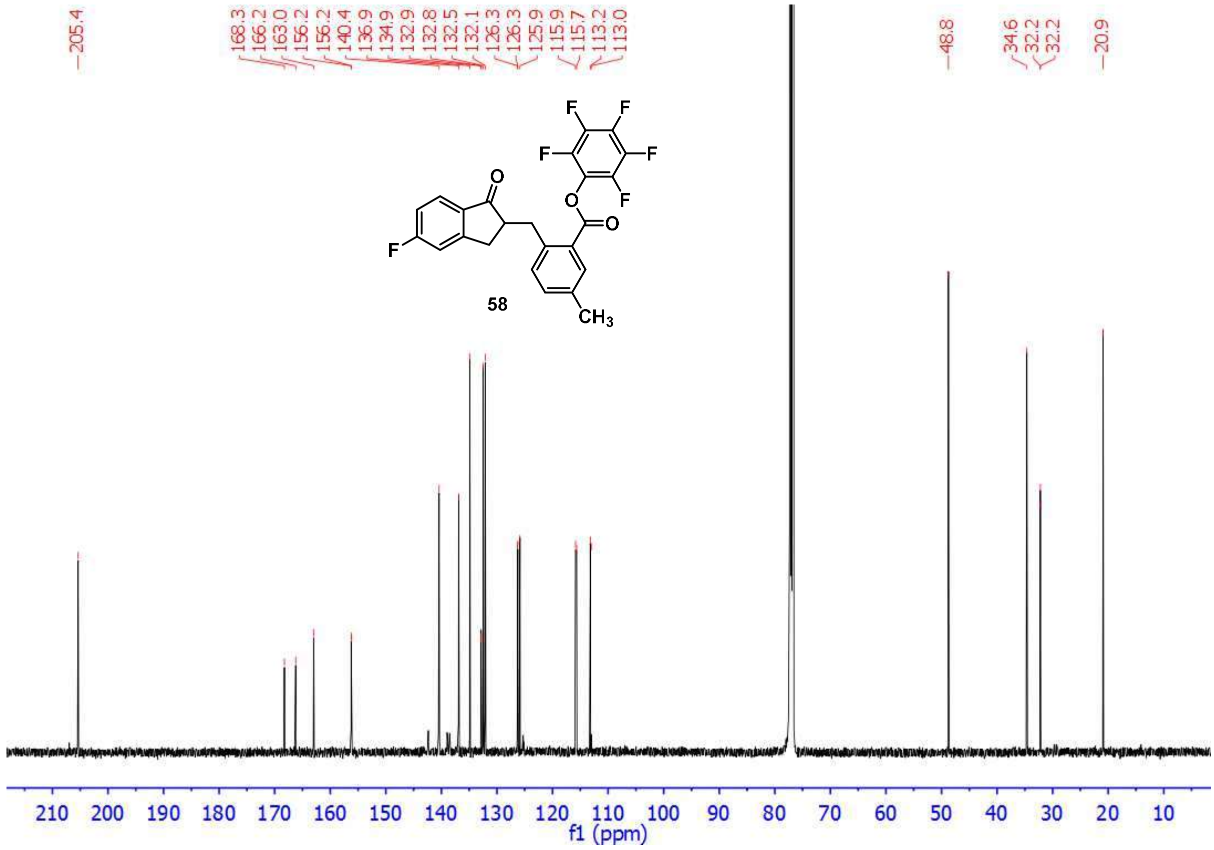

205.4
168.3
166.2
163.0
156.2
156.2
140.4
136.9
134.9
132.9
132.8
132.5
132.1
126.3
126.3
125.9
115.9
115.7
113.2
113.0
48.8
34.6
32.2
32.2
20.9
F
O
CH3
58
210 200 190 180 170 160 150 140 130 120 110 100 90 80 70 60 50 40 30 20 10
f1 (ppm)

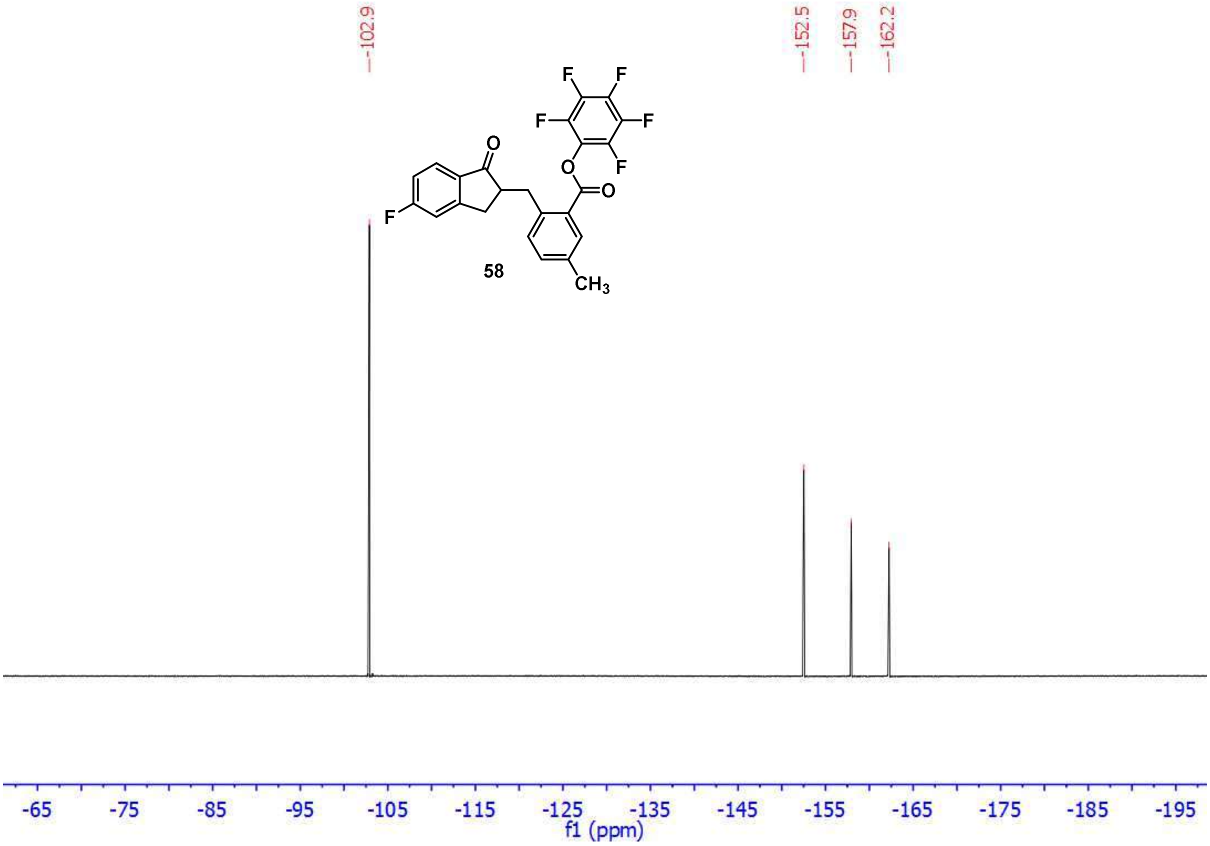

-102.9
-152.5
-157.9
-162.2
58
-65 -75 -85 -95 -105 -115 -125 -135 -145 -155 -165 -175 -185 -195
f1 (ppm)


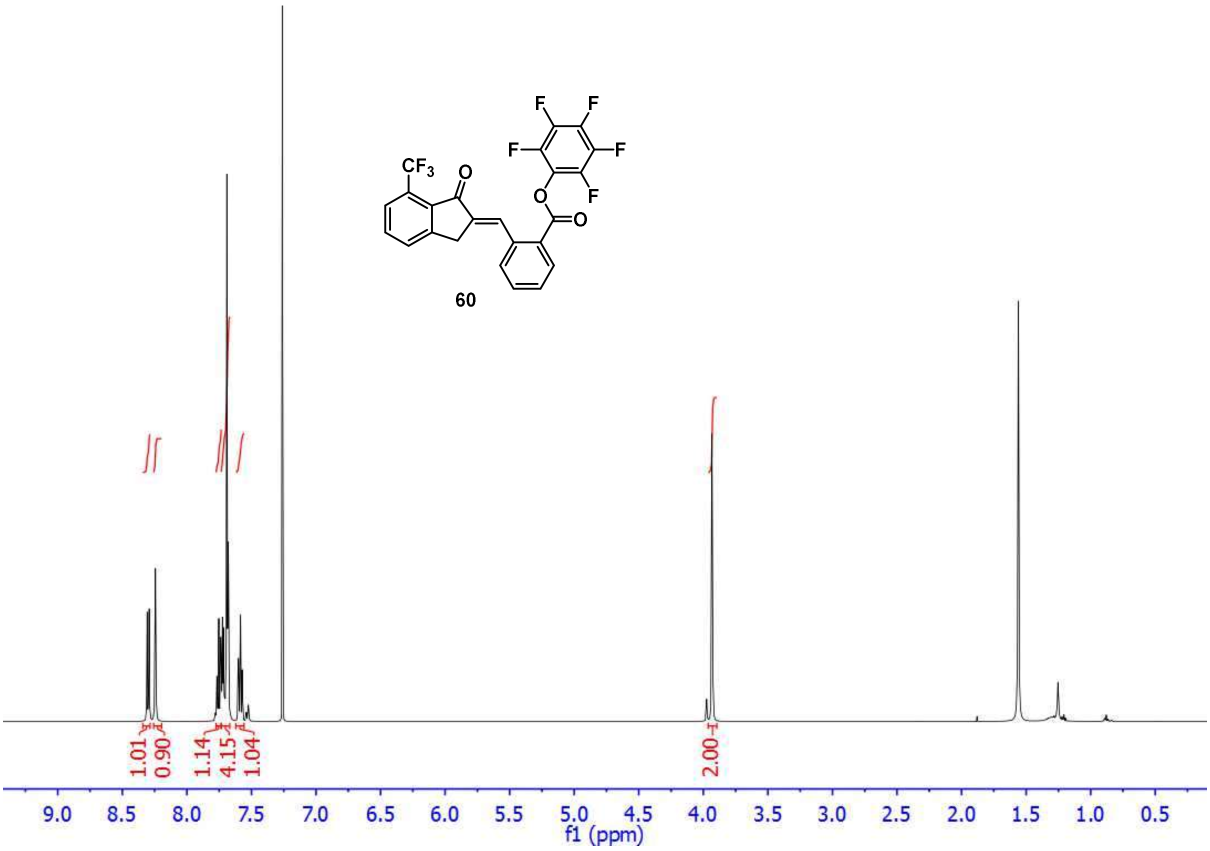

60
1.01
0.90
1.14
4.15
1.04
2.00
9.0 8.5 8.0 7.5 7.0 6.5 6.0 5.5 5.0 4.5 4.0 3.5 3.0 2.5 2.0 1.5 1.0 0.5
f1 (ppm)

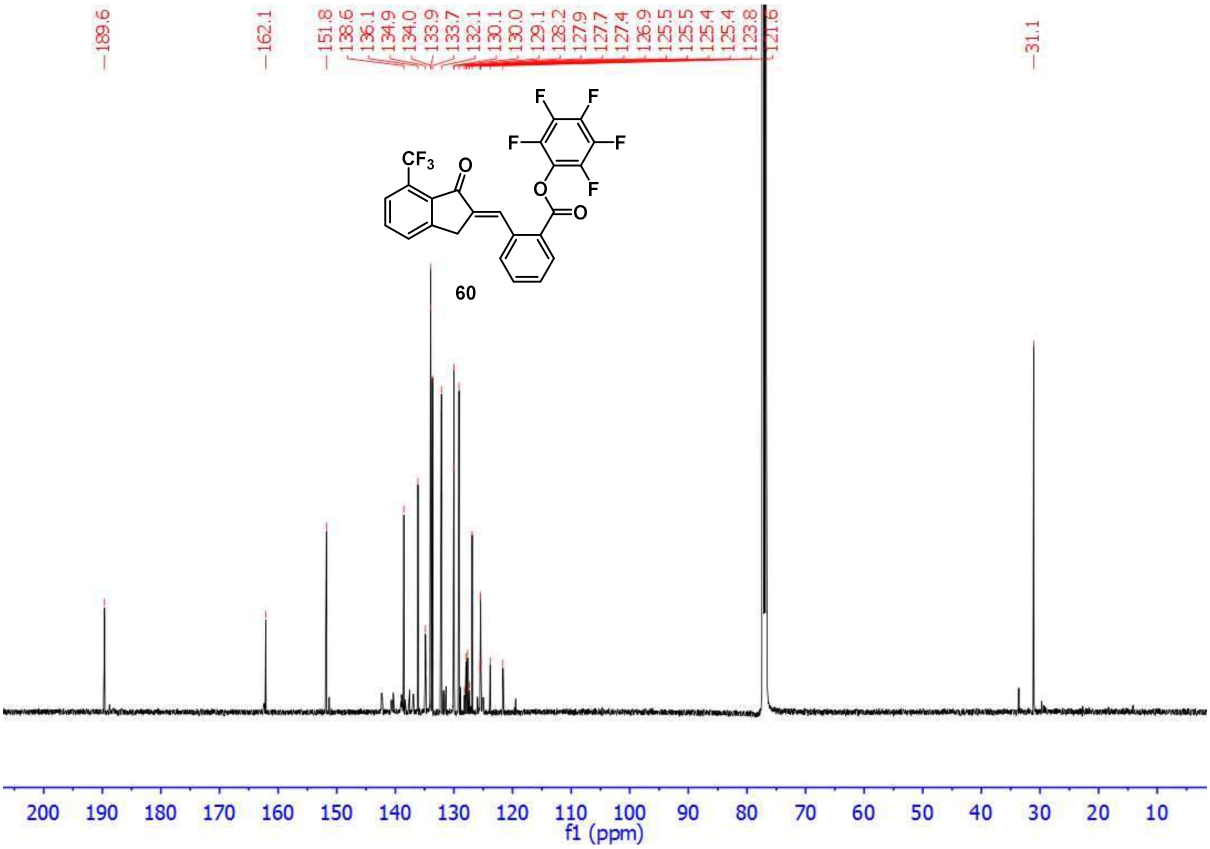
189.6
162.1
151.8
138.6
136.1
134.9
134.0
133.9
133.7
132.1
130.1
130.0
129.1
128.2
127.9
127.7
127.4
126.9
125.5
125.5
125.4
125.4
123.8
121.6
31.1
CF3
O
F
F
F
F
F
O
O
60
200
190
180
170
160
150
140
130
120
110
100
90
80
70
60
50
40
30
20
10
f1 (ppm)

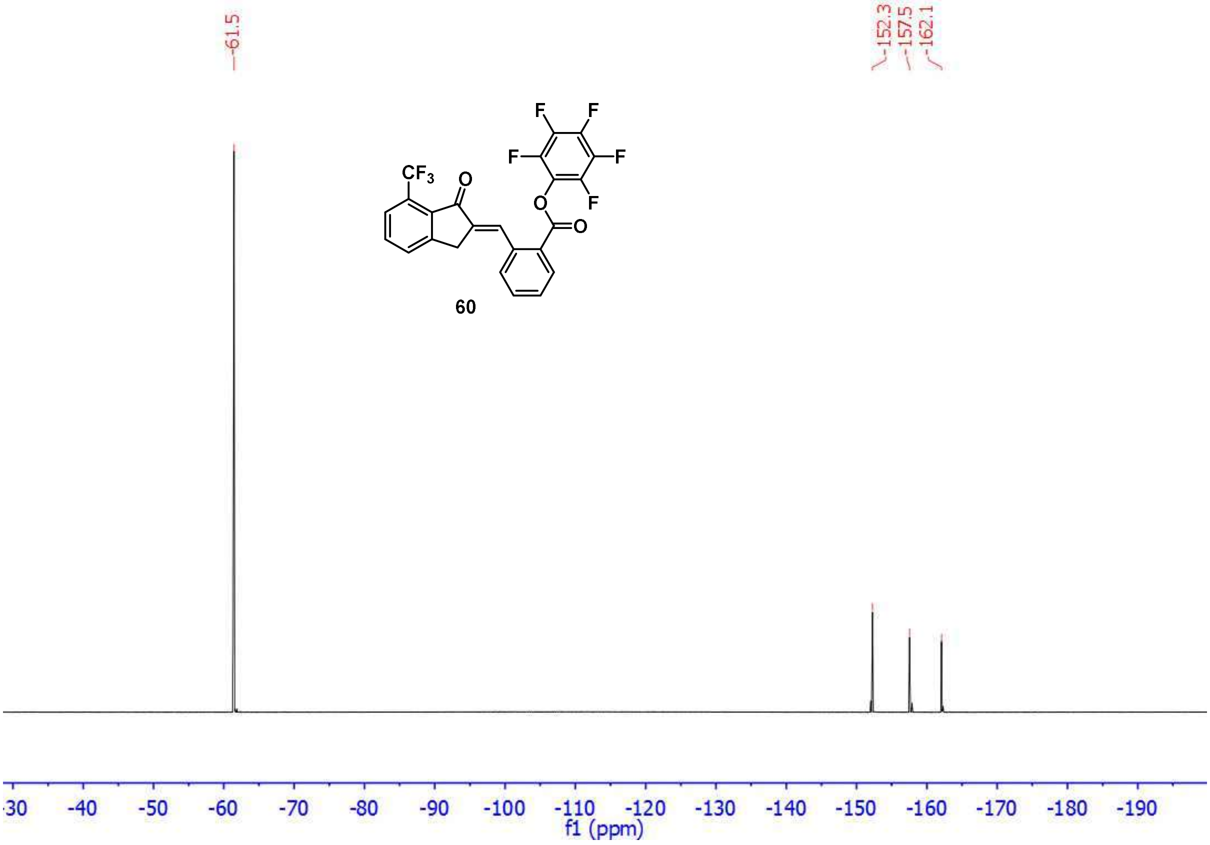
-61.5
-152.3
-157.5
-162.1
CF3
O
F
F
F
F
F
O
O
60
-40
-50
-60
-70
-80
-90
-100
-110
-120
-130
-140
-150
-160
-170
-180
-190
f1 (ppm)

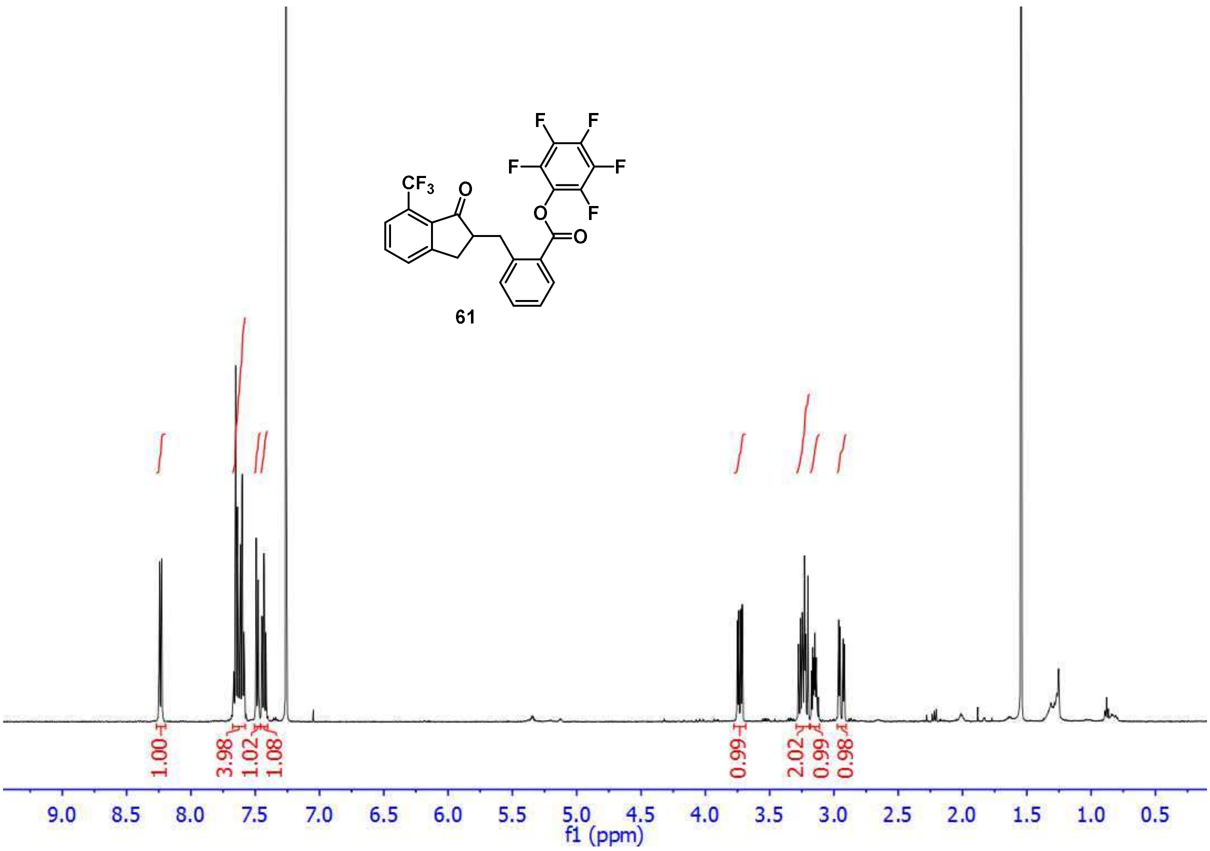
CF3
O
F
F
F
F
F
O
O
61
1.00
3.98
1.02
1.08
0.99
2.02
0.99
0.98
9.0 8.5 8.0 7.5 7.0 6.5 6.0 5.5 5.0 4.5 4.0 3.5 3.0 2.5 2.0 1.5 1.0 0.5
f1 (ppm)

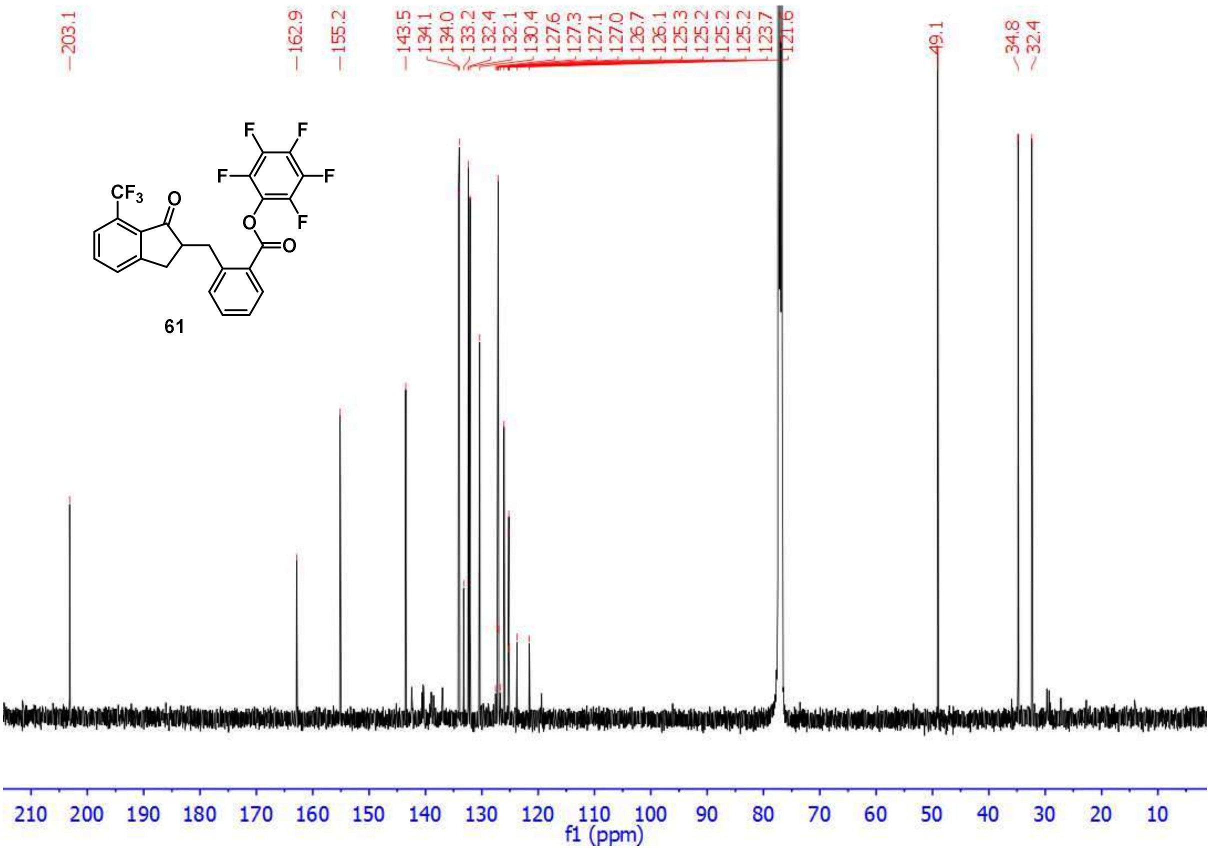
203.1
162.9
155.2
143.5
134.1
134.0
133.2
132.4
132.1
130.4
127.6
127.3
127.1
127.0
126.7
126.1
125.3
125.2
125.2
125.2
123.7
121.6
49.1
34.8
32.4
CF3
O
F
F
F
F
F
O
O
61
210 200 190 180 170 160 150 140 130 120 110 100 90 80 70 60 50 40 30 20 10
f1 (ppm)

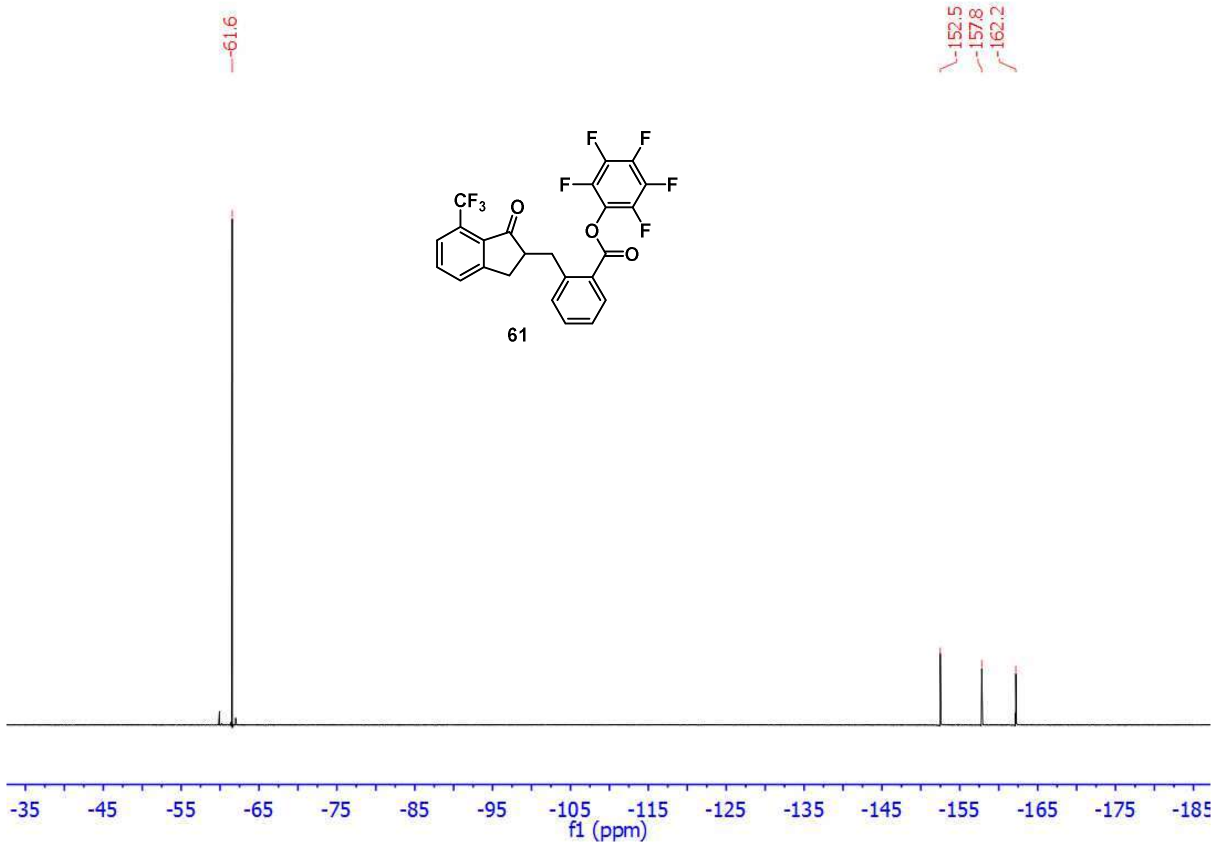
-61.6
-152.5
-157.8
-162.2
CF3
O
F
61
-35 -45 -55 -65 -75 -85 -95 -105 -115 -125 -135 -145 -155 -165 -175 -185
f1 (ppm)

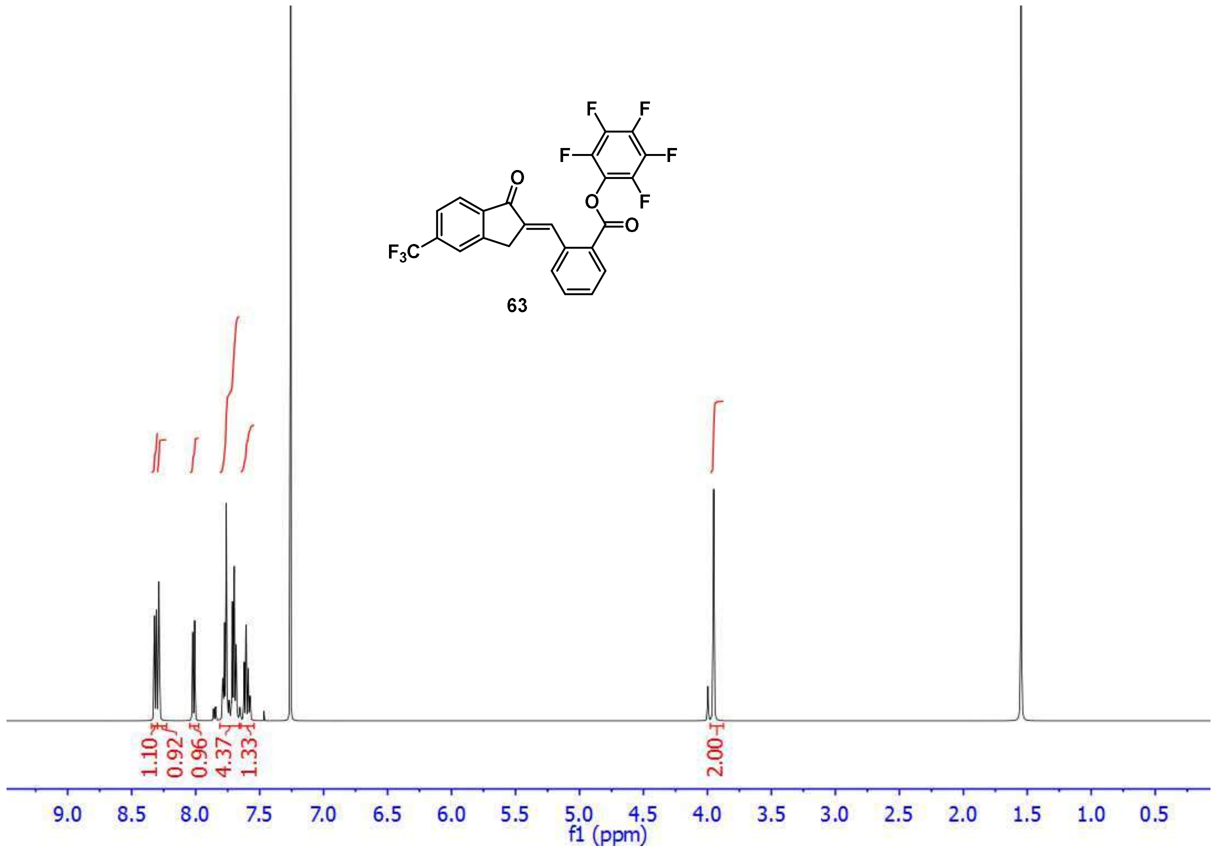
F3C
O
F
63
1.10
0.92
0.96
4.37
1.33
2.00
9.0 8.5 8.0 7.5 7.0 6.5 6.0 5.5 5.0 4.5 4.0 3.5 3.0 2.5 2.0 1.5 1.0 0.5
f1 (ppm)

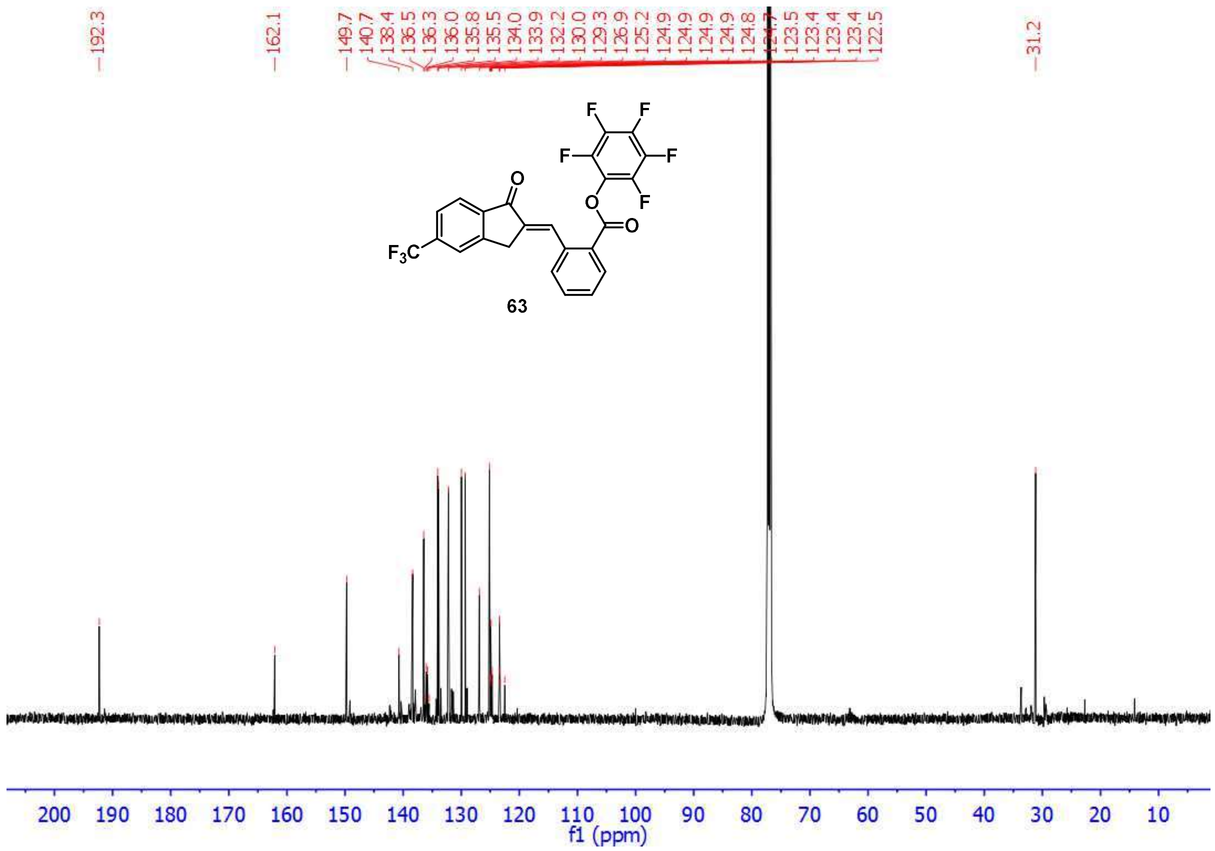
192.3
162.1
149.7
140.7
138.4
136.5
136.3
136.0
135.8
135.5
134.0
133.9
132.2
130.0
129.3
126.9
125.2
124.9
124.9
124.9
124.9
124.8
124.7
123.5
123.4
123.4
123.4
122.5
31.2
F
F
F
F
F
O
O
O
$F_3C$
63
200
190
180
170
160
150
140
130
120
110
100
90
80
70
60
50
40
30
20
10
f1 (ppm)

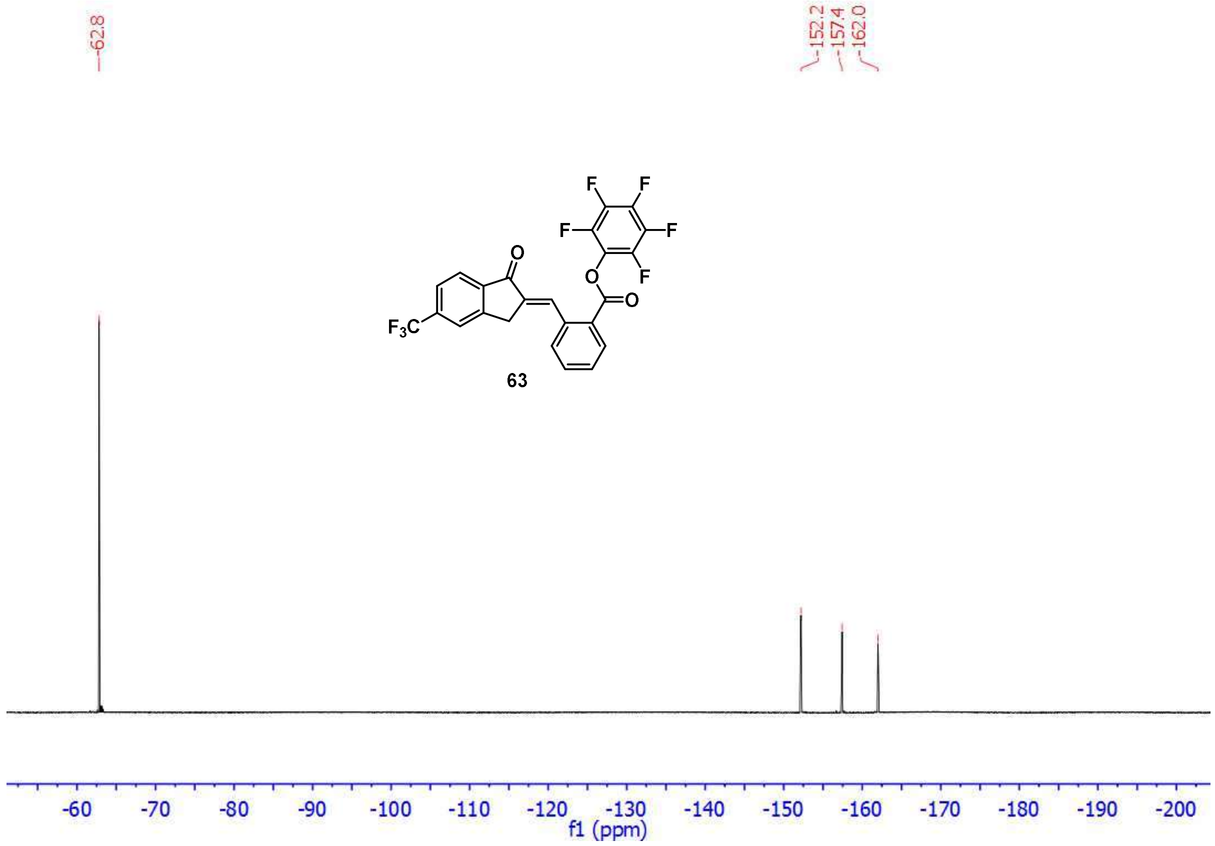
-62.8
-152.2
-157.4
-162.0
F
F
F
F
F
O
O
O
$F_3C$
63
-60
-70
-80
-90
-100
-110
-120
-130
-140
-150
-160
-170
-180
-190
-200
f1 (ppm)

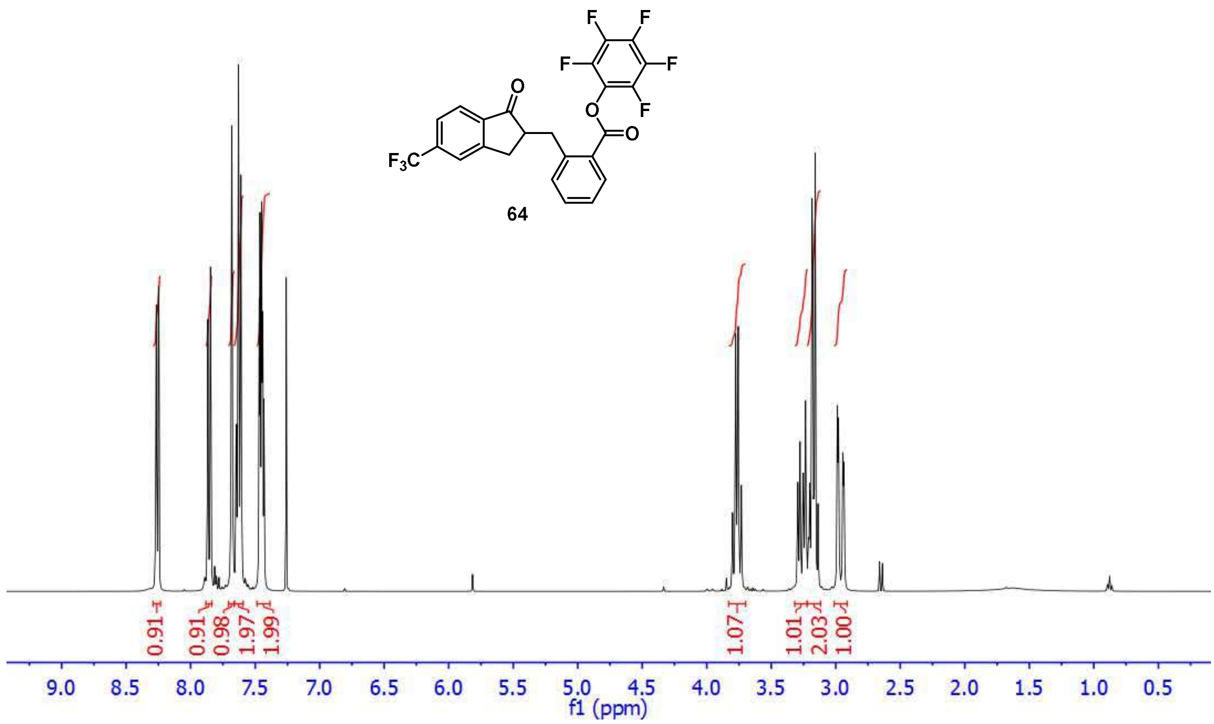

64
0.91
0.91
0.98
1.97
1.99
1.07
1.01
2.03
1.00
9.0 8.5 8.0 7.5 7.0 6.5 6.0 5.5 5.0 4.5 4.0 3.5 3.0 2.5 2.0 1.5 1.0 0.5
f1 (ppm)


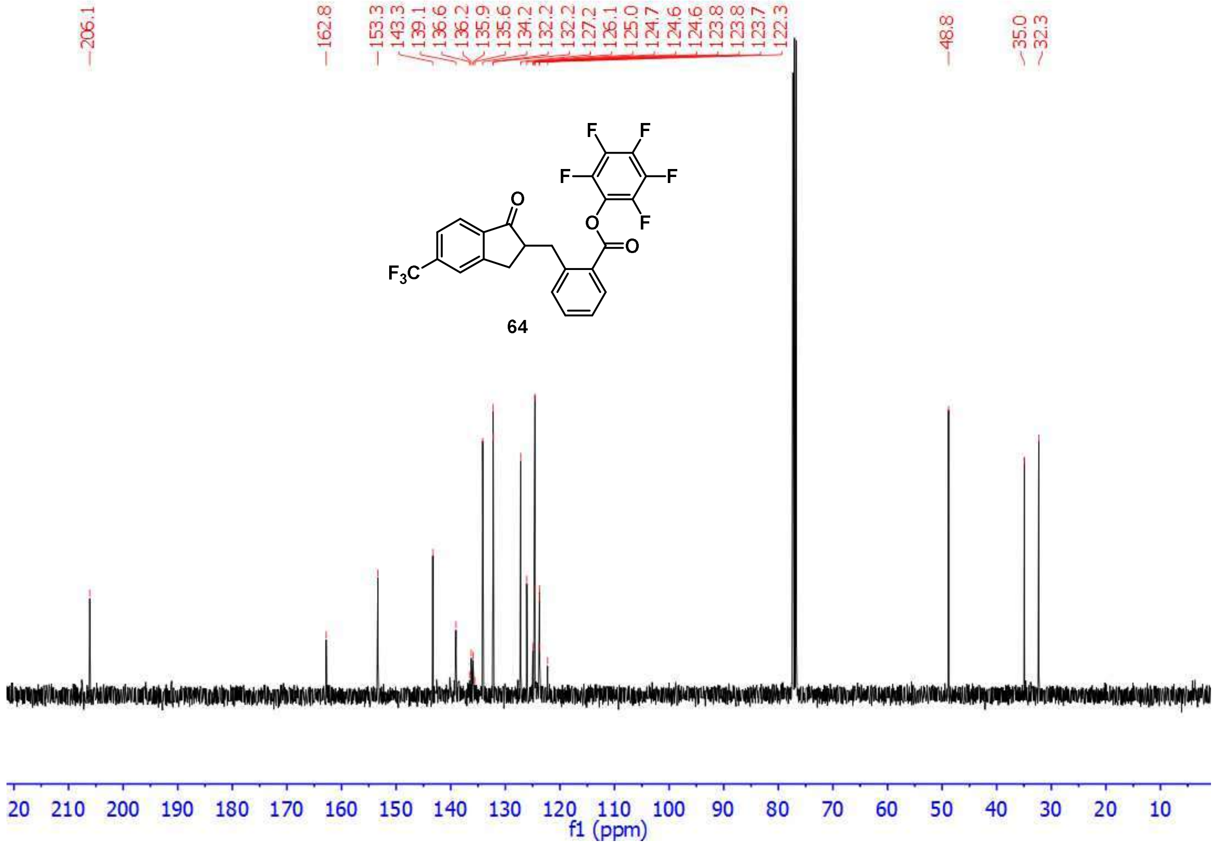

206.1
162.8
153.3
143.3
139.1
136.6
136.2
135.9
135.6
134.2
132.2
132.2
127.2
126.1
125.0
124.7
124.6
124.6
123.8
123.8
123.7
122.3
48.8
35.0
32.3
64
20 210 200 190 180 170 160 150 140 130 120 110 100 90 80 70 60 50 40 30 20 10
f1 (ppm)

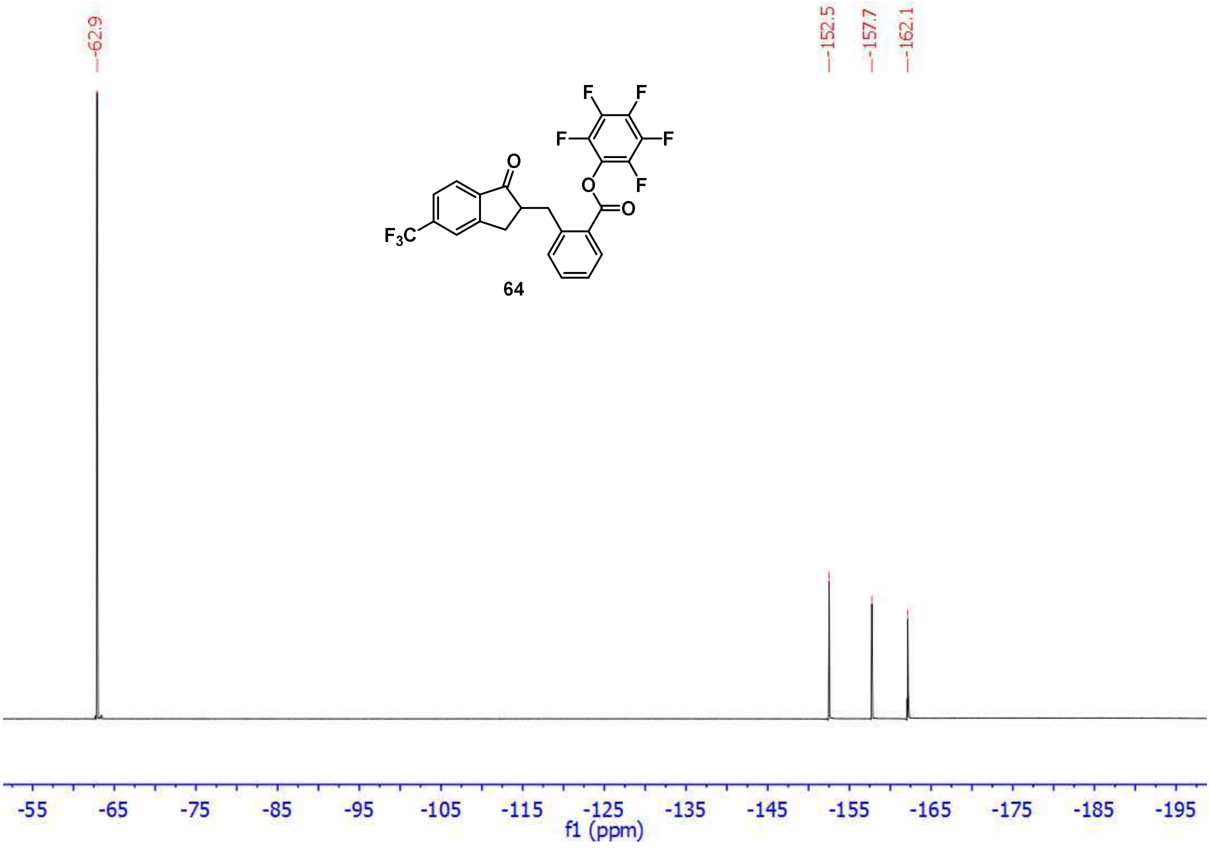
-62.9
-152.5
-157.7
-162.1
64
-55 -65 -75 -85 -95 -105 -115 -125 -135 -145 -155 -165 -175 -185 -195
f1 (ppm)

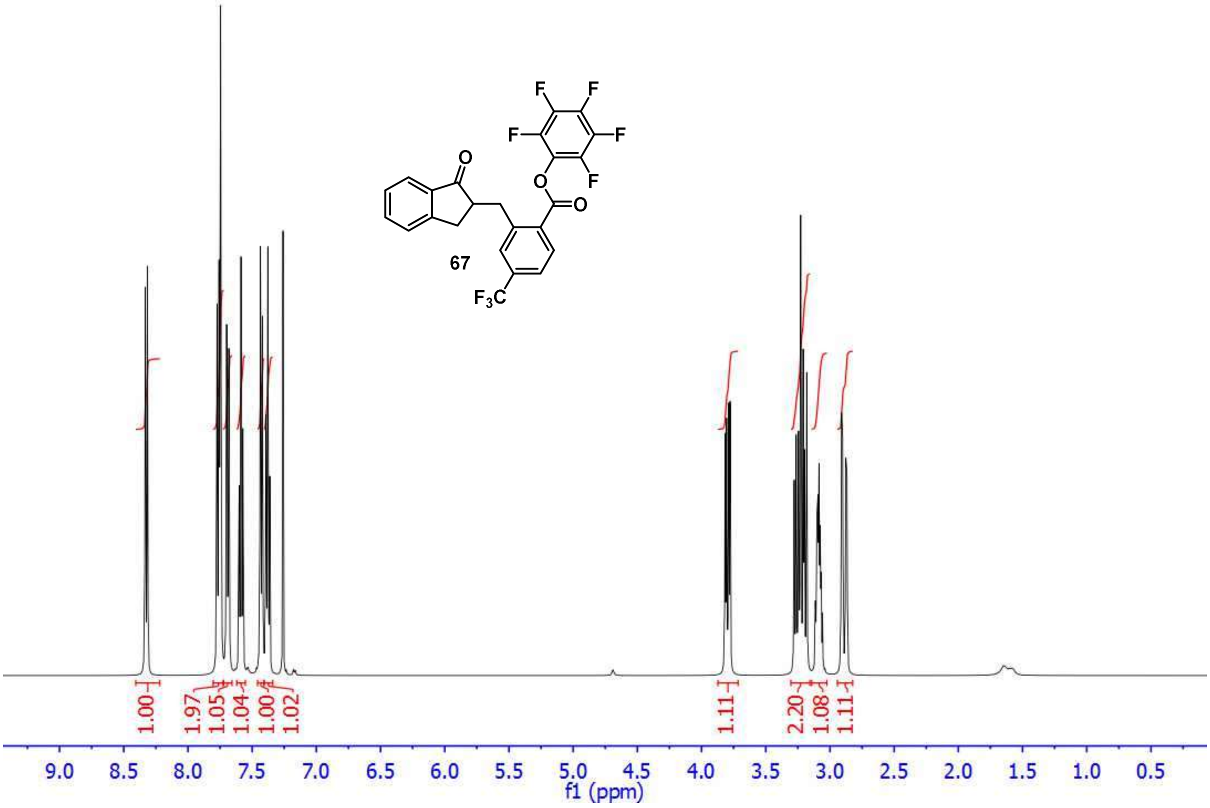
67
1.00
1.97
1.05
1.04
1.00
1.02
1.11
2.20
1.08
1.11
9.0 8.5 8.0 7.5 7.0 6.5 6.0 5.5 5.0 4.5 4.0 3.5 3.0 2.5 2.0 1.5 1.0 0.5
f1 (ppm)

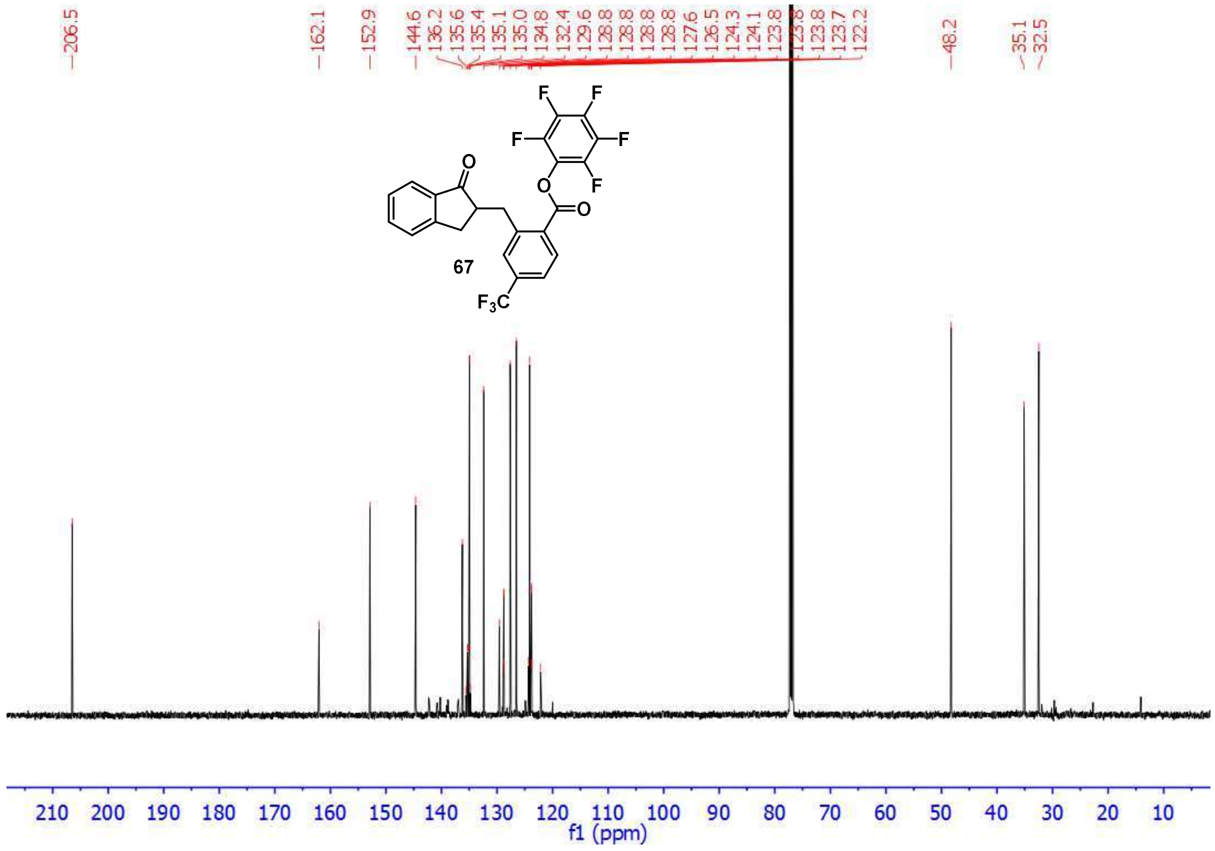
206.5
162.1
152.9
144.6
136.2
135.6
135.4
135.1
135.0
134.8
132.4
129.6
128.8
128.8
128.8
128.8
127.6
126.5
124.3
124.1
123.8
123.8
123.8
123.7
122.2
48.2
35.1
32.5
F
F
F
F
F
O
O
O
67
F3C
210 200 190 180 170 160 150 140 130 120 110 100 90 80 70 60 50 40 30 20 10
f1 (ppm)

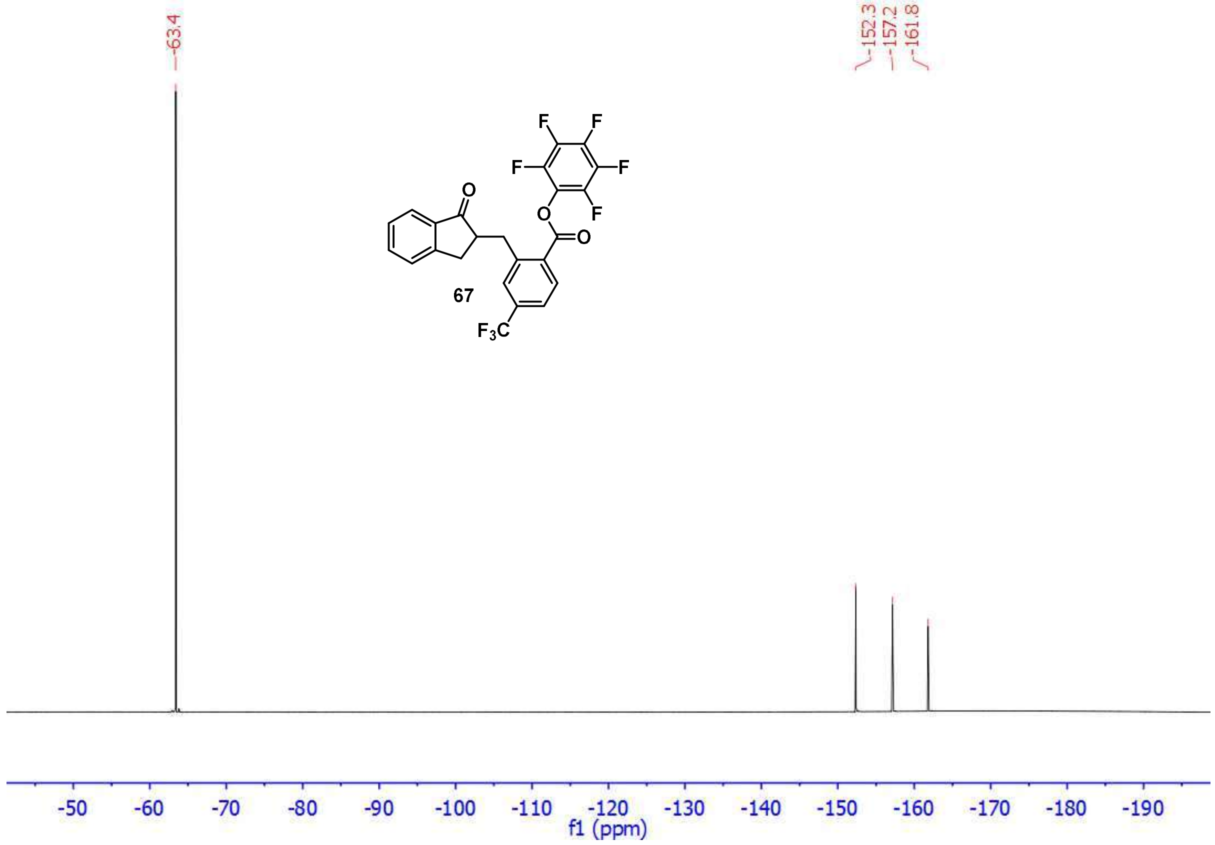
-63.4
-152.3
-157.2
-161.8
F
F
F
F
F
O
O
O
67
F3C
-50 -60 -70 -80 -90 -100 -110 -120 -130 -140 -150 -160 -170 -180 -190
f1 (ppm)

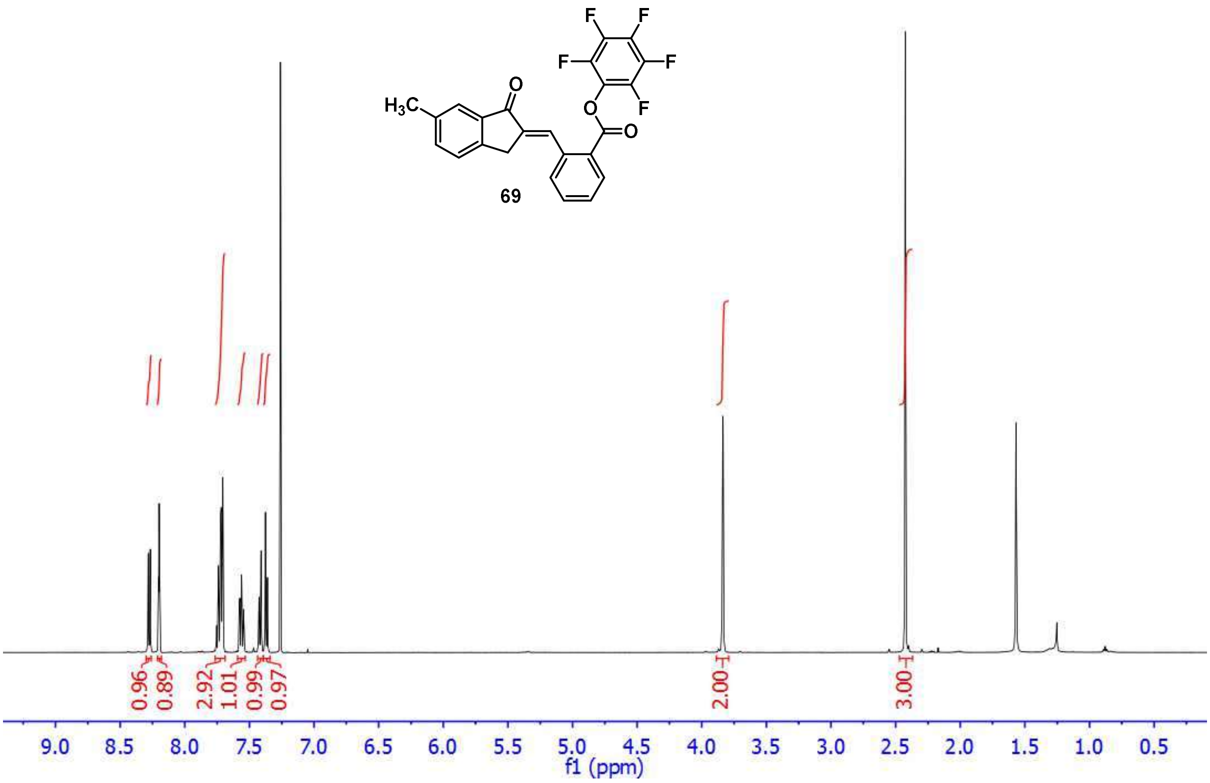

69
0.96
0.89
2.92
1.01
0.99
0.97
2.00
3.00
9.0 8.5 8.0 7.5 7.0 6.5 6.0 5.5 5.0 4.5 4.0 3.5 3.0 2.5 2.0 1.5 1.0 0.5
f1 (ppm)


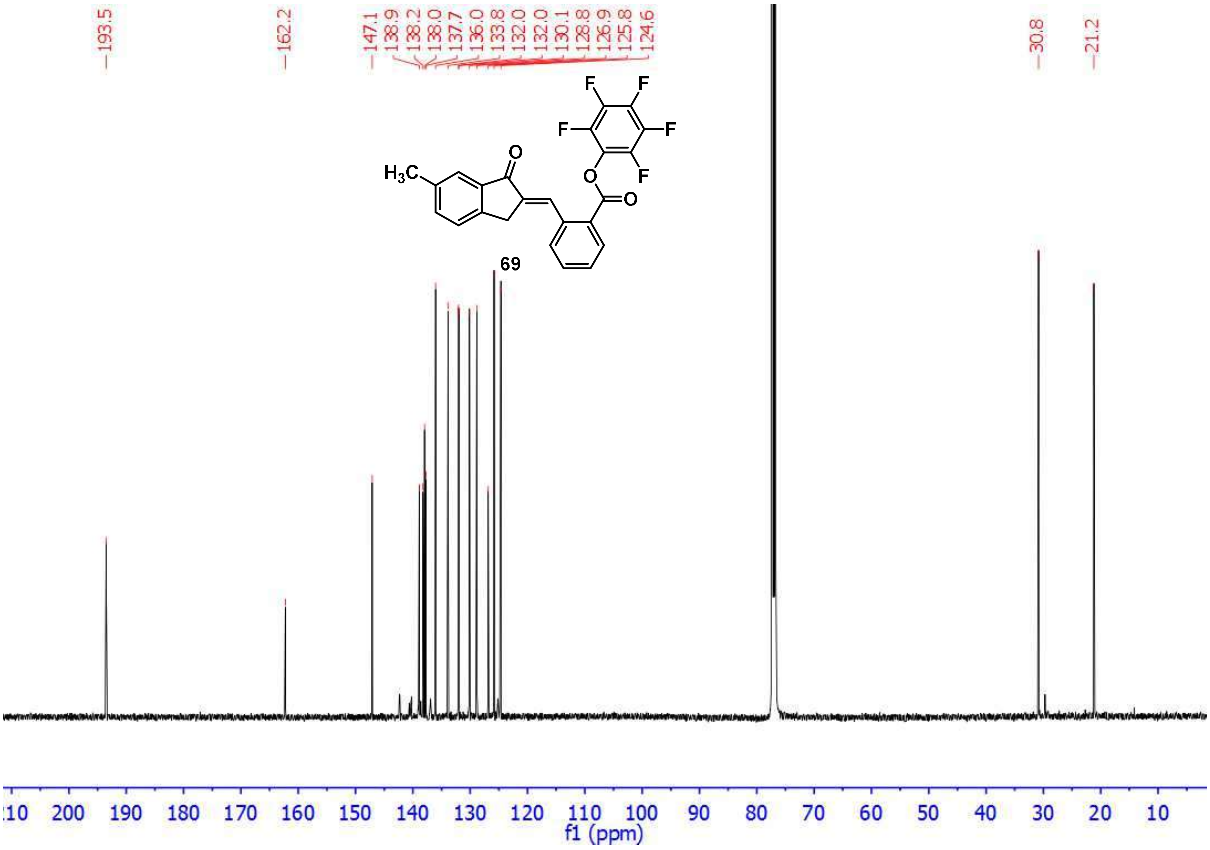

193.5
162.2
147.1
138.9
138.2
138.0
137.7
136.0
133.8
132.0
132.0
130.1
128.8
126.9
125.8
124.6
30.8
21.2
69
200 190 180 170 160 150 140 130 120 110 100 90 80 70 60 50 40 30 20 10
f1 (ppm)

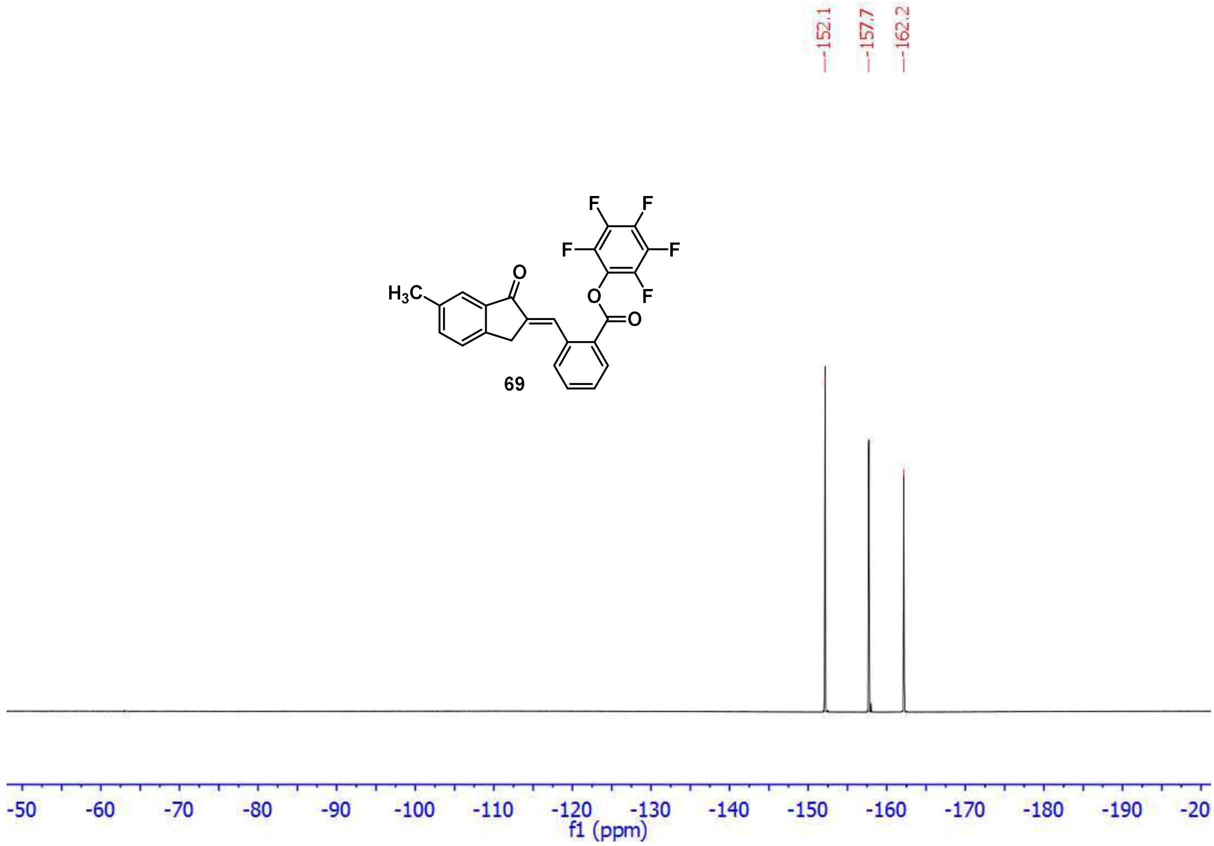

-152.1
-157.7
-162.2
69
-50 -60 -70 -80 -90 -100 -110 -120 -130 -140 -150 -160 -170 -180 -190 -20
f1 (ppm)


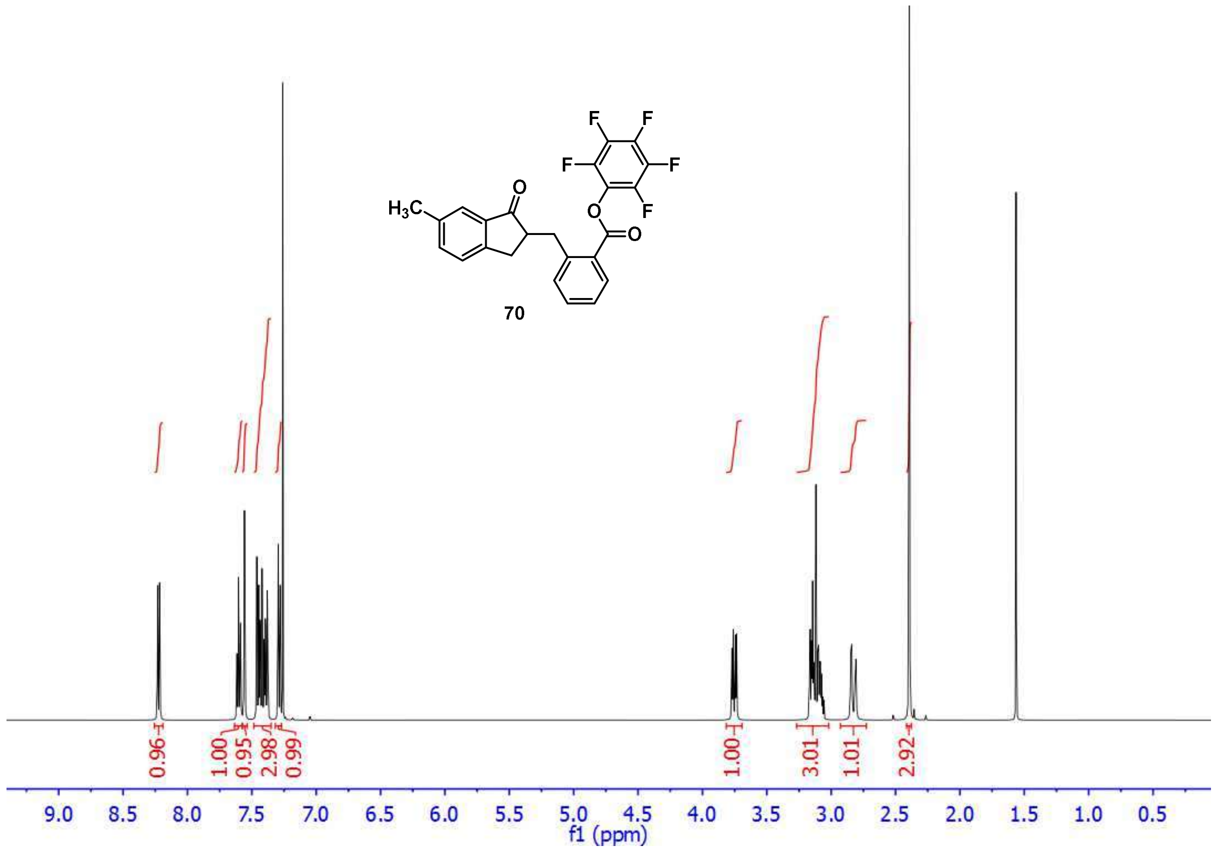

70
0.96
1.00
0.95
2.98
0.99
1.00
3.01
1.01
2.92
9.0 8.5 8.0 7.5 7.0 6.5 6.0 5.5 5.0 4.5 4.0 3.5 3.0 2.5 2.0 1.5 1.0 0.5
f1 (ppm)

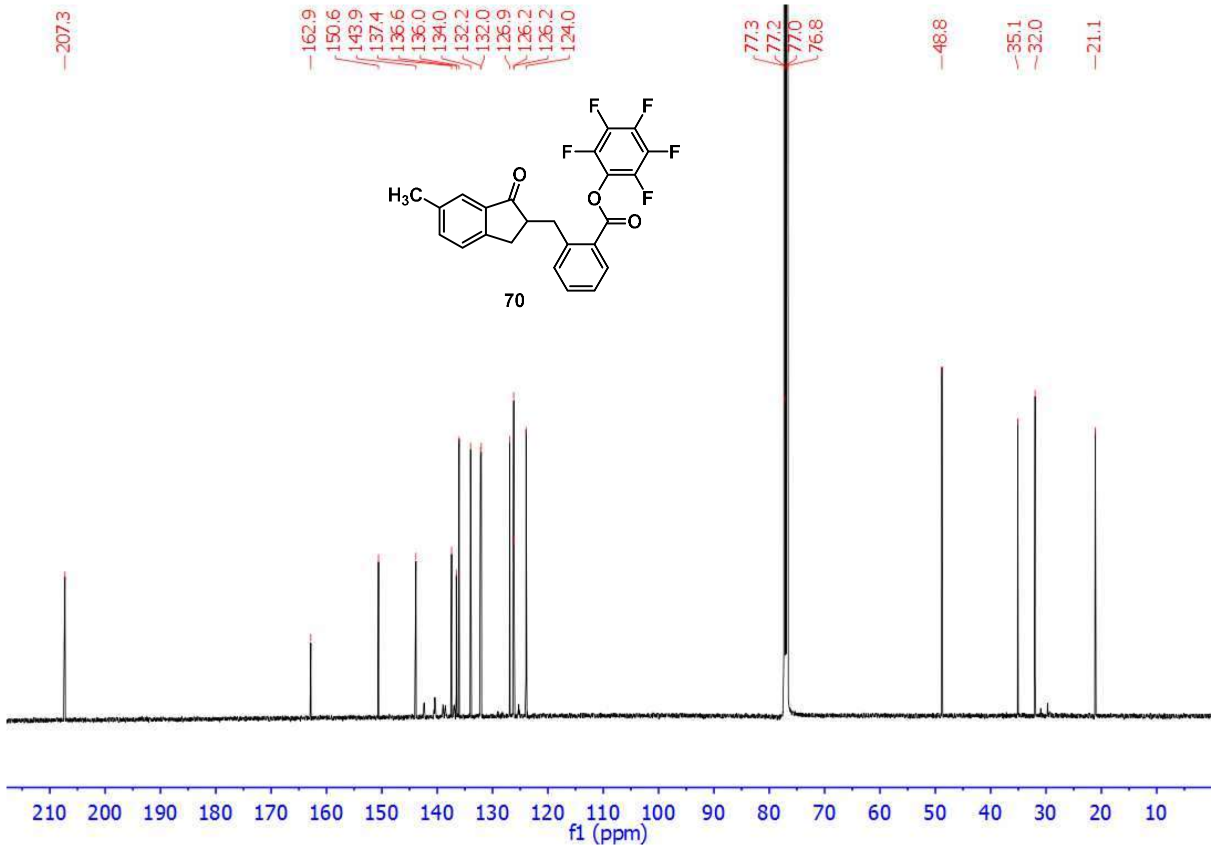
207.3
162.9
150.6
143.9
137.4
136.6
136.0
134.0
132.2
132.0
126.9
126.2
126.2
124.0
77.3
77.2
77.0
76.8
48.8
35.1
32.0
21.1
70
f1 (ppm)

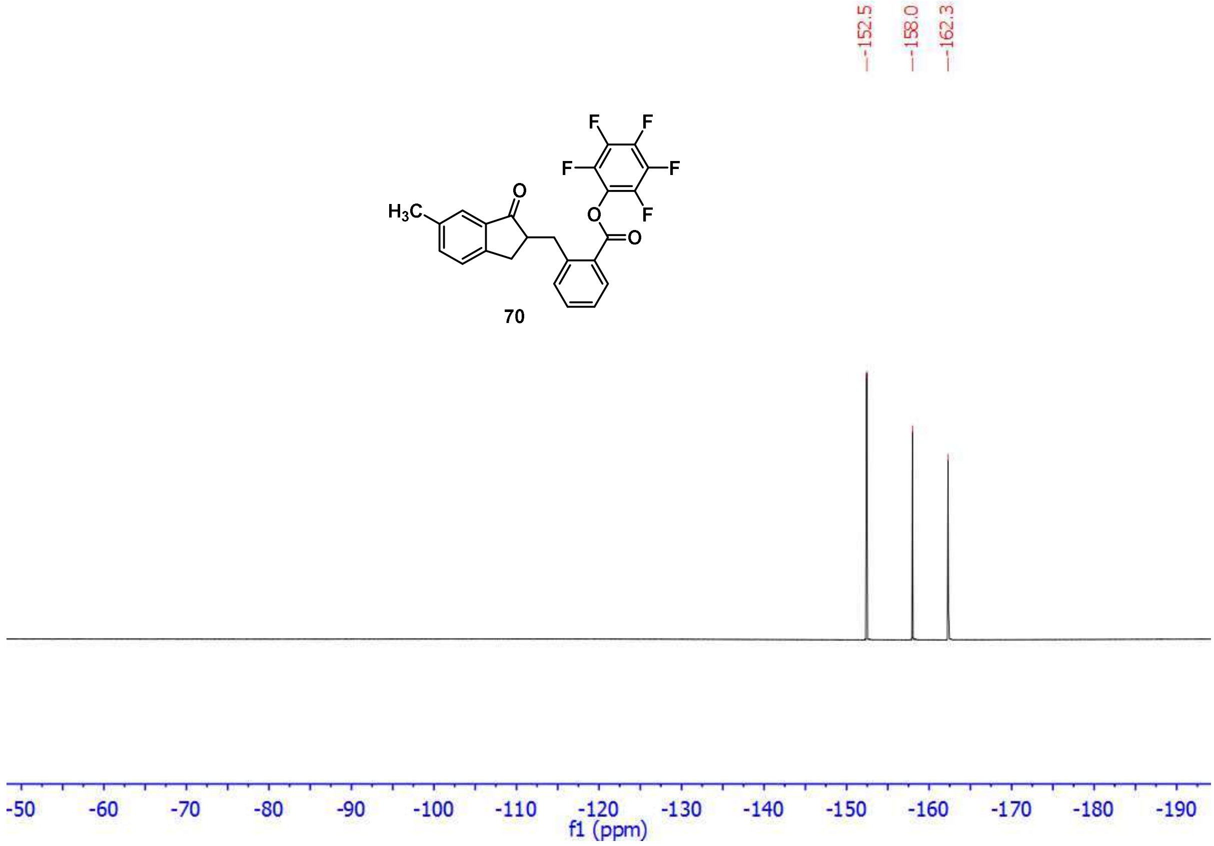
-152.5
-158.0
-162.3
70
f1 (ppm)

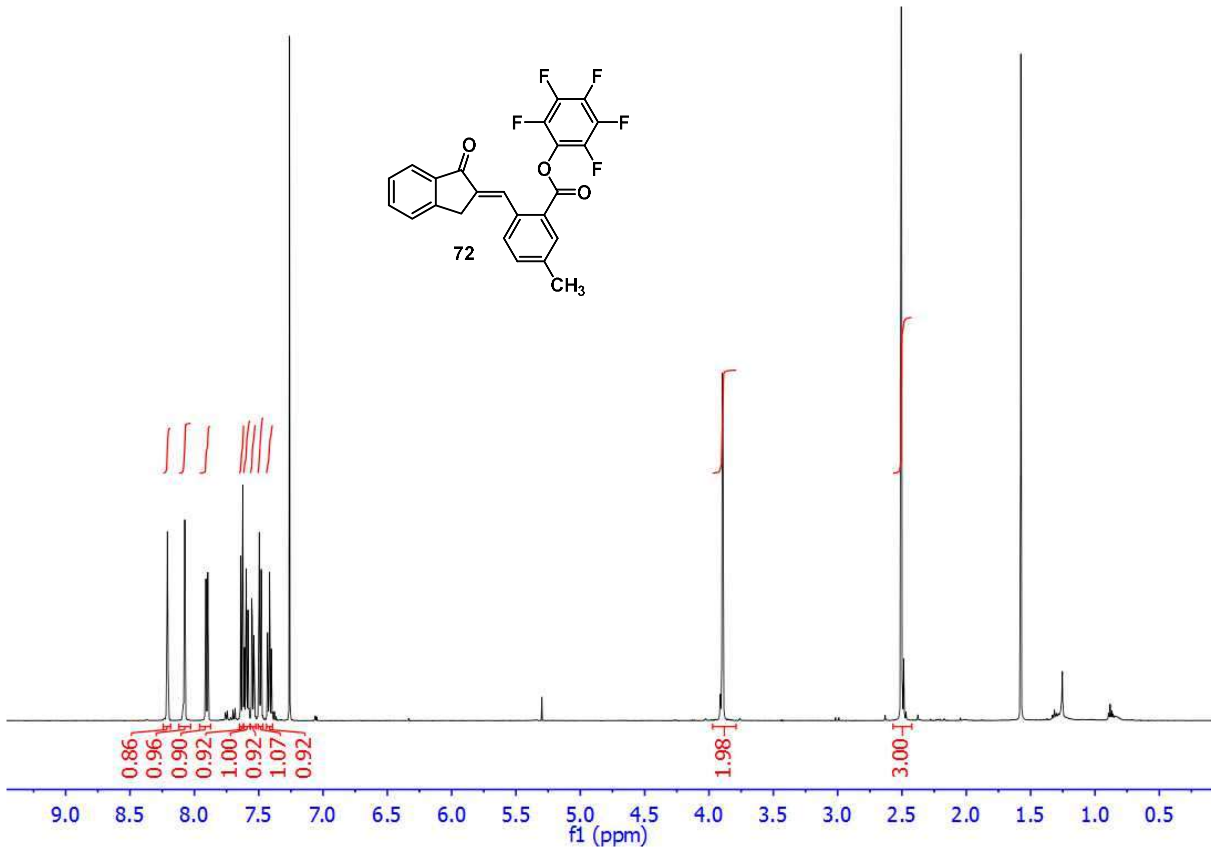

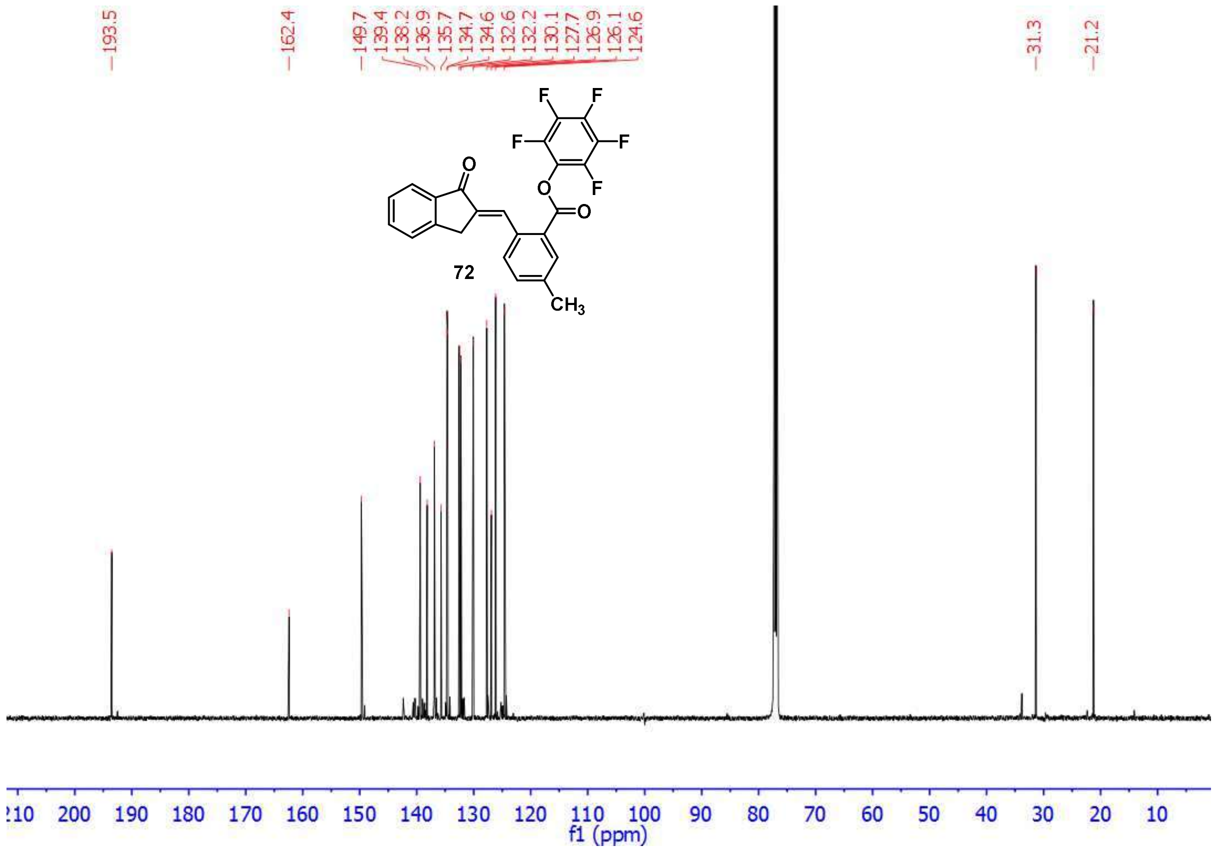

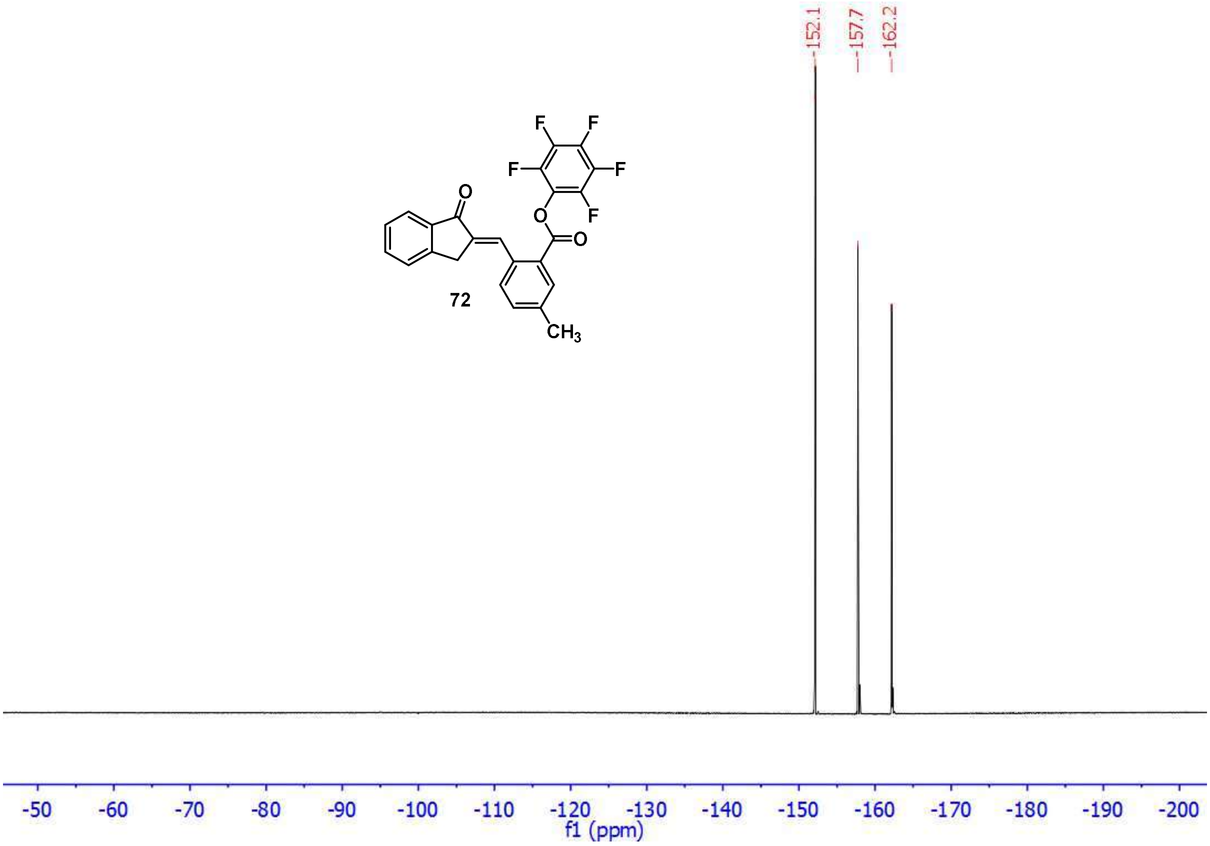
-152.1
-157.7
-162.2
72
-50 -60 -70 -80 -90 -100 -110 -120 -130 -140 -150 -160 -170 -180 -190 -200
f1 (ppm)

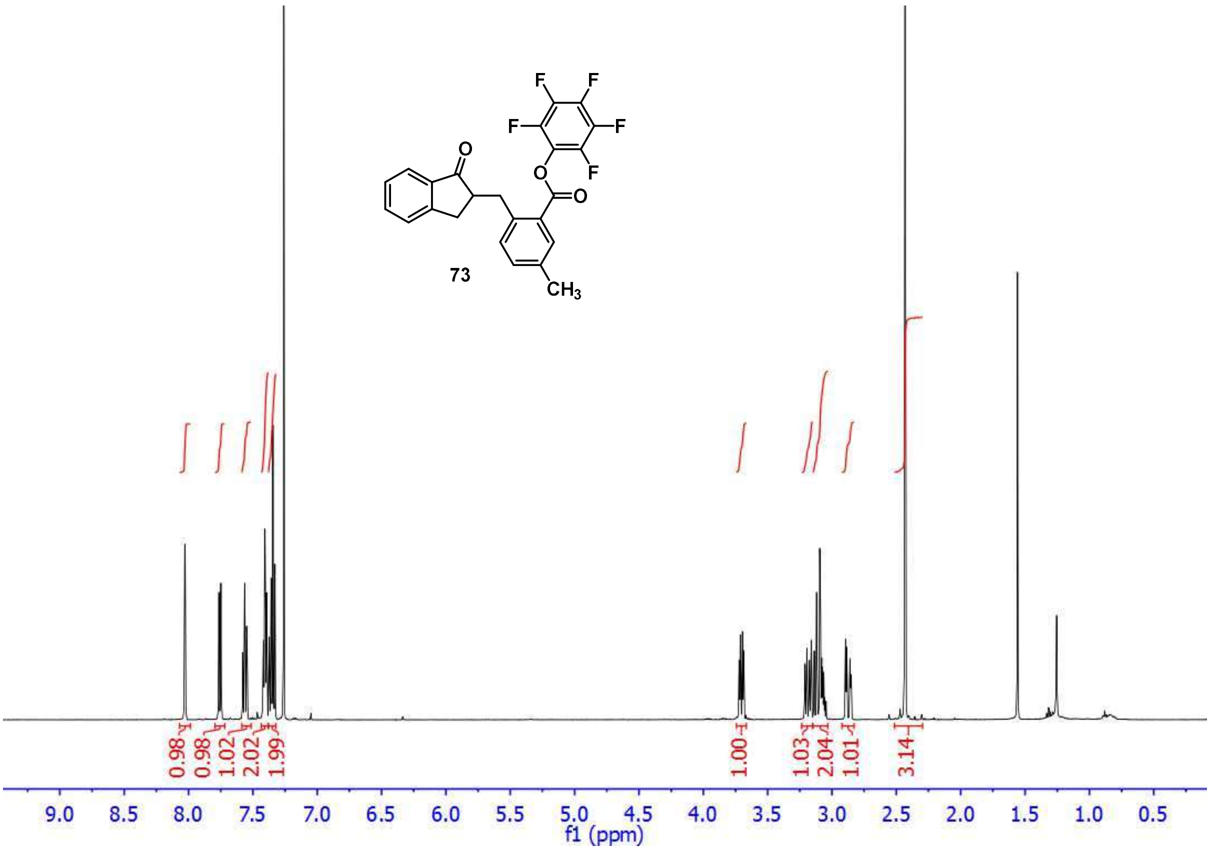
73
0.98
0.98
1.02
2.02
1.99
1.00
1.03
2.04
1.01
3.14
9.0 8.5 8.0 7.5 7.0 6.5 6.0 5.5 5.0 4.5 4.0 3.5 3.0 2.5 2.0 1.5 1.0 0.5
f1 (ppm)

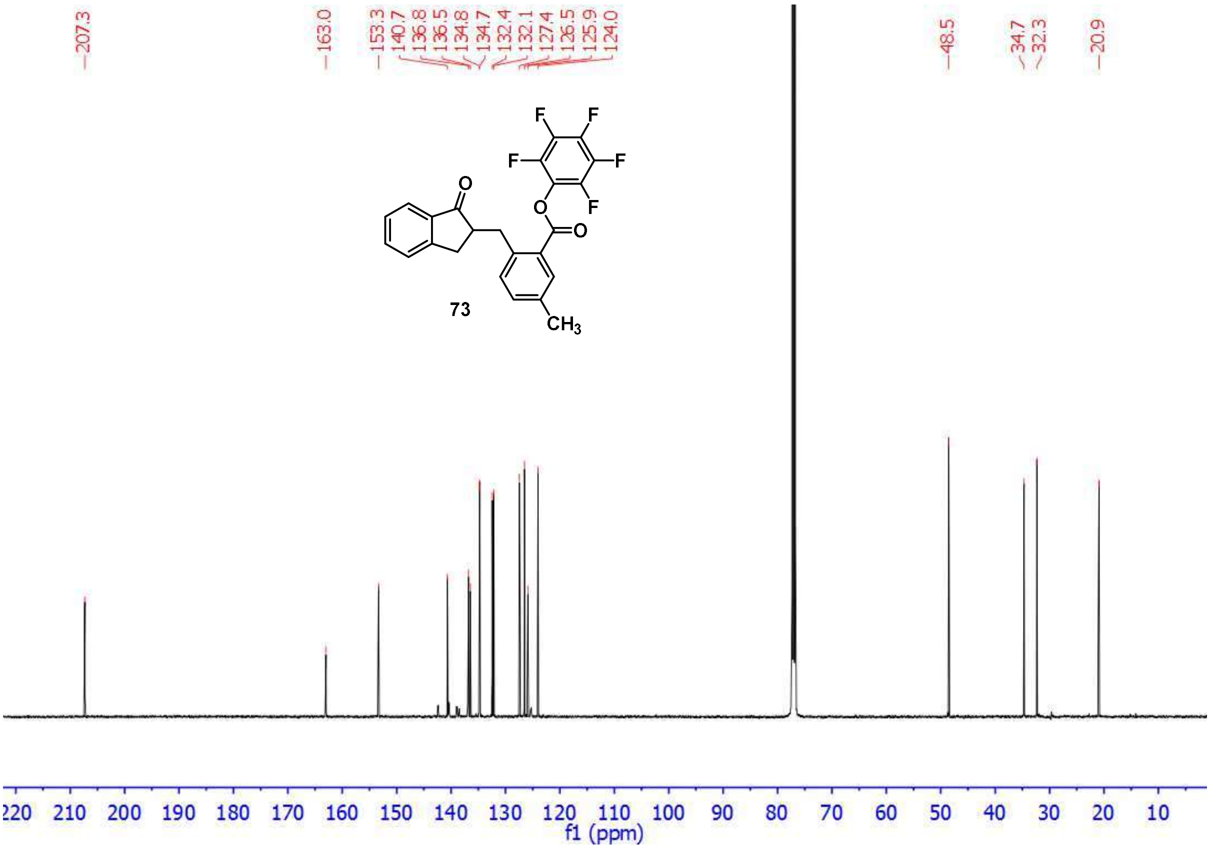
207.3
163.0
153.3
140.7
136.8
136.5
134.8
134.7
132.4
132.1
127.4
126.5
125.9
124.0
48.5
34.7
32.3
20.9
73
f1 (ppm)

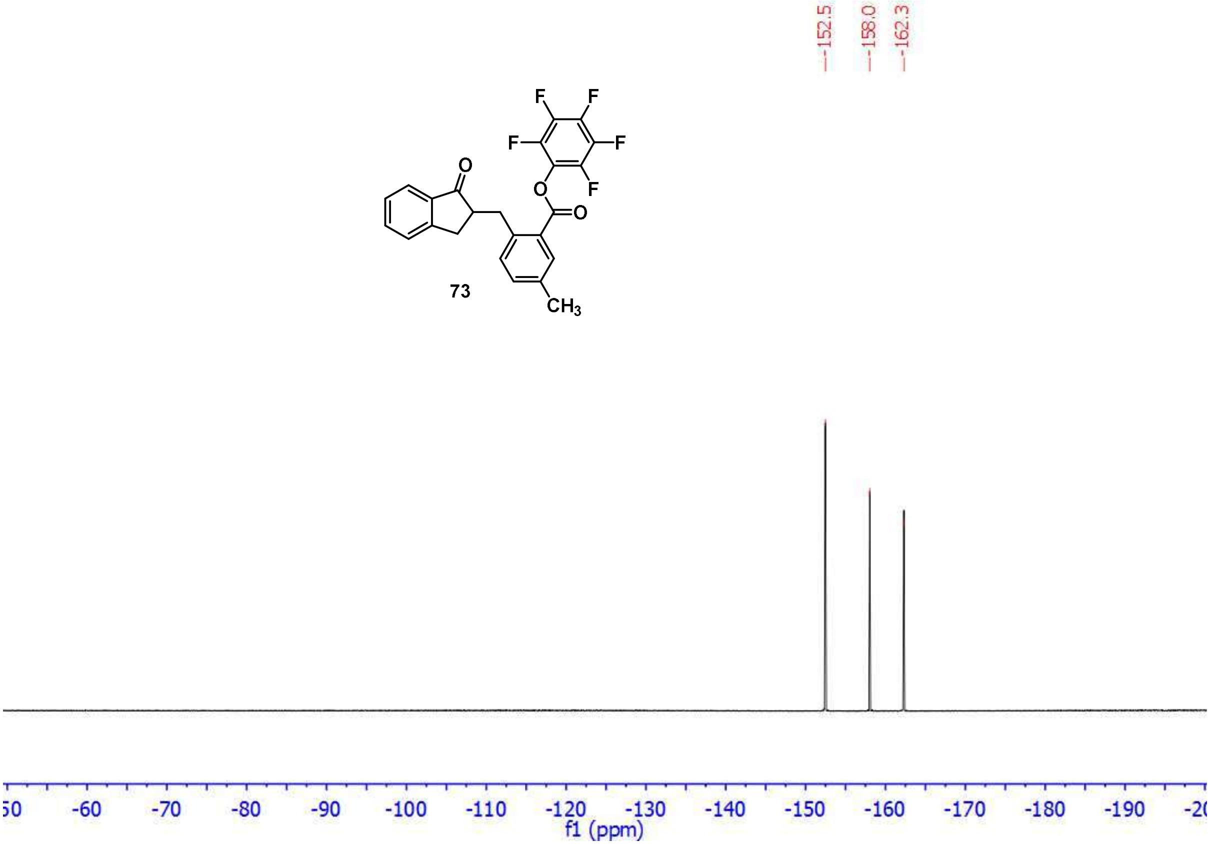
-152.5
-158.0
-162.3
73
f1 (ppm)

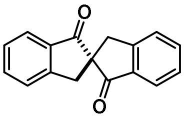

**9**

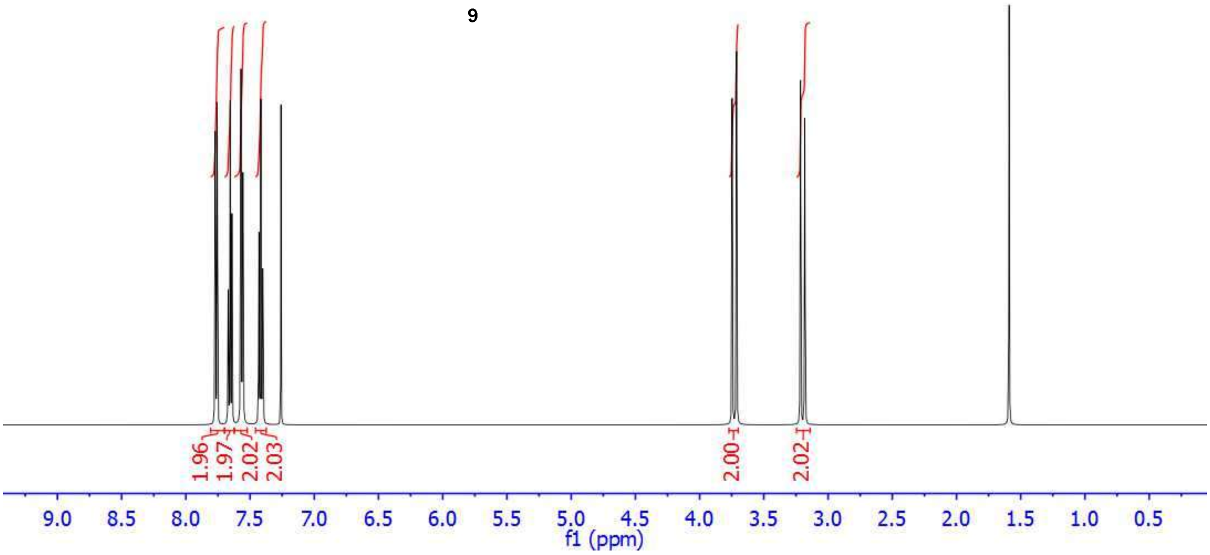

1.96
1.97
2.02
2.03
2.00
2.02
9.0 8.5 8.0 7.5 7.0 6.5 6.0 5.5 5.0 4.5 4.0 3.5 3.0 2.5 2.0 1.5 1.0 0.5
f1 (ppm)


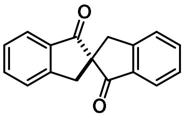

**9**

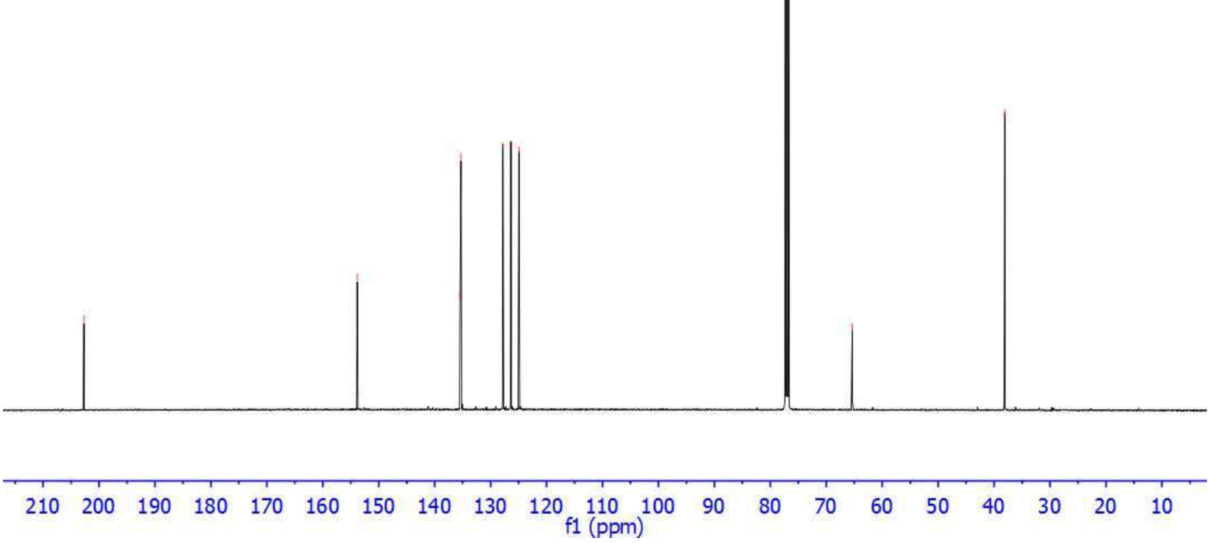

202.7
153.8
135.5
135.3
127.8
126.4
124.9
65.4
38.1
210 200 190 180 170 160 150 140 130 120 110 100 90 80 70 60 50 40 30 20 10
f1 (ppm)

**Chiral HPLC:** (Chiralpak ADH, 20% isopropanol, 80% hexane, 1.0 mL/min, λ = 250 nm) $\tau_R$ (minor) = 9.0 min, $\tau_R$ (major) = 15.3 min.

## Asymmetric

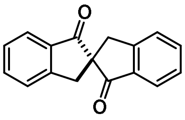

**9**

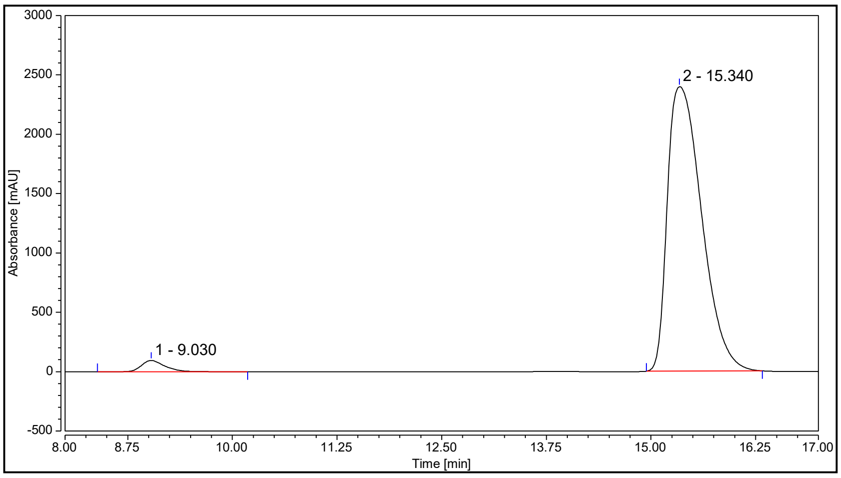


| No. | Peak Name | Retention Time | Area | Height | Relative Area |
|---|---|---|---|---|---|
| | | min | mAU*min | mAU | % |
| 1 | | 9.030 | 30.275 | 94.499 | 2.59 |
| 2 | | 15.340 | 1139.529 | 2395.900 | 97.41 |
| **Total:** | | | **1169.804** | **2490.399** | **100.00** |

## Asymmetric (gram scale)

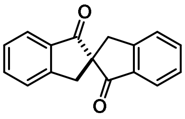

**9**

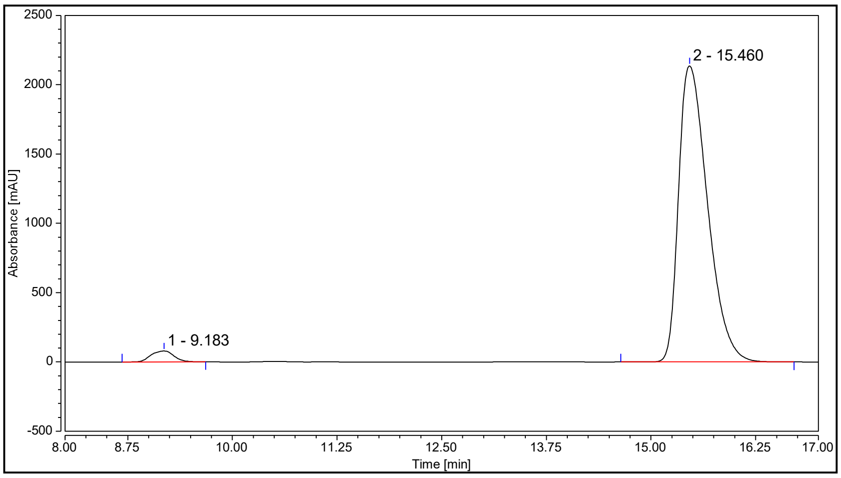


| No. | Peak Name | Retention Time | Area | Height | Relative Area |
|---|---|---|---|---|---|
| | | min | mAU*min | mAU | % |
| 1 | | 9.183 | 24.843 | 78.783 | 2.82 |
| 2 | | 15.460 | 856.897 | 2135.931 | 97.18 |
| **Total:** | | | **881.741** | **2214.714** | **100.00** |

## Racemic

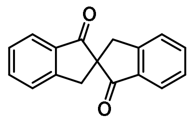

(*rac*)-**9**

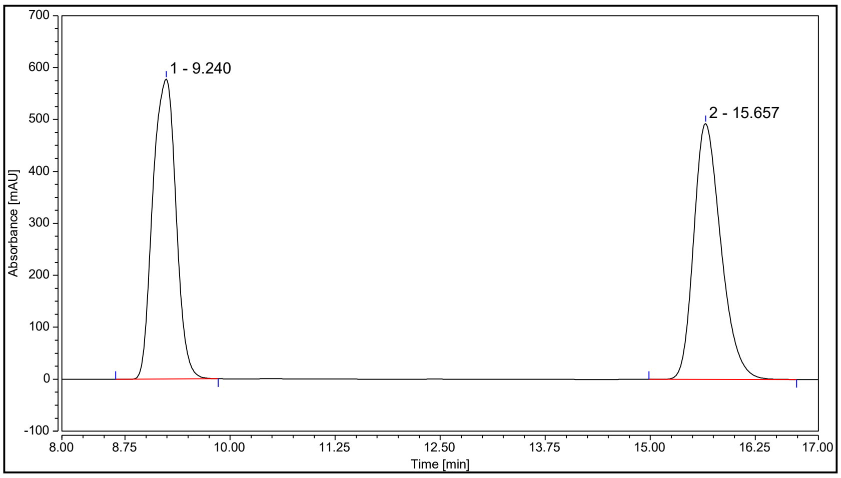


| No. | Peak Name | Retention Time | Area | Height | Relative Area |
|---|---|---|---|---|---|
| | | min | mAU*min | mAU | % |
| 1 | | 9.240 | 184.531 | 577.256 | 49.97 |
| 2 | | 15.657 | 184.729 | 492.536 | 50.03 |
| **Total:** | | | **369.260** | **1069.792** | **100.00** |

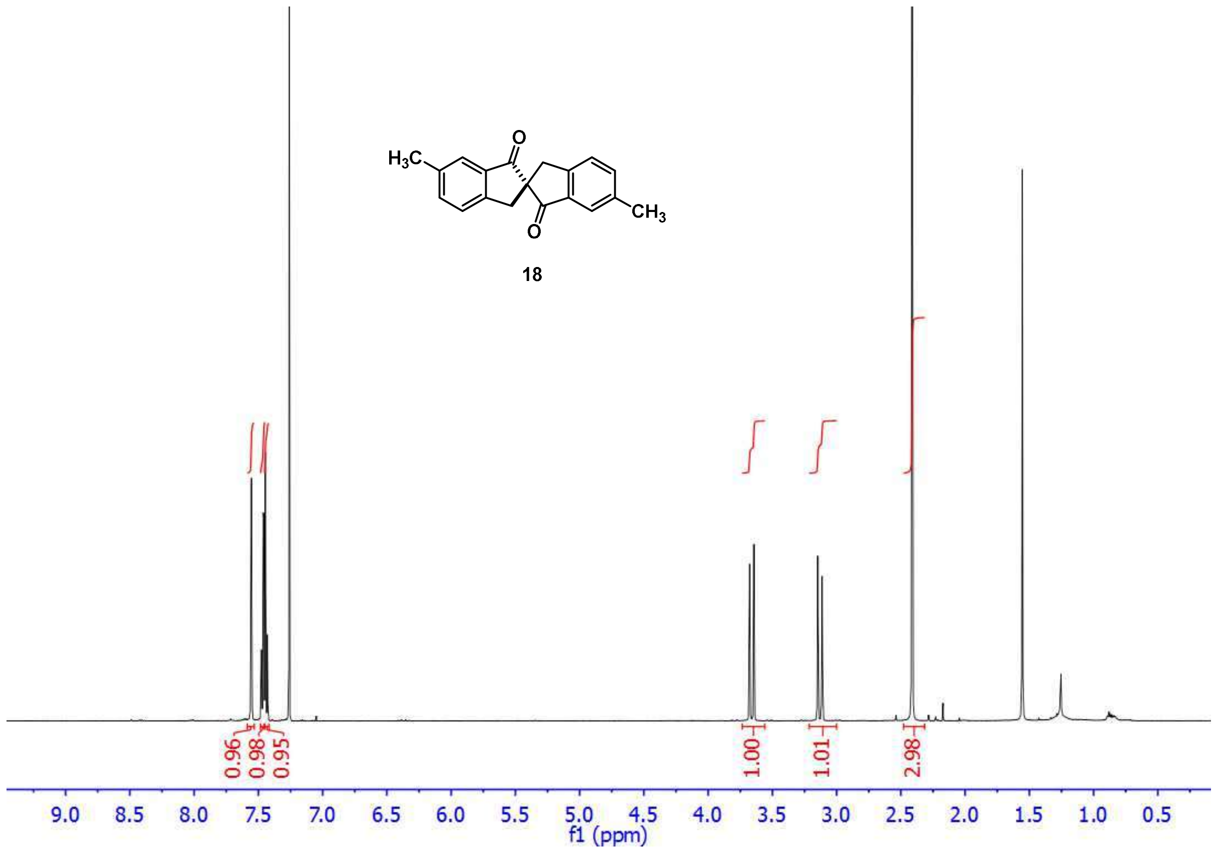
H3C
O
O
CH3
18
0.96
0.98
0.95
1.00
1.01
2.98
9.0 8.5 8.0 7.5 7.0 6.5 6.0 5.5 5.0 4.5 4.0 3.5 3.0 2.5 2.0 1.5 1.0 0.5
f1 (ppm)

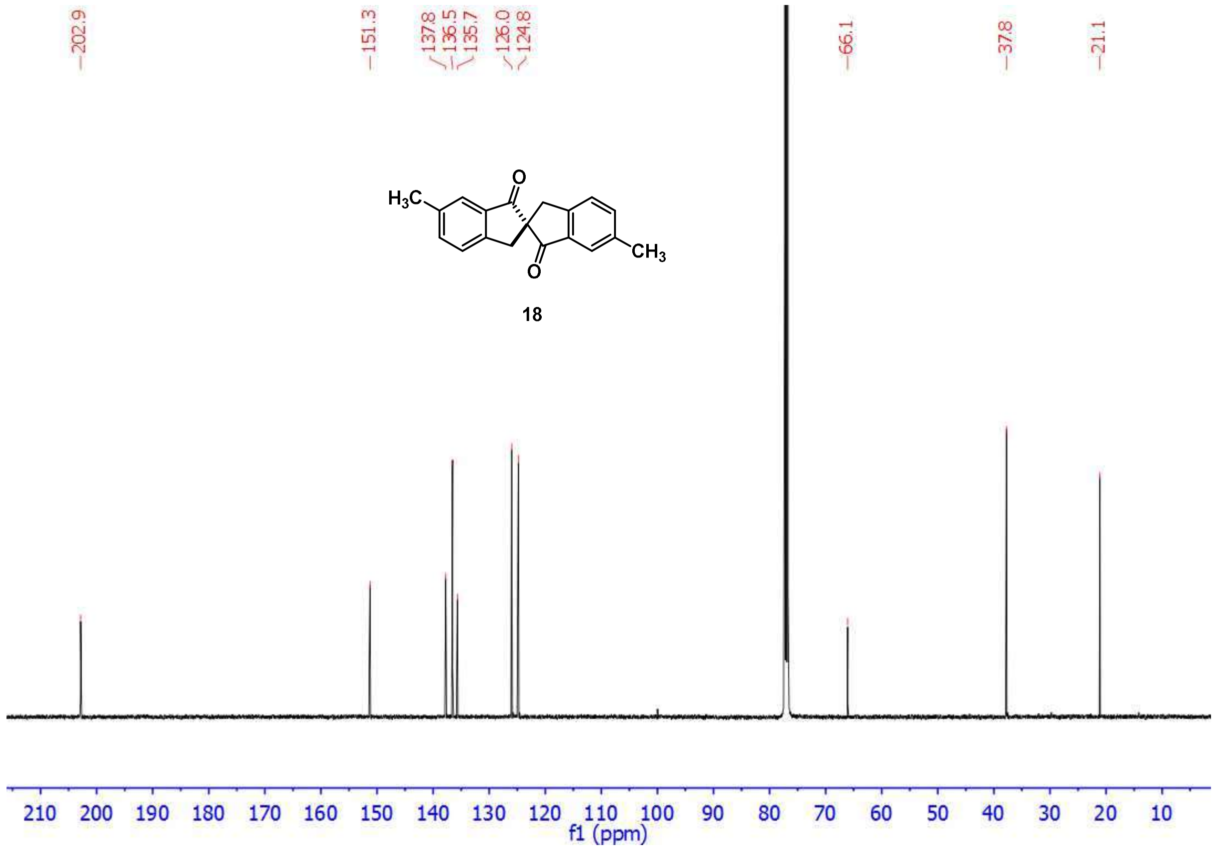
202.9
151.3
137.8
136.5
135.7
126.0
124.8
66.1
37.8
21.1
H3C
O
O
CH3
18
210 200 190 180 170 160 150 140 130 120 110 100 90 80 70 60 50 40 30 20 10
f1 (ppm)

**Chiral HPLC:** (Chiralpak ADH, 20% isopropanol, 80% hexane, 1.0 mL/min, λ = 299 nm) $\tau_R$ (minor) = 7.7 min, $\tau_R$ (major) = 24.8 min.

## Asymmetric

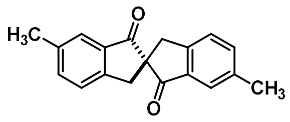


**18**

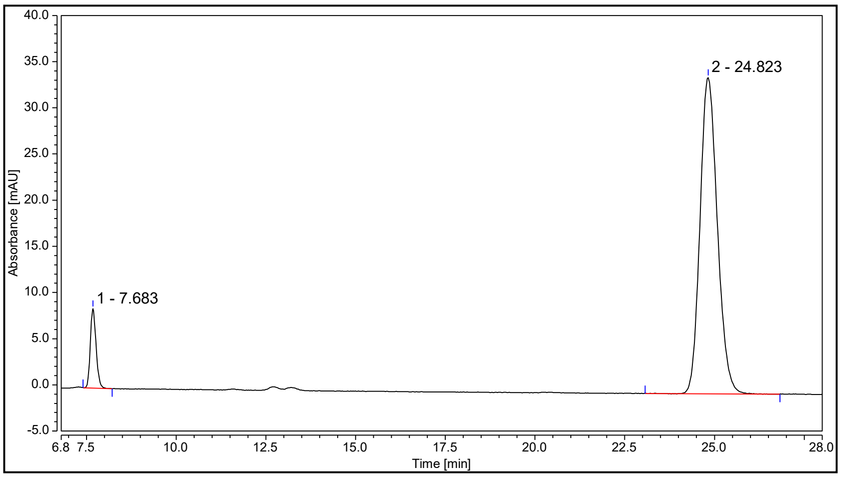


| No. | Peak Name | Retention Time | Area | Height | Relative Area |
|---|---|---|---|---|---|
| | | min | mAU*min | mAU | % |
| 1 | | 7.683 | 1.616 | 8.597 | 7.77 |
| 2 | | 24.823 | 19.167 | 34.253 | 92.23 |
| **Total:** | | | **20.783** | **42.850** | **100.00** |

## Racemic

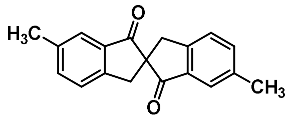


(*rac*)-**18**

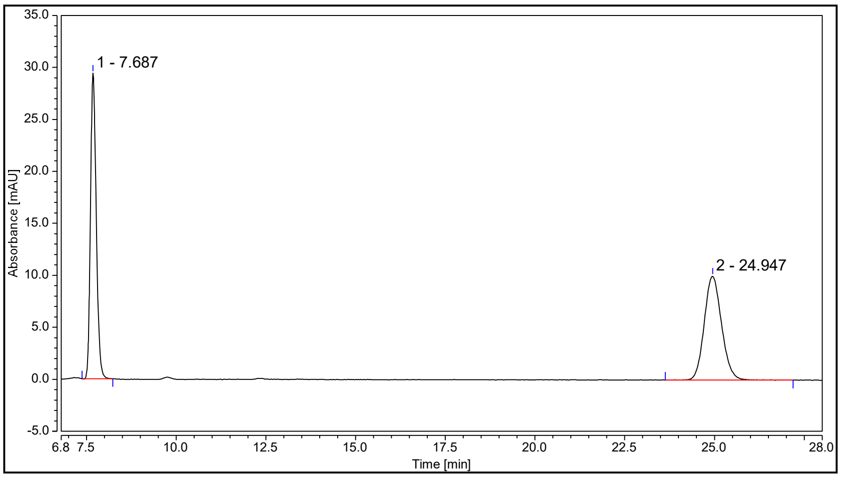


| No. | Peak Name | Retention Time | Area | Height | Relative Area |
|---|---|---|---|---|---|
| | | min | mAU*min | mAU | % |
| 1 | | 7.687 | 5.502 | 29.402 | 49.68 |
| 2 | | 24.947 | 5.573 | 9.992 | 50.32 |
| **Total:** | | | **11.075** | **39.395** | **100.00** |

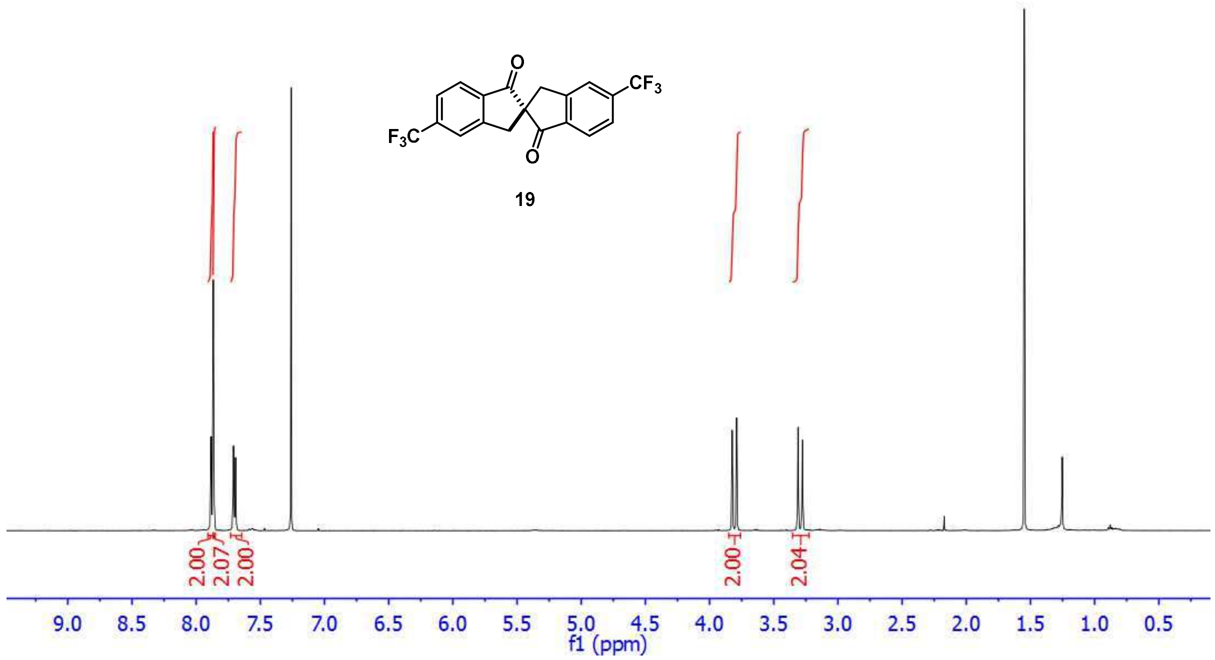

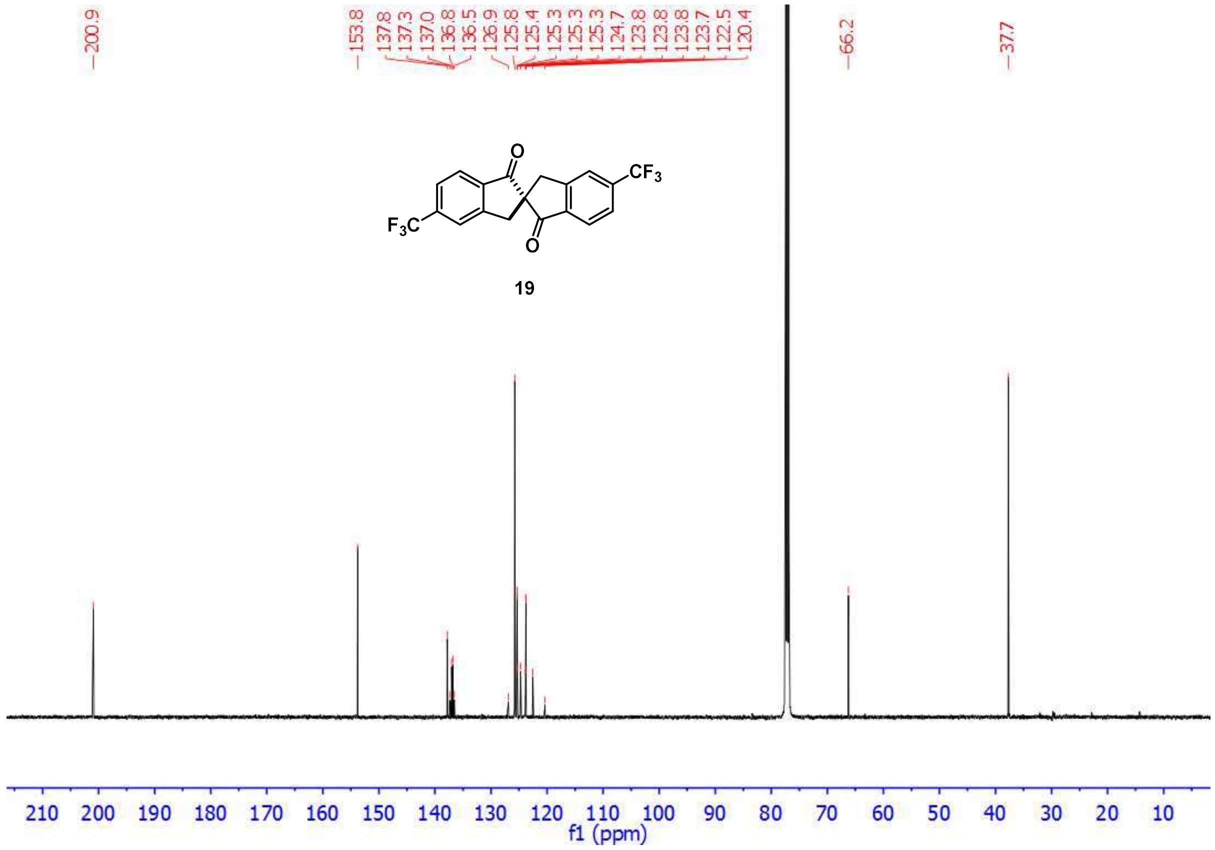

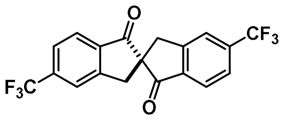
O
CF3
F3C
O

19

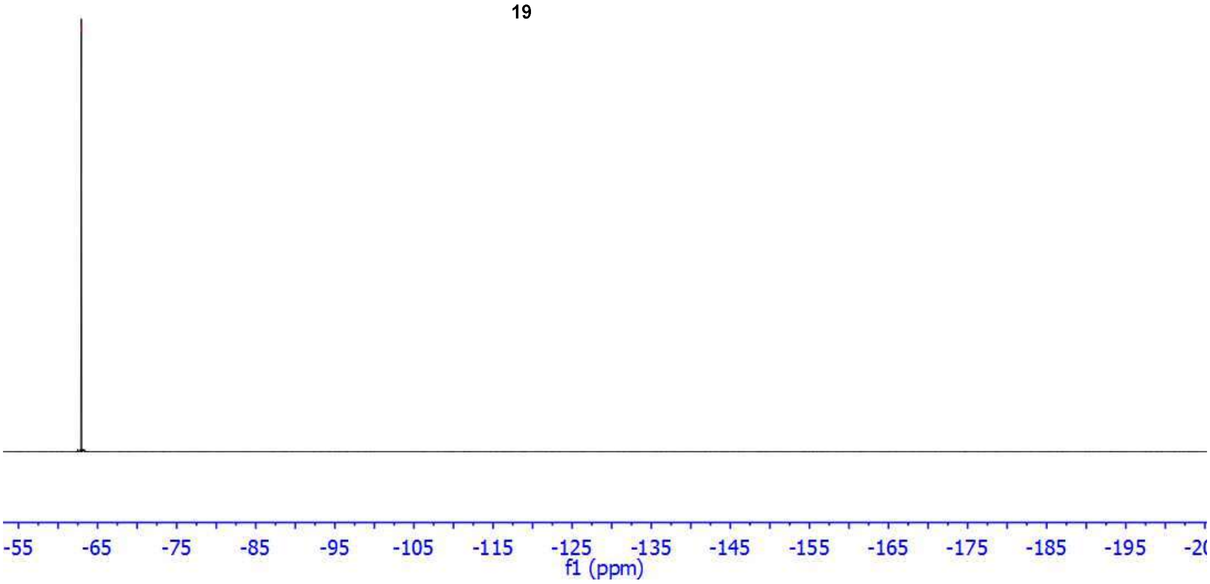
-63.0
-55
-65
-75
-85
-95
-105
-115
-125
-135
-145
-155
-165
-175
-185
-195
f1 (ppm)

**Chiral HPLC:** (Chiralpak ADH, 2% isopropanol, 98% hexane, 1.0 mL/min, λ = 250 nm) $\tau_R$ (minor) = 20.7 min, $\tau_R$ (major) = 23.0 min.

**Asymmetric**

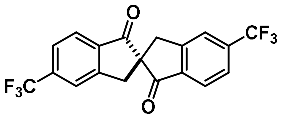

19

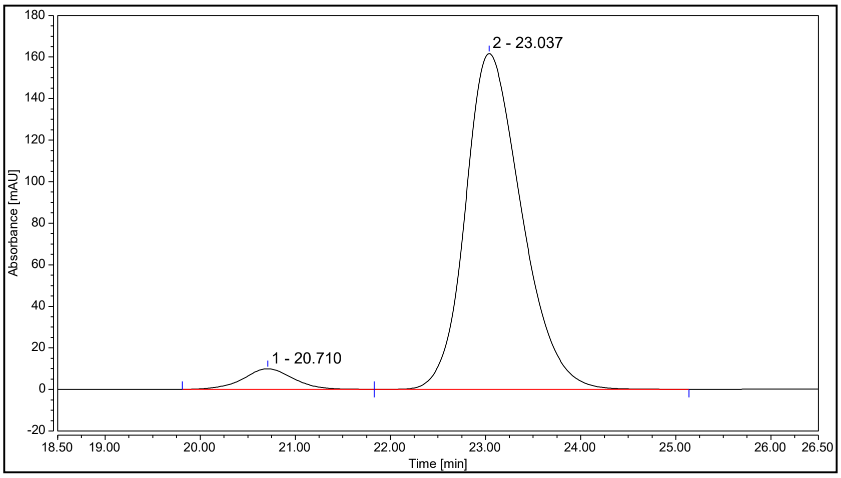


| No. | Peak Name | Retention Time | Area | Height | Relative Area |
|---|---|---|---|---|---|
| | | min | mAU*min | mAU | % |
| 1 | | 20.710 | 6.027 | 9.896 | 5.17 |
| 2 | | 23.037 | 110.648 | 161.576 | 94.83 |
| **Total:** | | | **116.675** | **171.472** | **100.00** |

**Racemic**

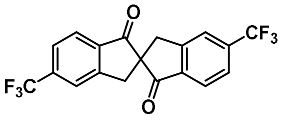

(*rac*)-19

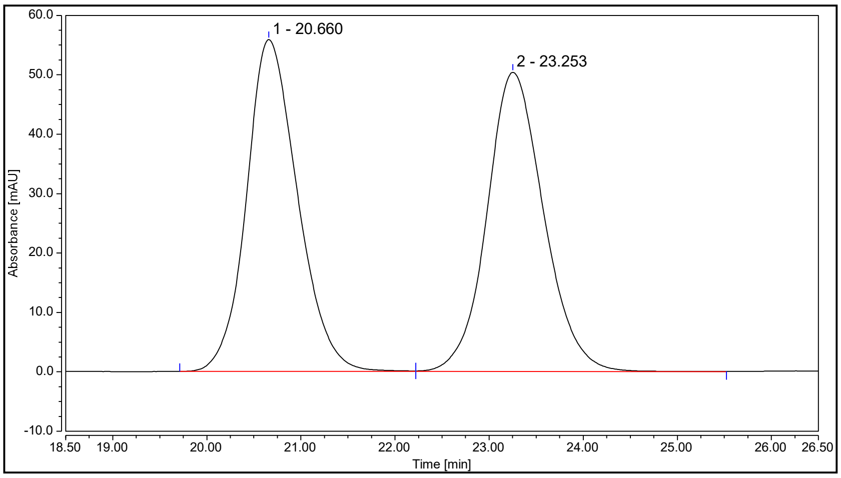


| No. | Peak Name | Retention Time | Area | Height | Relative Area |
|---|---|---|---|---|---|
| | | min | mAU*min | mAU | % |
| 1 | | 20.660 | 35.159 | 55.880 | 50.00 |
| 2 | | 23.253 | 35.157 | 50.378 | 50.00 |
| **Total:** | | | **70.316** | **106.258** | **100.00** |

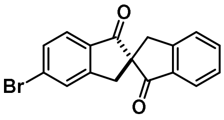

**20**

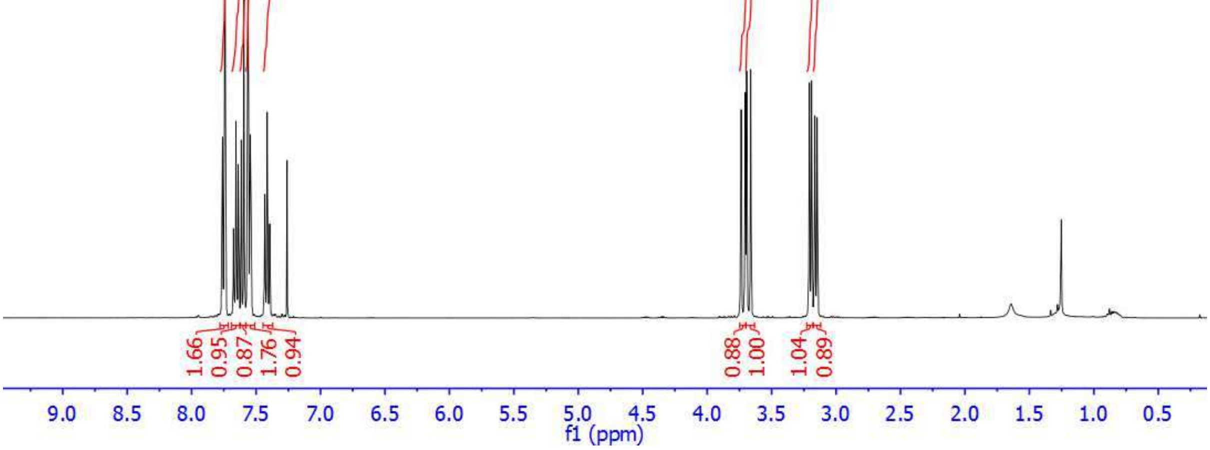

1.66
0.95
0.87
1.76
0.94
0.88
1.00
1.04
0.89
9.0 8.5 8.0 7.5 7.0 6.5 6.0 5.5 5.0 4.5 4.0 3.5 3.0 2.5 2.0 1.5 1.0 0.5
f1 (ppm)


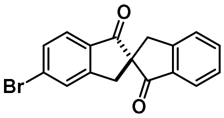

**20**

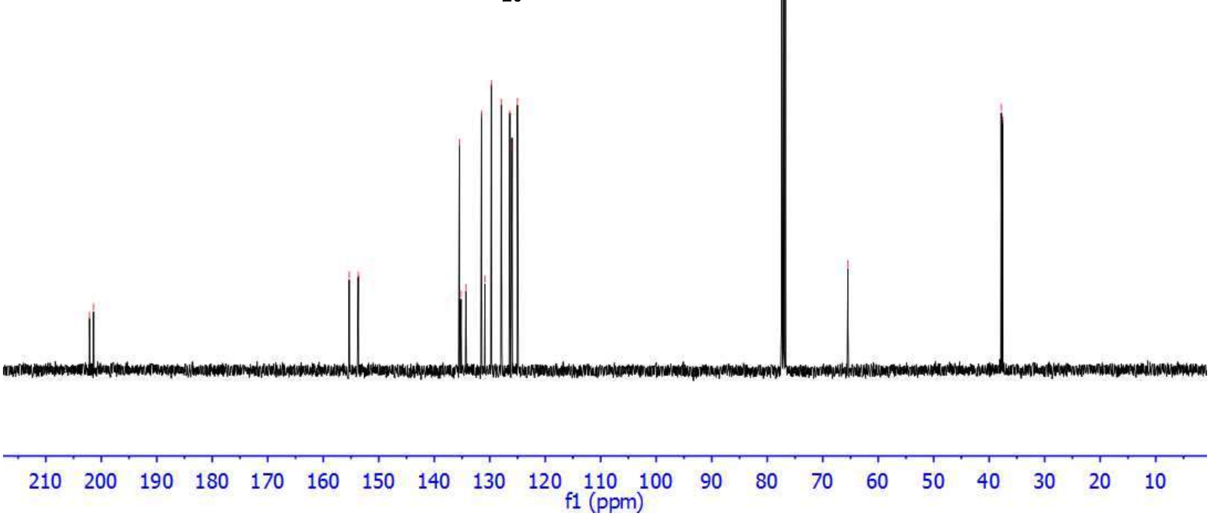

202.1
201.4
155.3
153.7
135.5
135.2
134.3
131.5
130.9
129.7
127.9
126.4
126.0
125.0
65.4
37.8
37.6
210 200 190 180 170 160 150 140 130 120 110 100 90 80 70 60 50 40 30 20 10
f1 (ppm)

**Chiral HPLC:** (Chiralpak ADH, 20% isopropanol, 80% hexane, 1.0 mL/min, λ = 250 nm) $\tau_R$ (minor) = 10.7 min, $\tau_R$ (major) = 13.0 min.

## Asymmetric

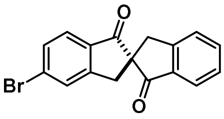


**20**

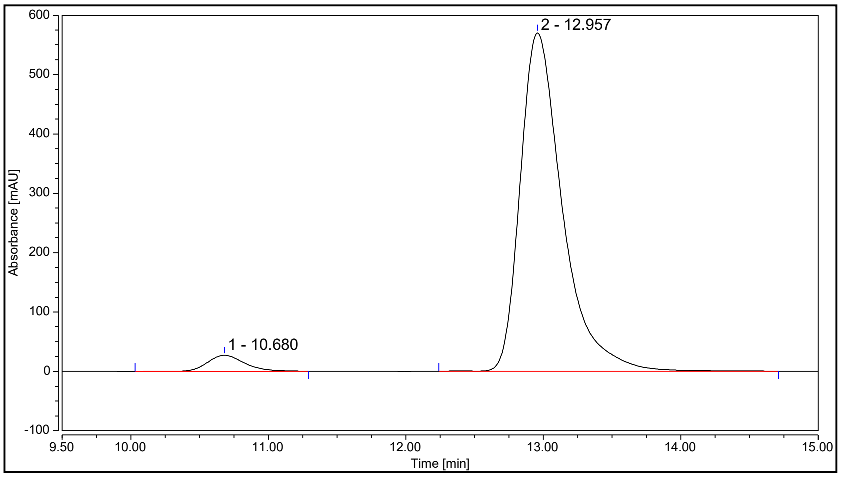


| No. | Peak Name | Retention Time | Area | Height | Relative Area |
|---|---|---|---|---|---|
| | | min | mAU*min | mAU | % |
| 1 | | 10.680 | 8.321 | 26.873 | 3.95 |
| 2 | | 12.957 | 202.563 | 570.137 | 96.05 |
| **Total:** | | | **210.884** | **597.010** | **100.00** |

## Asymmetric (2)

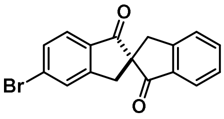


**20**

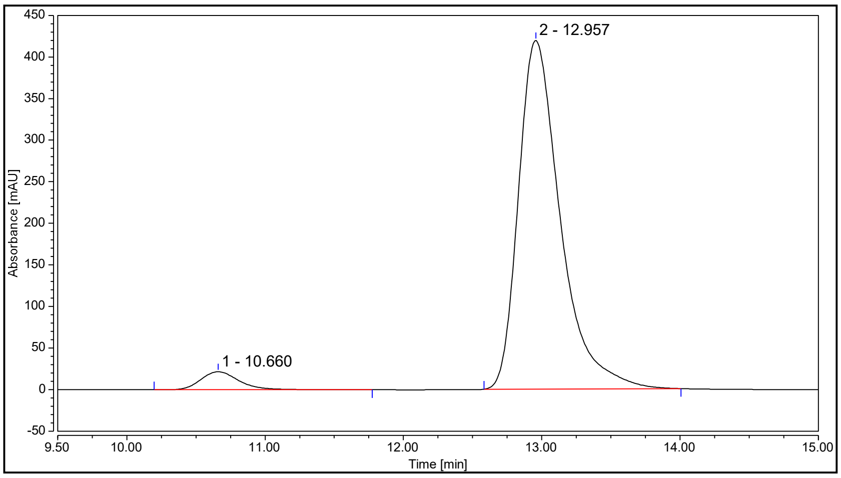


| No. | Peak Name | Retention Time | Area | Height | Relative Area |
|---|---|---|---|---|---|
| | | min | mAU*min | mAU | % |
| 1 | | 10.660 | 6.985 | 21.777 | 4.53 |
| 2 | | 12.957 | 147.086 | 419.163 | 95.47 |
| **Total:** | | | **154.071** | **440.940** | **100.00** |

## Racemic

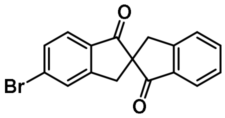


(*rac*)-**20**

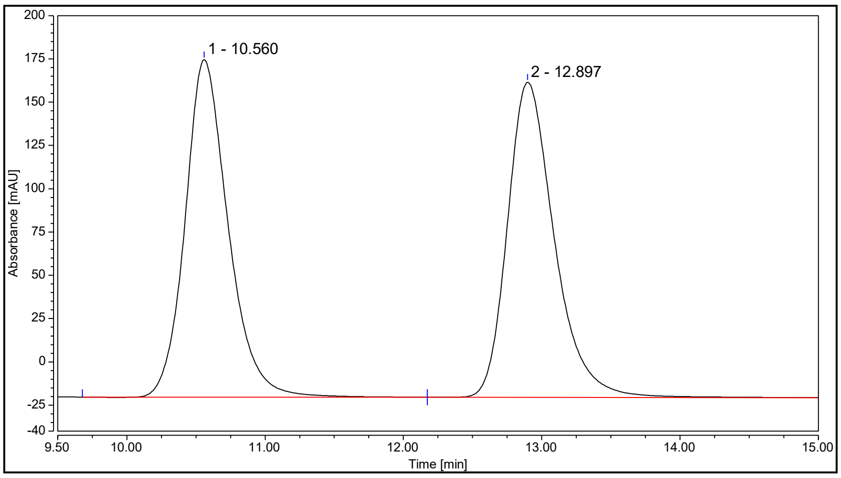


| No. | Peak Name | Retention Time | Area | Height | Relative Area |
|---|---|---|---|---|---|
| | | min | mAU*min | mAU | % |
| 1 | | 10.560 | 70.326 | 194.956 | 49.55 |
| 2 | | 12.897 | 71.592 | 182.035 | 50.45 |
| **Total:** | | | **141.919** | **376.991** | **100.00** |

## Single Crystal used for Absolute Configuration Determination

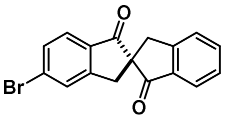


**20**

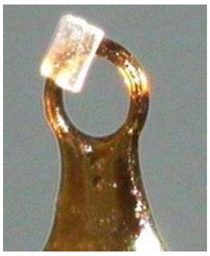

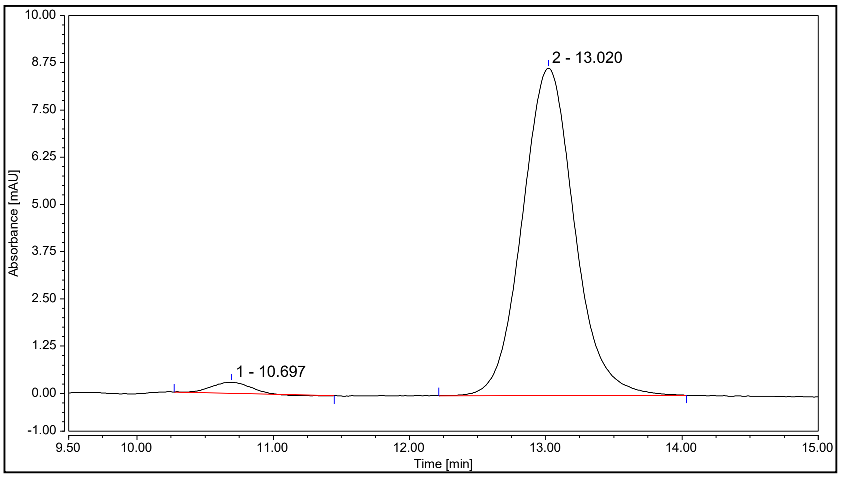


| No. | Peak Name | Retention Time | Area | Height | Relative Area |
|---|---|---|---|---|---|
| | | min | mAU*min | mAU | % |
| 1 | | 10.697 | 0.091 | 0.293 | 2.32 |
| 2 | | 13.020 | 3.834 | 8.664 | 97.68 |
| **Total:** | | | **3.925** | **8.957** | **100.00** |

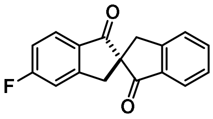

O
F
O


**21**

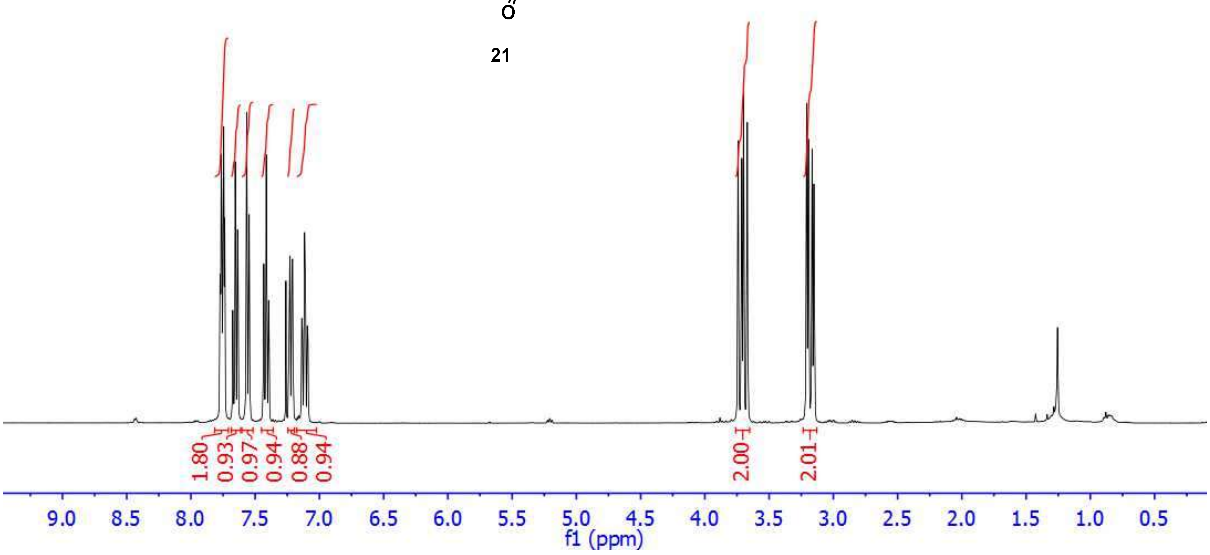

1.80
0.93
0.97
0.94
0.88
0.94
2.00
2.01
9.0 8.5 8.0 7.5 7.0 6.5 6.0 5.5 5.0 4.5 4.0 3.5 3.0 2.5 2.0 1.5 1.0 0.5
f1 (ppm)


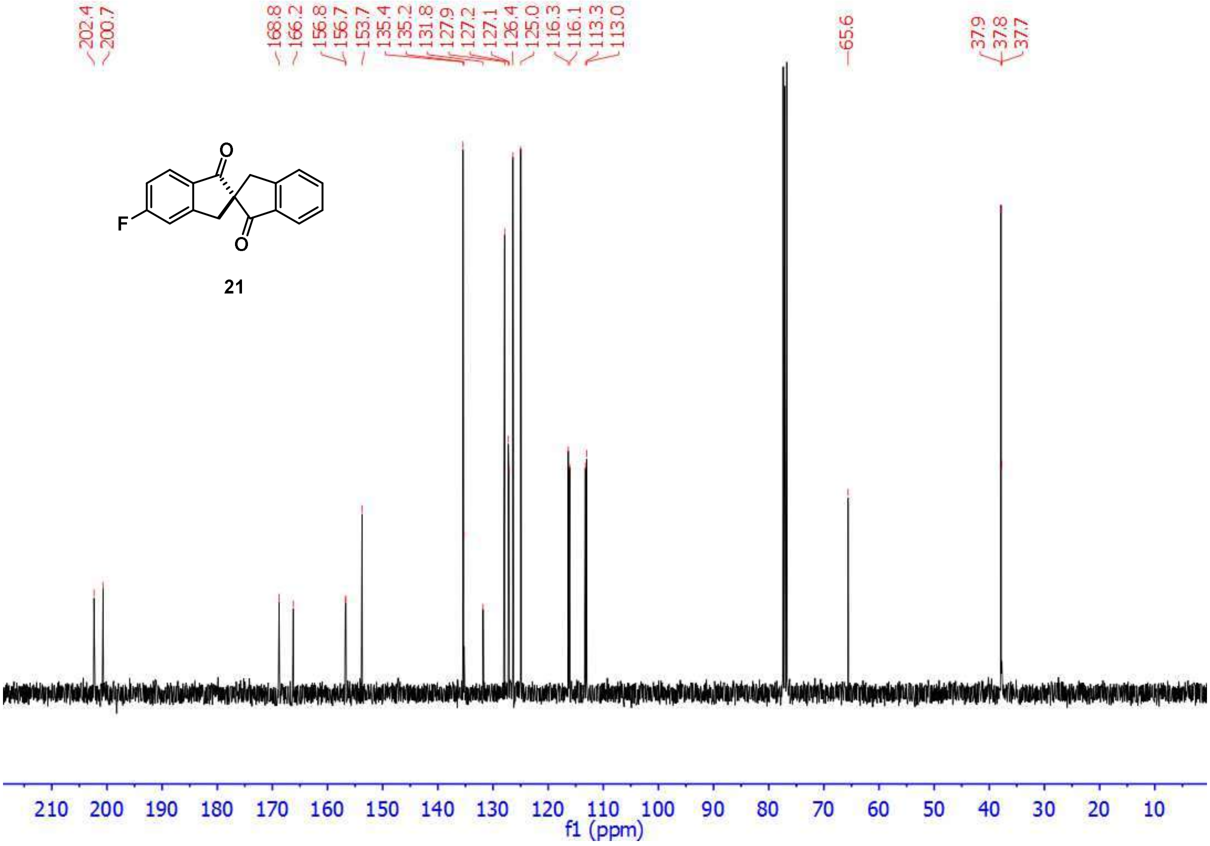

202.4
200.7
168.8
166.2
156.8
156.7
153.7
135.4
135.2
131.8
127.9
127.2
127.1
126.4
125.0
116.3
116.1
113.3
113.0
65.6
37.9
37.8
37.7
O
F
O
21
210 200 190 180 170 160 150 140 130 120 110 100 90 80 70 60 50 40 30 20 10
f1 (ppm)

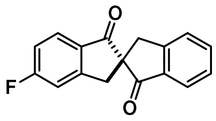

21

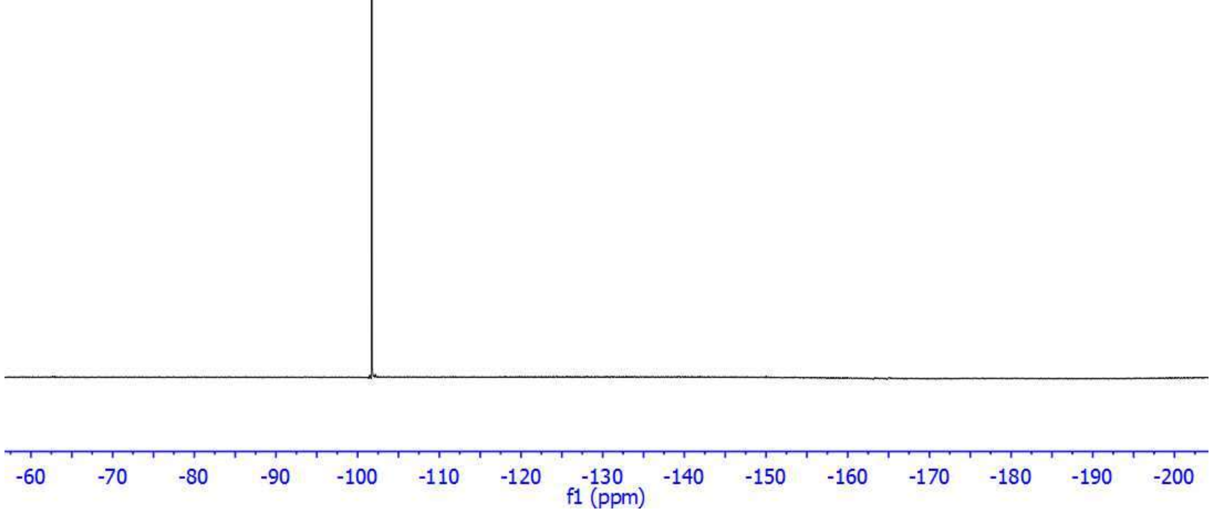
-101.7
-60
-70
-80
-90
-100
-110
-120
-130
-140
-150
-160
-170
-180
-190
-200
f1 (ppm)

**Chiral HPLC:** (Chiralpak ADH, 20% isopropanol, 80% hexane, 1.0 mL/min, λ = 210 nm) $\tau_R$ (minor) = 10.4 min, $\tau_R$ (major) = 14.7 min.

## Asymmetric

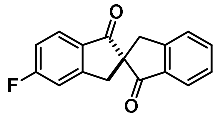


**21**

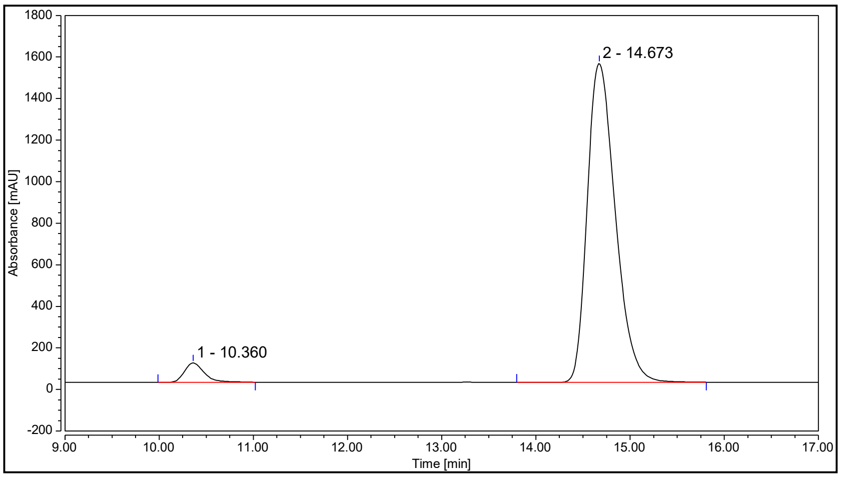


| No. | Peak Name | Retention Time | Area | Height | Relative Area |
|---|---|---|---|---|---|
| | | min | mAU*min | mAU | % |
| 1 | | 10.360 | 22.990 | 93.660 | 4.17 |
| 2 | | 14.673 | 528.143 | 1533.846 | 95.83 |
| **Total:** | | | **551.133** | **1627.506** | **100.00** |

## Racemic

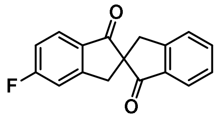


(*rac*)-**21**

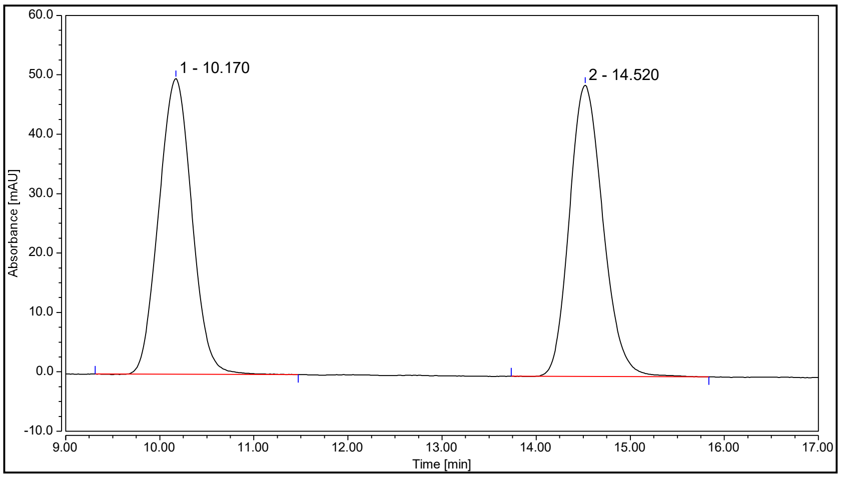


| No. | Peak Name | Retention Time | Area | Height | Relative Area |
|---|---|---|---|---|---|
| | | min | mAU*min | mAU | % |
| 1 | | 10.170 | 20.197 | 49.773 | 50.00 |
| 2 | | 14.520 | 20.196 | 49.016 | 50.00 |
| **Total:** | | | **40.393** | **98.788** | **100.00** |

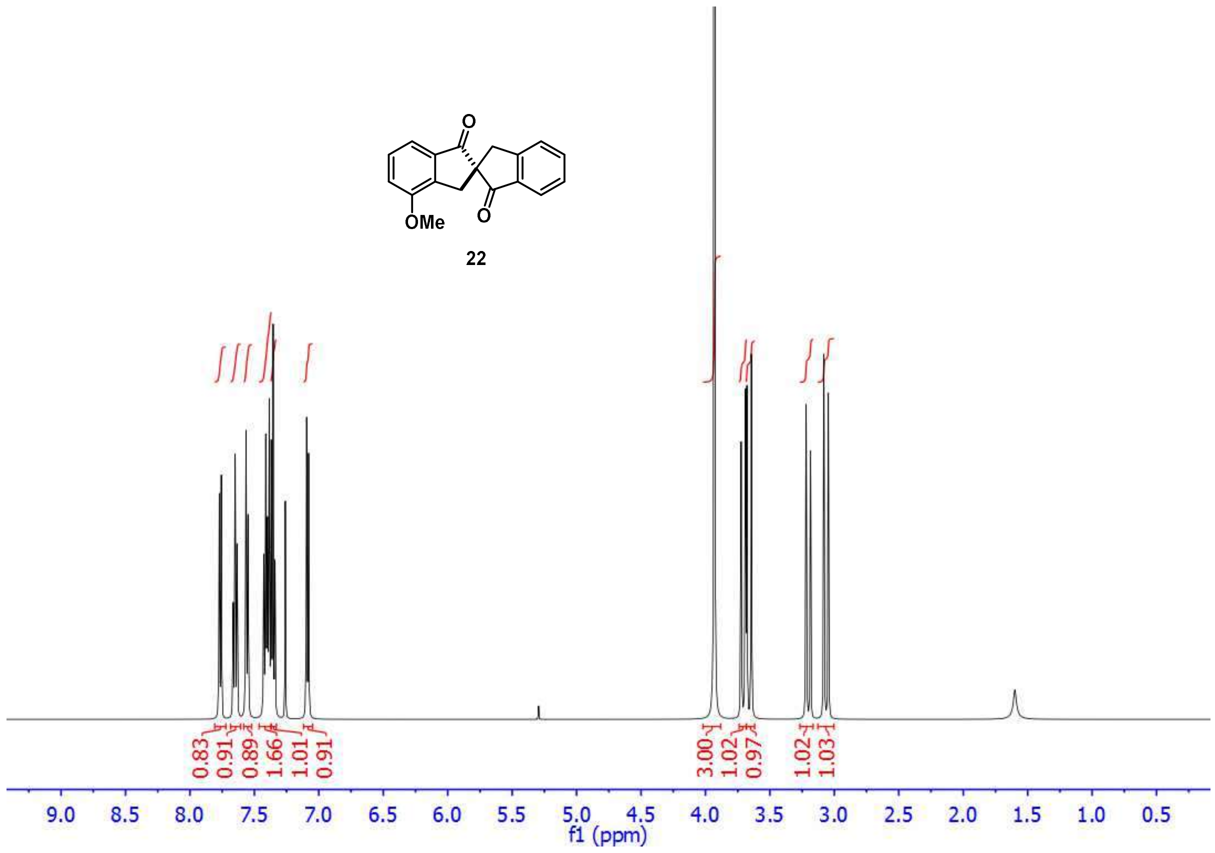

O
OMe
O
22
0.83
0.91
0.89
1.66
1.01
0.91
3.00
1.02
0.97
1.02
1.03
9.0 8.5 8.0 7.5 7.0 6.5 6.0 5.5 5.0 4.5 4.0 3.5 3.0 2.5 2.0 1.5 1.0 0.5
f1 (ppm)


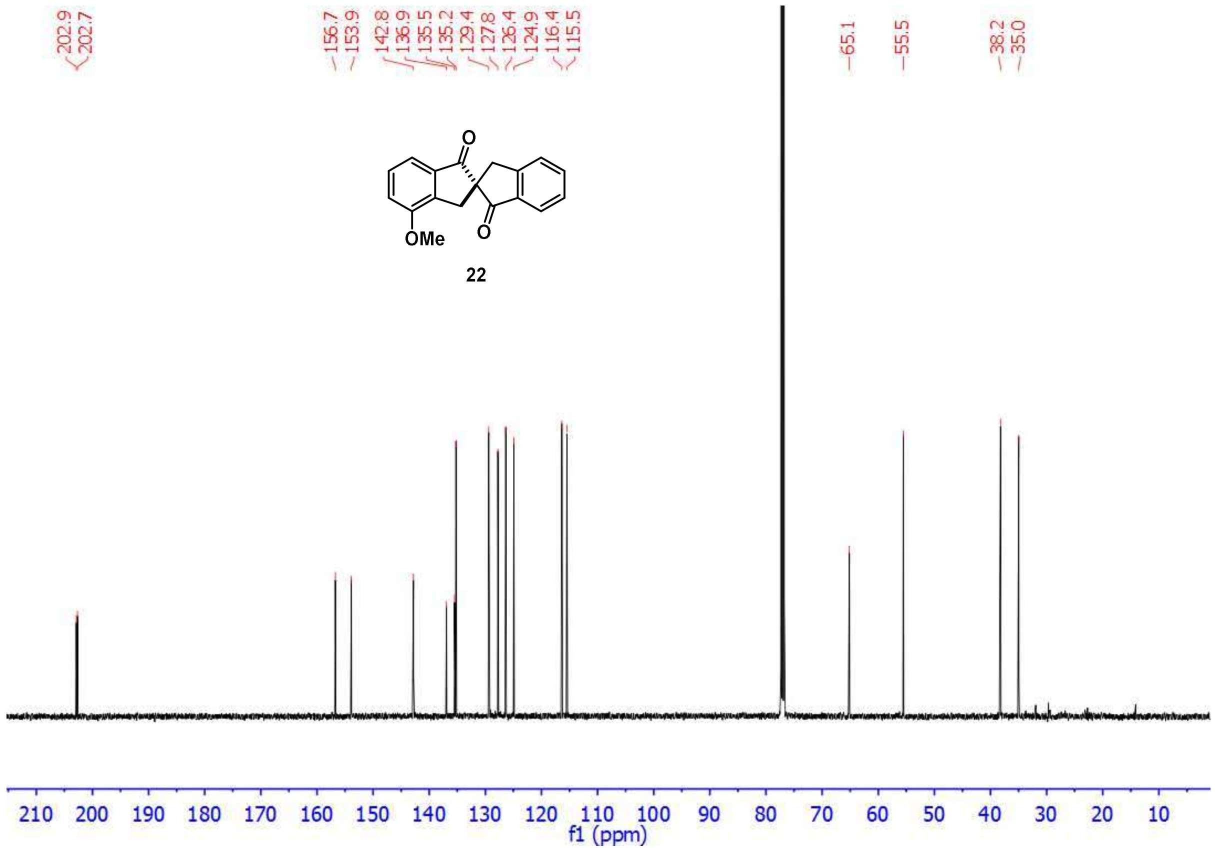

202.9
202.7
156.7
153.9
142.8
136.9
135.5
135.2
129.4
127.8
126.4
124.9
116.4
115.5
65.1
55.5
38.2
35.0
O
OMe
O
22
210 200 190 180 170 160 150 140 130 120 110 100 90 80 70 60 50 40 30 20 10
f1 (ppm)

**Chiral HPLC:** (Chiralpak ADH, 20% isopropanol, 80% hexane, 1.0 mL/min, λ = 250 nm) $\tau_R$ (minor) = 11.6 min, $\tau_R$ (major) = 16.6 min.

**Asymmetric**

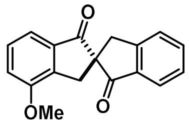

**22**

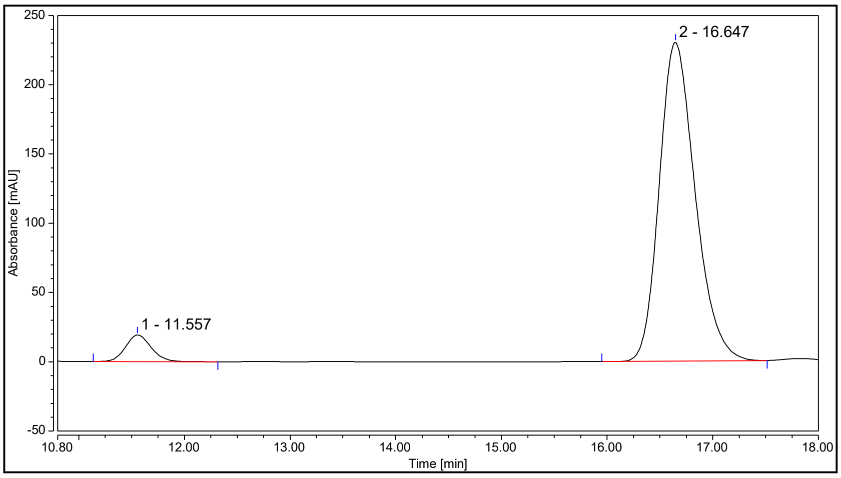


| No. | Peak Name | Retention Time | Area | Height | Relative Area |
|---|---|---|---|---|---|
| | | min | mAU*min | mAU | % |
| 1 | | 11.557 | 5.407 | 19.214 | 5.63 |
| 2 | | 16.647 | 90.682 | 230.148 | 94.37 |
| **Total:** | | | **96.089** | **249.362** | **100.00** |

**Racemic**

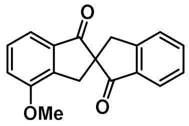

(*rac*)-**22**

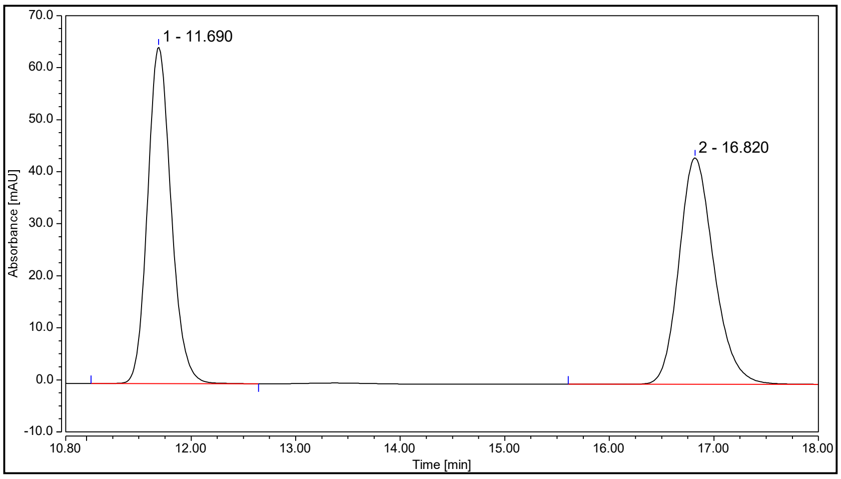


| No. | Peak Name | Retention Time | Area | Height | Relative Area |
|---|---|---|---|---|---|
| | | min | mAU*min | mAU | % |
| 1 | | 11.690 | 16.893 | 64.696 | 50.06 |
| 2 | | 16.820 | 16.855 | 43.507 | 49.94 |
| **Total:** | | | **33.748** | **108.202** | **100.00** |

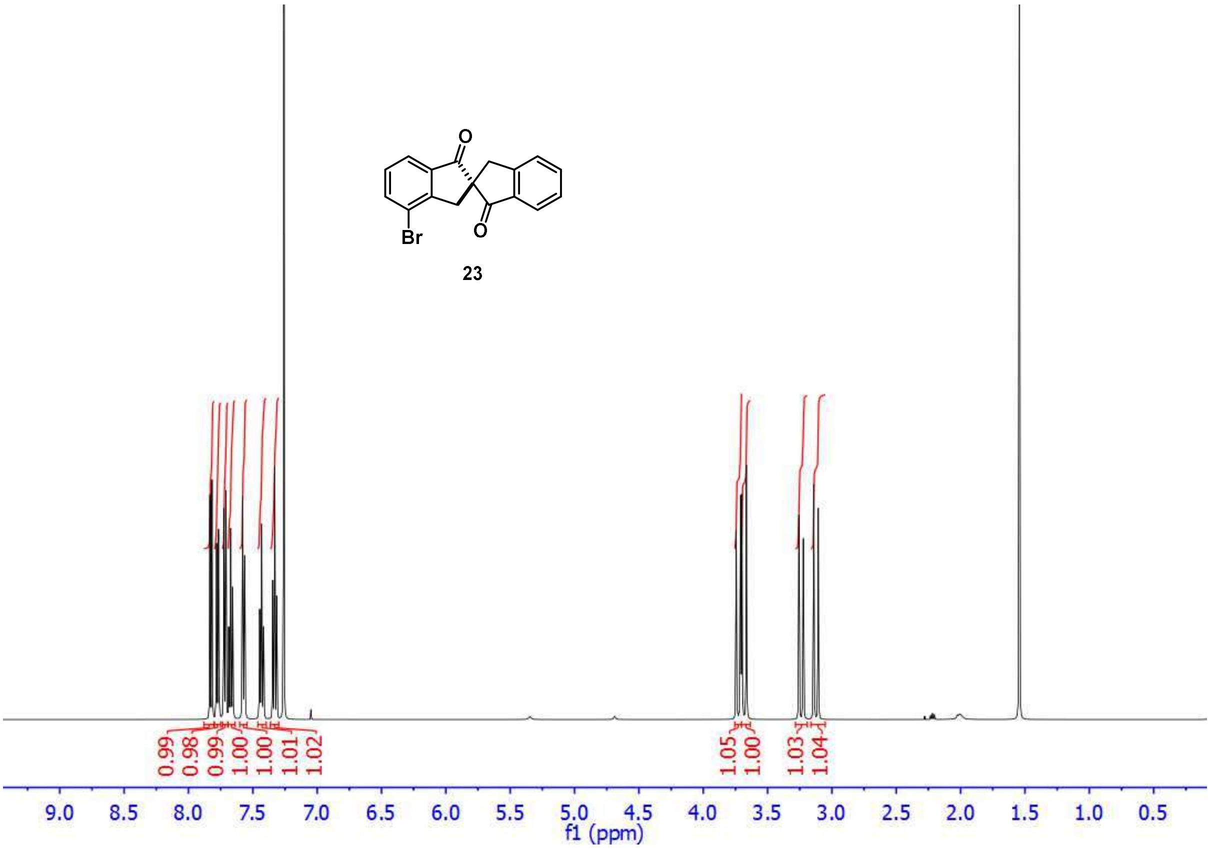
23
0.99
0.98
0.99
1.00
1.00
1.01
1.02
1.05
1.00
1.03
1.04
9.0 8.5 8.0 7.5 7.0 6.5 6.0 5.5 5.0 4.5 4.0 3.5 3.0 2.5 2.0 1.5 1.0 0.5
f1 (ppm)

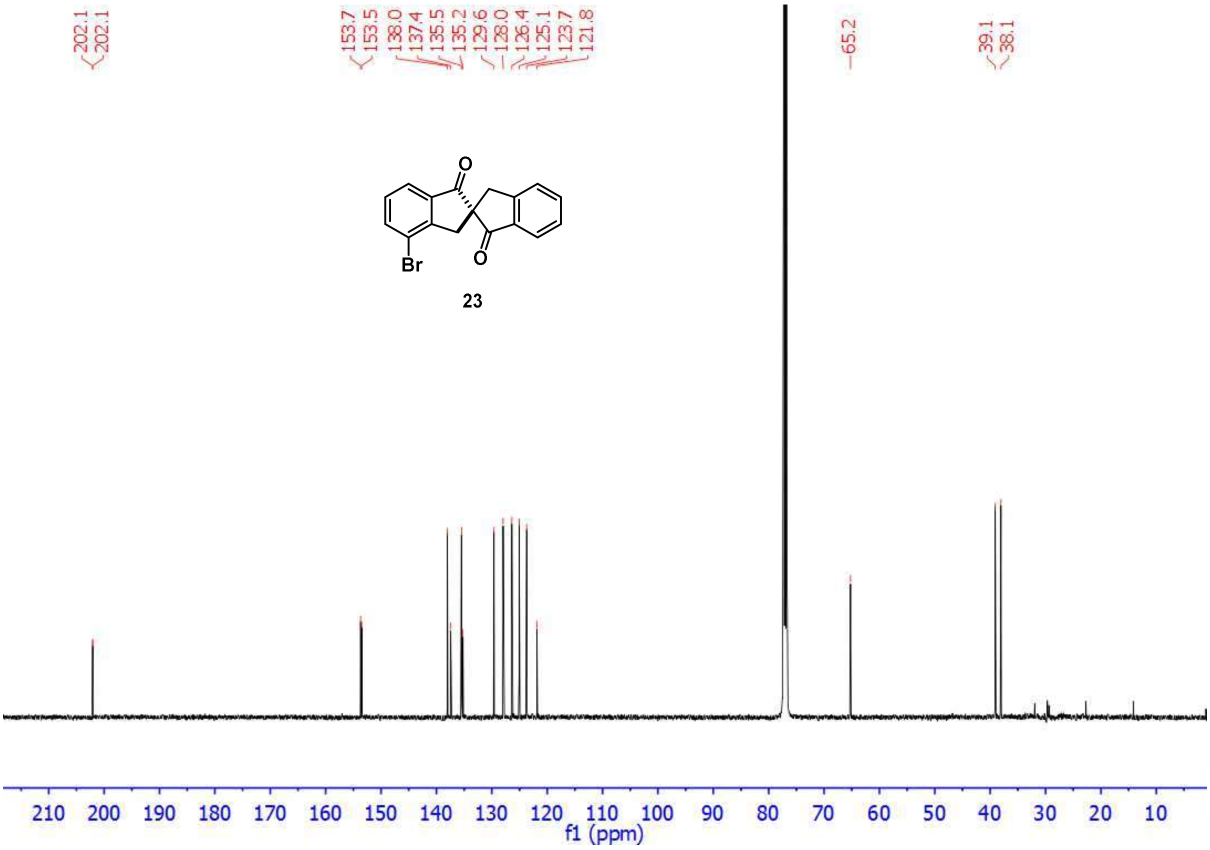
202.1
202.1
153.7
153.5
138.0
137.4
135.5
135.2
129.6
128.0
126.4
125.1
123.7
121.8
65.2
39.1
38.1
23
210 200 190 180 170 160 150 140 130 120 110 100 90 80 70 60 50 40 30 20 10
f1 (ppm)

**Chiral HPLC:** (Chiralpak ADH, 2% isopropanol, 98% hexane, 1.0 mL/min, λ = 254 nm) $\tau_R$ (minor) = 10.1 min, $\tau_R$ (major) = 14.4 min.

**<u>Asymmetric</u>**

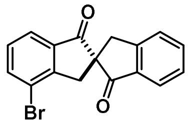


**23**

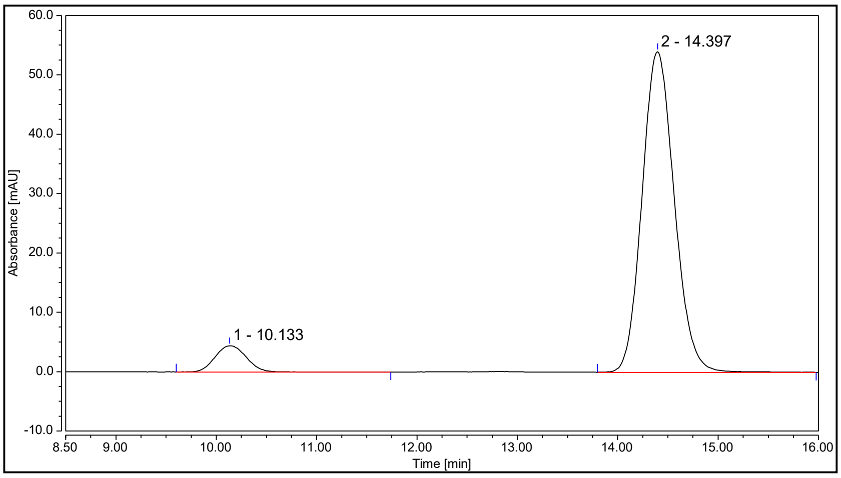


| No. | Peak Name | Retention Time | Area | Height | Relative Area |
|---|---|---|---|---|---|
| | | min | mAU*min | mAU | % |
| 1 | | 10.133 | 1.596 | 4.429 | 7.35 |
| 2 | | 14.397 | 20.125 | 54.021 | 92.65 |
| **Total:** | | | **21.721** | **58.450** | **100.00** |

**<u>Racemic</u>**

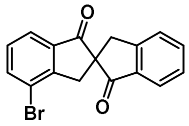


(*rac*)-**23**

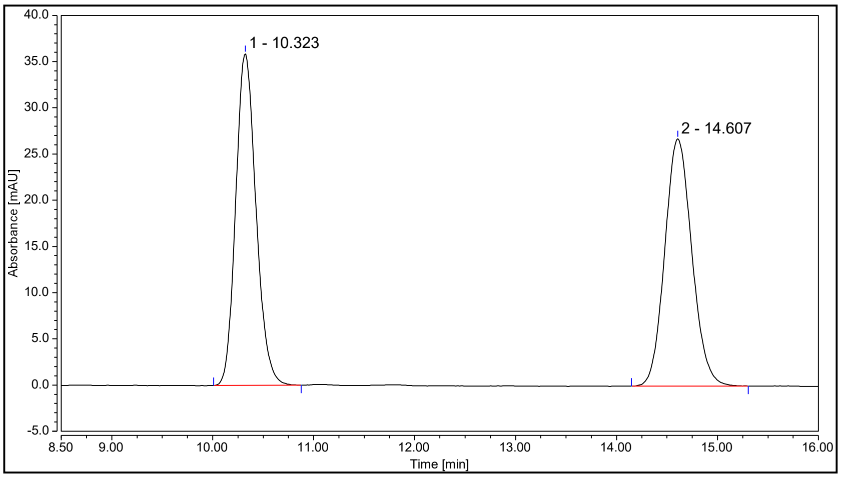


| No. | Peak Name | Retention Time | Area | Height | Relative Area |
|---|---|---|---|---|---|
| | | min | mAU*min | mAU | % |
| 1 | | 10.323 | 8.361 | 35.914 | 50.07 |
| 2 | | 14.607 | 8.337 | 26.769 | 49.93 |
| **Total:** | | | **16.699** | **62.683** | **100.00** |

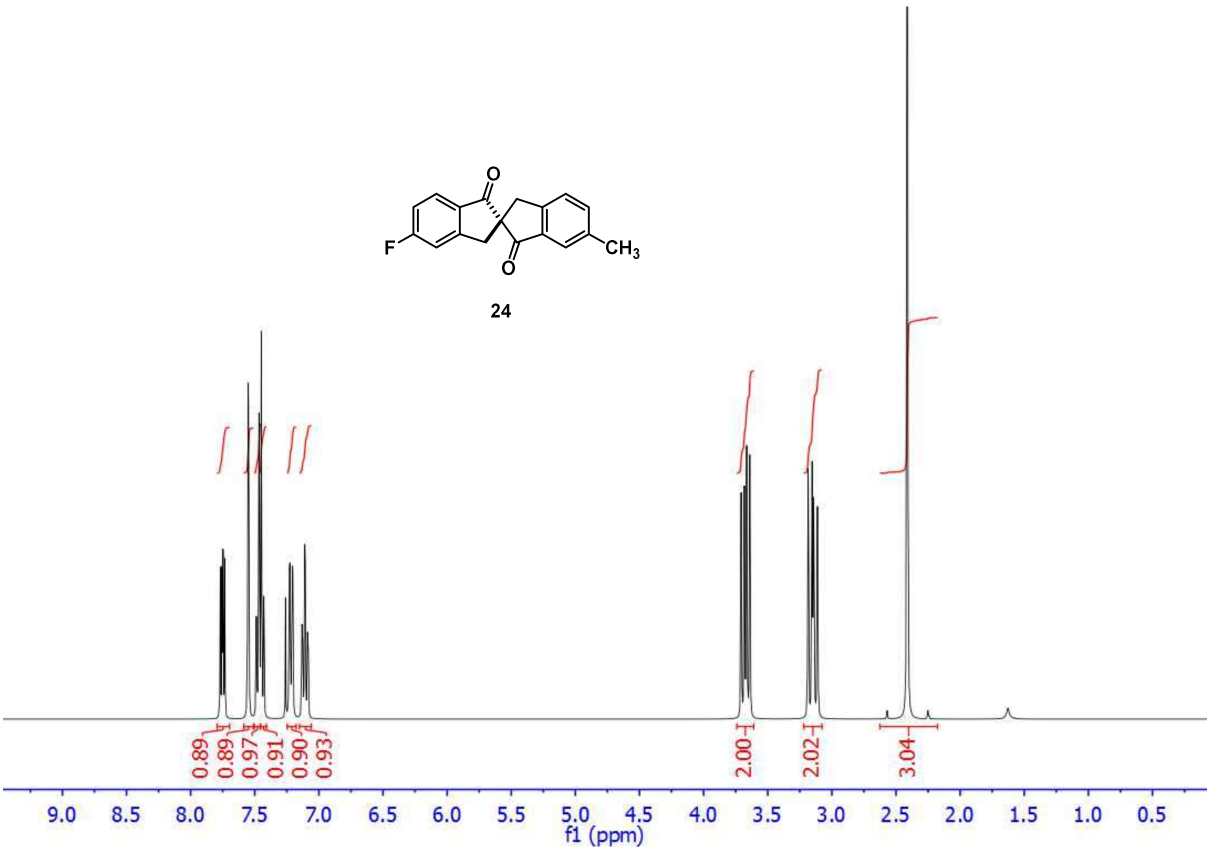
F
O
O
CH3
24
0.89
0.89
0.97
0.91
0.90
0.93
2.00
2.02
3.04
9.0 8.5 8.0 7.5 7.0 6.5 6.0 5.5 5.0 4.5 4.0 3.5 3.0 2.5 2.0 1.5 1.0 0.5
f1 (ppm)

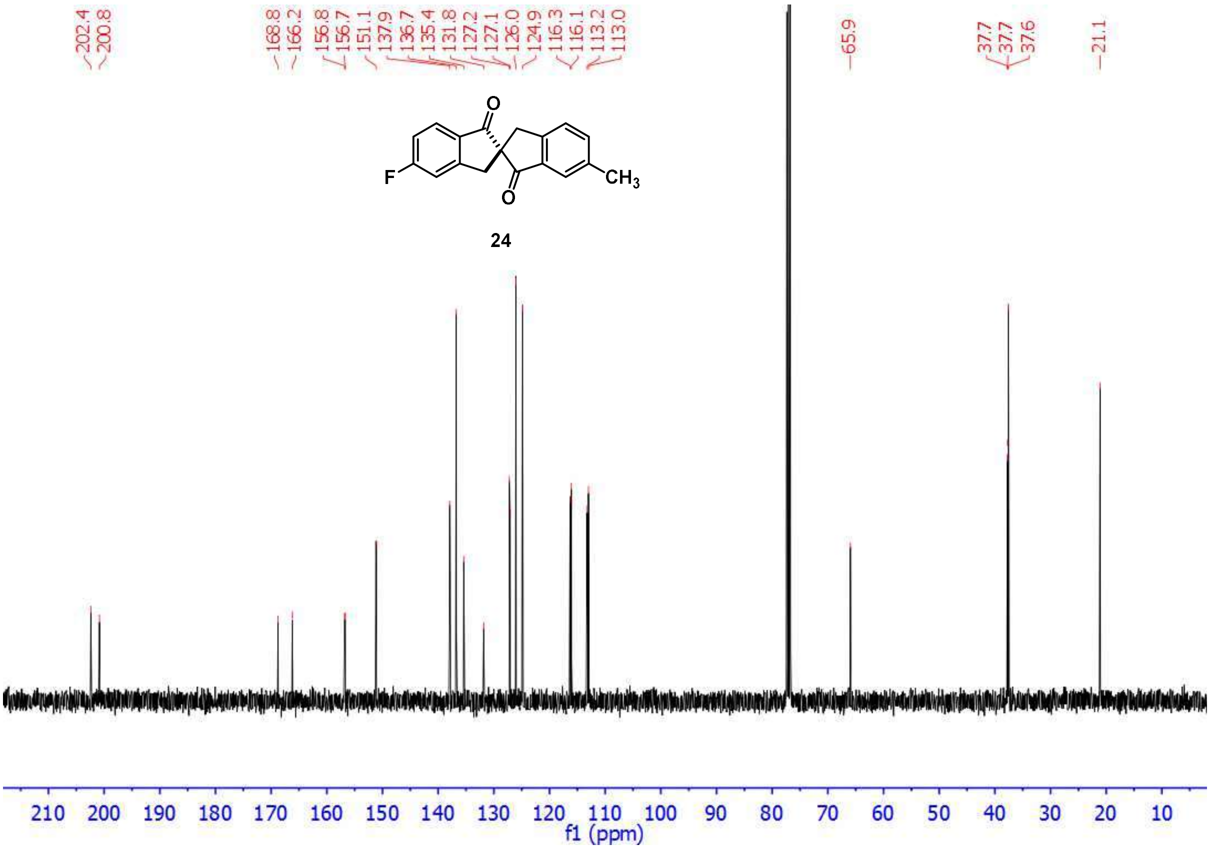
202.4
200.8
168.8
166.2
156.8
156.7
151.1
137.9
136.7
135.4
131.8
127.2
127.1
126.0
124.9
116.3
116.1
113.2
113.0
65.9
37.7
37.7
37.6
21.1
F
O
O
CH3
24
210 200 190 180 170 160 150 140 130 120 110 100 90 80 70 60 50 40 30 20 10
f1 (ppm)

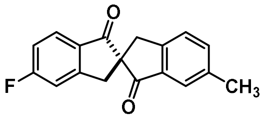
F
O
O
CH3

24

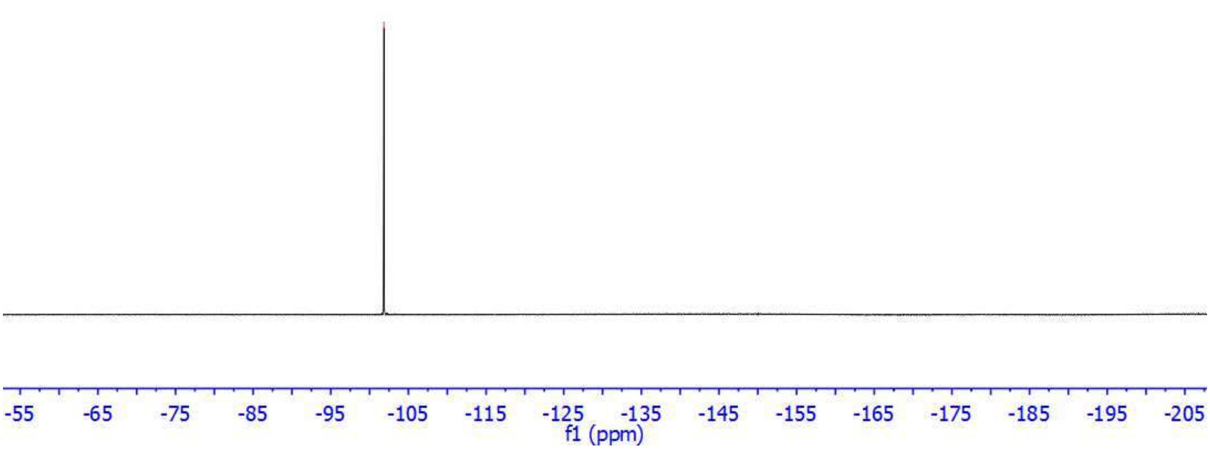
—101.8
-55
-65
-75
-85
-95
-105
-115
-125
-135
-145
-155
-165
-175
-185
-195
-205
f1 (ppm)

**Chiral HPLC:** (Chiralpak ADH, 20% isopropanol, 80% hexane, 1.0 mL/min, $\lambda$ = 299 nm) $\tau_R$ (minor) = 9.8 min, $\tau_R$ (major) = 15.6 min.

## Asymmetric

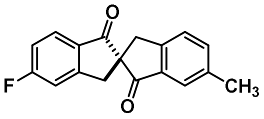


**24**

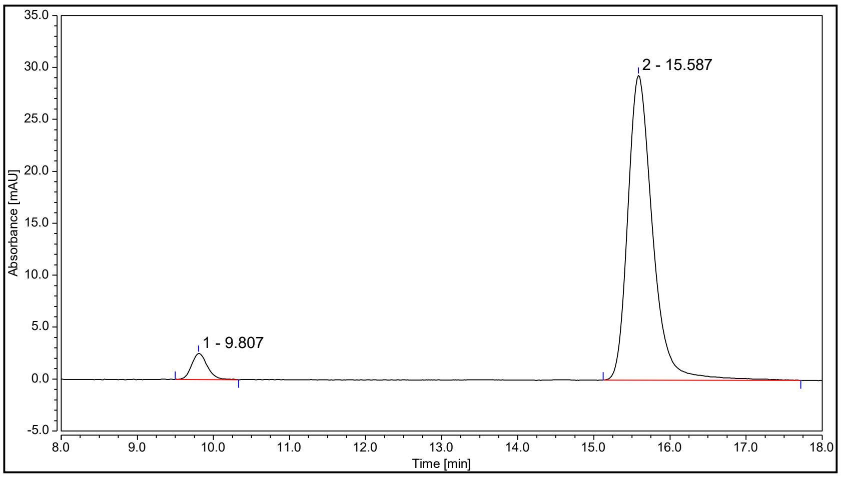


| No. | Peak Name | Retention Time | Area | Height | Relative Area |
|---|---|---|---|---|---|
| | | min | mAU*min | mAU | % |
| 1 | | 9.807 | 0.604 | 2.506 | 5.15 |
| 2 | | 15.587 | 11.132 | 29.337 | 94.85 |
| **Total:** | | | **11.735** | **31.844** | **100.00** |

## Racemic

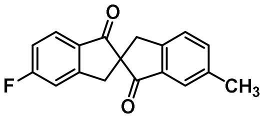


(*rac*)-**24**

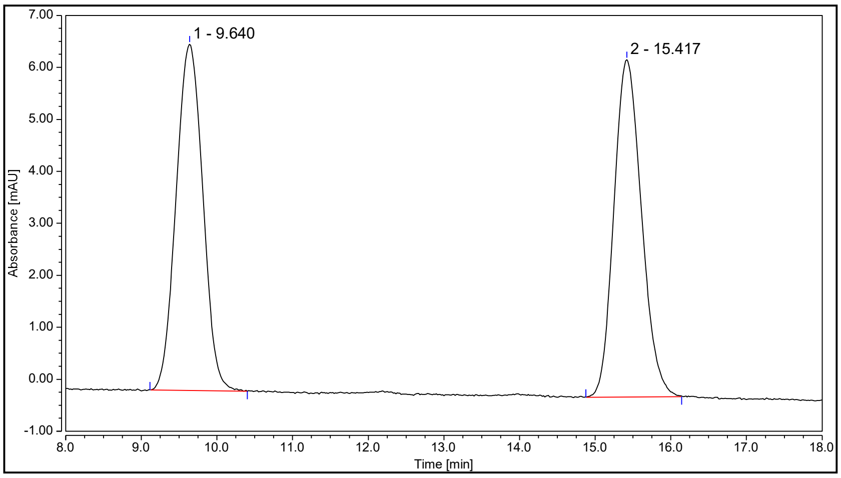


| No. | Peak Name | Retention Time | Area | Height | Relative Area |
|---|---|---|---|---|---|
| | | min | mAU*min | mAU | % |
| 1 | | 9.640 | 2.725 | 6.663 | 49.82 |
| 2 | | 15.417 | 2.745 | 6.492 | 50.18 |
| **Total:** | | | **5.470** | **13.155** | **100.00** |

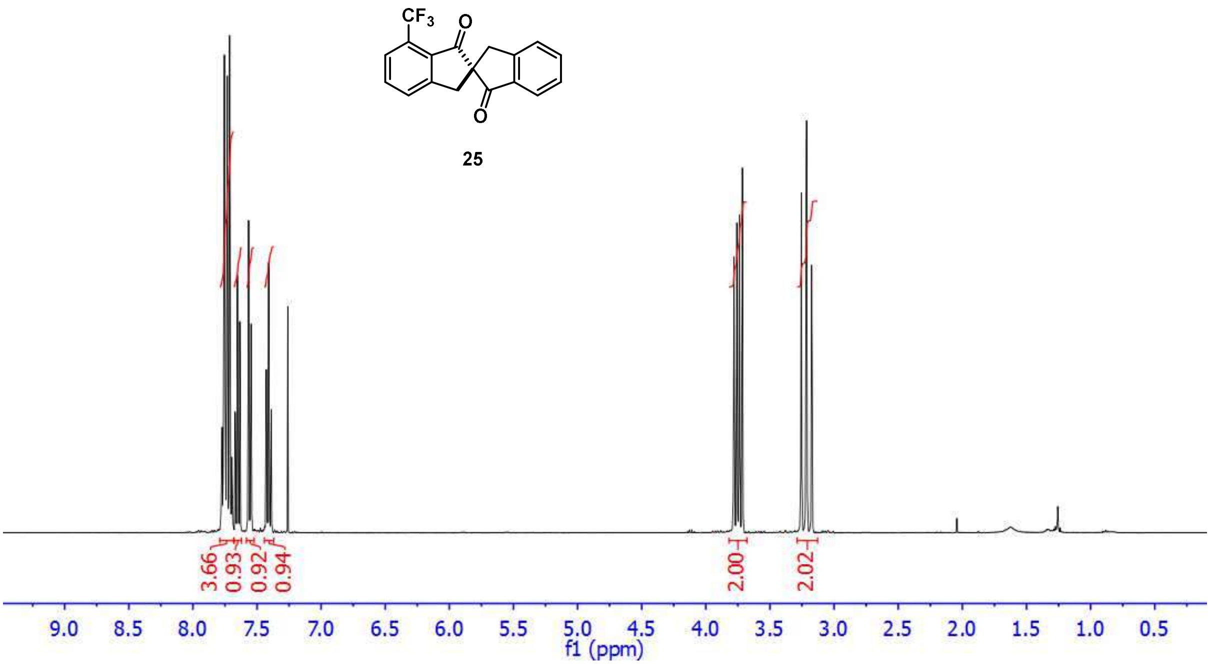
CF3
O
O
25
3.66
0.93
0.92
0.94
2.00
2.02
9.0 8.5 8.0 7.5 7.0 6.5 6.0 5.5 5.0 4.5 4.0 3.5 3.0 2.5 2.0 1.5 1.0 0.5
f1 (ppm)

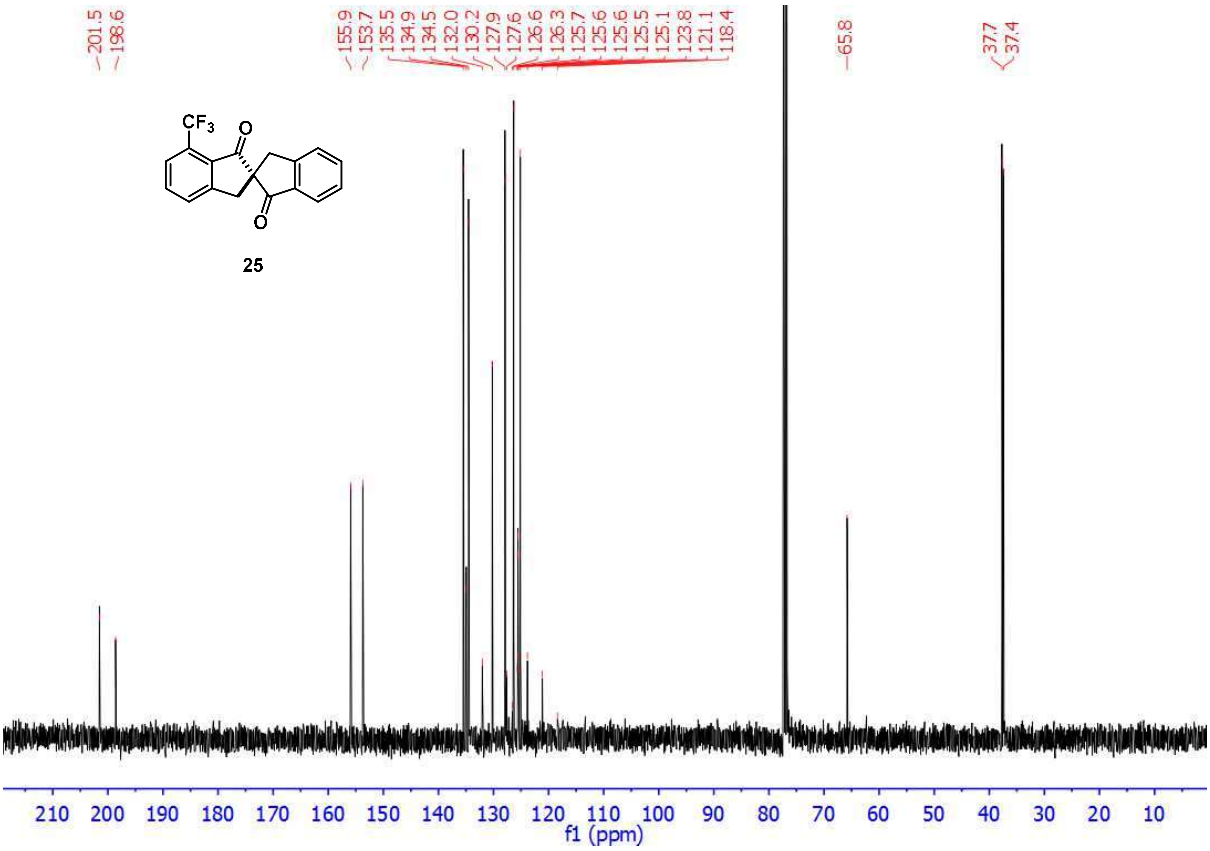
201.5
198.6
155.9
153.7
135.5
134.9
134.5
132.0
130.2
127.9
127.6
126.6
126.3
125.7
125.6
125.6
125.5
125.1
123.8
121.1
118.4
65.8
37.7
37.4
CF3
O
O
25
210 200 190 180 170 160 150 140 130 120 110 100 90 80 70 60 50 40 30 20 10
f1 (ppm)

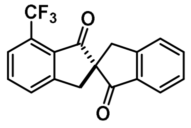

CF3
O
O


**25**

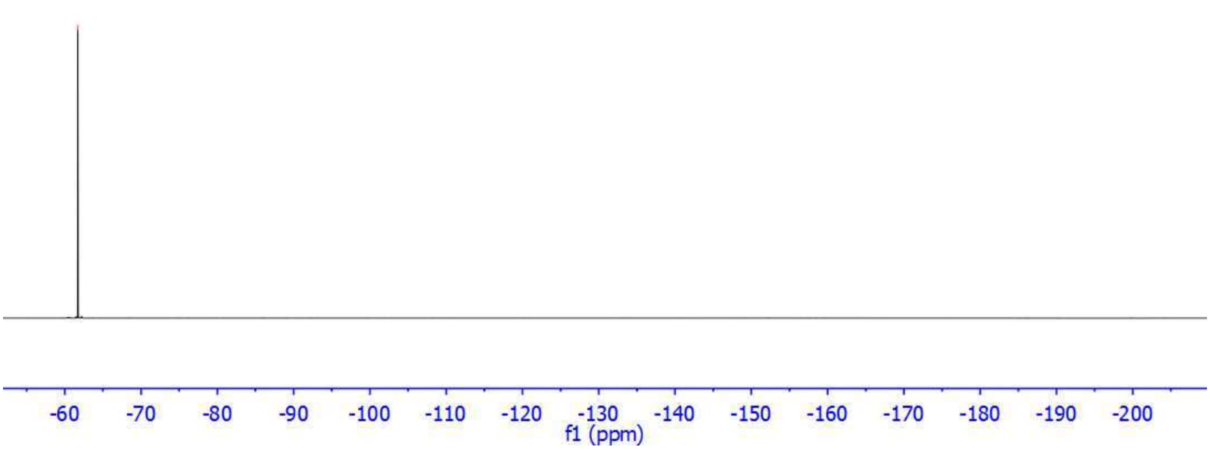

-61.7
-60
-70
-80
-90
-100
-110
-120
-130
-140
-150
-160
-170
-180
-190
-200
f1 (ppm)

**Chiral HPLC:** (Chiralpak ADH, 10% isopropanol, 90% hexane, 1.0 mL/min, $\lambda$ = 250 nm) $\tau_R$ (minor) = 10.7 min, $\tau_R$ (major) = 26.0 min.

## Asymmetric

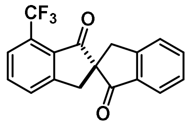


**25**

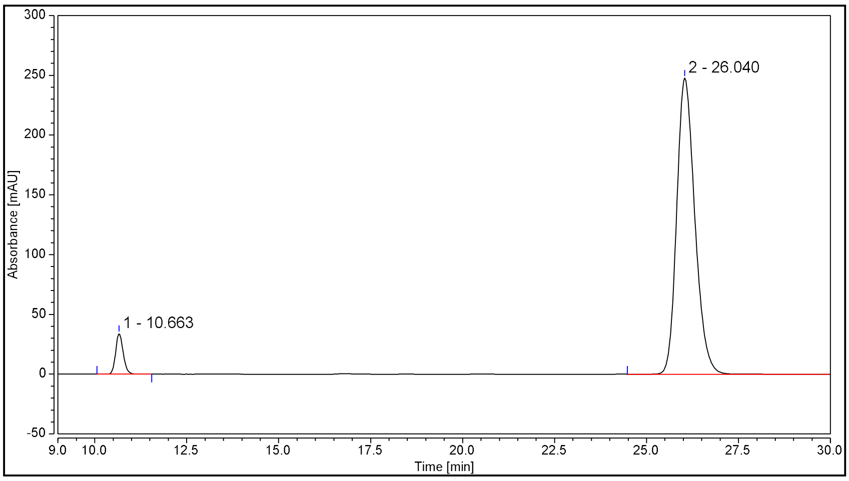


| No. | Peak Name | Retention Time | Area | Height | Relative Area |
|---|---|---|---|---|---|
| | | min | mAU*min | mAU | % |
| 1 | | 10.663 | 8.054 | 33.959 | 5.39 |
| 2 | | 26.040 | 141.476 | 247.668 | 94.61 |
| **Total:** | | | **149.530** | **281.627** | **100.00** |

## Racemic

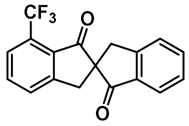


(*rac*)-**25**

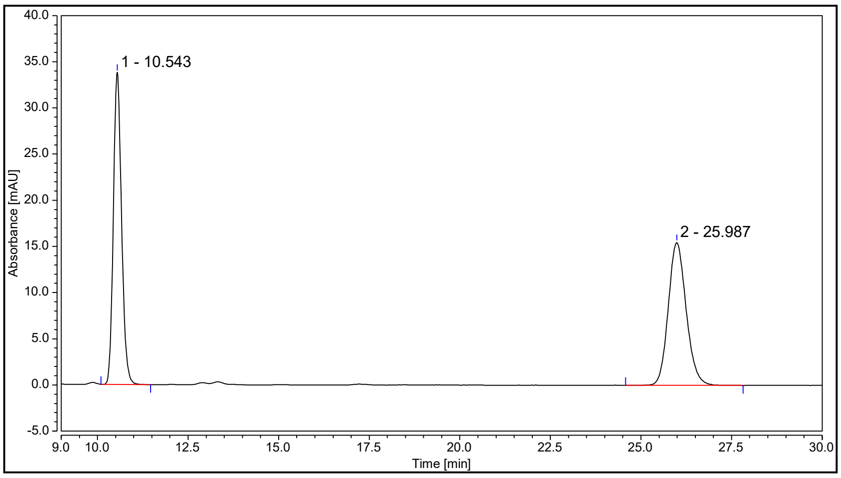


| No. | Peak Name | Retention Time | Area | Height | Relative Area |
|---|---|---|---|---|---|
| | | min | mAU*min | mAU | % |
| 1 | | 10.543 | 8.852 | 33.798 | 49.99 |
| 2 | | 25.987 | 8.856 | 15.466 | 50.01 |
| **Total:** | | | **17.707** | **49.264** | **100.00** |

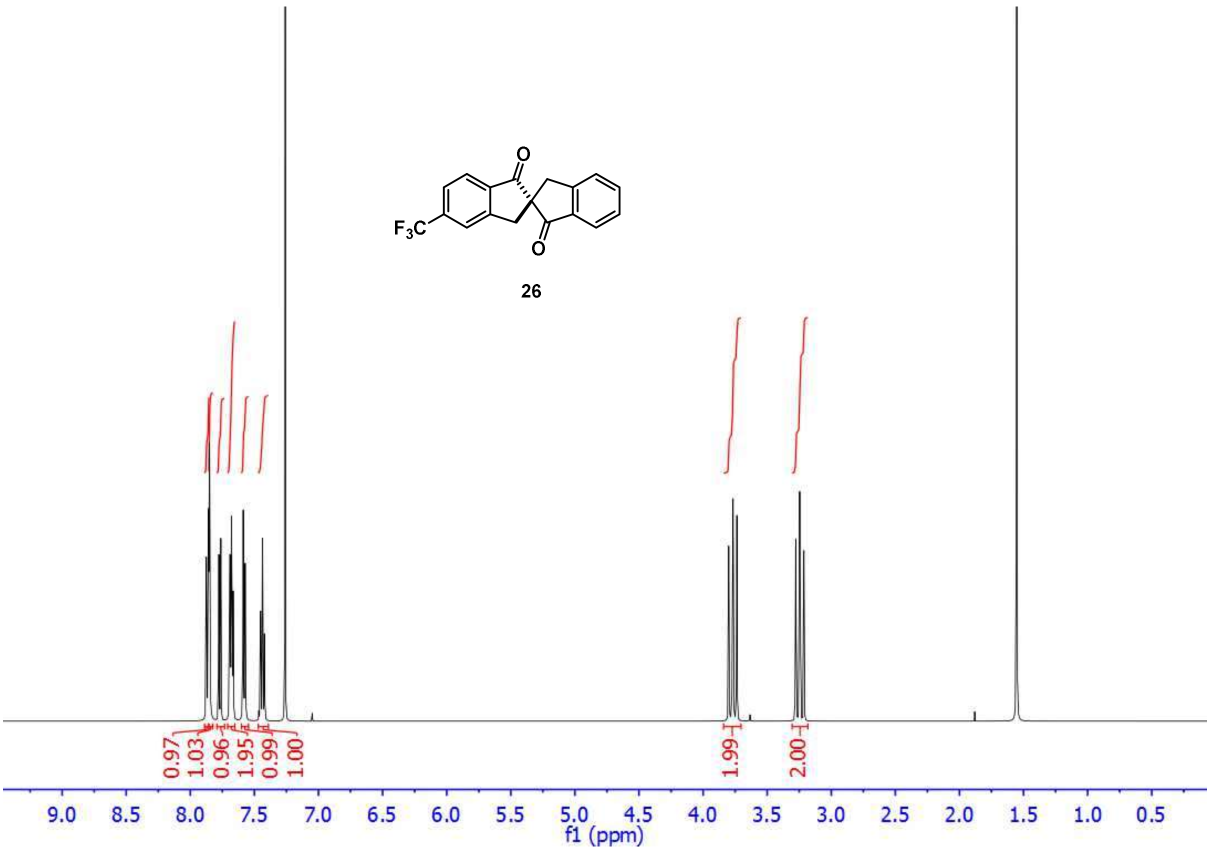

F3C
O
O
26
0.97
1.03
0.96
1.95
0.99
1.00
1.99
2.00
9.0
8.5
8.0
7.5
7.0
6.5
6.0
5.5
5.0
4.5
4.0
3.5
3.0
2.5
2.0
1.5
1.0
0.5
f1 (ppm)


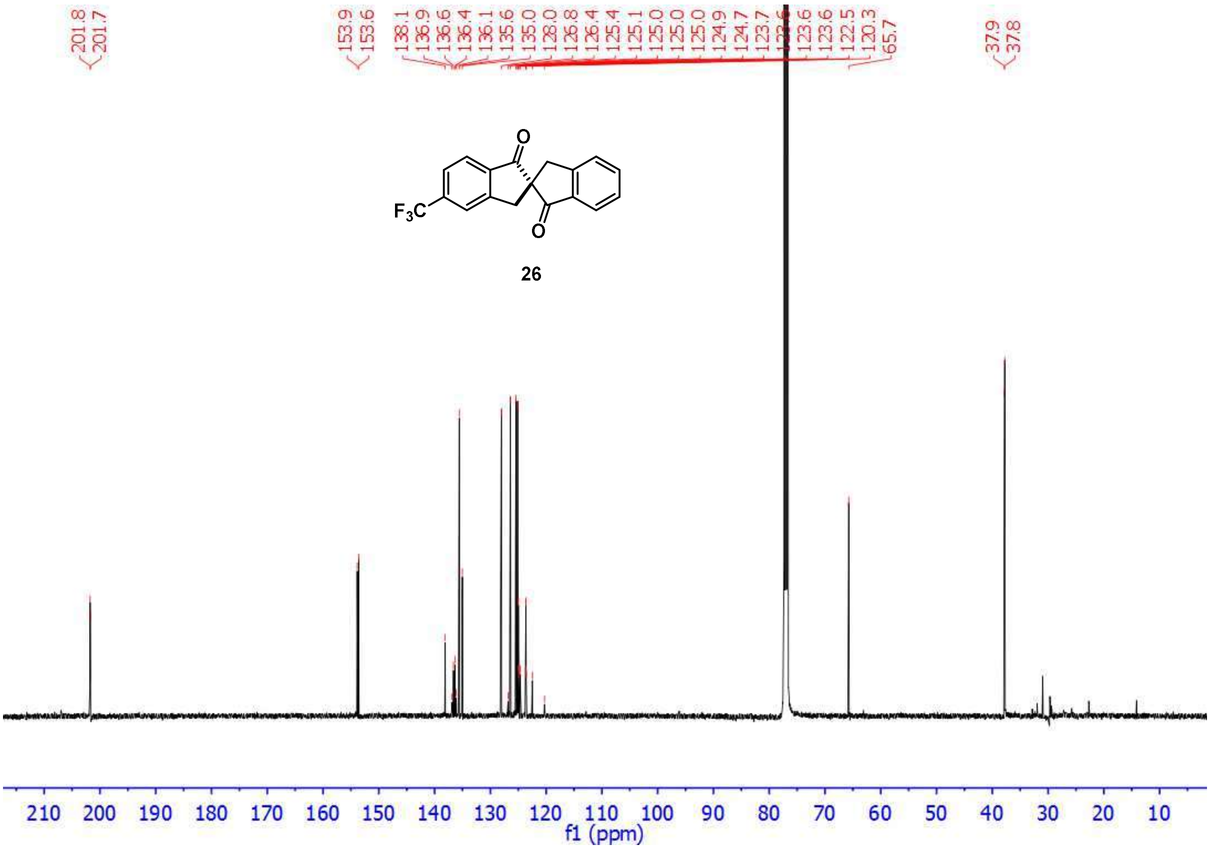

201.8
201.7
153.9
153.6
138.1
136.9
136.6
136.4
136.1
135.6
135.0
128.0
126.8
126.4
125.4
125.1
125.0
125.0
125.0
124.9
124.7
123.7
123.6
123.6
122.5
120.3
65.7
37.9
37.8
F3C
O
O
26
210
200
190
180
170
160
150
140
130
120
110
100
90
80
70
60
50
40
30
20
10
f1 (ppm)

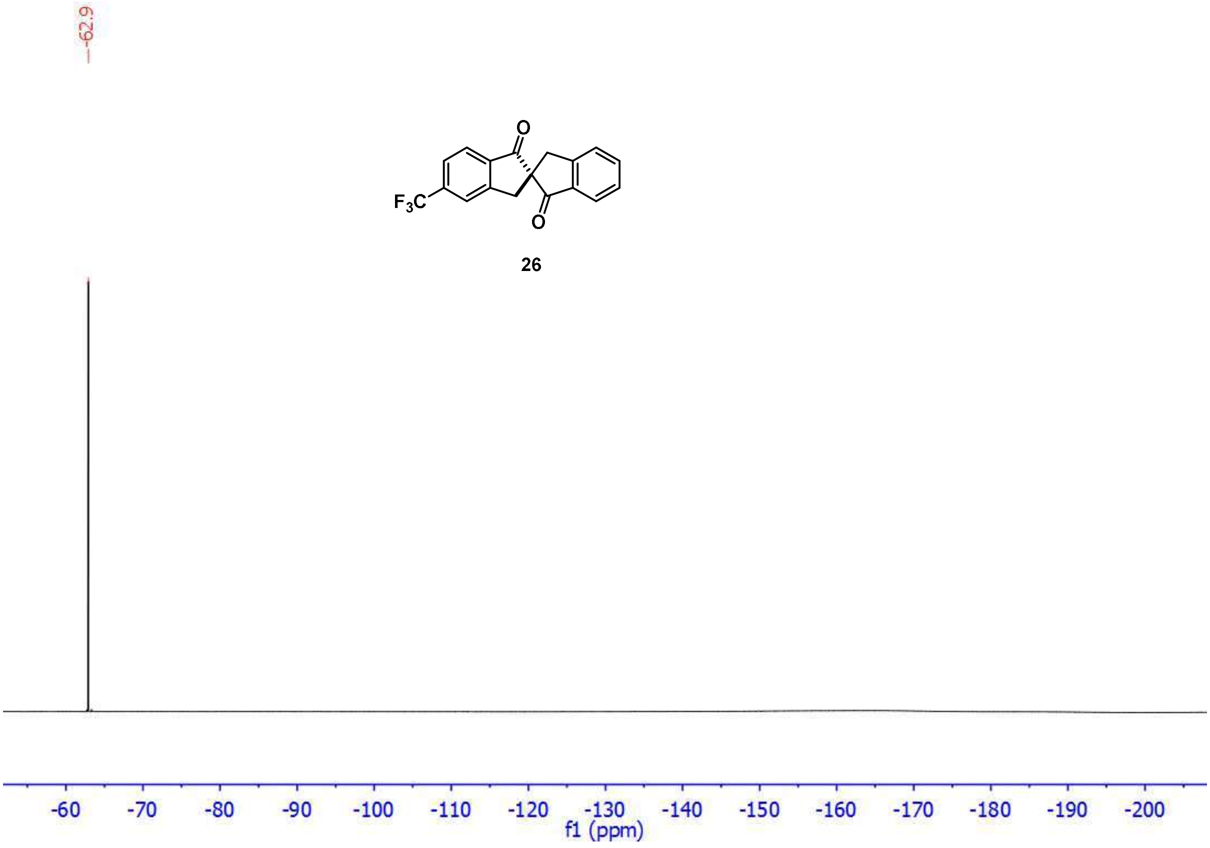
-62.9
F3C
O
O
26
-60 -70 -80 -90 -100 -110 -120 -130 -140 -150 -160 -170 -180 -190 -200
f1 (ppm)

**Chiral HPLC:** (Chiralpak IA, 2% isopropanol, 98% hexane, 1.0 mL/min, λ = 250 nm)
$\tau_R$ (minor) = 23.9 min, $\tau_R$ (major) = 26.8 min.

**Asymmetric (1)**

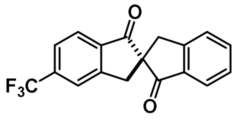


**26**

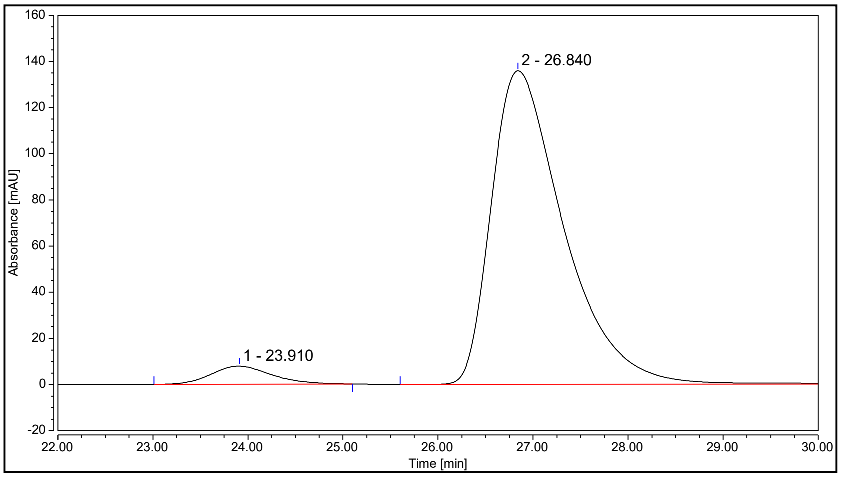


| No. | Peak Name | Retention Time | Area | Height | Relative Area |
|---|---|---|---|---|---|
| | | min | mAU*min | mAU | % |
| 1 | | 23.910 | 5.773 | 7.822 | 4.59 |
| 2 | | 26.840 | 119.920 | 135.818 | 95.41 |
| **Total:** | | | **125.692** | **143.639** | **100.00** |

**Asymmetric (2)**

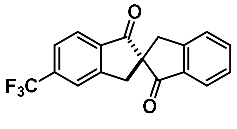


**26**

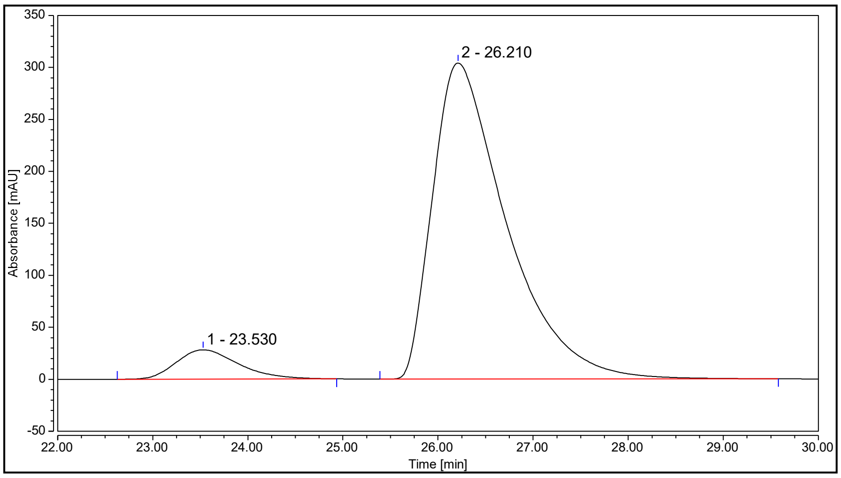


| No. | Peak Name | Retention Time | Area | Height | Relative Area |
|---|---|---|---|---|---|
| | | min | mAU*min | mAU | % |
| 1 | | 23.530 | 21.271 | 28.150 | 7.21 |
| 2 | | 26.210 | 273.707 | 304.012 | 92.79 |
| **Total:** | | | **294.978** | **332.162** | **100.00** |

# Racemic

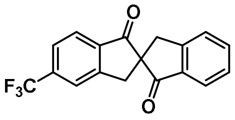


(*rac*)-**26**

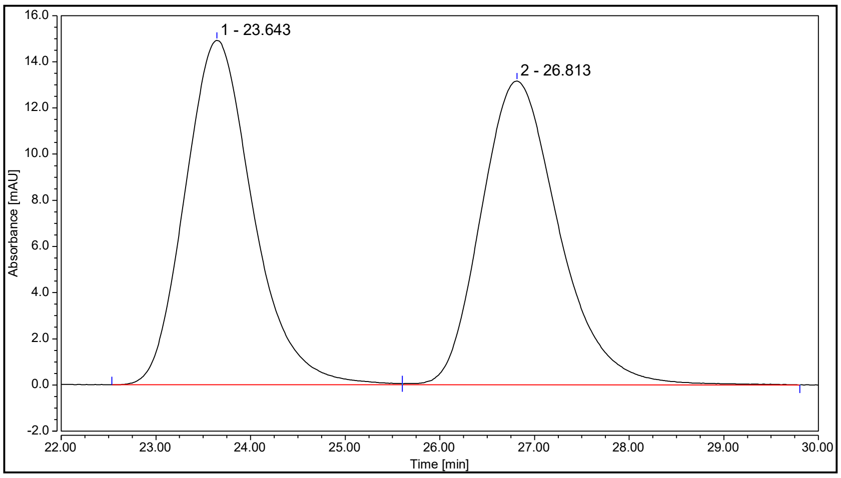


| No. | Peak Name | Retention Time | Area | Height | Relative Area |
|---|---|---|---|---|---|
| | | min | mAU*min | mAU | % |
| 1 | | 23.643 | 12.433 | 14.920 | 49.80 |
| 2 | | 26.813 | 12.534 | 13.163 | 50.20 |
| **Total:** | | | **24.967** | **28.083** | **100.00** |

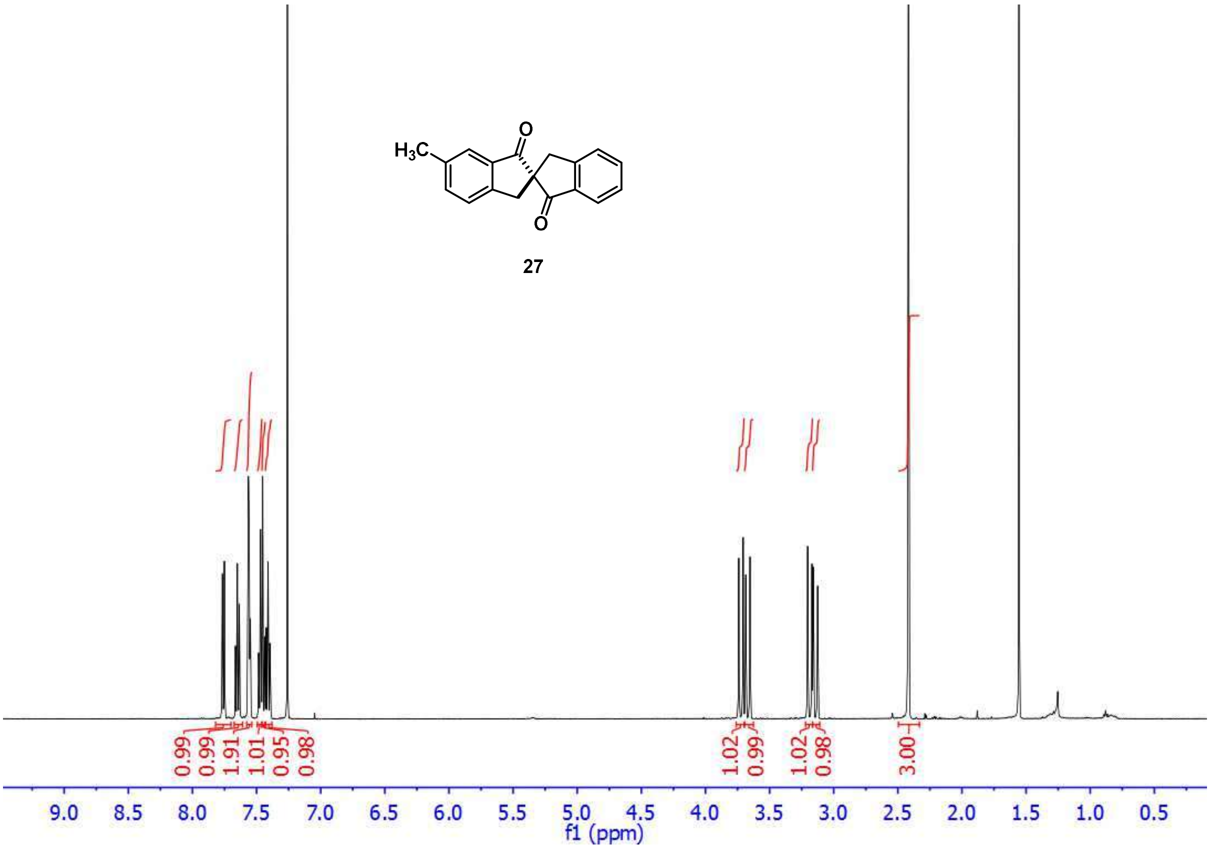
H3C
O
O
27
0.99
0.99
1.91
1.01
0.95
0.98
1.02
0.99
1.02
0.98
3.00
9.0 8.5 8.0 7.5 7.0 6.5 6.0 5.5 5.0 4.5 4.0 3.5 3.0 2.5 2.0 1.5 1.0 0.5
f1 (ppm)

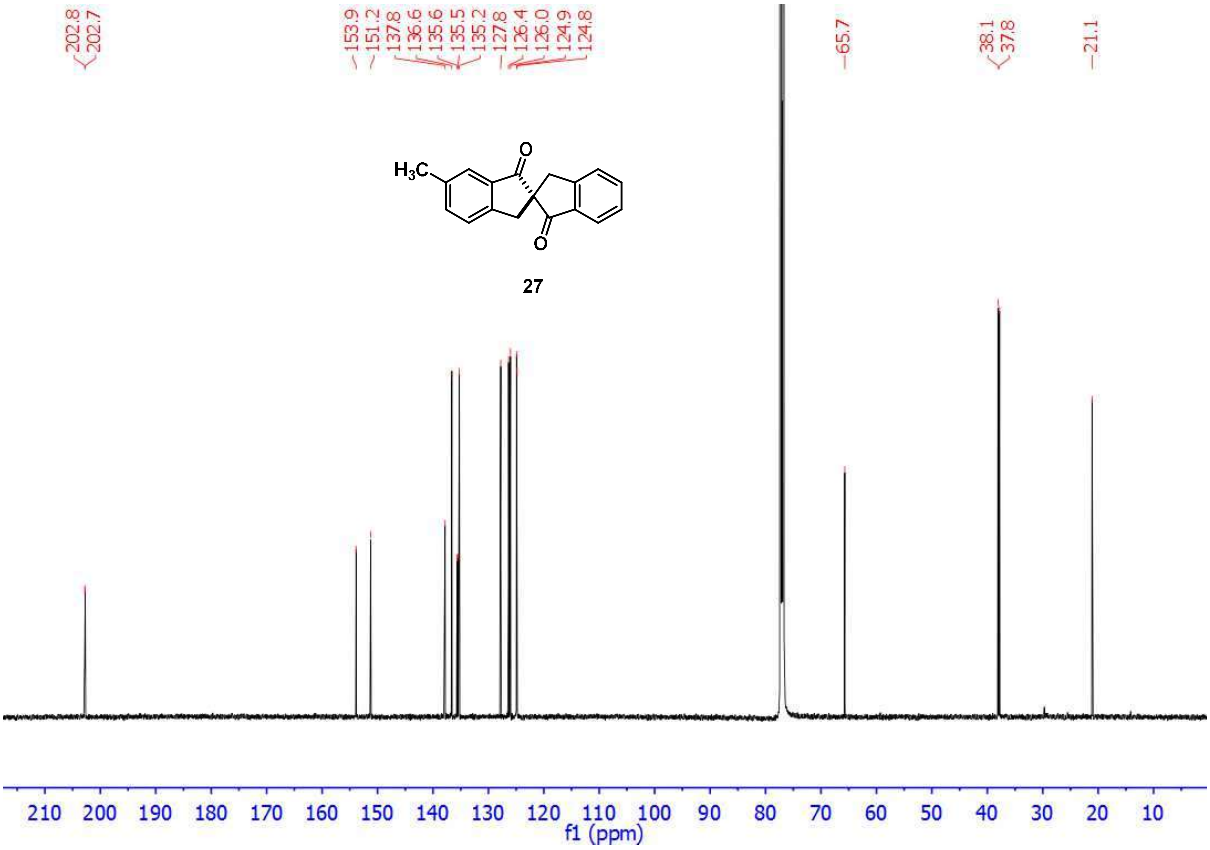
202.8
202.7
153.9
151.2
137.8
136.6
135.6
135.5
135.2
127.8
126.4
126.0
124.9
124.8
65.7
38.1
37.8
21.1
H3C
O
O
27
210 200 190 180 170 160 150 140 130 120 110 100 90 80 70 60 50 40 30 20 10
f1 (ppm)

**Chiral HPLC:** (Chiralpak ADH, 20% isopropanol, 80% hexane, 1.0 mL/min, λ = 299 nm) $\tau_R$ (minor) = 8.5 min, $\tau_R$ (major) = 18.6 min.

## Asymmetric (1)

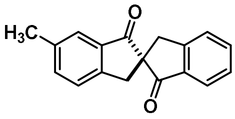


27

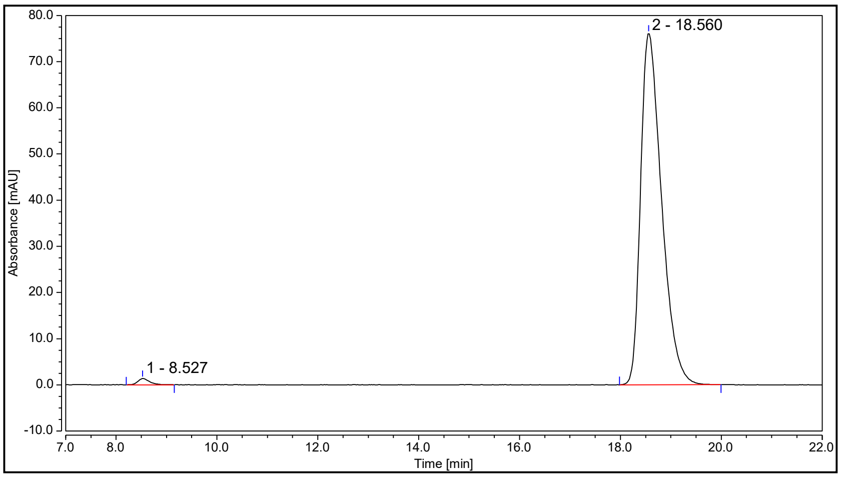


| No. | Peak Name | Retention Time | Area | Height | Relative Area |
|---|---|---|---|---|---|
| | | min | mAU*min | mAU | % |
| 1 | | 8.527 | 0.372 | 1.337 | 1.00 |
| 2 | | 18.560 | 36.665 | 76.139 | 99.00 |
| **Total:** | | | **37.037** | **77.477** | **100.00** |

## Asymmetric (2)

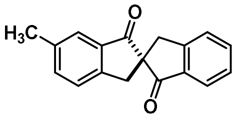


27

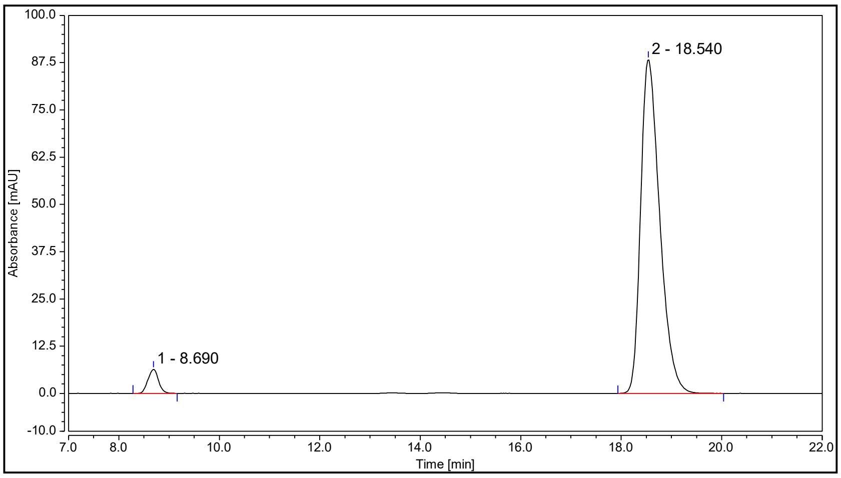


| No. | Peak Name | Retention Time | Area | Height | Relative Area |
|---|---|---|---|---|---|
| | | min | mAU*min | mAU | % |
| 1 | | 8.690 | 1.548 | 6.380 | 3.82 |
| 2 | | 18.540 | 38.969 | 88.381 | 96.18 |
| **Total:** | | | **40.517** | **94.761** | **100.00** |

## Racemic

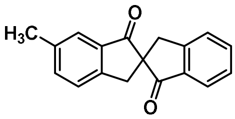

(*rac*)-**27**

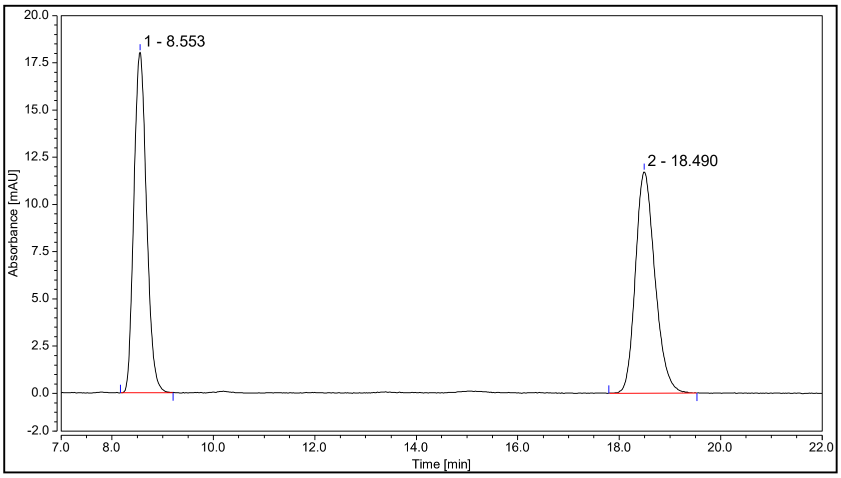


| No. | Peak Name | Retention Time | Area | Height | Relative Area |
|---|---|---|---|---|---|
| | | min | mAU*min | mAU | % |
| 1 | | 8.553 | 5.216 | 18.031 | 49.88 |
| 2 | | 18.490 | 5.240 | 11.737 | 50.12 |
| **Total:** | | | **10.456** | **29.767** | **100.00** |

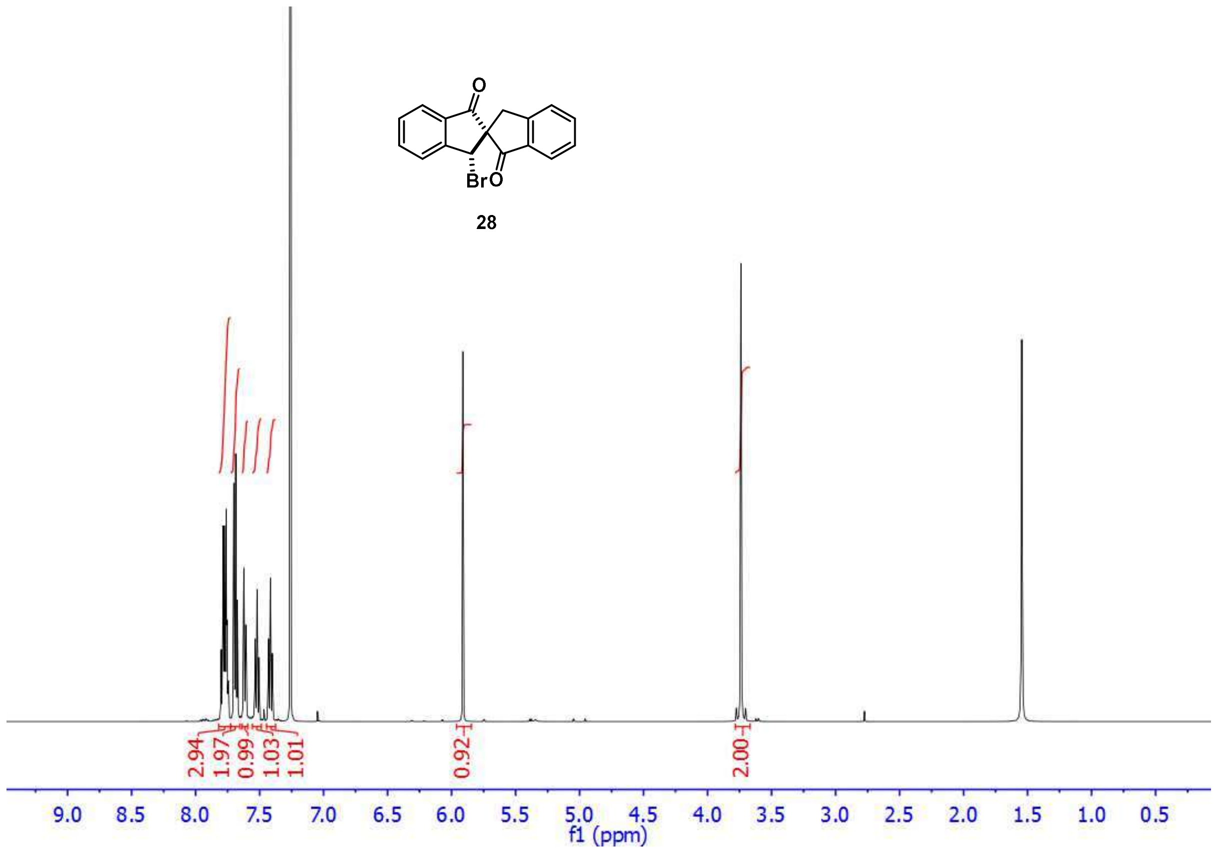
O
Br O
28
2.94
1.97
0.99
1.03
1.01
0.92
2.00
9.0 8.5 8.0 7.5 7.0 6.5 6.0 5.5 5.0 4.5 4.0 3.5 3.0 2.5 2.0 1.5 1.0 0.5
f1 (ppm)

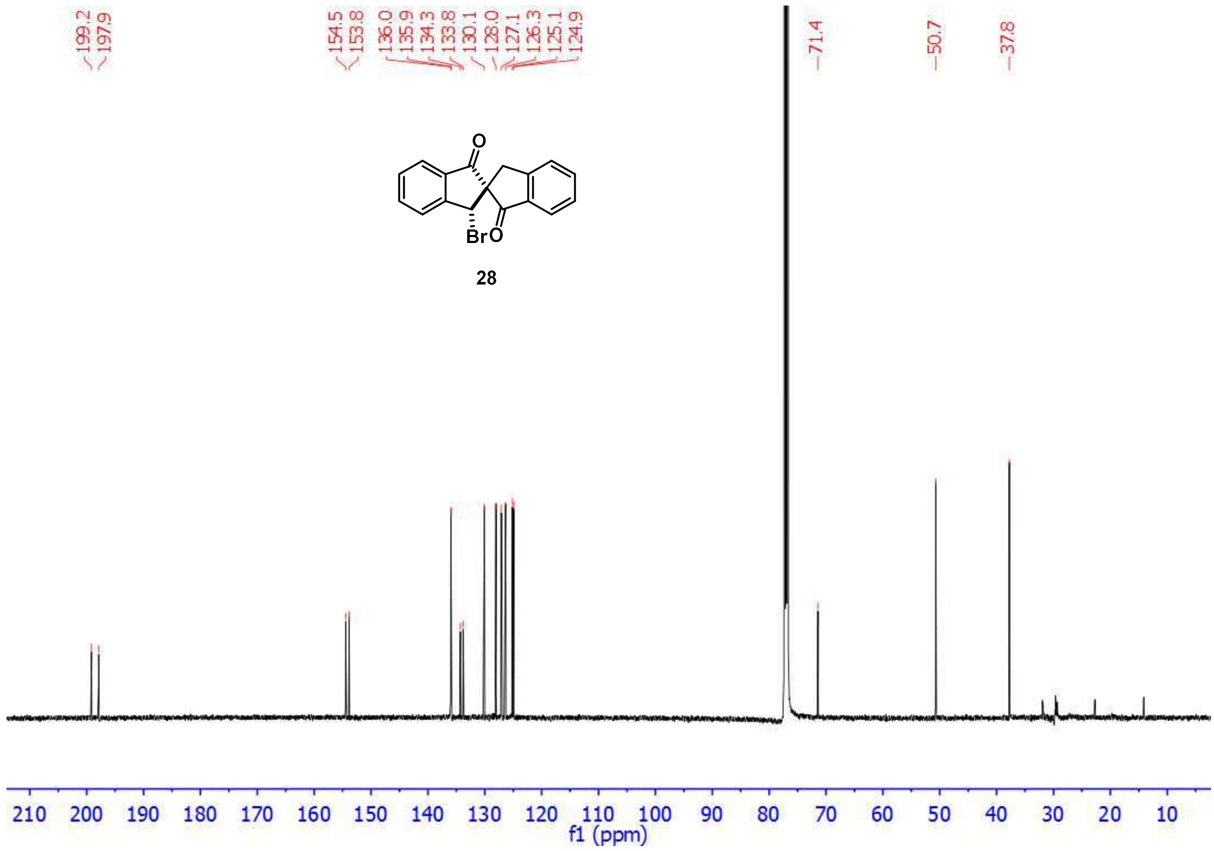
199.2
197.9
154.5
153.8
136.0
135.9
134.3
133.8
130.1
128.0
127.1
126.3
125.1
124.9
71.4
50.7
37.8
O
Br O
28
210 200 190 180 170 160 150 140 130 120 110 100 90 80 70 60 50 40 30 20 10
f1 (ppm)

**Chiral HPLC:** (Chiralpak ADH, 20% isopropanol, 980% hexane, 1.0 mL/min, λ = 250 nm) $\tau_R$ (minor) = 8.0 min, $\tau_R$ (major) = 12.8 min.

**Asymmetric**

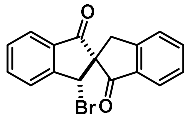


**28**

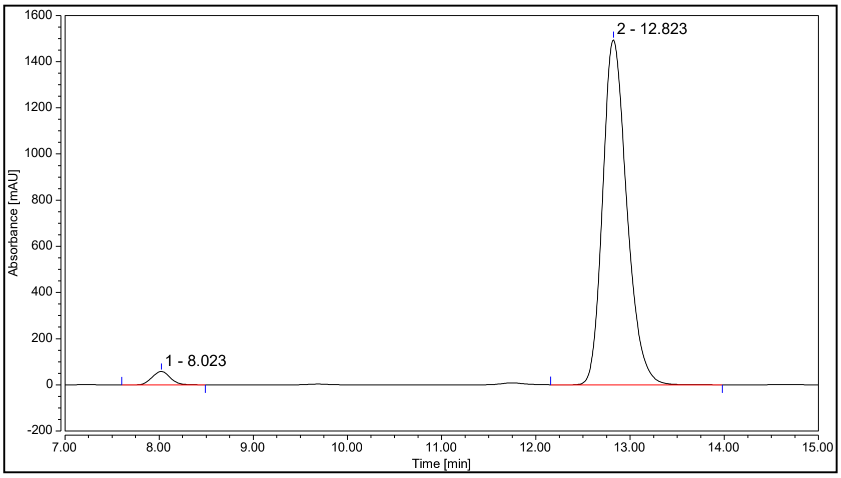


| No. | Peak Name | Retention Time | Area | Height | Relative Area |
|---|---|---|---|---|---|
| | | min | mAU*min | mAU | % |
| 1 | | 8.023 | 12.792 | 58.001 | 2.80 |
| 2 | | 12.823 | 443.257 | 1494.590 | 97.20 |
| **Total:** | | | **456.049** | **1552.591** | **100.00** |

**Racemic**

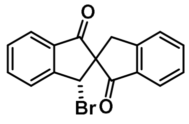


(*rac*)-**28**

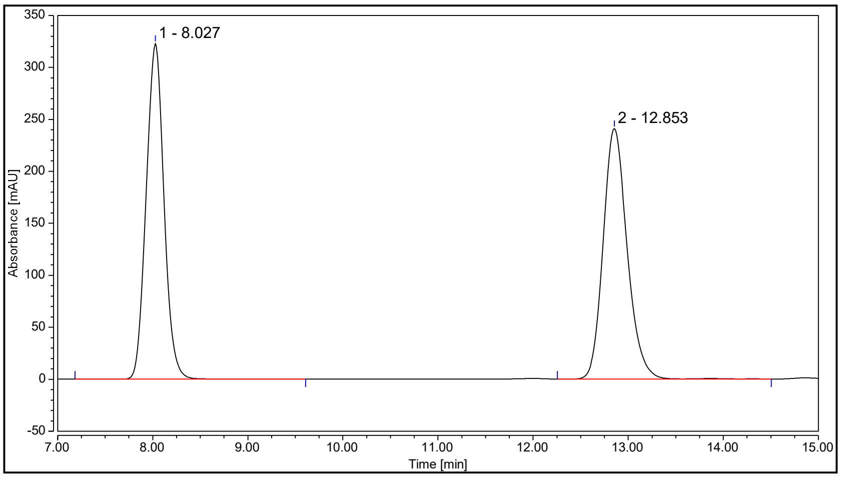


| No. | Peak Name | Retention Time | Area | Height | Relative Area |
|---|---|---|---|---|---|
| | | min | mAU*min | mAU | % |
| 1 | | 8.027 | 70.155 | 322.938 | 49.90 |
| 2 | | 12.853 | 70.425 | 241.033 | 50.10 |
| **Total:** | | | **140.581** | **563.970** | **100.00** |

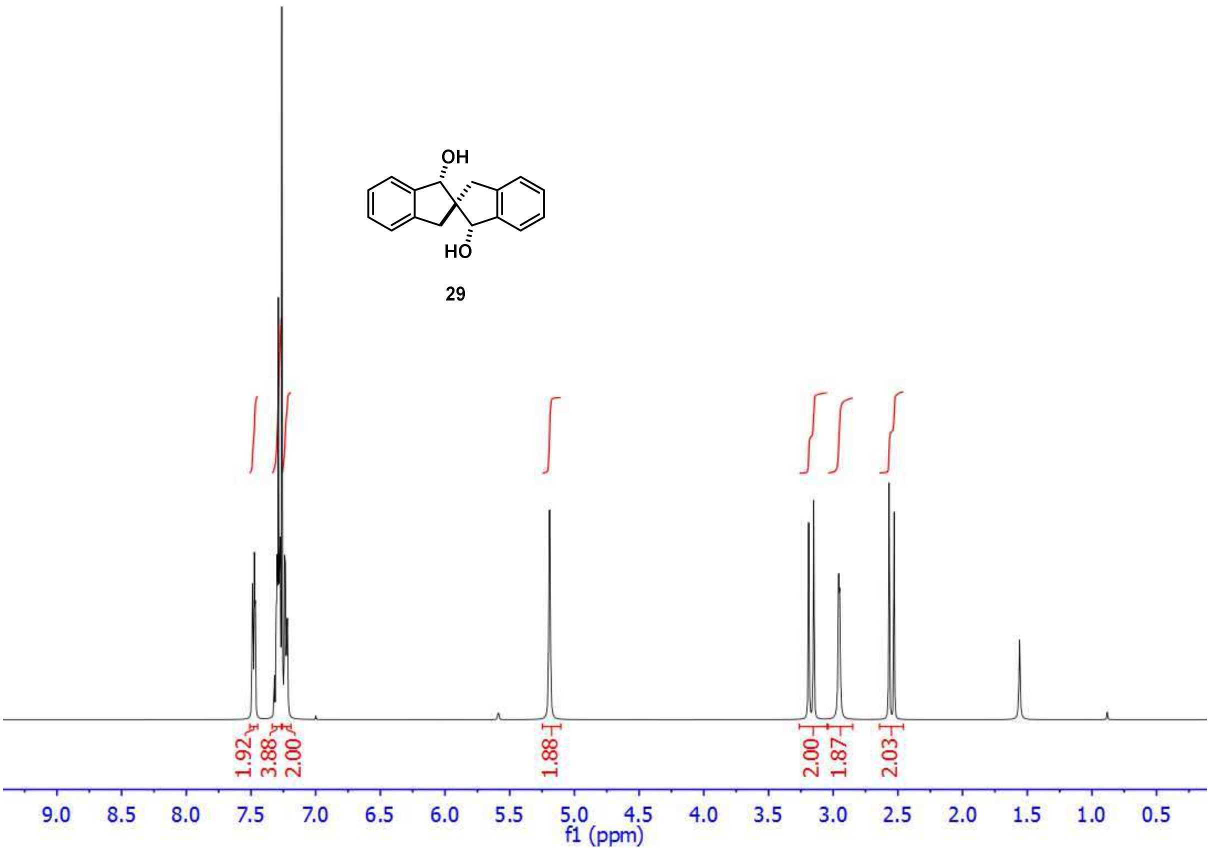

OH
HO
29
1.92
3.88
2.00
1.88
2.00
1.87
2.03
9.0 8.5 8.0 7.5 7.0 6.5 6.0 5.5 5.0 4.5 4.0 3.5 3.0 2.5 2.0 1.5 1.0 0.5
f1 (ppm)


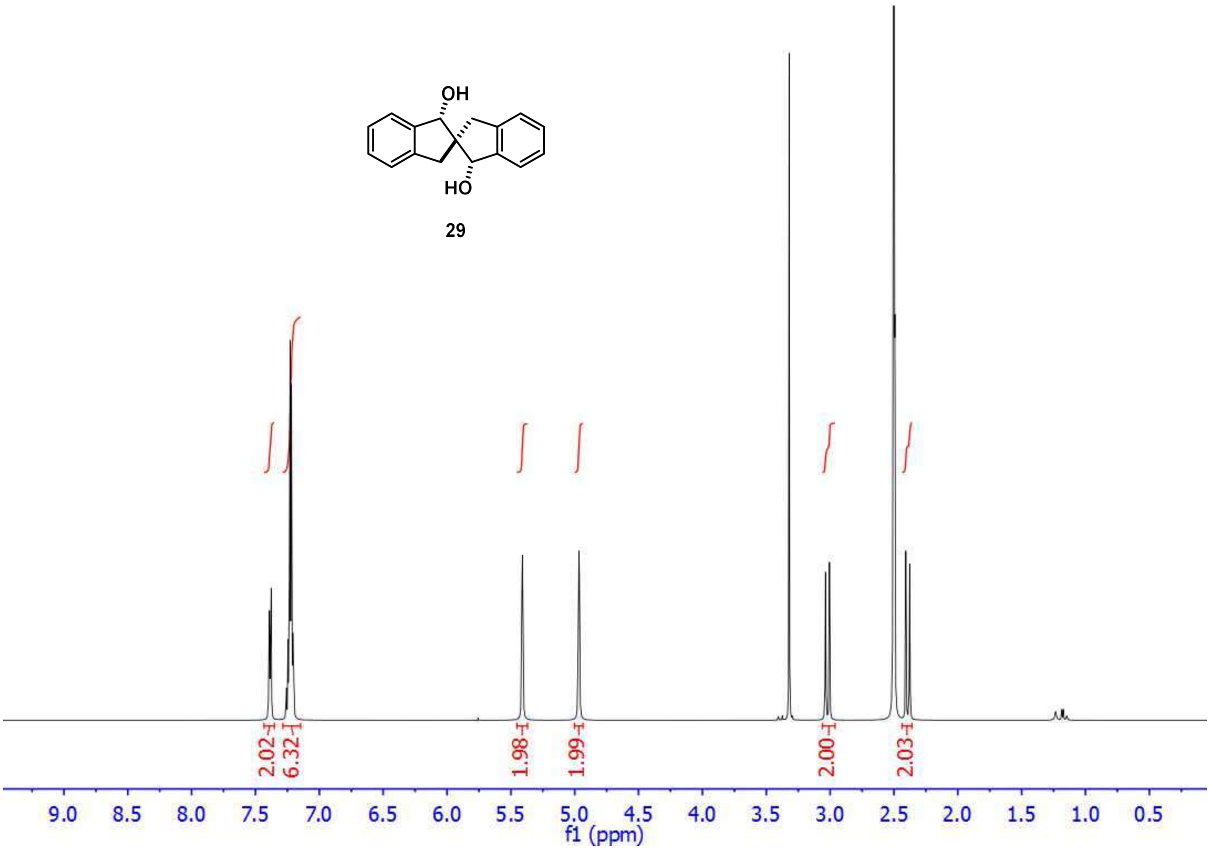

OH
HO
29
2.02
6.32
1.98
1.99
2.00
2.03
9.0 8.5 8.0 7.5 7.0 6.5 6.0 5.5 5.0 4.5 4.0 3.5 3.0 2.5 2.0 1.5 1.0 0.5
f1 (ppm)

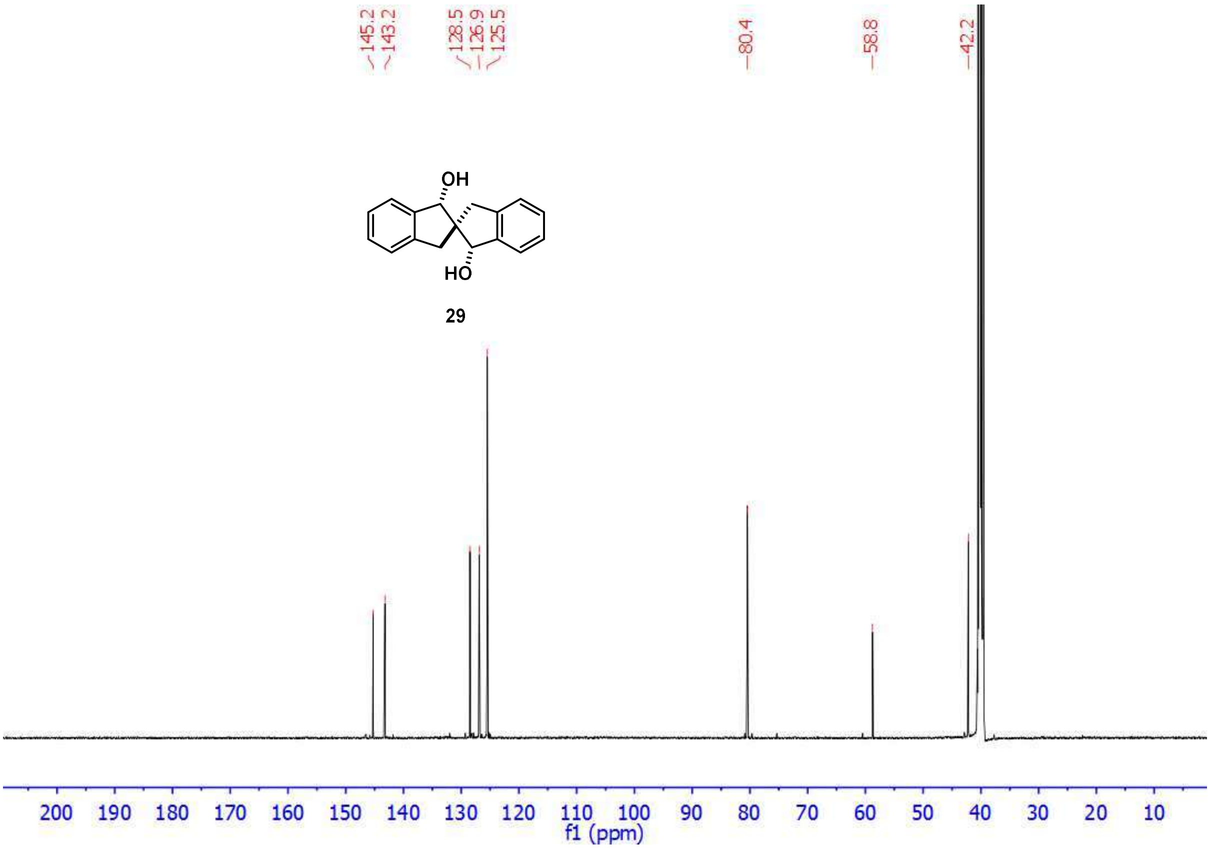

145.2
143.2
128.5
126.9
125.5
80.4
58.8
42.2
OH
HO
200 190 180 170 160 150 140 130 120 110 100 90 80 70 60 50 40 30 20 10
f1 (ppm)


29

**Chiral HPLC:** (Chiralpak AS, 15% isopropanol, 85% hexane, 0.5 mL/min, λ = 273 nm) $\tau_R$ (minor) = 15.0 min, $\tau_R$ (major) = 19.2 min.

## Asymmetric (1)

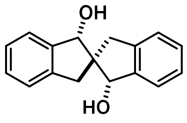


29

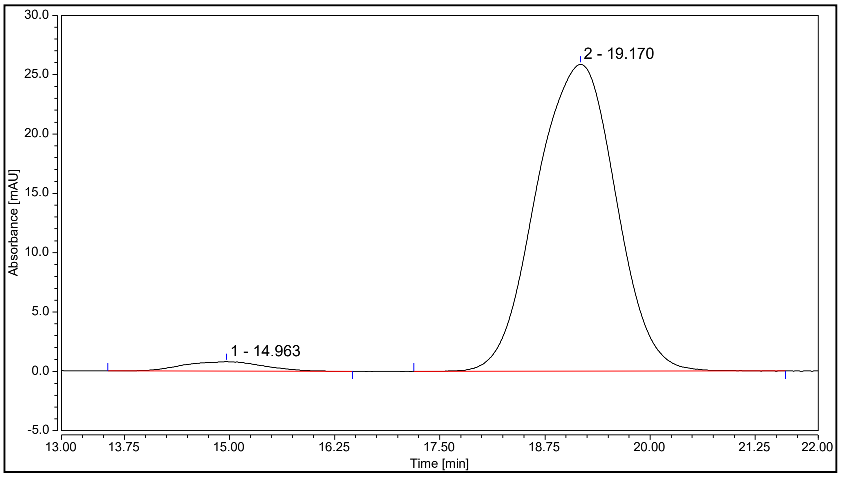


| No. | Peak Name | Retention Time | Area | Height | Relative Area |
|---|---|---|---|---|---|
| | | min | mAU*min | mAU | % |
| 1 | | 14.963 | 0.850 | 0.796 | 2.92 |
| 2 | | 19.170 | 28.270 | 25.834 | 97.08 |
| **Total:** | | | **29.120** | **26.630** | **100.00** |

## Asymmetric (2)

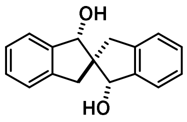


29

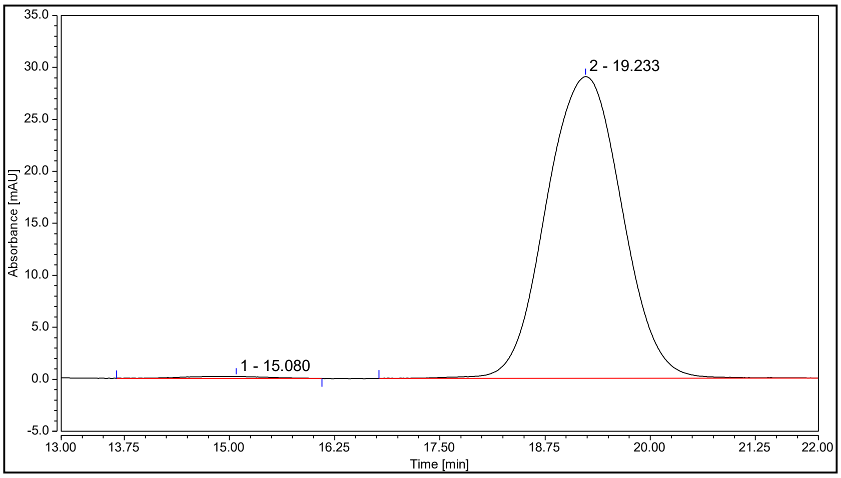


| No. | Peak Name | Retention Time | Area | Height | Relative Area |
|---|---|---|---|---|---|
| | | min | mAU*min | mAU | % |
| 1 | | 15.080 | 0.237 | 0.204 | 0.78 |
| 2 | | 19.233 | 30.257 | 29.017 | 99.22 |
| **Total:** | | | **30.494** | **29.221** | **100.00** |

# Racemic

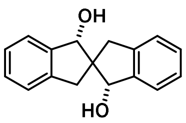

(*rac*)-**29**

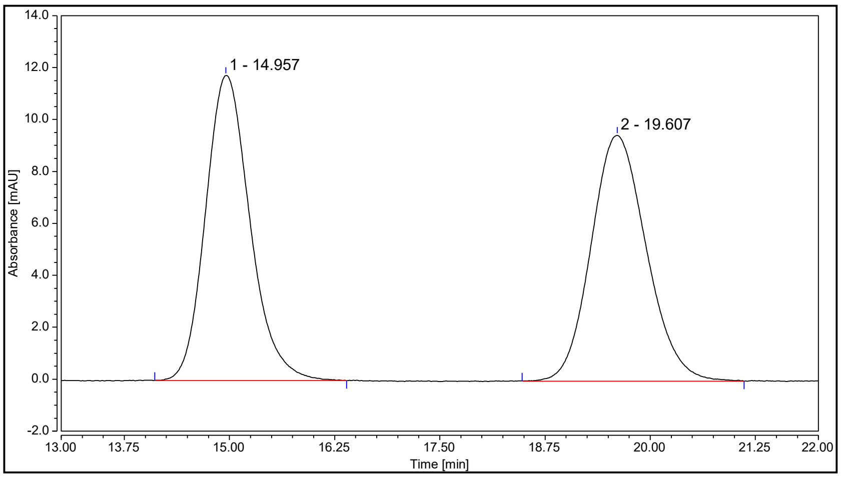


| No. | Peak Name | Retention Time | Area | Height | Relative Area |
|---|---|---|---|---|---|
| | | min | mAU*min | mAU | % |
| 1 | | 14.957 | 7.184 | 11.746 | 49.93 |
| 2 | | 19.607 | 7.205 | 9.464 | 50.07 |
| **Total:** | | | **14.389** | **21.210** | **100.00** |

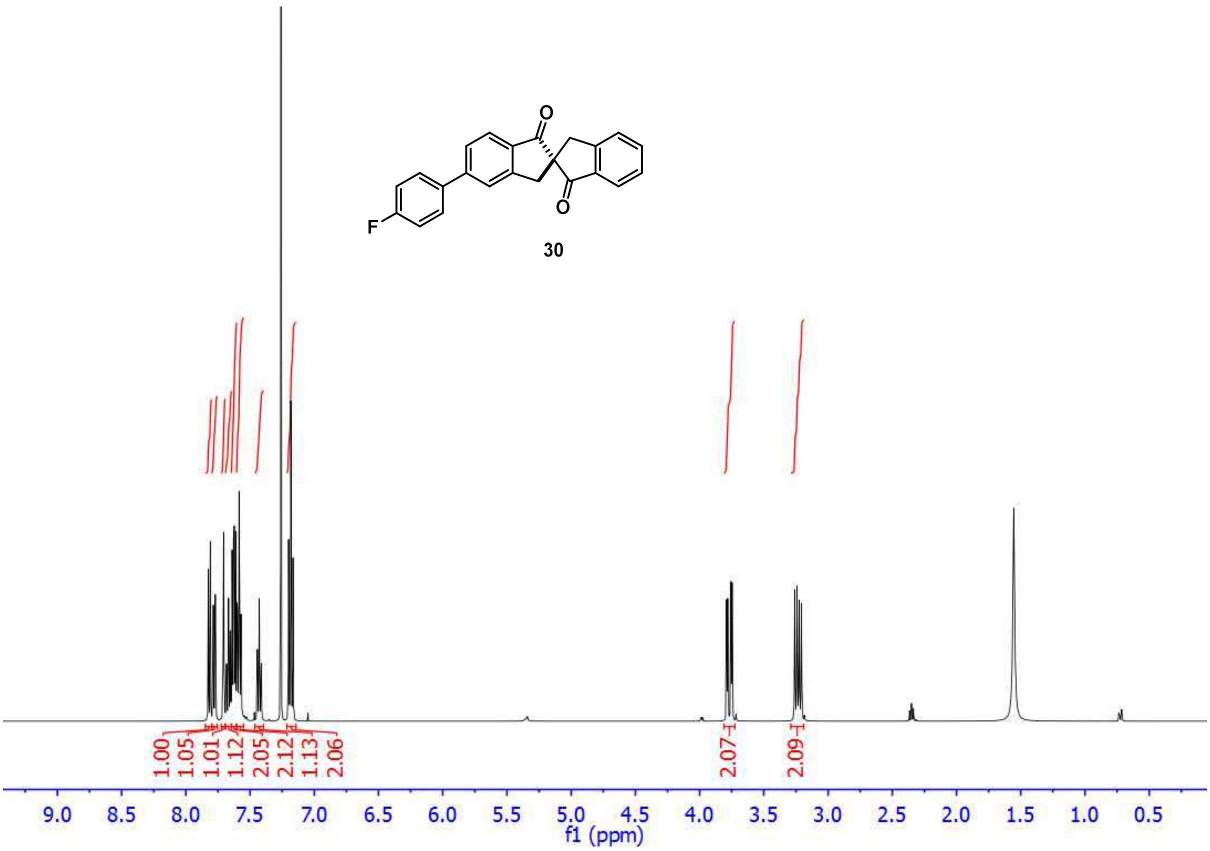

O
F
O
30
1.00
1.05
1.01
1.12
2.05
2.12
1.13
2.06
2.07
2.09
9.0 8.5 8.0 7.5 7.0 6.5 6.0 5.5 5.0 4.5 4.0 3.5 3.0 2.5 2.0 1.5 1.0 0.5
f1 (ppm)


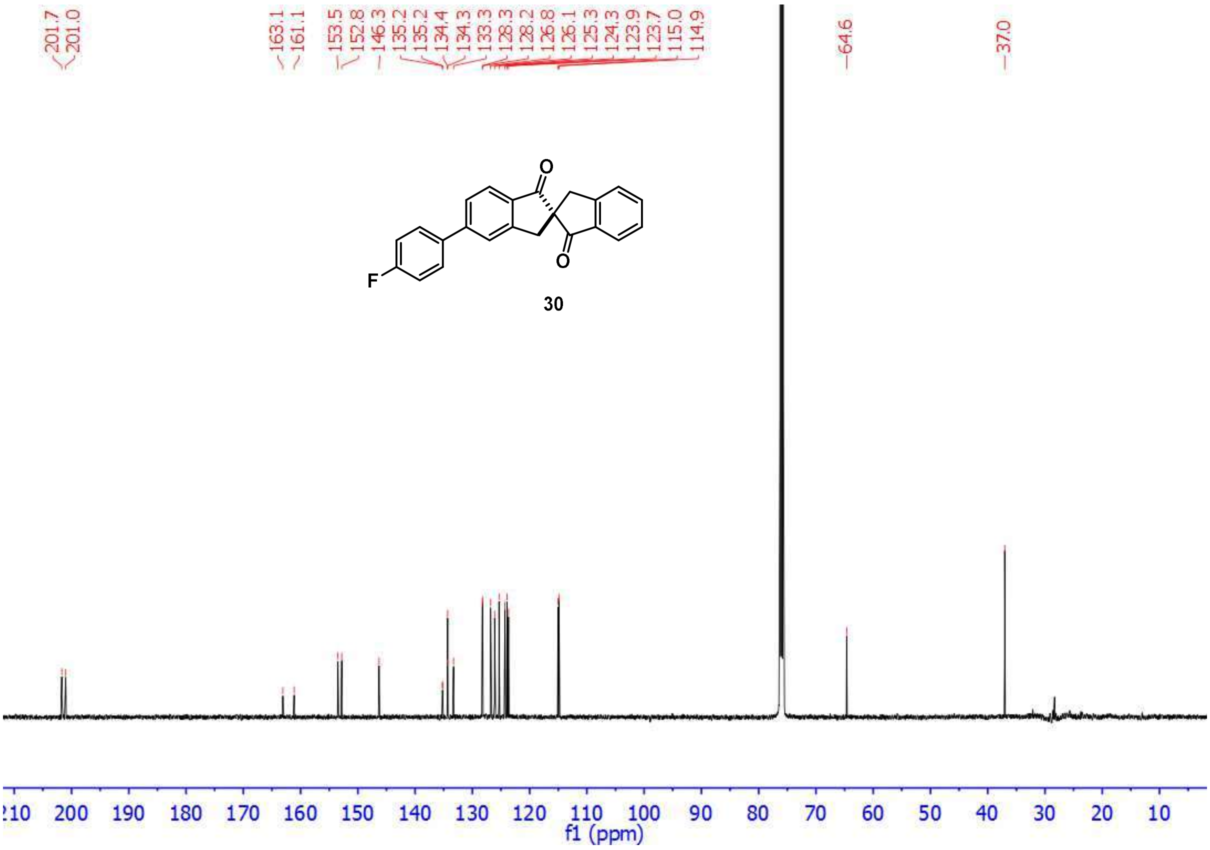

201.7
201.0
163.1
161.1
153.5
152.8
146.3
135.2
135.2
134.4
134.3
133.3
128.3
128.3
128.2
126.8
126.1
125.3
124.3
123.9
123.7
115.0
114.9
64.6
37.0
O
F
O
30
210 200 190 180 170 160 150 140 130 120 110 100 90 80 70 60 50 40 30 20 10
f1 (ppm)

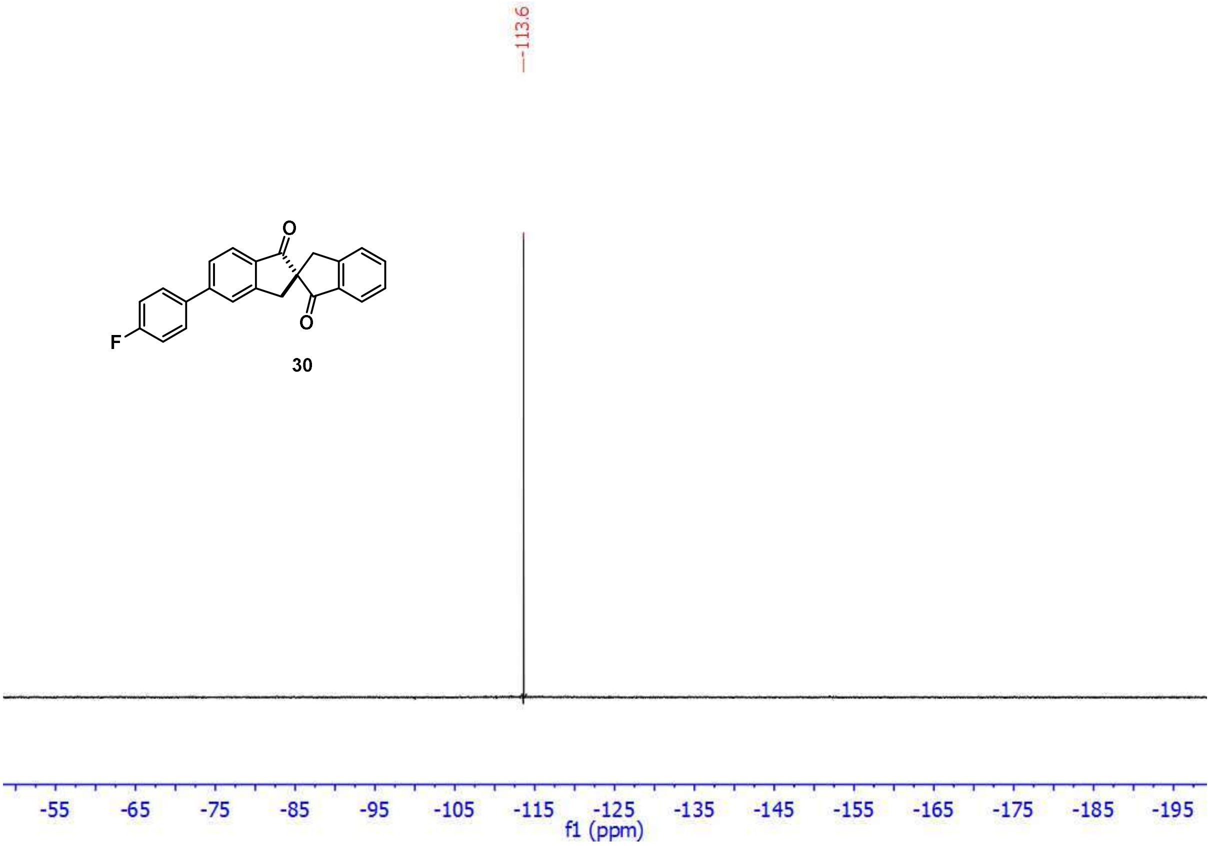
-113.6
O
O
F
30
-55 -65 -75 -85 -95 -105 -115 -125 -135 -145 -155 -165 -175 -185 -195
f1 (ppm)

**Chiral HPLC:** (Chiralpak IA, 20% isopropanol, 80% hexane, 1.0 mL/min, λ = 299 nm) $\tau_R$ (minor) = 15.5 min, $\tau_R$ (major) = 26.8 min.

## Asymmetric

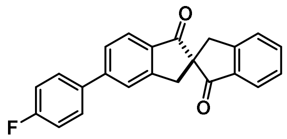


**30**

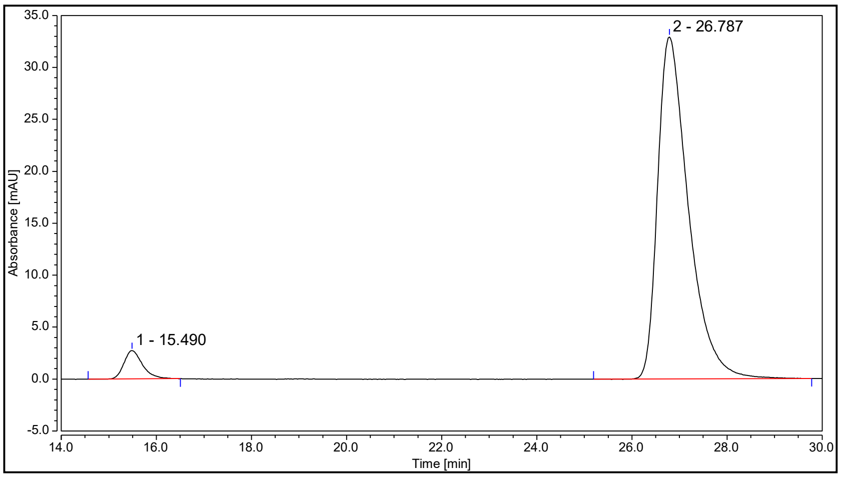


| No. | Peak Name | Retention Time | Area | Height | Relative Area |
|---|---|---|---|---|---|
| | | min | mAU*min | mAU | % |
| 1 | | 15.490 | 1.230 | 2.719 | 4.74 |
| 2 | | 26.787 | 24.715 | 32.924 | 95.26 |
| **Total:** | | | **25.945** | **35.644** | **100.00** |

## Racemic

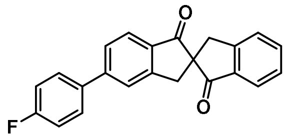


(*rac*)-**30**

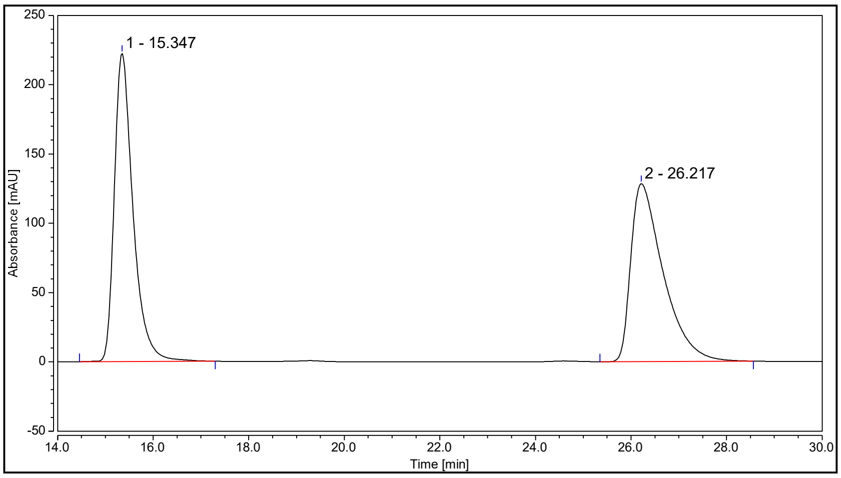


| No. | Peak Name | Retention Time | Area | Height | Relative Area |
|---|---|---|---|---|---|
| | | min | mAU*min | mAU | % |
| 1 | | 15.347 | 100.821 | 222.331 | 50.10 |
| 2 | | 26.217 | 100.433 | 128.312 | 49.90 |
| **Total:** | | | **201.254** | **350.643** | **100.00** |

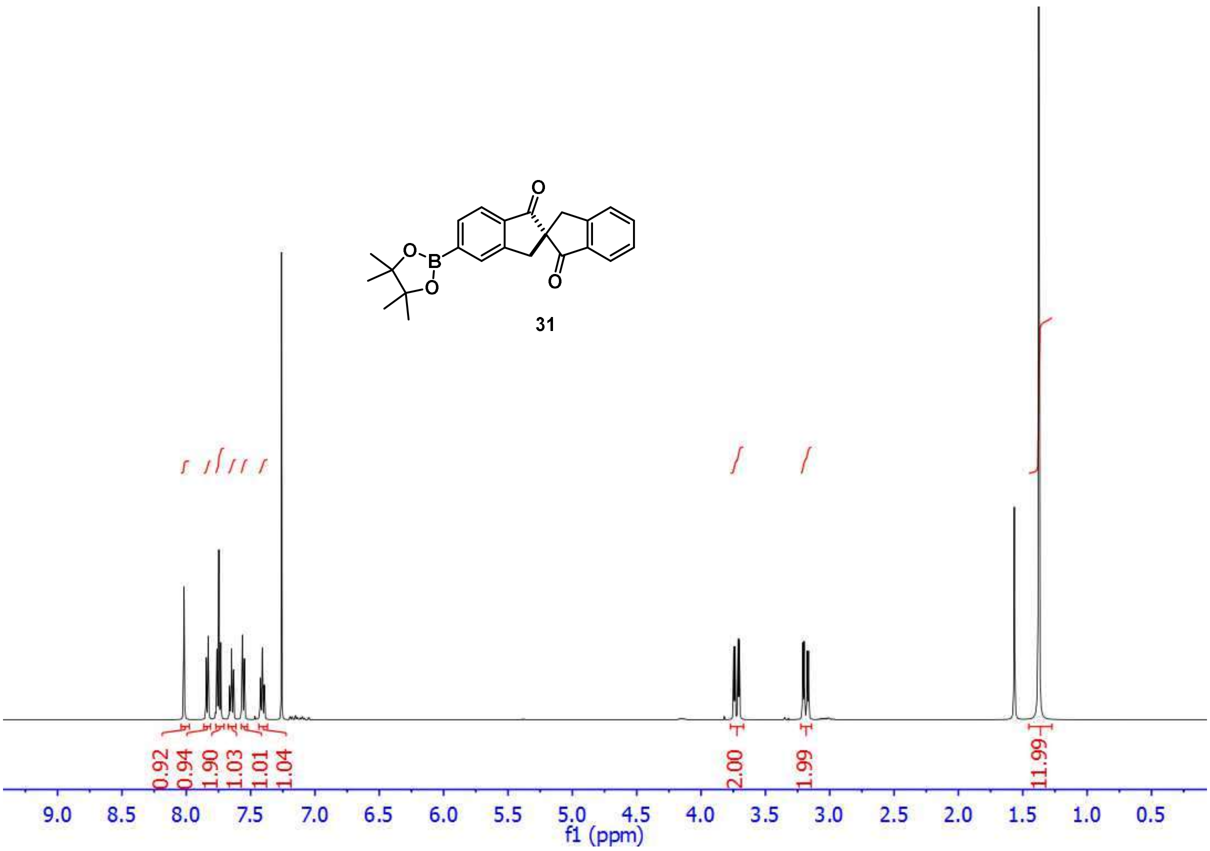
O
B
O
O
O
31
0.92
0.94
1.90
1.03
1.01
1.04
2.00
1.99
11.99
9.0 8.5 8.0 7.5 7.0 6.5 6.0 5.5 5.0 4.5 4.0 3.5 3.0 2.5 2.0 1.5 1.0 0.5
f1 (ppm)

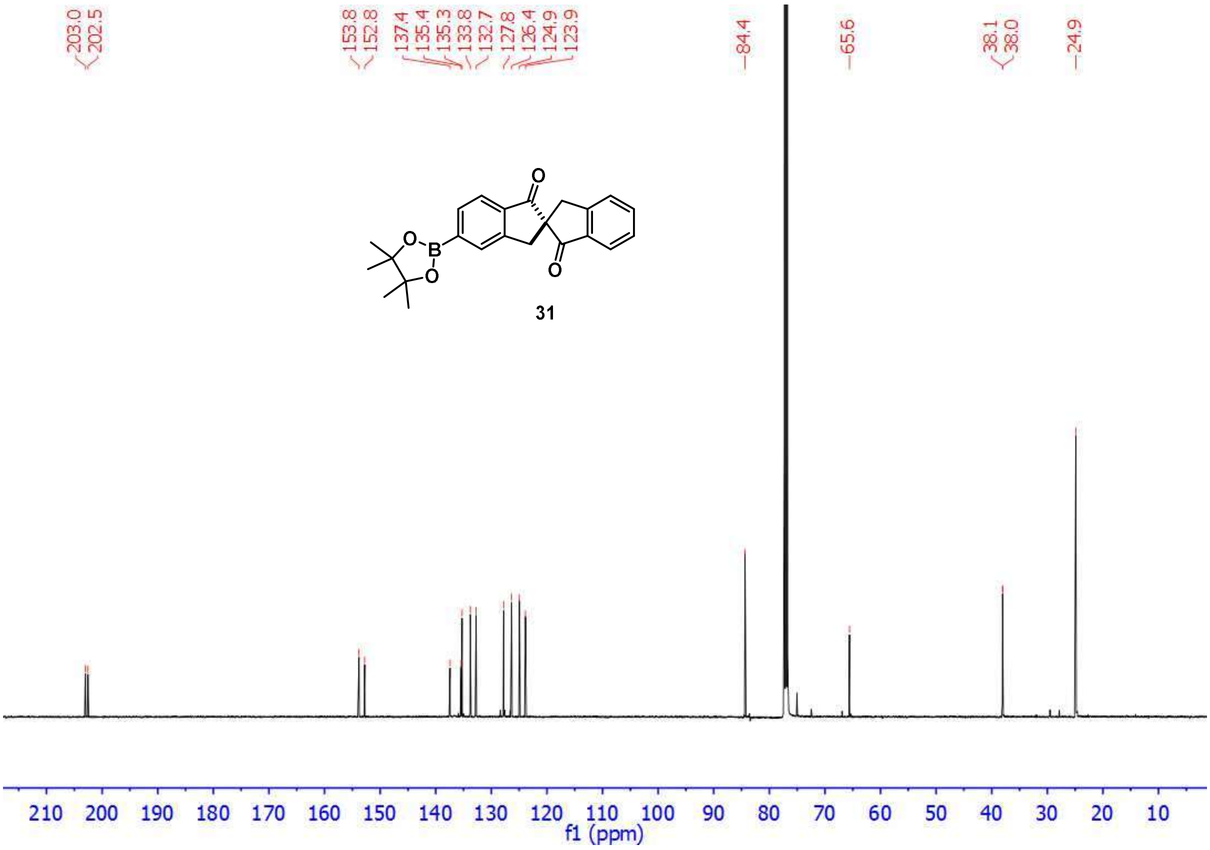
203.0
202.5
153.8
152.8
137.4
135.4
135.3
133.8
132.7
127.8
126.4
124.9
123.9
84.4
65.6
38.1
38.0
24.9
O
B
O
O
O
31
210 200 190 180 170 160 150 140 130 120 110 100 90 80 70 60 50 40 30 20 10
f1 (ppm)

**Chiral HPLC:** (Chiralpak IA, 20% isopropanol, 80% hexane, 1.0 mL/min, λ = 299 nm) $\tau_R$ (minor) = 15.5 min, $\tau_R$ (major) = 26.6 min.

## Asymmetric

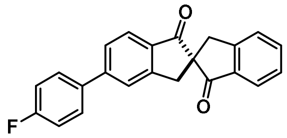


**30** (from **31**)

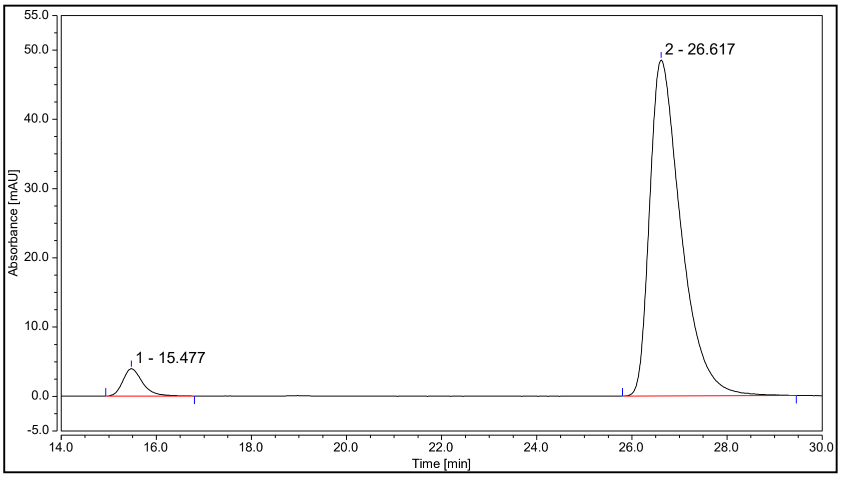


| No. | Peak Name | Retention Time | Area | Height | Relative Area |
|---|---|---|---|---|---|
| | | min | mAU*min | mAU | % |
| 1 | | 15.477 | 1.924 | 3.965 | 4.94 |
| 2 | | 26.617 | 37.033 | 48.515 | 95.06 |
| **Total:** | | | **38.958** | **52.481** | **100.00** |

## Racemic

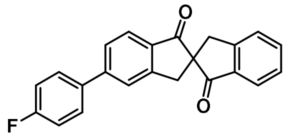


(*rac*)-**30**

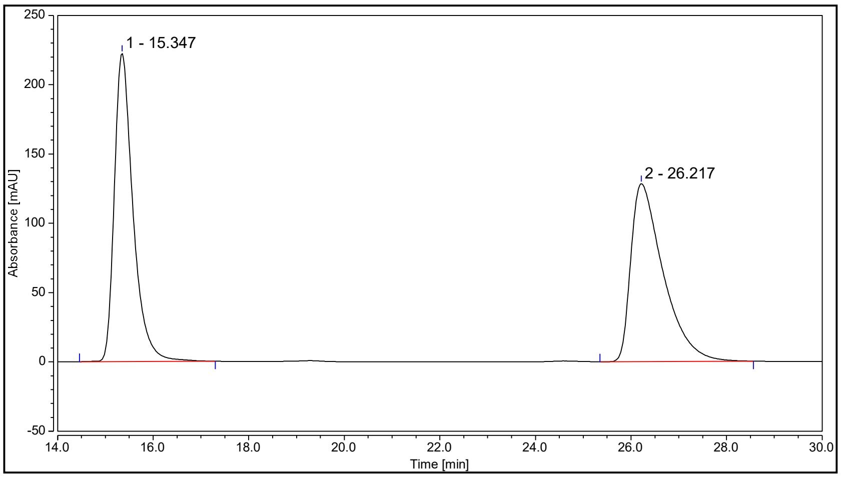


| No. | Peak Name | Retention Time | Area | Height | Relative Area |
|---|---|---|---|---|---|
| | | min | mAU*min | mAU | % |
| 1 | | 15.347 | 100.821 | 222.331 | 50.10 |
| 2 | | 26.217 | 100.433 | 128.312 | 49.90 |
| **Total:** | | | **201.254** | **350.643** | **100.00** |
